# Large-scale molecular phylogeny, morphology, divergence-time estimation, and the fossil record of advanced caenophidian snakes (Squamata: Serpentes)

**DOI:** 10.1371/journal.pone.0216148

**Published:** 2019-05-10

**Authors:** Hussam Zaher, Robert W. Murphy, Juan Camilo Arredondo, Roberta Graboski, Paulo Roberto Machado-Filho, Kristin Mahlow, Giovanna G. Montingelli, Ana Bottallo Quadros, Nikolai L. Orlov, Mark Wilkinson, Ya-Ping Zhang, Felipe G. Grazziotin

**Affiliations:** 1 Museu de Zoologia, Universidade de São Paulo, São Paulo, São Paulo, Brazil; 2 CR2P –Centre de Recherche en Paléontologie – Muséum national d’Histoire naturelle – Sorbonne Université, Paris, France; 3 Centre for Biodiversity, Royal Ontario Museum, Toronto, Ontario, Canada; 4 State Key Laboratory of Genetic Resources and Evolution, Kunming Institute of Zoology, Kunming, China; 5 Laboratório de Herpetologia, Museu Paraense Emílio Goeldi, Belém, Pará, Brazil; 6 Museum für Naturkunde, Leibniz Institute for Evolution and Biodiversity Science, Berlin, Germany; 7 Zoological Institute, Russian Academy of Sciences, Saint Petersburg, Russia; 8 Department of Life Sciences, The Natural History Museum, London, United Kingdom; 9 Laboratory for Conservation and Utilization of Bio-resources, Yunnan University, Kunming, China; 10 Laboratório de Coleções Zoológicas, Instituto Butantan, São Paulo, São Paulo, Brazil; State Museum of Natural History, GERMANY

## Abstract

Caenophidian snakes include the file snake genus *Acrochordus* and advanced colubroidean snakes that radiated mainly during the Neogene. Although caenophidian snakes are a well-supported clade, their inferred affinities, based either on molecular or morphological data, remain poorly known or controversial. Here, we provide an expanded molecular phylogenetic analysis of Caenophidia and use three non-parametric measures of support–Shimodaira-Hasegawa-Like test (SHL), Felsentein (FBP) and transfer (TBE) bootstrap measures–to evaluate the robustness of each clade in the molecular tree. That very different alternative support values are common suggests that results based on only one support value should be viewed with caution. Using a scheme to combine support values, we find 20.9% of the 1265 clades comprising the inferred caenophidian tree are unambiguously supported by both SHL and FBP values, while almost 37% are unsupported or ambiguously supported, revealing the substantial extent of phylogenetic problems within Caenophidia. Combined FBP/TBE support values show similar results, while SHL/TBE result in slightly higher combined values. We consider key morphological attributes of colubroidean cranial, vertebral and hemipenial anatomy and provide additional morphological evidence supporting the clades Colubroides, Colubriformes, and Endoglyptodonta. We review and revise the relevant caenophidian fossil record and provide a time-calibrated tree derived from our molecular data to discuss the main cladogenetic events that resulted in present-day patterns of caenophidian diversification. Our results suggest that all extant families of Colubroidea and Elapoidea composing the present-day endoglyptodont fauna originated rapidly within the early Oligocene–between approximately 33 and 28 Mya–following the major terrestrial faunal turnover known as the “Grande Coupure” and associated with the overall climate shift at the Eocene-Oligocene boundary. Our results further suggest that the caenophidian radiation originated within the Caenozoic, with the divergence between Colubroides and Acrochordidae occurring in the early Eocene, at ~ 56 Mya.

## Introduction

Determining the phylogenetic affinities within snakes was viewed by many herpetologists in the past as an insurmountable challenge. Underwood [1] expressed his profound frustration with a simple sentence: "I have found snake systematics to be a hard test to intellectual honesty”. Although the phylogenetic affinities of snakes were indeed difficult to determine on morphological grounds, monophyly of some higher-level taxa represent a long-standing consensus. This is the case for the clade Caenophidia, a group of advanced alethinophidian snakes recognized formally by Hoffstetter [2] to accommodate the families Colubridae, Dipsadidae, Hydrophiidae, Elapidae, and Viperidae. Hoffstetter’s Caenophidia was characterized by the absence of a coronoid bone and included the colubrid subfamily Acrochordinae, already known to share several additional derived morphological traits with the remaining caenophidian families [3,4]. The same group of “advanced alethinophidian snakes” was also recognized by Romer [5], who preferred to accommodate them in a newly erected superfamily Colubroidea, equating the latter with Hoffstetter’s concept of Caenophidia. “Acrochordoids” and “colubroids” were only later recognized as two distinct superfamilies within Caenophidia after Groombridge [6,7] argued convincingly that acrochordids were the sister-group of the remaining caenophidians based on a number of synapomorphies derived from the vomeronasal capsule, musculature, hyoid and costal cartilages [8,9,10].

Molecular phylogenies ultimately provided strong support for the monophyly of Caenophidia, and further corroborated more controversial morphological hypotheses, such as the polyphyly of solenoglyphous [11] and proteroglyphous snakes [12]. On the other hand, analyses of molecular evidence also obtained conflicting results for the positions of acrochordids and xenodermids at the base of the Caenophidian tree and highlighted the need of substantial taxonomic changes in order to obtain monophyletic familial level taxa [13–28]. Thus, despite notable advances, many questions regarding the higher-level phylogeny and taxonomy of Caenophidia remain unanswered, and a period of taxonomic instability has seen a number of different, and sometimes contradictory, classification schemes, with none of them being entirely satisfactory (S1 Table).

**Fig 1:**
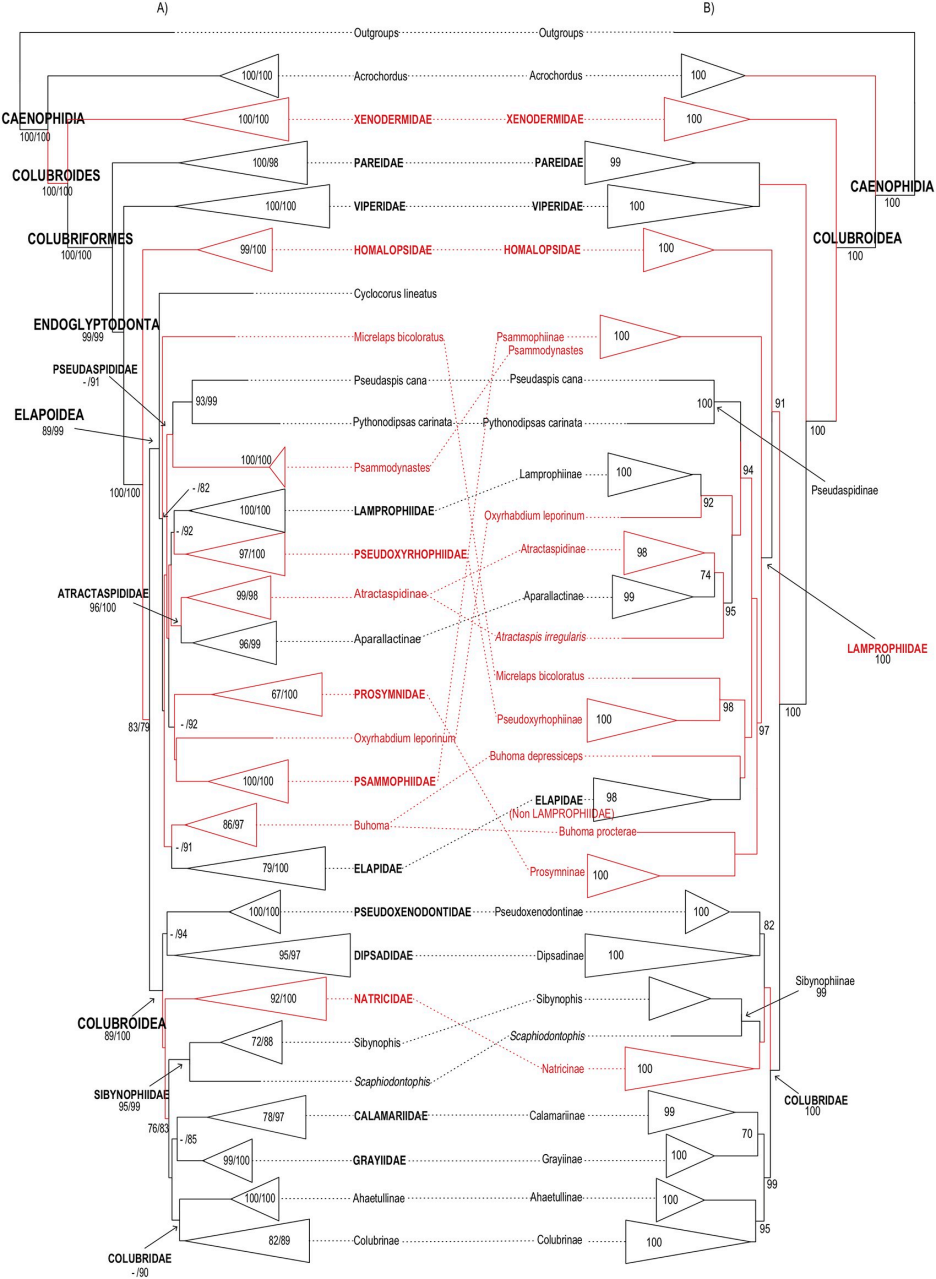
Higher-level caenophidian phylogenies. Comparison between Maximum-likelihood phylogenetic estimates from (A) the present study and (B) Figueroa et al. [28]. Tips represent commonly recognized families, subfamilies and rogue taxa. Names in red correspond to taxa with distinct phylogenetic positions in the topologies compared. Numbers on each branch and within expanded tips correspond to our and previously reported support values: (A) FBP (left) and SHL (right); (B) FBP. Branches without numbers have support <70%.

**Fig 2:**
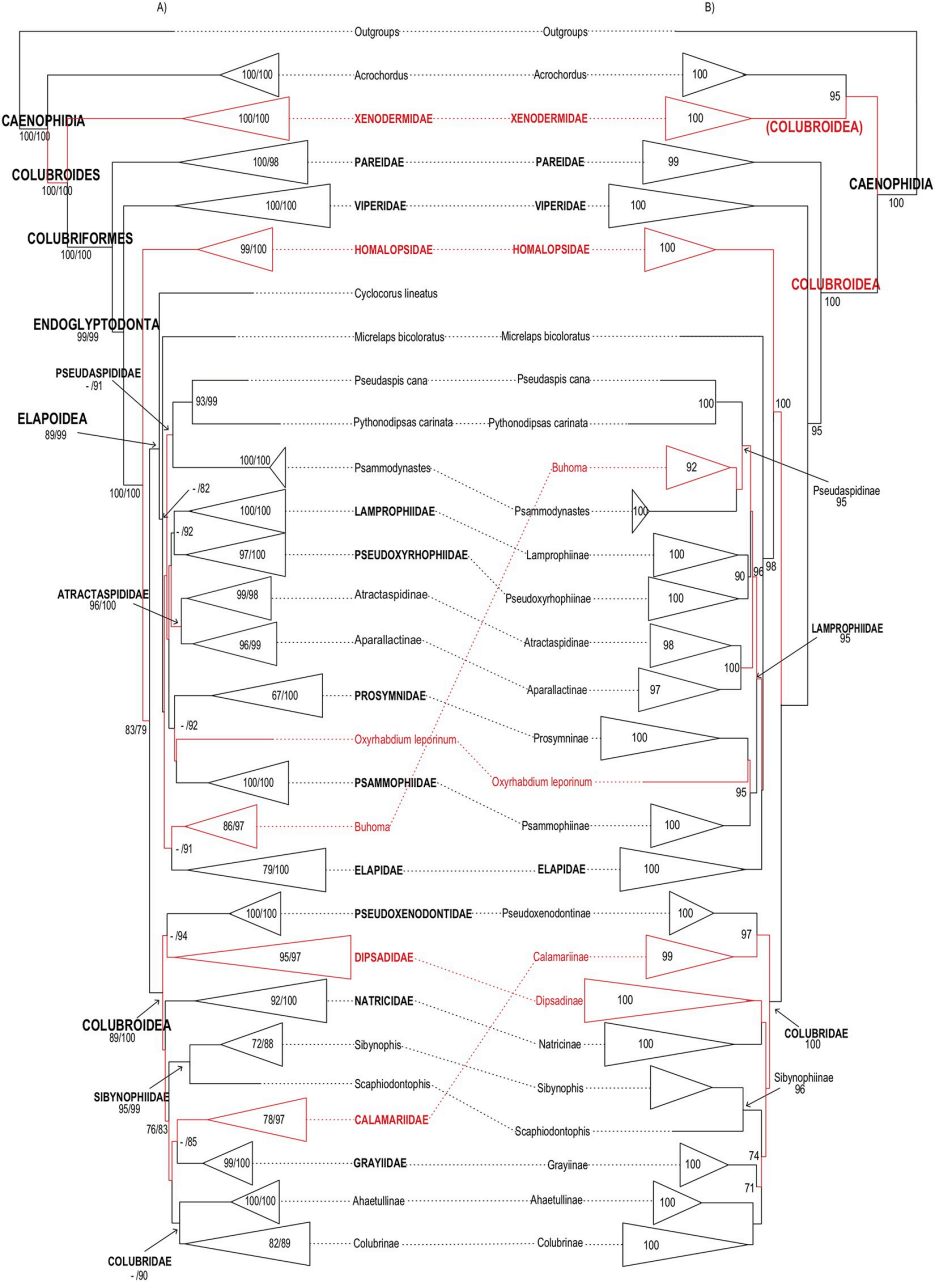
Higher-level caenophidian phylogenies. Comparison between Maximum-likelihood phylogenetic estimates from (A) the present study and (B) Pyron et al. [26]. Tips represent commonly recognized families, subfamilies and rogue taxa. Names in red correspond to taxa with distinct phylogenetic positions in the topologies compared. Numbers on each branch and within expanded tips correspond to our and previously reported support values: (A) FBP (left) and SHL (right); (B) SHL. Branches without numbers have support <70%.

**Fig 3:**
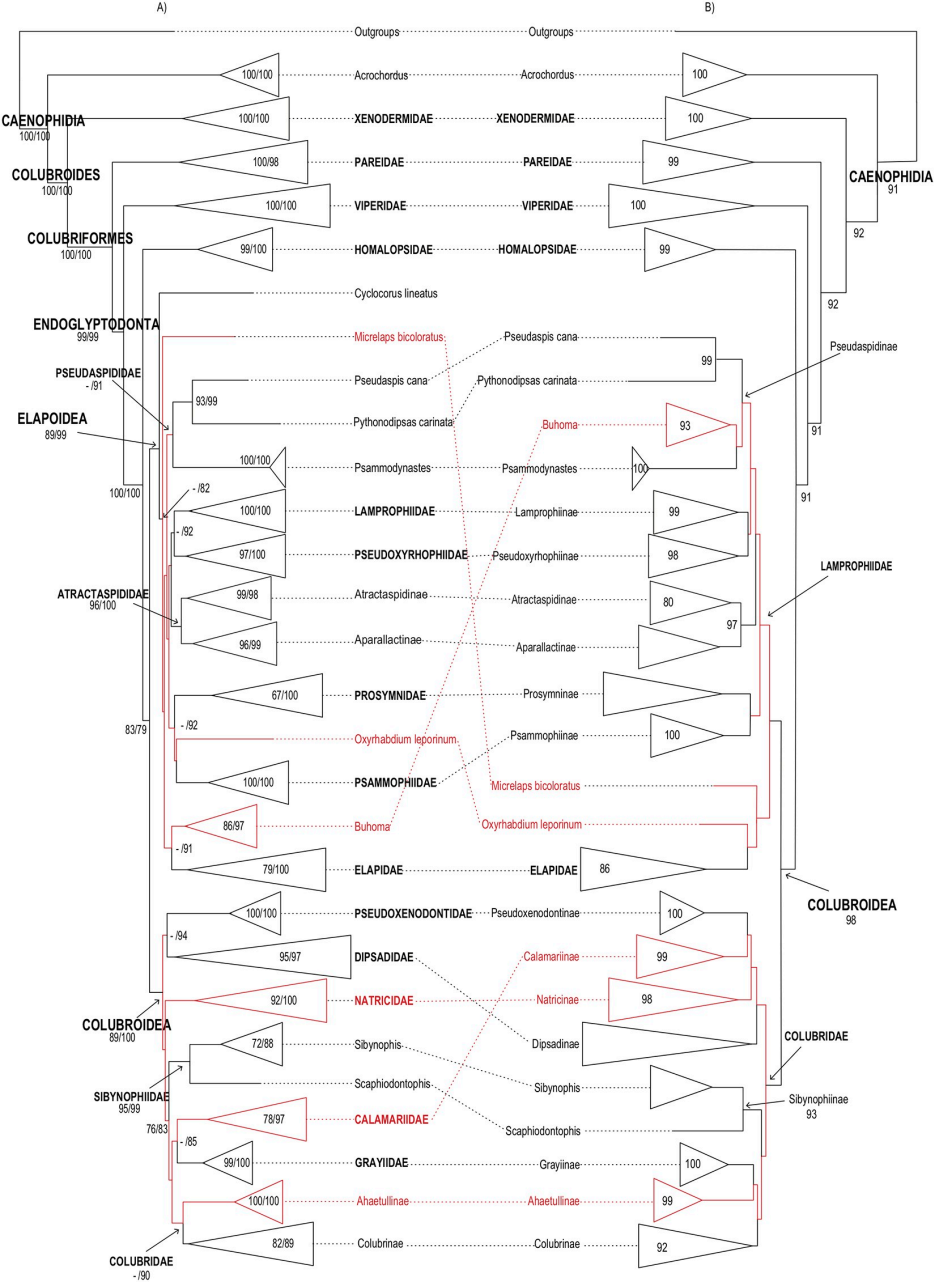
Higher-level caenophidian phylogenies. Comparison between Maximum-likelihood phylogenetic estimates from (A) the present study and (B) Zheng and Wiens [27]. Tips represent commonly recognized families, subfamilies and rogue taxa. Names in red correspond to taxa with distinct phylogenetic positions in the topologies compared. Numbers on each branch and within expanded tips correspond to our and previously reported support values: (A) FBP (left) and SHL (right); (B) FBP. Branches without numbers have support <70%.

Three large-scale molecular phylogenies of snakes were published recently [26,27,28]. However, despite their impressive taxon sampling, substantial overlap in data and similar analytical strategies, these studies have produced a surprisingly large number of differences in inferred relationships at the familial and generic levels (Figs 1–3). Pyron et al. [26] and Figueroa et al. [28] based a number of taxonomic actions exclusively on their molecular phylogenetic analyses with no attempt to reconcile these with the available morphological and paleontological evidence. This is understandable given that one of the main advantages of molecular over morphological phylogenetics is the wider coverage of species that the technique allows within a relatively short amount of time.

Large-sample comparative morphological studies are often difficult to achieve due to the need for destructive investigative procedures on limited museum specimens and by the lack of comprehensive taxonomic coverage in skeletal collections all over the world. Morphological information on caenophidian snake anatomy is still very limited compared to the diversity within the group, with only a few detailed analyses of larger clades being available in the literature and mainly focused on the cranial and hemipenial complexes [11,29]. A notable exception is the monographic study of Cundall and Irish [30] of the skull of snakes, which provides the first comprehensive large-scale comparative analysis of caenophidian cranial anatomy.

The paleontological record for caenophidian snakes is largely biased towards disarticulated postcranial (vertebral) materials [31,32]. Although caenophidian vertebral elements are frequently found in Caenozoic vertebrate-bearing deposits, their identification at the generic and familial levels are often difficult to ascertain, mostly because of our limited knowledge of vertebral morphology and its variation within caenophidian families. However, although limited, our present knowledge on the cranial, vertebral, and hemipenial anatomy of the group still constitutes an important body of evidence that can be evaluated within an explicit molecular phylogenetic framework, helping highlight major events in the origin and diversification of caenophidian snakes. This approach can help circumvent conflicts between multiple alternative molecular hypotheses of relationships [26,27,28] that seem to be correlated with poorly sampled groups or short internal branches combined with terminal taxa with long branches resulting from the accumulation of molecular autapomorphies [21].

Here, we provide an expanded molecular phylogenetic tree of Caenophidia, highlighting strongly and weakly supported hypotheses of relationships that need further investigation. We evaluate alternatively three non-parametric support values–Shimodaira-Hasegawa-Like test (SHL), Felsentein bootstrap proportions (FBP), and transfer bootstrap expectation metrics (TBE)–and combine two of these (FBP and SHL) to give a seven-category classification of the robustness of clades in the molecular tree (S2 Table). Contradictory support values are frequently encountered, suggesting that results based on only one of these three support values, should be viewed with caution. We use our molecular tree as a backbone phylogeny to review some key morphological characters of caenophidian snakes in an attempt to reconcile both morphological and molecular bodies of evidence at the familial and suparfamilial levels of the tree. We focus on two main anatomical complexes in the skull of caenophidian snakes–the optic nerve foramen/fenestra and the naso-frontal joint–known to be phylogenetically informative at higher levels [30]. We also revise anatomical evidence from the vertebrae and hemipenes of representatives of all known extant colubroidean families. Finally, we combine information from the known fossil record and a time-calibrated tree derived from our molecular data to discuss the main cladogenetic events that resulted in present-day patterns of caenophidian diversification.

## Materials and methods

### Taxonomic background

Lawson et al. [18], Zaher et al. [23], and Pyron et al. [26] provided a listing of extant genera considered valid under their family-group names, while Uetz et al. [33] and Wallach et al. [34] went further and compiled complete listings of all extant species. In addition to known extant taxa, Wallach et al. [34] also provided a listing of extinct genera and species. Uetz et al. [33] species list represents a compilation of valid names that follows, in most respects, the latest taxonomic opinions, and thus can be highly unstable. On the other hand, Wallach et al.’s [34] work includes a large number of changes and corrections based on their own critical taxonomic opinion. In that sense, the latter work is a valuable source of original information. However, Uetz et al. [33] taxonomic list seems to integrate more accurately the massive contribution of the Herpetological community in recent years and we use that as a framework to describe our results with respect to valid genera and species. However, we consider both taxonomic schemes at the family level to be problematic in several respects and follow instead the supra-familial and familial taxonomic scheme proposed by Zaher et al. [23,25], expanded here to include recently erected or recognized Pseudoxyrhophiinae, Grayiinae, Prosymnidae, and Pseudaspididae [22,26]. We also followed Savage [35] in the use of the family names Pareidae and Xenodermidae instead of Pareatidae and Xenodermatidae. Recently, Weinell and Brown [36] resolved the phylogenetic affinities of the genera *Cyclocorus* and *Oxyrhabdium*, which along with *Hologerrhum* and *Myersophis*, were retrieved in their analysis as a well-supported elapoid clade of endemic Philippine snakes. Weinell and Brown [36] accommodated these four genera in a new subfamily, Cyclocorinae, further referred herein as a family, Cyclocoridae. As a result, we considered the following 19 families here: Xenodermidae, Pareidae, Viperidae, Homalopsidae, Elapidae, Psammophiidae, Atractaspididae, Pseudoxyrhophiidae, Lamprophiidae, Cyclocoridae, Prosymnidae, Pseudaspididae, Sibynophiidae, Calamariidae, Grayiidae, Colubridae, Pseudoxenodontidae, Dipsadidae, and Natricidae.

The potential taxonomic instability generated by unstable species or species groups representing rogue taxa [37] in molecular analyses is also of concern here. Recently, many of these taxa were given new generic or subgeneric names by R. Hoser who’s approach is considered unethical and potentially harmful for taxonomic stability, resulting in a request by a large consorsium of herpetologists, that the International Commission of Zoological Nomenclature (ICZN) invalidate these new names [38], a petition we strongly support. We refrain from using these names until a definitive decision on the validity of such names is reached by the ICZN.

### Taxon and gene sampling

We assembled a data matrix comprising 1278 (15 outgroup and 1263 ingroup) terminal taxa representing all caenophidian families (see S3 Table for number of genes and accession numbers; see also S4 and S5 Tables for more details on taxon sampling). We obtained 5063 sequences from GenBank and generated 1384 new sequences for up to 15 genes, including six mitochondrial (12S, 16S, *cox1*, *cytb*, *nd2*, *nd4*) and nine nuclear (*amel*, *bdnf*, *c-mos*, *jun*, *hoxa13*, *nt3*, *r35*, *rag1*, *rag2*) loci, for a total of 6447 sequences for 15 genes (S3 Table).

More than 640,000 nucleotide sequences for snakes are currently deposited in GenBank, representing an unparalleled resource for studies of the genetic diversity of the group. However, the quality and reliability of these data are a concern because of misidentification, mislabelling, and sequence contamination which seem to be the principal sources of error present in public databases [39]. In our search for sequences of Caenophidian snakes deposited in GenBank, we found 38 problematic sequences that were not included in the present analysis. All questionable sequences are reported in the supporting information (S6 Table) with succinct descriptions of the possible problems affecting each rejected sequence.

New sequences generated in this study represented 21% of our whole matrix (S3 Table), with more than 50% and up to 80% of new sequences added to previously known sequences for genes *amel*, *bdnf*, *nt3*, *jun*, and *hoxa13*, while no sequences were generated for *cox1*, *nd2*, *nd4*, *r35*, and *rag2* (S3 Table). Newly added sequences are illustrated with colored diamonds on each tip of terminals, representing the percentage of data generated in this study (white, 0%; light gray, between 1% and 50%; dark gray, between 50% and 99%; black, 100%). We did not sample all terminals for all genes, and the percentage of missing terminals varied from 16% for cytb to 93% for amel (S3 Table). The total number of species for families and subfamilies recognized in Uetz et al. [33] are summarized as supporting information (S5 Table). Tissue samples were obtained from museum collections.

According to Uetz et al.’s [33] generic and specific taxonomic listing, our complete taxon sampling of colubroids includes 78% and 42% of all recognized genera and species, respectively, totaling 344 genera and 1263 species (S4 Table). From this total of 1263 species, 20 species are recognized here but not listed by Uetz et al. [33] (S7 Table). According to these slightly revised numbers, summarized in S4 Table, we have the following generic and specific representations in percentage, respectively: Colubridae (77% and 37%), Dipsadidae (81% and 31%), Elapidae (85% and 54%), Homalopsidae (57% and 47%), Lamprophiidae (83% and 42%), Natricidae (68% and 39%), Pareidae (100% and 60%), Pseudoxenodontidae (100% and 40%), Viperidae (97% and 73%), Xenodermidae (67% and 28%).

Outgroup sampling included representatives of the following families (number of terminals in parenthesis): Acrochordidae (3), Aniliidae (1), Boidae (2), Bolyeriidae (1), Calabariidae (1), Cylindrophiidae (1), Erycidae (1), Loxocemidae (1), Pythonidae (1), Uropeltidae (1) and Xenopeltidae (1). Trees were rooted with the typhlopid *Indotyphlops braminus*.

### DNA sequencing

DNA was extracted from scales, shed skin, liver or muscle tissues using the phenol:chloroform method following specific protocols for each tissue [40,41]. PCRs were performed using standard protocols [41] for 11 genes, including four mitochondrial (12S, 16S, *cox1*, *cytb*) and seven nuclear (*amel*, *bdnf*, *c-mos*, *jun*, *hoxa13*, *nt3*, *rag1*). The sequences for each pair of primers and their respective references are provided as supporting information (S8 Table). PCRs were purified with shrimp alkaline phosphatase and exonuclease I (GE Healthcare, Piscataway, NJ). Sequences were generated in Brazil at the Laboratório de Biologia Genômica e Molecular, Pontifícia Universidade Católica do Rio Grande do Sul (Porto Alegre, Rio Grande do Sul) using the DYEnamic ET Dye Terminator Cycle Sequencing Kit in a MegaBACE 1000 automated sequencer (GE Healthcare); and in China at Laboratory for Conservation and Utilization of Bio-resources, Yunnan University (Kunming, Yunnan) using BigDye Terminator cycle sequencing kit in an ABI 3700 sequencer (Applied Biosystems, Foster City, CA). Both strands were sequenced for all fragments and sequences were edited and assembled using Geneious 5.5 (http://www.geneious.com) [42].

### Phylogenetic analysis

Sequences were aligned using MAFFT version 6 [43] applying the E-INS-i algorithm for rRNAs (12S and 16S) and the FFT-NS-i algorithm for protein coding sequences. The scoring matrix for nucleotide sequences was set to 200PAM/k = 2 and gap opening penalty was set to 1,53. Because sequences from GenBank present significant differences in size, we aligned them using a specific procedure that accounts for blocks of overlapping sequences to avoid alignment errors in both extremities of the aligned sequences. Extremities were then realigned using the same algorithm previously applied for each separate gene.

Although our matrix retains high levels of missing data (average of 77.9%), sequences data per taxon range from 286 to 12659 bp with an average of 3299 bp. Similarly, highly incomplete taxa have been argued to be of minor concern in large-scale analyses that include many informative characters [44–45] and, as elsewhere [26], our most highly incomplete taxa were consistently placed in phylogenetic positions that are similar to previous works.

We used PartitionFinder v1.1.1 [46] in order to select a partition scheme and evolutionary models based on AICc. We used the program RAxML version 7.2.8 [47] to perform a phylogenetic analysis employing Maximum Likelihood (ML) as the optimality criterion. We ran 1000 pseudoreplications of non-parametric bootstrap and we calculated FBP [48] using the rapid bootstrap algorithm implemented in RAxML (*-f a*). This also conducts a search for the ML tree using each 5^th^ bootstrap tree as a starting point for the rapid hill-climbing search (totaling 200 starting trees). Based on the 1000 trees derived from the pseudoreplications we calculated the TBE [49] using RAxML-NG [50]. We also calculated branch support using SHL [51] as implemented in RAxML (option–*f E*) for each branch of the tree.

### Comparing measures of clade support

We compared SHL, FBP, and TBE for clades in our molecular tree and used them in combination to evaluate the robustness of specific clades [48,49,51]. We choose to use only the joint values of SHL and FBP to comment the results and base our discussion because they produced more conservative values than TBE (see results on comparisons of support metrics below for more details). We classified the robustness of each clade in seven categories based on the combined clade supports given by the SHL/FBP pair of support measures. These categories are graphically illustrated as supporting information (S2 Table) and summarized on the upper left corner of figures in the text, and are described as follows: 1) unambiguously supported, when both support methods recover values of 100%; 2) robustly supported, when clade support is not unambiguous, but both methods recover values ≥ 90%, or ≥ 80% in one method and 100% in the other; 3) strongly supported, when clade support does not reach percentages equal to previous categories 1 and 2, but both methods recover values ≥ 80%, or values ≥ 70% in one method and ≥ 90% in the other; 4) moderately supported, when clade support does not reach percentages equal to previous categories 1, 2, and 3, but both methods recover values ≥ 70%; 5) ambiguously supported, when clade support presents highly discrepant values, with < 70% in one method and ≥ 80% in the other method; 6) poorly supported, when clade support presents values < 70% in one method and between 70% and 80% in the other method; 7) unsupported, when clade support presents values < 70% for both methods (S2 Table).

Although recognizing the subjectivity and arbitrariness of the described categories, we apply this approach in order to clearly state our reasoning and to facilitate the description of our general level of confidence in each clade retrieved by our phylogenetic analysis. Based on our seven categories, we suggest two main groups of combined support values: a first one with unquestionable or confident combined support values (categories 1, 2, 3, and 4), and a second one with contradictory or unsatisfactory support values (categories 5, 6, and 7).

We highlight clades with contradictory (ambiguous) FBP/SHL support values because we consider them to be potentially erroneous and thus problematic when used in taxonomy or as presumptions for studies applying phylogenetic comparative methods (traits evolution), estimations of diversification rates (speciation/extinction rates) approaches of historical biogeography (discovery and event-based biogeography) and other methods requiring estimates of phylogeny. These unsupported clades should be treated with caution and either the uncertainty taken into account or their use eschewed altogether.

### Morphological comparisons

We revised key morphological characters from the skull, hemipenis and vertebrae in representatives of most extant caenophidian families. Regarding the skull, we focused in two main anatomical complexes–the optic nerve foramen/fenestra and the naso-frontal joint–known to be phylogenetically informative in caenophidian snakes [30]. In that sense, we do not intend to provide here a thorough revision of colubroidean anatomy, and prefer to refer to Underwood [1], McDowell [8], Zaher [29], and Cundall and Irish [30] for a more complete review of the pertinent literature related to these morphological complexes. However, we provide figures of the relevant views of the skulls (S1 Appendix), vertebrae (S2 Appendix), and hemipenes (S3 Appendix) from representatives of most caenophidian groups in order to illustrate the character states discussed herein. Specimens examined are listed in their respective supporting information files (S1–S3 Appendices). Institutional acronyms are as follow: AMNH, American Museum of Natural History, New York; BMNH, The Natural History Museum, London; FMNH, Field Museum of Natural History, Chicago; HUJR, Museum of Zoology, Hebrew University, Jerusalem; IBSP, Instituto Butantan, São Paulo; KU, Museum of Natural History, University of Kansas, Lawrence; LSUMZ, Louisiana State University, Baton Rouge; MNHN, Muséum national d’Histoire naturelle de Paris; MZUSP, Museu de Zoologia, Universidade de São Paulo; ROM, Royal Ontario Museum, Toronto; UMMZ, Museum of Zoology, University of Michigan, Ann Arbor; USNM, National Museum of Natural History, Washington; ZMB, Museum für Naturkunde, Berlin.

### Divergence time estimates

We generated a time-calibrated tree for our complete molecular data set that provided a framework for the interpretation of paleontological, biogeographic, and cladogenetic patterns of caenophidian diversification.

The large size of our molecular matrix (1278 terminals and 15 genes) precluded the use of commonly available parametric uncorrelated relaxed clock methods, as implemented in BEAST [52] and PAML [53]. Instead, we used an autocorrelated relaxed clock method based on a penalized likelihood implemented in the program treePL [54,55]. Divergence times were calculated in treePL by applying a smoothing parameter that defined the penalty for shifting the evolutionary rates among branches. This semiparametric method has been very effective for other data sets with large numbers of taxa [56,57].

To determine the smoothing parameter, we iterated 20 times a cross-validation procedure based on the RSRCV method (random subsample and replicate cross-validation) [55] with lambda values ranging from 0.01 to 100,000 (select lambda = 10) and the *thorough* option to ensure that the run iterates until convergence. We used the multicore option to distribute the cross-validation analyses on 64 processors of a Linux server.

Since treePL only implements uniform prior distributions for node calibration points [54,55], the option of setting an open uniform distribution (by not defining a hard lower bound) can have two main undesirable effects: 1) estimating unrealistic older divergence times; and 2) providing a much larger space for parameter sorting, and thus decreasing the level of convergence of the estimation. In order to avoid these problems, we set maximum ages based on a phylogenetic approach, which takes into consideration the age of relative cladogenetic events. We made the assumption that the maximum age of a specific clade cannot be older than the minimum age of its more inclusive clade. For the purpose of our analysis, two lower bound dates were used to set uniform prior maximum ages: 93.9 Mya. (split between Alethinophidia and Scolecophidia) as the maximum age for our calibration of non-colubroidean nodes, and 54 Mya. (split between Colubriformes and Xenodermidae) as the maximum age for colubroidean nodes.

Calibration points, fossils and constraint dates chosen for our divergence time analysis were as follow:

1. **Alethinophidia stem clade**—*Haasiophis terrasanctus* Tchernov, Rieppel, Zaher, Polcyn & Jacobs, 2000 was set as the Most Recent Common Ancestor (MRCA) of *Anilius scytale* and *Indotyphlops braminus*. The holotype corresponds to a complete, articulated specimen recovered from the Ein Yabrud quarries, near Ramallah, West Bank Palestinian Territories (Hebrew University of Jerusalem Paleontological Collections, HJU-PAL EJ 695). Hsiang et al. [58] combined molecular and morphological data in an unconstrained analysis analysis and reported that *Haasiophis terrasanctus* was a stem-Alethinophidia instead of a crown-Alethinophidia and we adopted their conclusion. New interpretations of several characters have supported this view [58]. Because Hsiang et al.’s [58] analysis incorporates the known phylogenetic uncertainties related to the position of this fossil, we prefer to use *Haasiophis* as a stem-alethinophidian instead of a crown-taxa as traditionally used. The unclear position of the Ein Yabrud quarries within the Cenomanian may fall either in the Early Cenomanian Bet-Meir Formation [59] or the Late Cenomanian Amminadav Formation [60]. We followed Head [61] in using an age range that reflects the uncertainty of the position of Ein Yabrud, by assuming the minimum age set for the Cenomanian [62]. Thus this root-node was set as a hard bound [54], and constrained to 93.9 Mya [61].
2. **Boinae stem clade**—*Titanoboa cerrejonensis* Head, Bloch, Hastings, Bourque, Cadena, Herrera, Polly & Jaramillo, 2009 was set as the MRCA of *Boa constrictor* and *Eryx colubrinus*. The holotype corresponds to one single precloacal vertebra (UF/IGM 1), and referred material includes 185 additional precloacal vertebrae and associated ribs representing 28 individuals from the Cerrejón Coal Mine, Rancheria River Valley, Guajira Peninsula, Colombia [63]. Morphology of the paracotylar foramina and a convex anterior zygosphene margin suggested that *Titanoboa cerrejonensis* belongs to the Boinae [61,63,64]. Head’s [61] preliminary analysis of undescribed cranial elements placed it in the stem lineage of the boine radiation [65]. *Titanoboa cerrejonensis* was recovered from sediments located within the Palynological zone Cu-02 of the Cerrejón Formation, dated as Middle to Late Paleocene [66,67]. Thus, the minimum age was constrained to 58 Mya and maximum age constrained to 93.9 Mya.
3. **Colubriformes stem clade**—*Procerophis sahnii* Rage, Folie, Rana, Singh, Rose & Smith, 2008 was set as the MRCA of *Asthenodipsas vertebralis* and *Achalinus rufescens*. The holotype consists of one posterior precloacal vertebra ("Rana Collection" from Vastan, VAS 1014), and referred material includes five precloacal vertebrae and two caudal vertebrae from the Vastan Lignite mine, Gujarat, India [68]. The lightly built and elongate shape of the precloacal vertebrae, presence of tapering prezygapophyseal processes, and blade-like uniformly thin neural spine that reaches the roof of the zygosphene refer *P*. *sahnii* to the clade Colubriformes. The combination of these characteristics excludes *Procerophis* from an association with the families Acrochordidae, Russellophiidae, Anomalophiidae, and Xenodermidae (S2 Appendix). The differentiated para- and diapophysial articular facets further distinguishes *Procerophis* from russellophiids and anomalophiids. The presence of a plesiomorphic prezygapophyseal morphology, with articular facets predominantly anteriorly angled, supports a basal position within Colubroides [68,69]. The rich squamate fauna from the Vastan Mine of the Cambay Formation was recovered from thin continental lenses of dark claystone and underlying marine shell beds, indicative of a near-shore environment deposited about 1 m above one of the two major Lignite layers (Lignite 2) present in the mine [70]. The squamate layer is situated approximately 14 m below the occurrence of the age-diagnostic foraminiferan *Nummulites burdigalensis burdigalensis* [71,72], indicative of shallow benthic zone SBZ 10 of Middle Ypresian age, which defines a minimum age of ~53 Mya for the deposit [73–75]. However, we here follow [70] in constraining the age of the vertebrate bearing bed of Vastan mine to an early-middle Ypresian (~54 Mya). The occurrence in the section of dinoflagellate cysts of early Ypresian age (~54–55 Mya) [76] and Strontium isotope age estimates for the deposits based on ^87^Sr/^86^Sr values clustering at an age of 54 Mya [77] support this slightly older age. The minimum age was constrained to 54 Mya and maximum age constrained to 93.9 Mya.
4. **Viperidae stem clade**—*Vipera* cf. *V*. *antiqua* [78] was set as the MRCA of *Azemiops feae* and *Causus lichtensteinii*. A moderately well-preserved cervical vertebra (Staatliches Museum für Naturkunde, Stuttgart, SMNS uncatalogued) from Weisenau, Germany. The presence of a long and straight, slightly posteroventrally directed hypapophysis and a large condyle with a ventral portion lying on the posterior margin of the hypapophysis refers unambiguously the cervical vertebra from Weisenau to the family Viperidae. However, its assignment to the "*Vipera aspis* complex" by Szyndlar and Rage [79] is questionable since characters that are known to be diagnostic of the "*Vipera aspis* complex", such as an elongate centrum and short neural spines, are not marked in the cervical region. Additionally, an elongate centrum and short neural spines have been reported in distantly related genus *Causus* [80] and more closely related *Daboia mauritanica* [81]. Furthermore, the traditional subdivision of the "*Vipera aspis* complex", as originally proposed by Saint Girons [82] and detailed by Nilson and Andrén [83] and Herrmann and Joger [84], corresponds to a paraphyletic arrangement of species as evidenced in our analysis (S1 Fig).
  The vertebra from Weisenau, as well as those reported from Saint-Gerand-le-Puy [79], Hessler [85], and Amöneburg [86], are all from the earliest Miocene of Europe (European Land Mammal Age Neogene units MN 1 and MN 2), with the former likely being the earliest record for the family (MN 1). Although the precise age of the Saint-Gerand-le-Puy and Hessler viperids have been ambiguously associated to deposits that may come from either MN1 or MN 2 [85–87], Weisenau vipers are still associated with MN 1 deposits [86]. Thus, the minimum age was constrained to 22.1 Mya (MN 1) based on the Weisenau viperid vertebra. Maximum age was constrained to 93.9 Mya.
5. **Crotalinae stem clade**—Crotalinae gen. & sp. indet. A [88] was set as the MRCA of *Azemiops feae* and *Tropidolaemus wagleri*. A left maxilla with an almost complete tooth preserved in position (Department of Paleozoology, Institute of Zoology, Kiev, Ukraine, IZAN 3748) recovered from karstic fillings within a limestone quarry near the village of Gritsev, Shepetovski district, Ukraine. The maxilla was assigned unambiguously to the Crotalinae due to the deep depression on its posterolateral surface for the accomodation of the thermoreceptive (pit) organ. Holman [89] and Holman and Tanimoto [90] reported possible crown Crotalines from the lower Miocene of the U.S.A. and Japan, respectively. However, despite the fact that there are no known records of viperines in Japan or the New World, the identity of these older records as either crown or stem crotalines cannot be unambiguously determined based on vertebral morphology alone. Thus, we retained the maxilla described by Ivanov [88] as the only unambiguous crotaline record.
  Ivanov [88] reported that the stratigraphic age of the site from Gritsev corresponds to the middle Sarmatian MN 9a of Western Europe, with a minimum age of 10.4 Mya [91,92]. However, Vangengeim and Tesakov [93] argued that Gritsev was more accurately placed within the upper part of the middle Sarmatian, which lies in a zone of reversed polarity that is correlated to chron C5r. Therefore, we used the minimum age of 11.2 Mya estimated for the boundary between the upper and middle Sarmatian and correlated with subchron C5r.1n. The maximum age was constrained to 54 Mya.
6. **Elapidae stem clade**—Elapid Morphotype A [94] was set as the MRCA of *Naja naja* and *Buhoma depressiceps*. One mostly complete posterior trunk vertebra (Tanzanian Antiquities Unit, RRBP 04320) from locality TZ-01, Rukwa Rift Basin, southwestern Tanzania. This specimen was referred to the family Elapidae due to its low and robust hypapophysis and absence of a postzygapophyseal foramen [94]. It also shares a hypapophysis with a flattened and laterally expanded ventral edge with some members of the genus *Naja* [2]. According to McCartney et al. [94], the snake-bearing sites come from fluvial facies that belong to the Songwe Member of the Nsungwe Formation, which is temporally constrained to ~ 24.9 Mya by mammalian biostratigraphy [95–99], detrital zircon geochronology, and a radiometrically dated volcanic ashes [100–102]. Thus, we constrained the minimum age to 24.9 Mya. Maximum age is constrained to 54 Mya.
7. **“Oxyuranine” stem clade**—*Incongruelaps iteratus* Scanlon, Lee and Archer, 2003 was set as the MRCA of *Laticauda semifasciata* and *Cacophis squamulosus*. One mid-trunk (precloacal) vertebra (Paleontological Collection, Queensland Museum, Australia; QM F42691) and referred material, including a right maxilla, a left dentary fragment, 30 vertebrae, and incomplete ribs, from Encore Site, Riversleigh World Heritage Fossil Property, Queensland, Australia [103]. According to Scanlon et al. [103], *Incongruelaps* shares several derived traits with members of the “oxyuranine clade” of Australasian elapids, which includes all marine and terrestrial Australasian Hydrophiinae taxa, except *Laticauda* [22,104]. Riversleigh’s biochronological interval named “Faunal Zone D” [105,106] contains the Encore Local Fauna, which was biocorrelated at approximately 12–10 Mya (Early-Late Miocene) [107,108]. Thus, the minimum age was constrained to 10.0 Mya. Maximum age was constrained to 54 Mya.
8. **Colubroidea stem clade**—Colubrid indet. [109] was set as the MRCA of *Plagiopholis styani* and *Cyclocorus lineatus*. The material referred to Colubrid indet. corresponds to eight trunk and postcloacal vertebrae (Pioneer Trail Regional Museum, Bowman, North Dakota; PTRM 19641) from the Medicine Pole Hills of the Chadron Formation in North Dakota, U.S.A. There is no known vertebral synapomorphy that defines Colubroidea [23], yet the vertebrae from Medicine Pole Hills were assigned to this clade due to the following combination of characters: antero-posteriorly elongated vertebrae (centrum length at least 1.2–1.3 times the width of the neural arch) [109] with elongate prezygapophyseal accessory processes and uniformly narrow, sharp haemal keels in mid- and posterior trunk vertebrae (hypapophyses absent in these vertebrae) [31,69,109,110]. The combination of characters listed above occurs only in colubroid taxa, being absent in elapoids or in more basal colubroideans (i.e., homalopsids, viperids, pareids, and xenodermids), as well as in non-colubroidean caenophidians (i.e., russelophiids, anomalophiids, and acrochordids). Sediments of the Chadron Formation deposited in the hilltops of the Medicine Pole Hills are considered to belong to the Chadronian NALMA age from the upper Eocene [109,111–113]. Minimum age was constrained to 35.2 Mya and maximum age to 54 Mya.
9. **Natricidae crown clade**—*Natrix* aff. *longivertebrata* [114] was set as the MRCA of *Thamnophis atratus* and *Trachischium monticola*. The oldest cranial and vertebral records assigned to *Natrix longivertebrata* are from sediments of La Grive L7, France (MN7, Astaracian, Middle Miocene). These include three parabasisphenoids (Département des Sciences de la Terre, Université Claude Bernard, Lyon, France; UCBL 285075–76, 285452), 374 trunk vertebrae (UCBL 285078–285451), and a left compound bone (UCBL 285077). Rage and Szyndlar [114] and Szyndlar [115] discussed and illustrated the similarities between the cranial morphology of *N*. *longivertebrata* and extant European species of the genus *Natrix*, providing strong evidence for their close affinities. Recently, Pokrant et al. [116] stressed the morphological similarities between the younger type-material of *N*. *longivertebrata* from the Miocene of Gritsev and the extant species *Natrix astreptophora*. No unambiguous synapomorphy supports the allocation of *N*. aff. *longivertebrata* to *Natrix*, yet its cranial morphology places it solidly within the European Natricid radiation [114,115], and we refer it as a Natricidae *incertae sedis*. Several older records also have been attributed to *N*. *longivertebrata* (a fragmentary vertebra from La Grive L3) [115] or to *Natrix* (e.g., *N*. *mlynarskii*, *N*. *merkuriensis*, *N*. *sansaniensis*) [117,118]. However, all of these taxa consisted of either vertebral material or a few inconclusive cranial elements (e.g., compound bone, and quadrate), and none retain well-preserved parabasisphenoids. Therefore, *Natrix* aff. *longivertebrata* is here considered an unquestionable natricid record and is placed within colubroids, as a crown Natricidae. The minimum age was constrained to 13.8 Mya and maximum age to 54 Mya.
10. **Dipsadidae stem clade**—*Paleoheterodon tiheni* Holman, 1969 was set as the MRCA of *Thermophis baileyi* and *Carphophis amoenus*. It consists of a partially preserved and disarticulated skull (University of Nebraska State Museum, Lyncoln, U.S.A.; UNSM 46504) from the Myers Farm Fauna of Nebraska, U.S.A. (Barstovian, Middle Miocene) [119]. Skull elements include well-preserved left frontal and supraoccipital, partially preserved parietal, basioccipital, parabasiphenoid, left quadrate and left compound bone, fragmentary right maxilla, right ectopterygoid, and right pterygoid, and three isolated, blade-like enlarged posterior maxillary teeth. Holman [119] noted that the skull elements referred to *P*. *tiheni* are in many respects distinct from the cranial morphology of members of the genus *Heterodon*. According to Head *et al*. [69], *Paleoheterodon* shows some cranial similarities with *Heterodon*. However, the few features listed by these authors are also present in several other dipsadids [120]. Although there is no compelling evidence supporting a closer affinity of *Paleoheterodon* with either *Heterodon* or *Farancia*, the presence of well-developed blade-like “opisthodont” maxillary teeth [119] corresponds to a derived feature known to occur in several dipsadid tribes, including *Heterodon*. Thus, we refer *Paleoheterodon* to the New World dipsadid radiation, placing it as a stem Dipsadidae. The Myers Farm Fauna has been considered to be equivalent in age to the Valentine Railway Quarries Fauna (late Barstovian Ba2, Middle Miocene) [121–123]. Thus, the minimum age was constrained to 12.5 Mya. Maximum age was constrained to 54 Mya.

## Results

We compare our molecular data and phylogenetic results with three recently published large-scale molecular studies: Pyron et al. [26], Zheng and Wiens [27], and Figueroa et al. [28]). Pyron et al.’s [26] and Zheng and Wiens’ [27] included representatives of all recognised squamatan families, whereas Figueroa et al. [28] focused on snake lineages. Caenophidian coverage in Pyron et al. [26] and Zheng and Wiens [27] were identical (1062 species) and included sequences from up to 12 and up to 52 genes, respectively. Figueroa et al. [28] combined up to 10 genes for 1358 species of caenophidian snakes (excluding multiple individuals, unidentified, and misidentified species). Our study combines sequences from up to 15 genes for 1263 species (see S3 Table for number of genes and accession numbers; see also S4 and S5 Tables for more details on taxon sampling).

Higher-level relationships in these four large-scale studies are illustrated in Figs 1–3 and discussed below. The comparisons among support metrics are discussed below and illustrated in Figs 4 and 5. We also describe and illustrate separately the tree topology we obtained for each well-supported colubroidean family (Figs 6–21). The full tree (including outgroups) is provided as supporting information (S1 and S2 Figs). FBP and SHL, respectively, are provided in parenthesis for each recovered clade discussed below and in Figs 6–21. When applicable, the percentage of valid species sampled for a given genus is also shown in parentheses after the name of the genus (see S4 and S5 Tables for a summary and a list of sampled species per genus).

**Fig 4:**
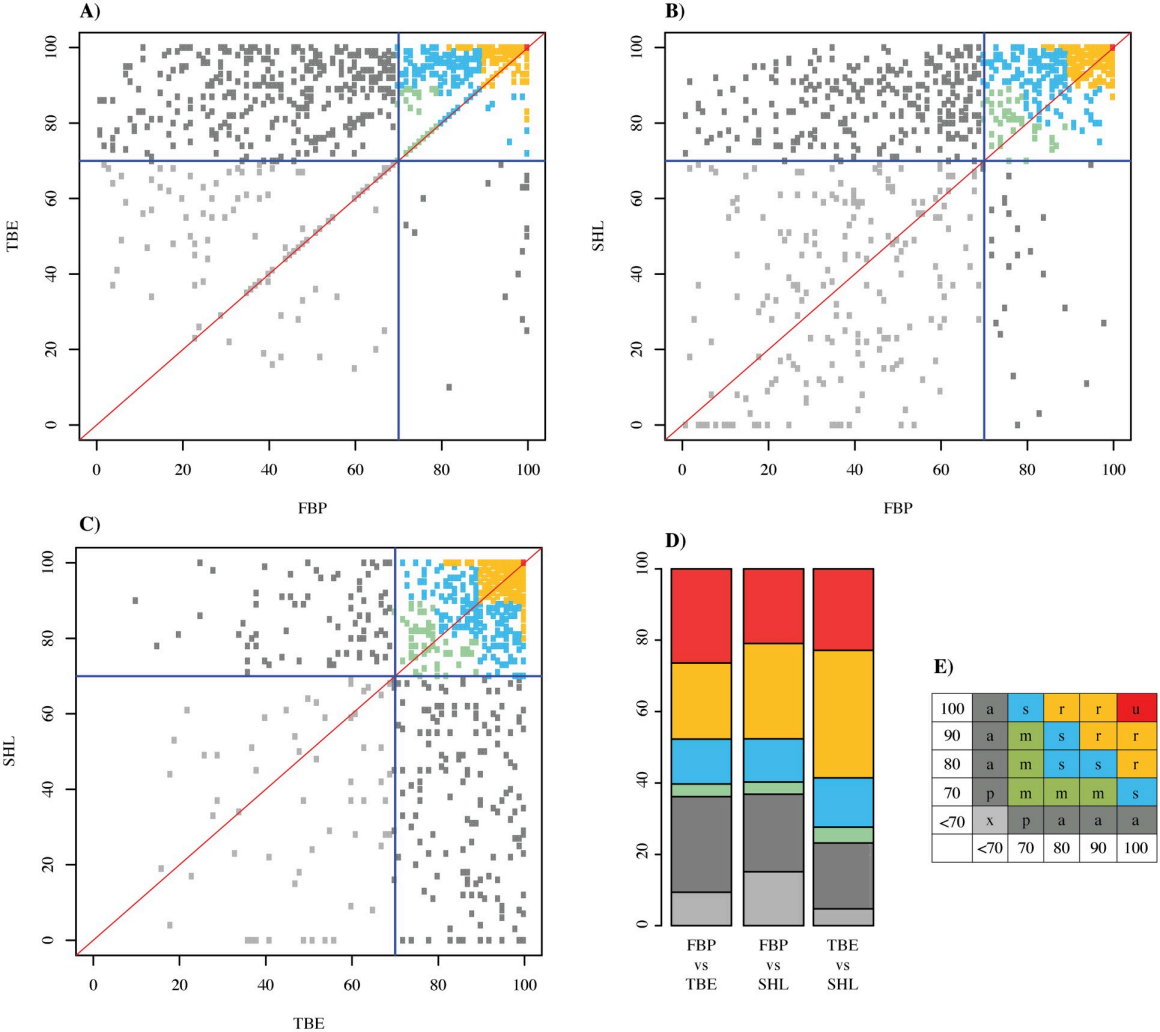
Scatterplots comparing support metrics for internal branches in the Maximum likelihood species-level phylogeny of Colubroides. A) TBE and FBP B) SHL and FBP C) SHL and TBE, D) Histogram showing the proportion of each category of joint support in each comparison of support metrics, E) Categories of joint support.

**Fig 5:**
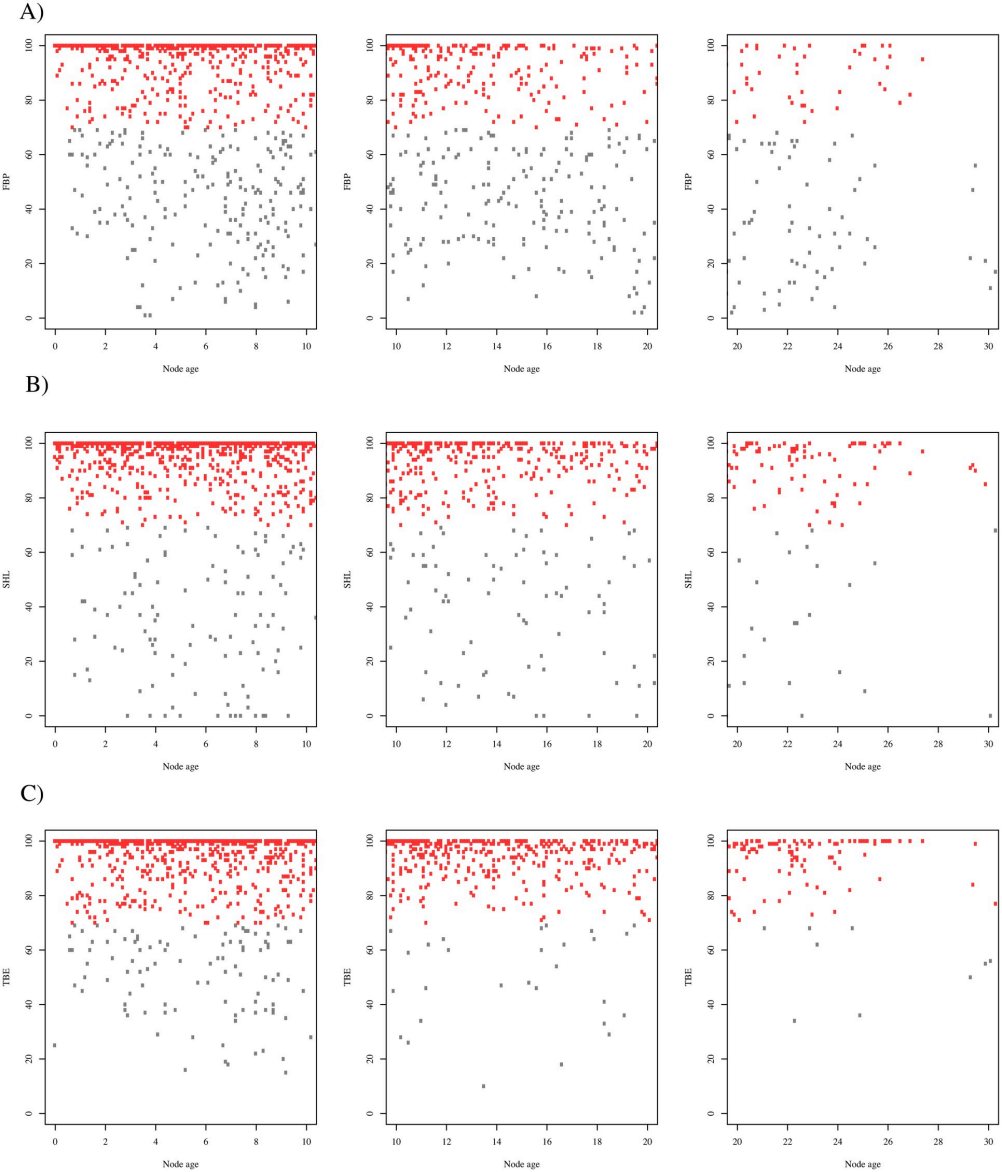
Distribution of branch support scores for each node age based on the Maximum likelihood species-level phylogeny of Colubroides. A) FBP distribution, B) SHL distribution, C) TBE distribution. Red dots represent values greater than 70%; gray dots indicate values smaller than 70%.

**Fig 6:**
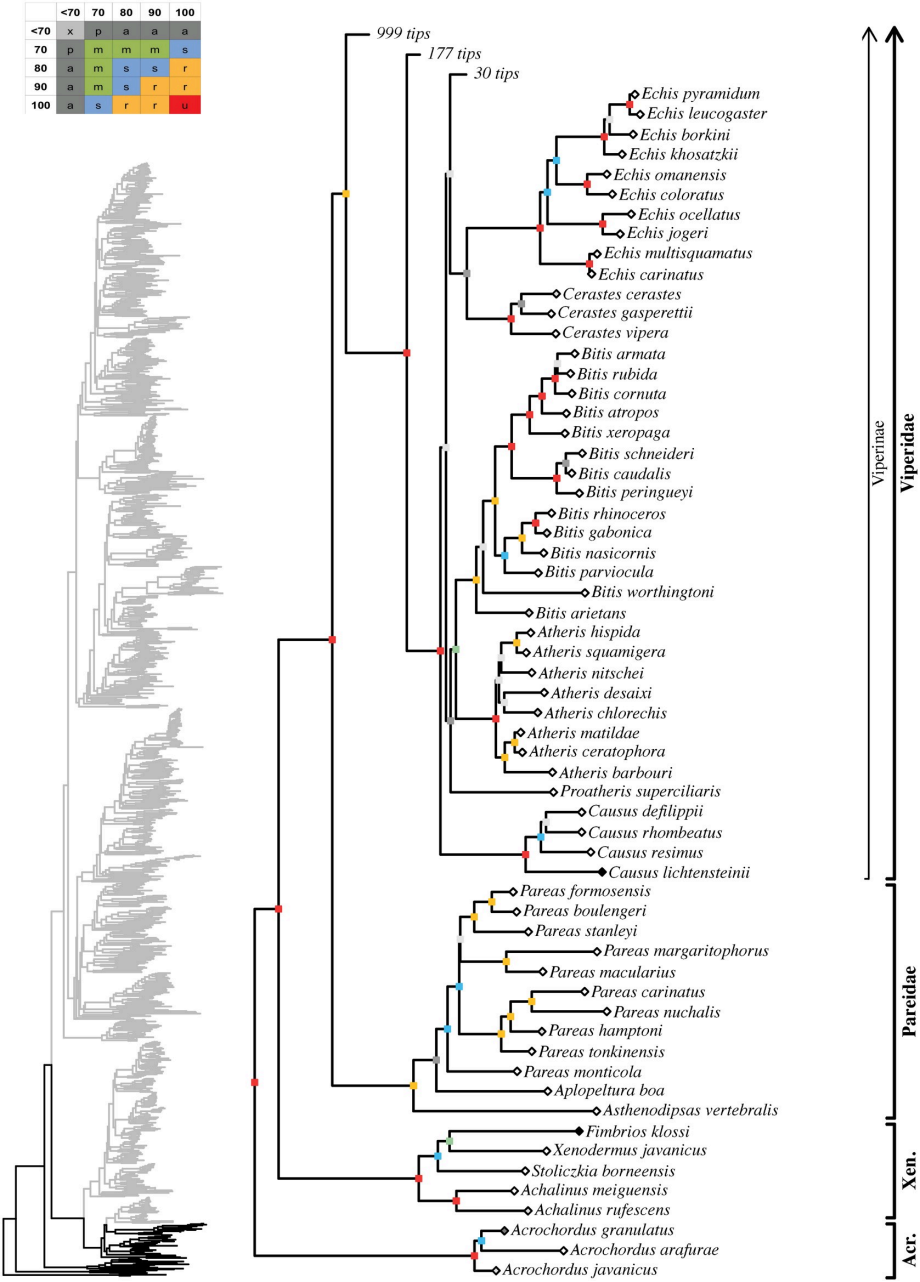
Maximum likelihood species-level phylogeny of Colubroides. Families Xenodermidae, Pareidae, subfamily Viperinae. Skeleton of the complete tree is displayed on the left, with the area of the tree corresponding to the present figure highlighted in black. Colored squares on each node represent bootstrap and SHL values following the categories of combined clade support described in S2 Table and summarized on the upper left corner of the figure. Diamonds on each tip represent the percentage of data generated in this study for each terminal: white, 0%; light grey, between 1% and 50%; dark grey, between 50% and 99%; black, 100%.

**Fig 7:**
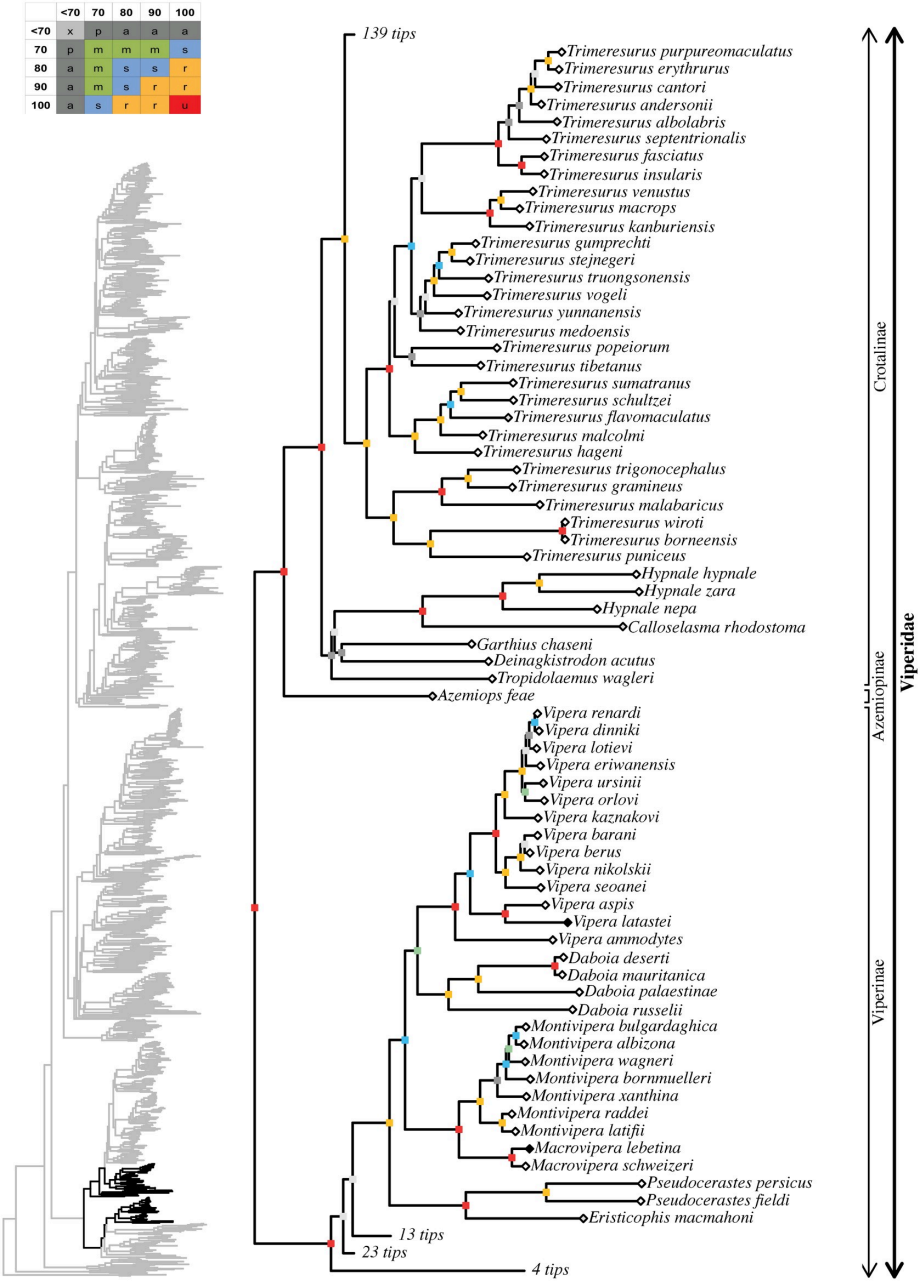
Maximum likelihood species-level phylogeny of Colubroides (continued). Family Viperidae, subfamilies Viperinae, Azemiopinae, Crotalinae.

**Fig 8:**
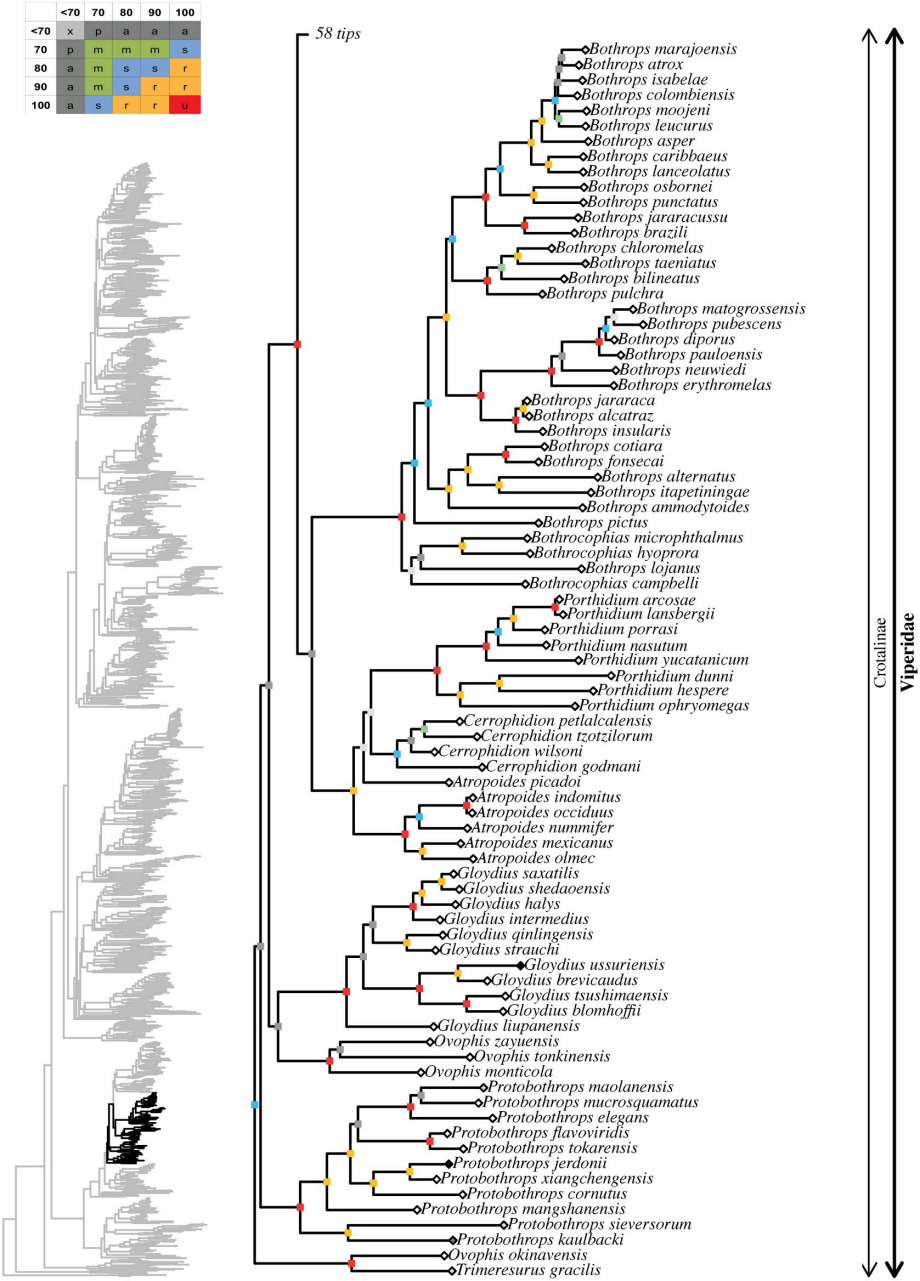
Maximum likelihood species-level phylogeny of Colubroides (continued). Family Viperidae, subfamily Crotalinae.

**Fig 9:**
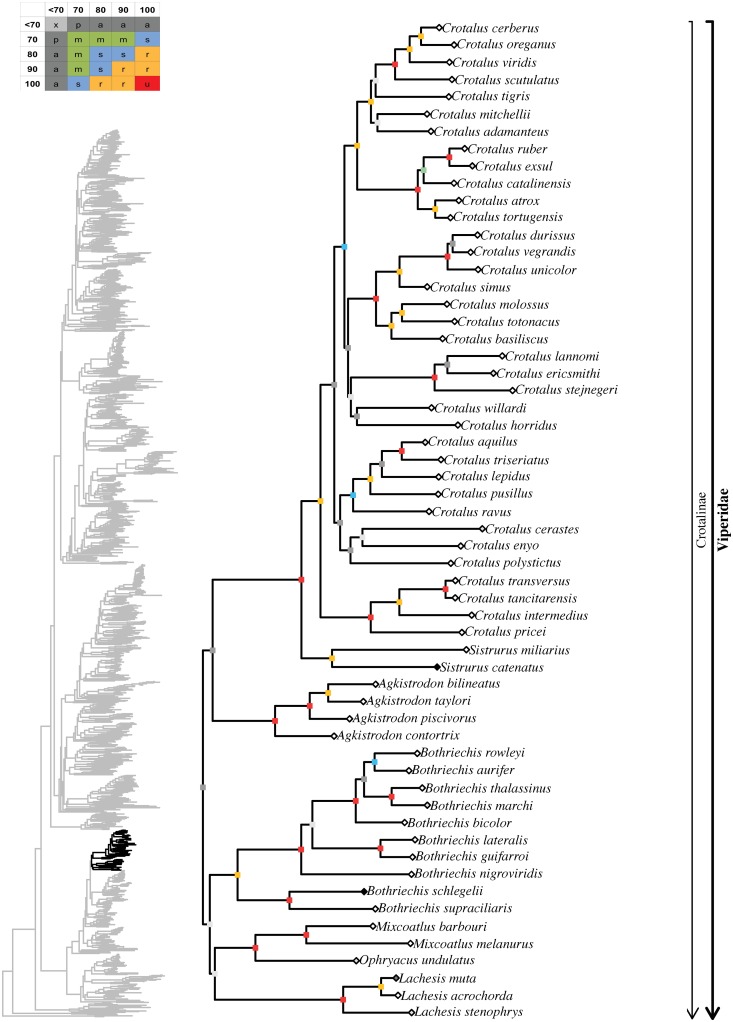
Maximum likelihood species-level phylogeny of Colubroides (continued). Family Viperidae, subfamily Crotalinae.

**Fig 10:**
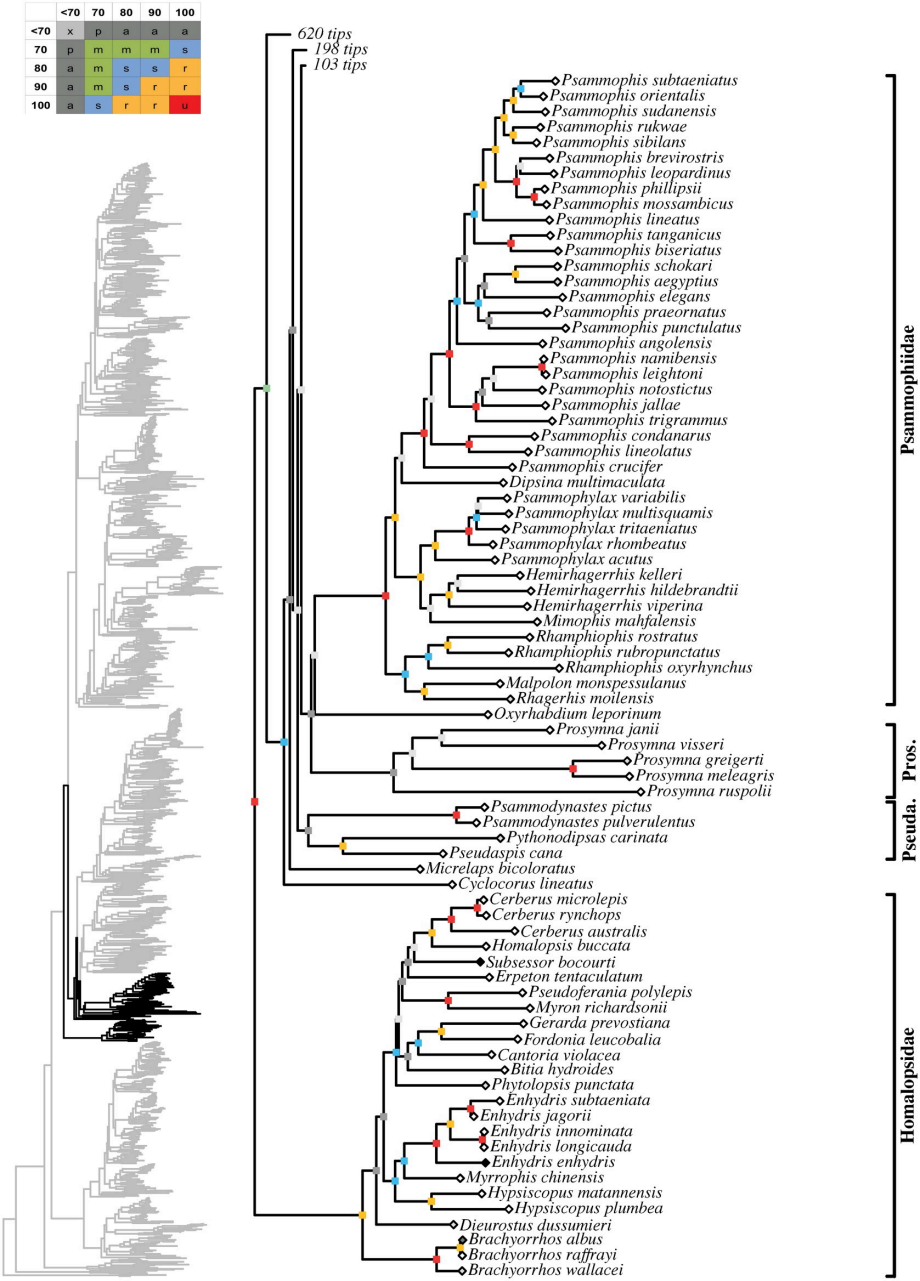
Maximum likelihood species-level phylogeny of Colubroides (continued). Basal Elapoidea, families Homalopsidae, Cyclocoridae, Pseudaspididae, Psammophiidae.

**Fig 11:**
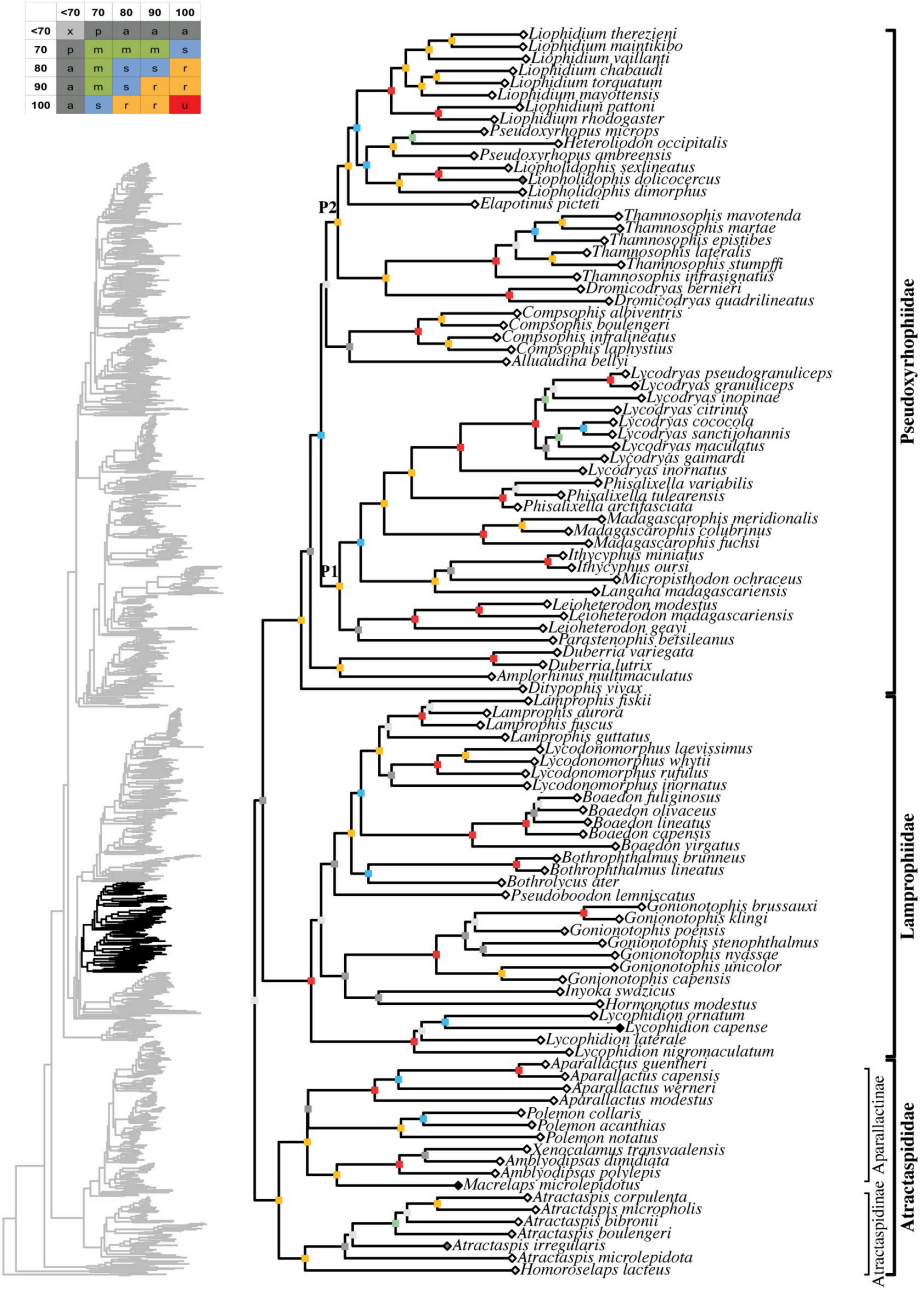
Maximum likelihood species-level phylogeny of Colubroides (continued). Families Atractaspididae, Lamprophiidae, Pseudoxyrhophiidae.

**Fig 12:**
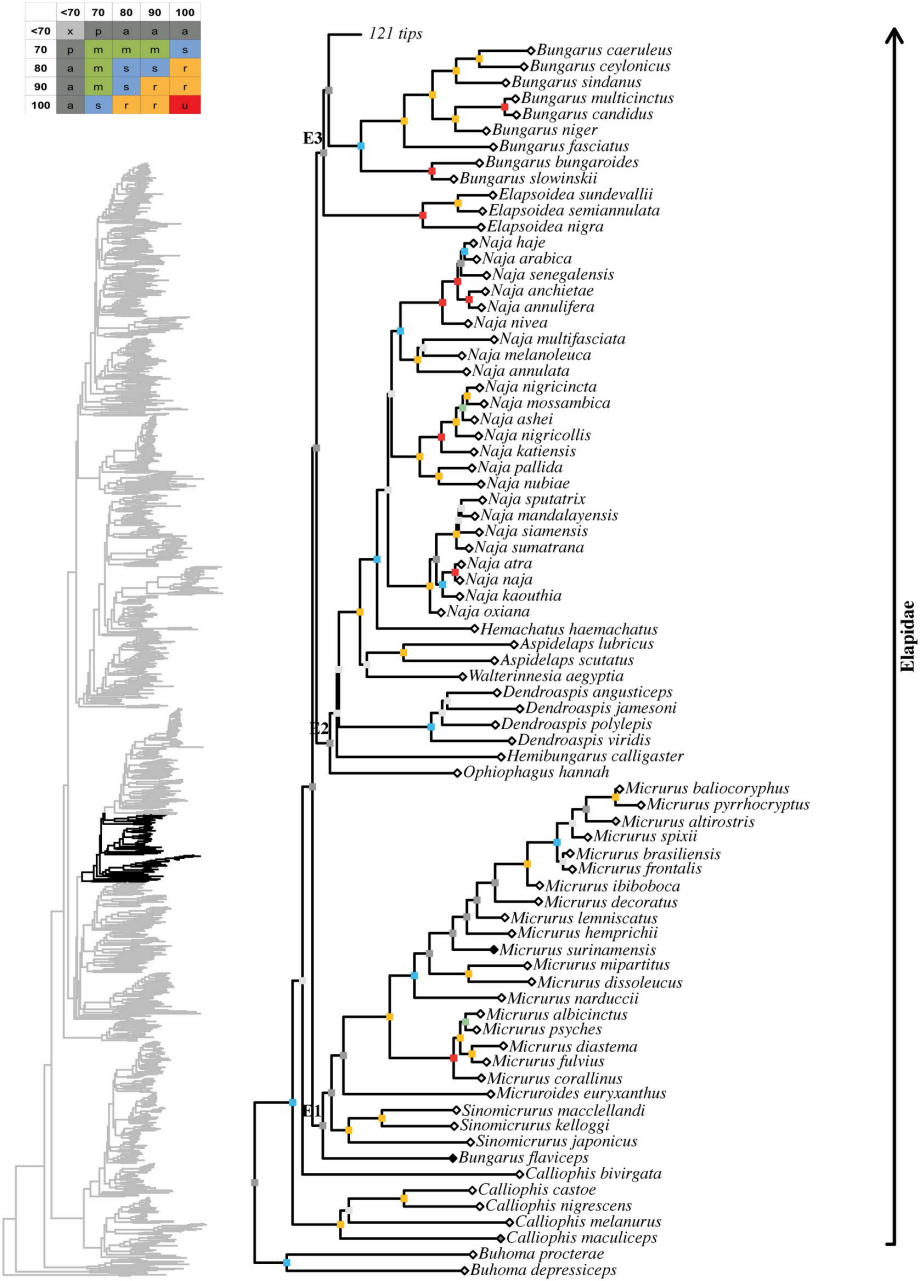
Maximum likelihood species-level phylogeny of Colubroides (continued). Family Elapidae.

**Fig 13:**
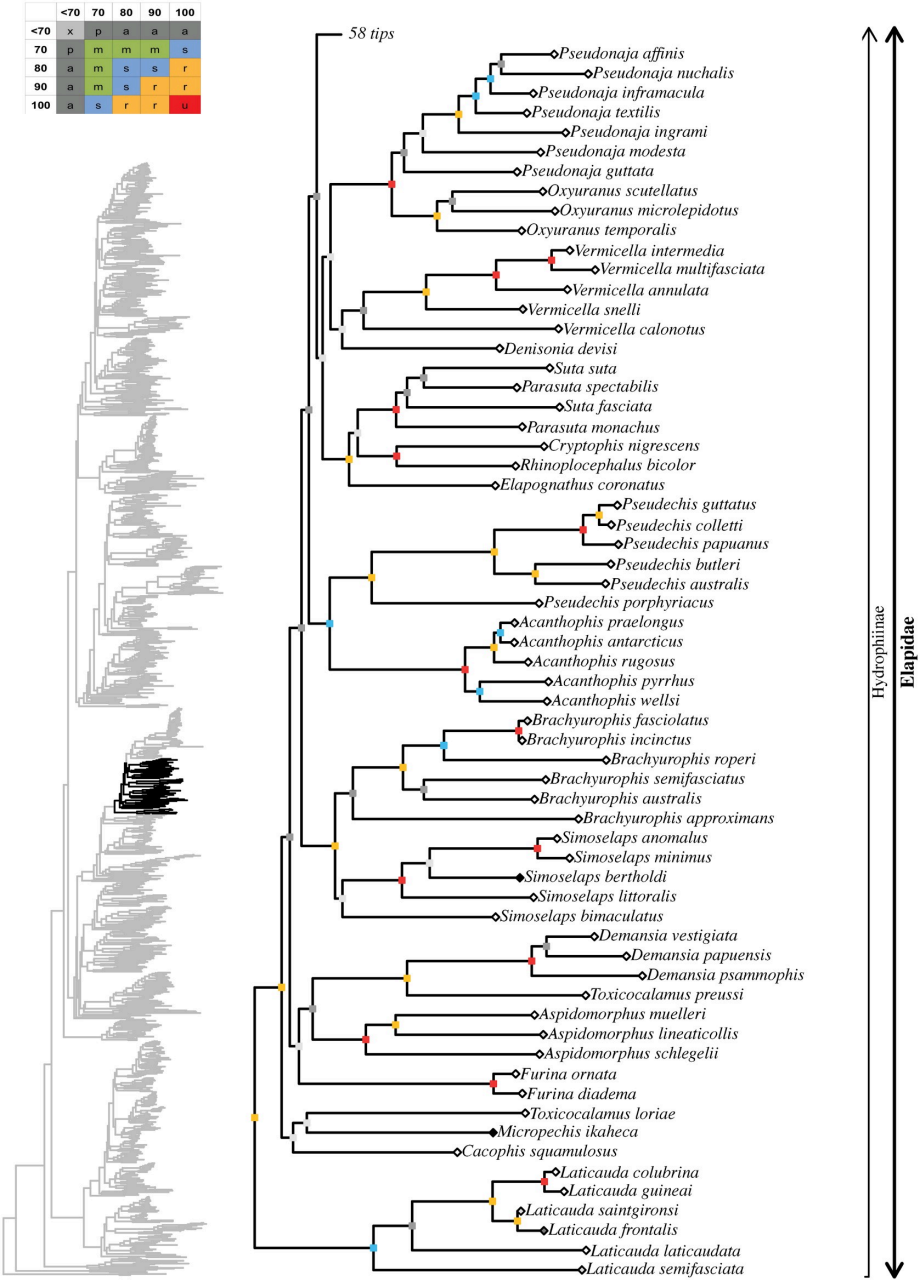
Maximum likelihood species-level phylogeny of Colubroides (continued). Family Elapidae (continued).

**Fig 14:**
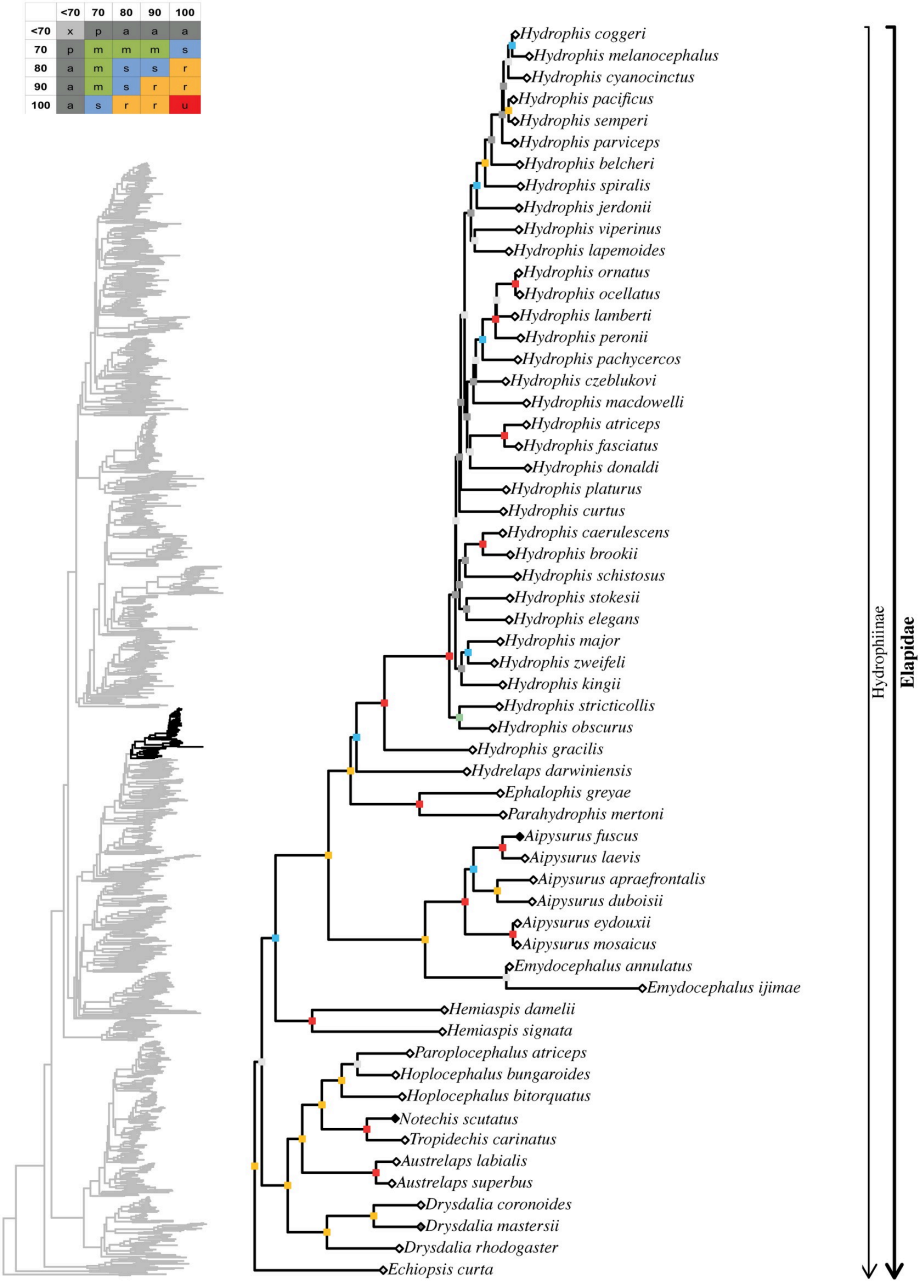
Maximum likelihood species-level phylogeny of Colubroides (continued). Family Elapidae (continued).

**Fig 15:**
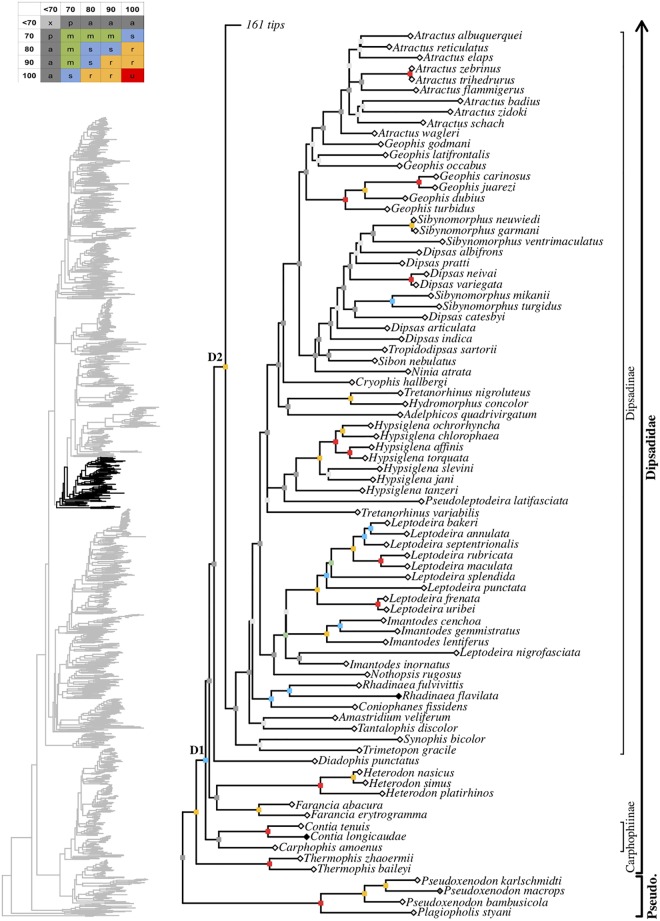
Maximum likelihood species-level phylogeny of Colubroides (continued). Families Pseudoxenodontidae and Dipsadidae.

**Fig 16:**
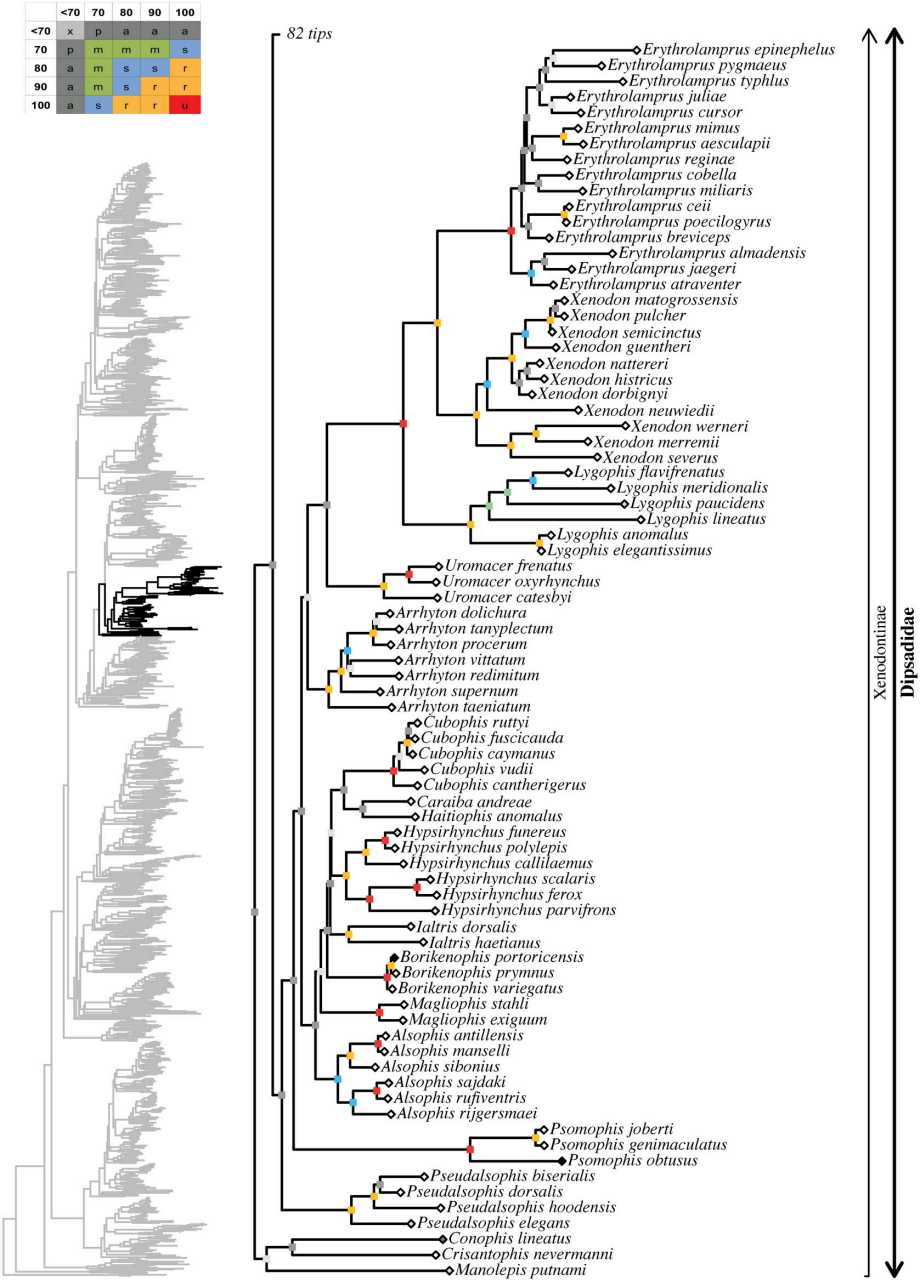
Maximum likelihood species-level phylogeny of Colubroides (continued). Family Dipsadidae (continued).

**Fig 17:**
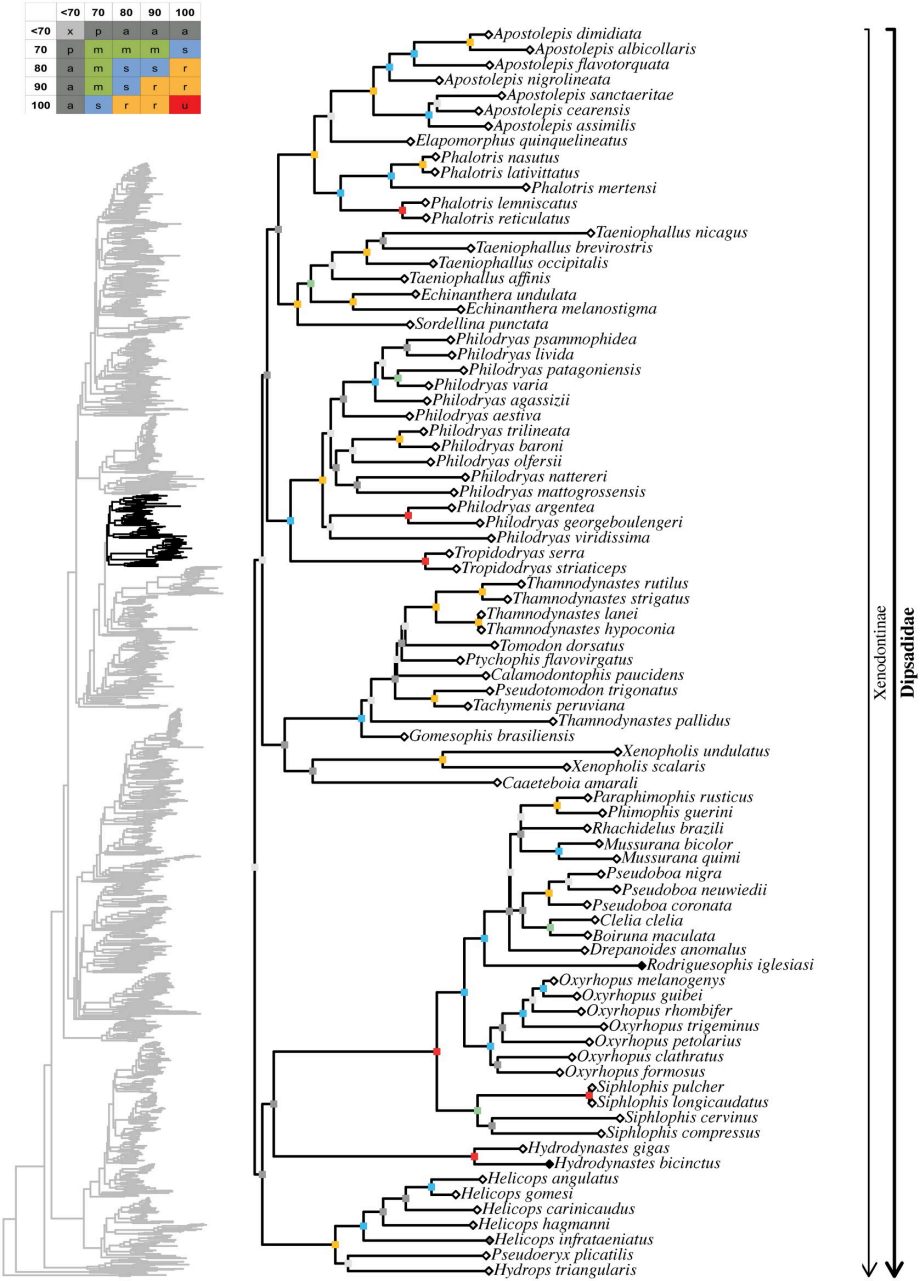
Maximum likelihood species-level phylogeny of Colubroides (continued). Family Dipsadidae (continued).

**Fig 18:**
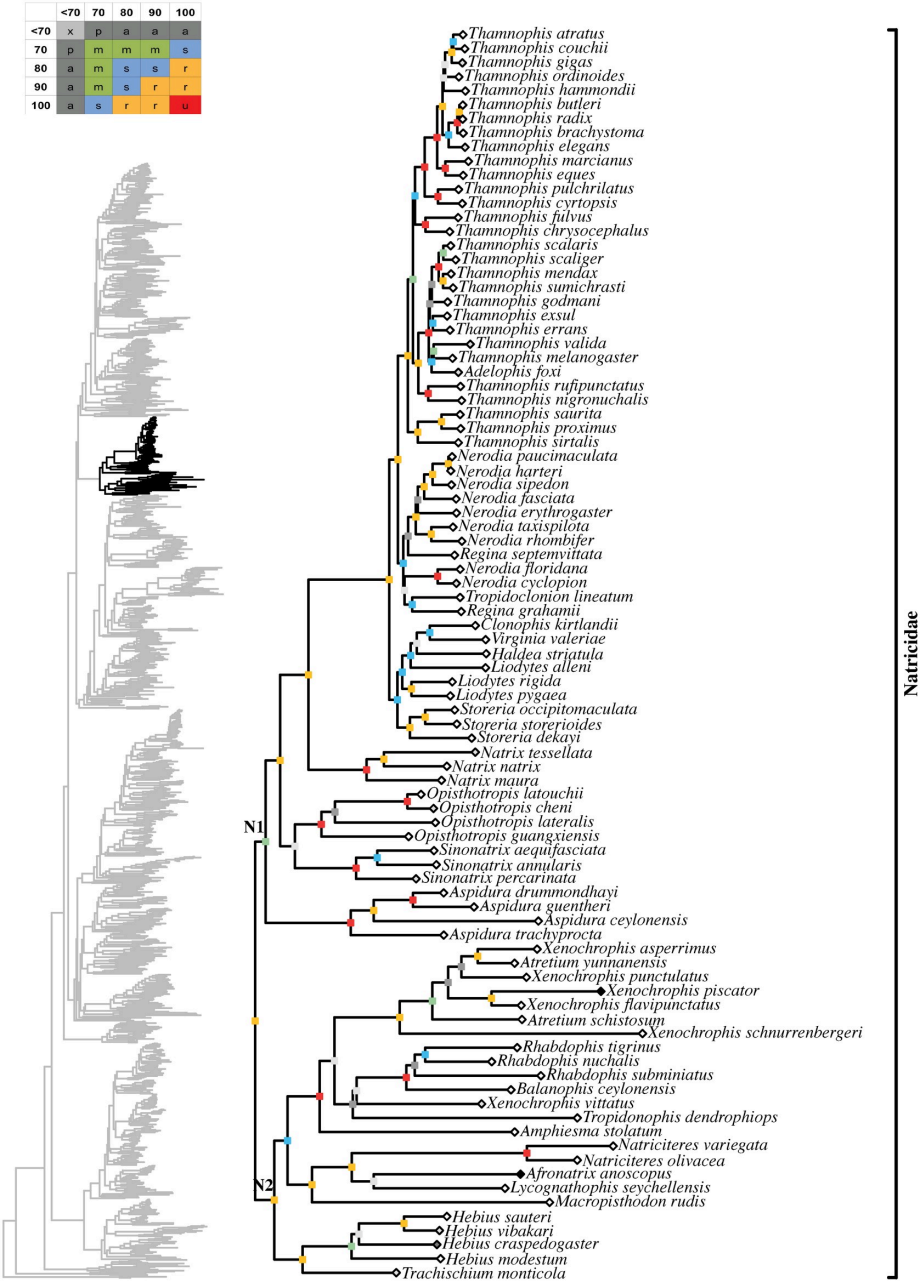
Maximum likelihood species-level phylogeny of Colubroides (continued). Family Natricidae.

**Fig 19:**
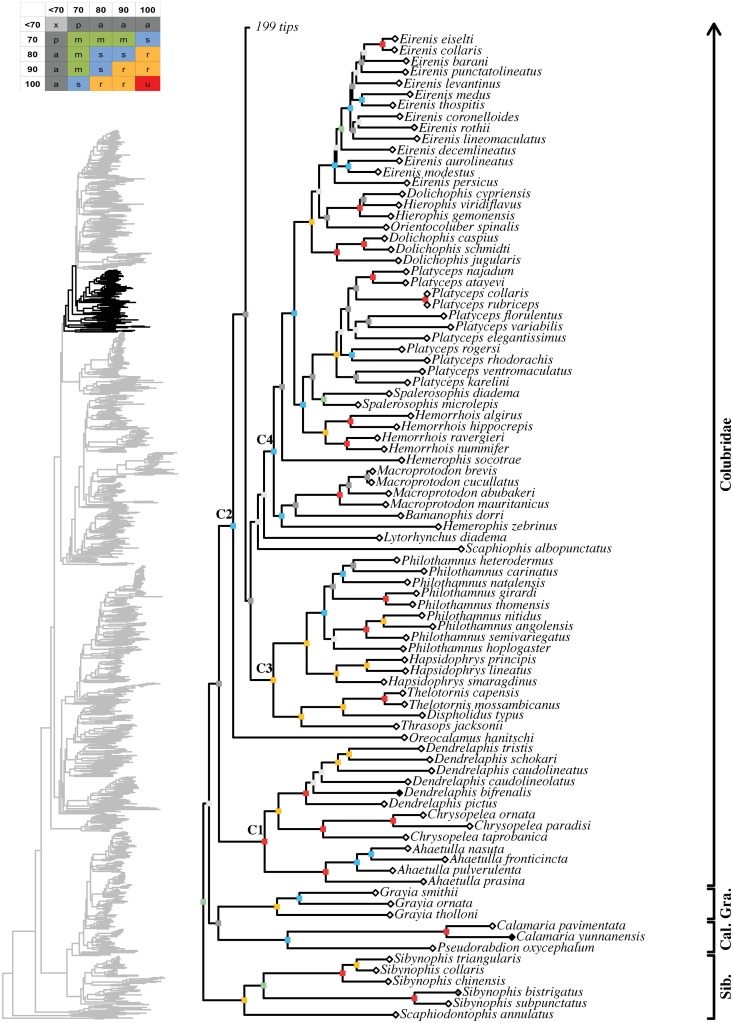
Maximum likelihood species-level phylogeny of Colubroides (continued). Families Sibynophiidae, Calamariidae, Grayiidae, and Colubridae.

**Fig 20:**
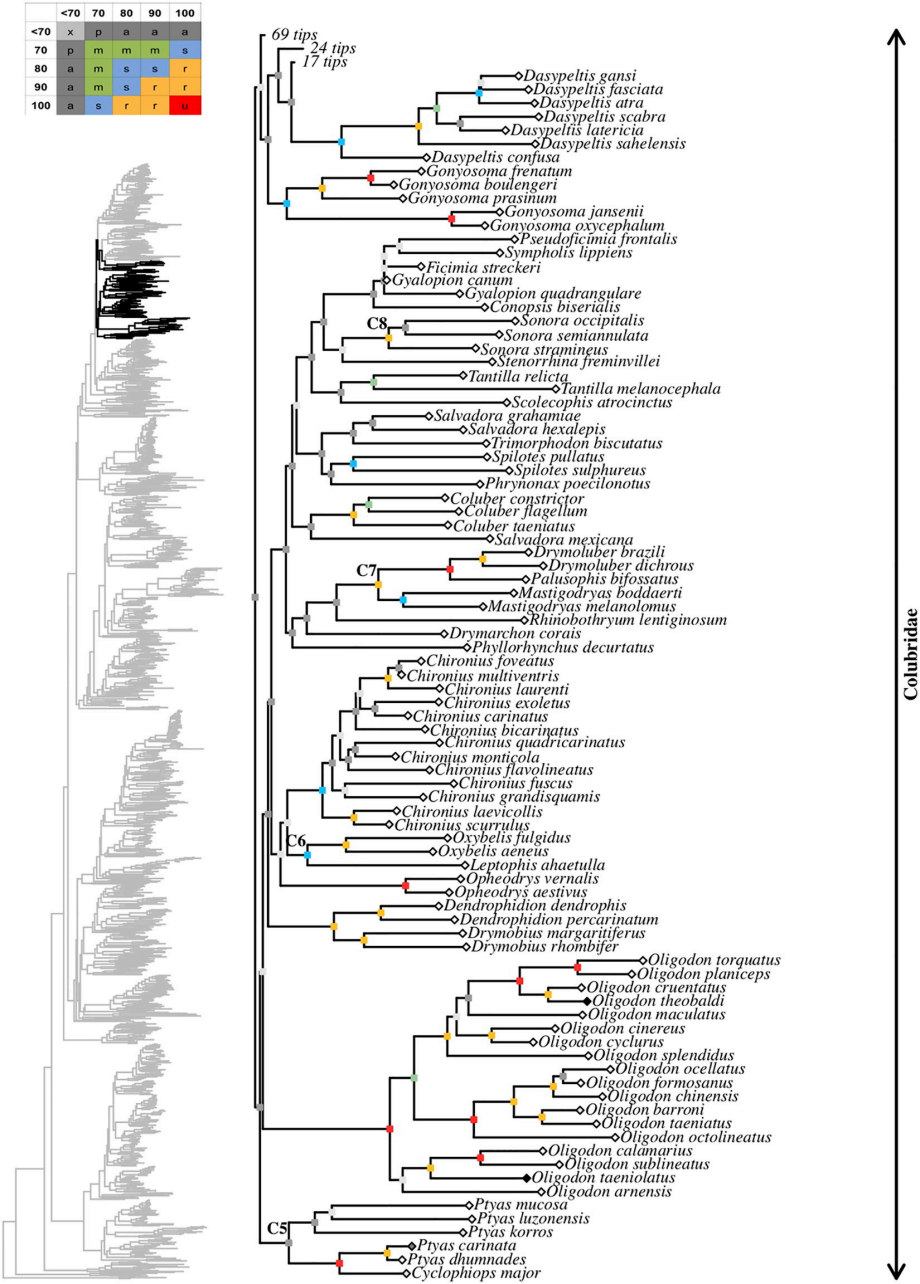
Maximum likelihood species-level phylogeny of Colubroides (continued). Family Colubridae (continued).

**Fig 21:**
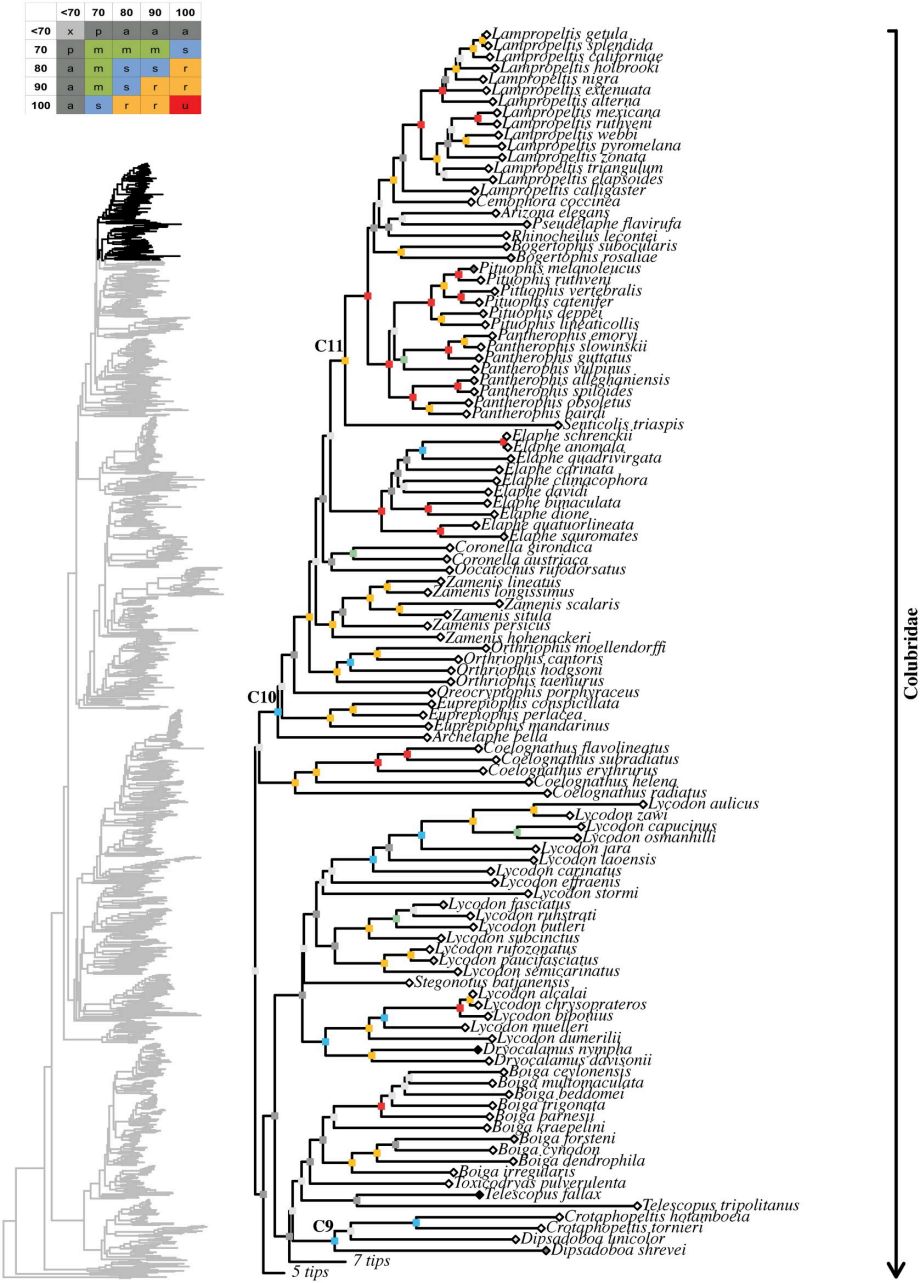
Maximum likelihood species-level phylogeny of Colubroides (continued). Family Colubridae (continued).

### Comparison of support metrics

Support scores are strongly but imperfectly correlated. Pairwise scatterplots (Fig 4) show that TBE and SHL scores tend to be higher (less conservative) than FBPs (see also S9 Table). Thus, combining FBP and SHL produces almost the same proportion of supported clades as does combining FBP and TBE, whereas combining TBE and SHL increases the number of seemingly well-supported clades. This suggests that SHL and TBE tend to inflate and/or FBP tends to underestimate the support for clades. Categories of combined support values can help express such discrepancies by classifying all ambiguous supports as weakly supported clades (gray points and bars in Fig 4). The tendency for SHL and TBE values to be higher than FBPs affects all clade ages (Fig 5; S3 Fig). FBP tends to have weaker support values especially for deeper nodes in our ML tree (S1 and S2 Figs).

Based on our statistical exploration of these three support methods (FBP, TBE, and SHL), we reinforce here the informative quality of providing combined support values for phylogenetic inference. By combining FBP with SHL or TBE we can easily spot nodes that are not well supported due to weak evidence in our datamatrix or by the presence of rogue terminals in our taxonomic sampling. Here, we preferred to comment the results and base our discussion using the joint values of FBP and SHL, since this approach produced more conservative values than FBP and TBE or SHL and TBE.

### Comparison of higher-level colubroidean affinities

Although our new and the three previous [26,27,28] studies are somewhat similar in scope, content and procedures, they all retrieved distinct sets of higher relationships throughout the caenophidian tree, reinforcing the view that estimates of caenophidian affinities leave significant room for improvement (Figs 1–3).

Deeper relationships in our tree ressemble more closely those found by Zheng and Wiens’ [27] (Fig 3B), with both studies finding unambiguous support values throughout that region of the caenophidian tree. Our tree differs most from that of Figueroa et al. [28] (Fig 1B). In all four caenophidian trees, all major families were retrieved as well-supported monophyletic lineages, but no consensus emerges in respect to deeper familial interrelationships or within the two larger endoglyptodont clades Elapoidea and Colubroidea.

Our analysis retrieved an unambiguously supported monophyletic Colubroides, as did Zheng and Wiens [27] and Figueroa et al. [28], but unlike Pyron et al. [26], who found Acrochordoidea nested within Colubroides as the sister-group of Xenodermidae (Fig 2B). In our tree, as in Zheng and Wiens [27] and Pyron et al. [26], Xenodermidae (100/100) and Pareidae (100/98) represented successive outgroups to the robustly supported clade Endoglyptodonta (99/99) formed by Viperidae (100/100) and the remaining colubroideans, while Figueroa et al. [28] retrieved Pareidae as the sister-group of Viperidae, although with very low SHL support. As in Zheng and Wiens [27], pareids and endoglyptodonts formed an unambiguously supported clade (100/100) Colubriformes. Within Endoglyptodonta, our results and those of Zheng and Wiens [27] further differed from Pyron et al. [26] and Figueroa et al. [28] by retrieving an unnamed, unambiguously supported clade in which Homalopsidae (99/100) is the sister group of the remaining endoglyptodont families (83/79) instead of the sister group of Lamprophiidae + Elapidae [26,28]. Both contradictory hypotheses of phylogenetic affinities for homalopsids presented robust to unambiguous support values in all four studies.

The remaining families of endoglyptodonts recognized here (i.e., Atractaspididae, Calamariidae, Colubridae, Dipsadidae, Elapidae, Grayiidae, Lamprophiidae, Natricidae, Prosymnidae, Psammophiidae, Pseudaspididae, Pseudoxenodontidae, Pseudoxyrhophiidae, and Sibynophiidae) formed a moderately supported clade (83/79) of higher colubroideans, consisting of two robustly supported sister clades recognized as superfamilies Elapoidea *sensu stricto* (89/99) and Colubroidea *sensu stricto* (89/100). The same two clades were retrieved by Pyron et al [26 and Figueroa et al [28] with robust support values, and by Zheng and Wiens [27] but with no significant support.

The superfamily Elapoidea contained the robustly to unambiguously supported families Elapidae (79/100), Lamprophiidae (100/100), Pseudoxyrhophiidae (97/100), Atractaspididae (96/100), and Psammophiidae (100/100) (Fig 1A; S1 Fig). Although elapids, atractaspidids, pseudoxyrhophiids, psammophiids, and lamprophiids formed strongly to unambiguously supported clades, their deeper interrelationships remained elusive, with support values being either non-significant or ambiguous in all four compared analyses (Figs 1–3). [26,27,28]. Additionally, the genera *Buhoma* (67%), *Oxyrhabdium* (50%), *Prosymna* (35%), *Psammodynastes* (100%), *Pythonodipsas* (100%), *Pseudaspis* (100%), *Micrelaps* (20%), and *Cyclocorus* (50%) were ambiguously positioned in our analysis. Our analysis did not obtain the family Cyclocoridae, recently suggested by Weinell and Brown [36] for four genera of endemic Philippine snakes, given that *Cyclocorus* and *Oxyrhabdium* did not cluster together as would be expected but were separated in an unsupported or poorly supported part of the tree at the base of the elapoid clade. The other three studies [26,27,28] did not sample *Cyclocorus*, and the remaining two genera of Cyclocoridae–*Hologerrhum* and *Myersophis*–were never sampled before. We interpret our results regarding the two sampled cyclocorids as illustrative of the spurious effects of poor taxon sampling. Contrary to Pyron et al. [26] and Zheng and Wiens [27], *Buhoma* was not retrieved within the Pseudaspididae but rather as the sister-group of Elapidae in an ambiguously supported clade (36/91). The genera *Psammodynastes*, *Pseudaspis*, and *Pythonodipsas* formed an ambiguously supported clade (22/91) of uncertain affinities in which *Pseudaspis* and *Pythonodipsas* clustered together as a robustly supported monophyletic group (93/99) representing the family Pseudaspididae [26]. Although Pseudaspididae including *Buhoma* was unambiguously supported by Pyron et al. [26], it was not statistically supported in Zheng and Wiens [27], suggesting that the status of Pseudaspididae as it stands deserves to be viewed with caution. Within Atractaspididae, subfamilies Aparallactinae (96/99) and Atractaspidinae (99/98) appeared as strongly supported clades only in our study and in Pyron et al. [26]. *Micrelaps* was retrieved as the sister group to an unsupported clade containing all the other elapoids (11/78) except *Cyclocorus*, a topology not reproduced by any of the three other studies. Although *Micrelaps* belonged firmly to Elapoidea (89/99), it did not cluster with the remaining Atractaspididae and its affinities within the elapoid radiation remained unsolved.

*Prosymna*, the only genus of the family Prosymnidae, was only ambiguously related to Psammophiidae (8/92) in our study. The same clade was also retrieved by Pyron et al. [26] and Zheng and Wiens [27], with high and no supports, respectively. In Figueroa et al. [28], *Prosymna* formed a distinct, unsupported clade with *Buhoma procterae*, breaking the monophyly of the genus *Buhoma*. Lamprophiidae and Pseudoxyrhophiidae formed an ambiguously supported clade (47/92) also retrieved by Pyron et al. [26] and Zheng and Wiens [27], although without support in the latter. Atractaspididae clustered with the latter clade with no statistical support in our analysis. Atractaspidids were not found in the same position in any of the other three studies [26,27,28], leaving its phylogenetic affinities within elapoids unknown.

Superfamily Colubroidea contained the robustly to unambiguously supported families Pseudoxenodontidae (100/100), Dipsadidae (95/97), Natricidae (92/100), Sibynophiidae (95/99), Grayiidae (99/100), the strongly supported family Calamariidae (78/97), and the ambiguously supported family Colubridae (56/90) (Fig 1A; S1 Fig). Similarly to elapoids, interrelationships between colubroid families remained unsolved, with sister-group relationships showing only ambiguous support values [e.g., Pseudoxenodontidae + Dipsadidae (51/94), Grayiidae + Calamariidae (21/85), Natricidae + Colubridae, Grayiidae, Calamariidae, and Sibynophiidae (27/75)]. Only the clade formed by Colubridae, Grayiidae, Calamariidae, and Sibynophiidae was retrieved with moderate support values (76/83).

Colubroid familial interrelationships in our study ressembled more closely Figueroa et al. [28], with only Natricidae being positioned differently in both studies. Our analysis clustered natricids as the sister group of a larger clade formed by the sibynophiids, calamariids, grayiids, and colubrids while Figueroa et al. [28] retrieved the natricids as the sister-group of Sibynophiidae. None of these hypotheses received minimum support. Our results differed more markedly from Pyron et al. [26] and Zheng and Wiens [27], in which the affinities of Dipsadidae, Calamariidae, Natricidae, and part of Colubridae (i.e., the Ahaetullinae) differed significantly. Additionally, as in Pyron et al. (26] and Zheng and Wiens [27], none of the colubroid familial interrelationships retrieved in our study received significant combined support. On the other hand, Figueroa et al. [28] found bootstrap support values superior to 80% for the following two clades: Pseudoxenodontidae-Dipsadidae (FBP 82%), Calamariidae-Grayiidae-Colubridae (FBP 99%).

### Affinities within major colubroidean families

#### Xenodermidae

Xenodermidae was represented by four genera and five species, corresponding to 67% and 28%, respectively, of its known diversity (Fig 6; S4 Table). Although poorly sampled, *Achalinus* (22%) was unambiguously supported and it formed the sister group of a strongly supported clade (83/83) containing *Stoliczkia*, *Xenodermus*, and *Fimbrios* in which *Stoliczkia* appeared as the sister group of a moderately supported clade (74/76) formed by *Xenodermus* (monotypic) and *Fimbrios* (50%). *Parafimbrios* was not sampled.

#### Pareidae

Our analysis included three genera and 12 species for Pareidae, representing 100% and 60% of their diversity, respectively (Fig 6; S4 Table). We incorporated new sequences for one species. *Asthenodipsas* and *Aplopeltura* were retrieved as successive sister taxa to a strongly supported clade that contained all 10 sampled species of *Pareas* (81/94; 56%) (including *Pareas tonkinensis* and *P*. *macularius* recognized here). *Aplopeltura* received only ambiguous support as the sister group of *Pareas* (51/78), a hypothesis that needs further testing. Within *Pareas*, *P*. *monticola* was retrieved as the sister group of a strongly supported clade (83/97) formed by the remaining species, which clustered into three robust clades, as follows: 1) *P*. *tonkinensis*, *P*. *hamptoni*, *P*. *nuchalis*, *P*. *carinatus* (92/100); 2) *P*. *macularius*, *P*. *margaritophorus* (85/100); and 3) *P*. *stanleyi*, *P*. *boulengeri*, *P*. *formosensis* (97/95). Within the first clade, *P*. *tonkinensis* and *P*. *hamptoni* were successive sister taxa to a robustly supported clade formed by *P*. *nuchalis* and *P*. *carinatus* (91/95), while within the third clade, *P*. *stanleyi* was the sister species to *P*. *boulengeri* and *P*. *formosensis* (99/100).

#### Viperidae

The unambiguously supported Viperidae was represented in our analysis by 34 genera and 247 species, which corresponded to 97% and 73% of its diversity, respectively (Figs 6–9; S4 Table). *Causus lichtensteinii* was not sequenced previously and we added sequences for 36 terminal taxa, which Alencar et al. [124] used previously (S3 Table). The basal divergence within the family is unambiguously and unquestionably resolved with subfamily Viperinae representing the sister-group to an unambiguously supported clade formed by Azemiopinae and the unambiguously supported Crotalinae. Higher-level affinities within Viperinae are not yet fully resolved, except for the robustly supported clade (100/99) formed by *Eristicophis*, *Pseudocerastes*, *Macrovipera*, *Montivipera*, *Daboia*, and *Vipera*. Within that clade, *Eristicophis* and *Pseudocerastes* formed an unambiguously supported group that was sister to a moderately supported clade (79/90) that included *Macrovipera* + *Montivipera* (100/100), on the one hand, and *Daboia* + *Vipera* (72/74), on the other hand. As for the rest of viperines, *Causus* appeared loosely positioned at the base of the clade (20/9), *Proatheris* was only ambiguously related (40/71) to a moderately supported clade (79/87) formed by *Atheris* and *Bitis*, while *Cerastes* and *Echis* were only ambiguously related to each other (65/98). All sampled polytypic genera were retrieved as monophyletic, with either robust or unambiguous support values as follows: *Causus* (100/100; 57%), *Atheris* (100/100; 50%), *Bitis* (99/100; 82%), *Cerastes* (100/100; 75%), *Echis* (100/100; 82%), *Pseudocerastes* (100/99; 67%), *Macrovipera* (100/100; 100%), *Montivipera* (99/95; 88%), *Daboia* (99/99; 80%), *Vipera* (100/100; 59%). Among viperids, only afrotropical *Montatheris* was not sampled.

Crotalinae ingroup affinities were mostly well resolved, except for some notable exceptions. The genera *Tropidolaemus*, *Deinagkistrodon*, *Garthius*, *Calloselasma*, and *Hypnale* formed an ambiguously supported clade (62/91) with unresolved interrelationships, except for *Calloselasma* uniting with *Hypnale* (100/100). The remaining crotalines formed a robust clade (97/100) that retrieved *Trimeresurus* (62%), *Ovophis* (67%), *Atropoides* (100%), and *Bothrops* (69%) as polyphyletic genera, despite recent extensive taxonomic changes within these groups (see [33]). All species of *Trimeresurus*, except *T*. *gracilis*, formed a robust clade (99/100) that was the sister group to a strongly supported clade (88/96) that included the remaining Paleartic, Neartic, and Neotropical species. Within that latter group, *Ovophis okinavensis* and *Trimeresurus gracilis* formed an unambiguously supported clade while the remaining species of *Ovophis* formed another clade that was only ambiguously related (27/76) to *Gloydius*. Within Neartic and Neotropical crotalines, the following suprageneric groups were retrieved: 1) a robustly supported, mainly Central American clade formed by the genera *Atropoides*, *Cerrophidion*, and *Porthidium* (94/100); 2) an ambiguously supported, mainly Meso and North American clade formed by *Lachesis*, *Ophryacus*, *Mixcoatlus*, *Bothriechis*, *Agkistrodon*, *Sistrurus*, and *Crotalus* (43/91); 3) an unambiguously supported South American clade formed by *Bothrocophias* and *Bothrops*. Within that latter group, the genus *Bothrops* appeared to be polyphyletic, with *Bothrops lojanus* nesting within *Bothrocophias* and the remaining species of *Bothrops* grouping together in a strongly supported clade (77/92).

Among crotalines, monophyly seemed well established for the following polytypic genera: *Hypnale* (100/100; 100%), *Protobothrops* (100/100; 79%), *Gloydius* (100/100; 71%), *Cerrophidion* (70/98; 80%), *Porthidium* (100/100; 89%), *Lachesis* (100/100; 75%), *Mixcoatlus* (100/100; 67%), *Bothriechis* (96/98; 91%), *Agkistrodon* (100/100; 67%), *Sistrurus* (100/99; 100%), *Crotalus* (97/98; 76%).

#### Homalopsidae

Our analysis included 16 genera and 25 species of homalopsids, which corresponded to 57% and 47% of its known diversity, respectively (Fig 10; S4 Table). Our data incorporated new sequences for five species of homalopsids (S3 Table). *Brachyorrhos* and *Dieurostus* formed successive sister taxa to the remaining homalopsids. The latter formed an ambiguously supported clade (51/82) to which *Dieurostus* was only poorly supported as its sister taxon (68/74), revealing that basal interrelationships in homalopsids remain unsolved. The remaining homalopsids clustered in two distinct clades: one exclusively composed by Indo-Malayan species (74/97), and the other formed by Indo-Malayan and Australasian species (84/98). The former, exclusively Indo-Malayan clade, was composed by *Hypsiscopus*, *Myrrophis*, and *Enhydris*, and the latter by *Phytolopsis*, *Bitia*, *Cantoria*, *Fordonia*, *Gerarda*, *Myron*, *Pseudoferania*, *Erpeton*, *Subsessor*, *Homalopsis*, and *Cerberus*. Interrelationships were well resolved within the first clade, with *Hypsiscopus* being the sister group to a strongly supported clade formed by *Myrrophis* and *Enhydris* (87/94). In contrast, the second clade had only unsupported or ambiguously supported affinities between most genera. Only three clades received strong to unambiguous support: 1) *Cantoria*, *Fordonia*, and *Gerarda* (79/93); 2) *Myron* and *Pseudoferania* (100/100); and 3) *Homalopsis* and *Cerberus* (100/100). Within the first, *Fordonia* + *Gerarda* received robust support (93/96). On the other hand, the genera *Bitia*, *Erpeton*, and *Subsessor* were only loosely positioned, showing unclear affinities within their larger clade. Among homalopsids, monophyly of the following polytypic genera was well established: *Brachyorrhos* (100/100; 75%), *Hypsiscopus* (96/100; 100%), *Enhydris* (100/100; 83%), *Cerberus* (100/100; 60%). The following 12 genera of homalopsids were not sampled: *Calamophis*, *Djokoiskandarus*, *Ferania*, *Gyiophis*, *Heurnia*, *Homalophis*, *Karnsophis*, *Kualatahan*, *Mintonophis*, *Miralia*, *Raclitia*, and *Sumatranus*.

#### Psammophiidae

Psammophiidae was represented by eight genera and 41 species, 100% and 79%, respectively, of its known diversity (Fig 10; S4 Table). We added new sequences for six species (S3 Table). This unambiguously supported clade showed a basal split, with two well supported monophyletic groups: a strongly supported clade (88/99) containing the genera *Rhagerhis* (monotypic), *Malpolon* (50%), and *Rhamphiophis* (100%), and a robustly supported clade (92/93) that included *Mimophis* (monotypic), *Hemirhagerrhis* (75%), *Psammophylax* (100%), *Dipsina* (monotypic), and *Psammophis* (76%). *Rhagerhis* and *Malpolon* formed a robust clade (100/98) within the former group. In the latter group, *Mimophis*, *Hemirhagerrhis*, and *Psammophylax* also formed a robust clade (90/100), while the sister group relationship retrieved for *Dipsina* and *Psammophis* had no statistical support. All four polytypic genera were monophyletic, with strong to unambiguous support values, as follows: *Rhamphiophis* (89/98), *Hemirhagerrhis* (98/97), *Psammophylax* (100/100), *Psammophis* (100/100).

#### Atractaspididae

Our analysis included eight recognized genera and 19 species of atractaspidid snakes, corresponding to 73% and 28% of its known diversity, respectively (Fig 11; S4 Table). However, retaining *Micrelaps* in the family renders it polyphyletic. Thus, we considered the latter to be *incertae sedis* within Elapoidea. Subfamilies Aparallactinae (99/98) and Atractaspidinae (96/99; excluding *Micrelaps*) formed two robust sister-groups. Aparallactinae was represented by five genera only and 12 species, accounting for 67% and 26% of its diversity. Within the subfamily, *Polemon* (25%) and *Aparallactus* (36%) are grouped together in an ambiguously supported clade (78/0). A robust clade (97/99) formed by *Macrelaps* (monotypic), *Amblyodipsas* (22%), and *Xenocalamus* (20%) was the sister group to *Polemon* + *Aparallactus*. The only sequenced species of *Xenocalamus* nested within a paraphyletic genus *Amblyodipsas*, suggesting that the former should be synonymized with the latter, which has priority. However, the low number of sampled species from both genera (no more than 22%) provided only a sketchy view of their phylogenetic affinities. Monophyly of these two closely related genera cannot be discarded at this moment. On the other hand, *Polemon* (97/100) and *Aparallactus* (100/100) were retrieved as robust and unambiguous clades, respectively. Atractaspidinae included *Homoroselaps* and *Atractaspis* [33] with two and 21 species, respectively. Our analysis involved six species of *Atractaspis* (29%) and only one of *Homoroselaps* (50%), for which new sequences were produced (S3 Table). While the subfamily was clearly monophyletic, surprisingly, the genus *Atractaspis* was retrieved with ambiguous support (60/95). Regardless, the presence of several apomorphic morphological characters defining this genus leaves little doubt about its monophyly [125,126]. We did not sample *Brachyophis*, *Chilorhinophis*, and *Hypoptophis*.

#### Lamprophiidae

The lamprophiid radiation accounts for 12 genera and 72 species, from which we evaluated 10 genera (83%) and 29 species (40%) (Fig 11). New sequences were generated for five species (S3 Table). Higher-level interrelationships remained unresolved with all the deep groupings having only poor or ambiguous support. The position of *Lycophidion* as the sister group of a clade formed by the remaining nine sampled genera received poor support, and the latter clustered in two ambiguously supported clades: 1) *Hormonotus*, *Inyoka*, and *Gonionotophis* (63/97); 2) *Pseudoboodon*, *Bothrolycus*, *Bothrophthalmus*, *Boaedon*, *Lycodonomorphus*, and *Lamprophis* (55/82). Monotypic *Hormonotus* and *Inyoka* were only ambiguously supported as sister taxa (66/81), while *Bothrolycus*, *Bothrophthalmus*, *Boaedon*, *Lycodonomorphus*, and *Lamprophis* formed a robust clade (93/97) for which *Pseudoboodon* clustered ambiguously as its sister group. *Bothrolycus* and *Bothrophthalmus* formed a strongly supported clade (83/94) that was the sister group to another strongly supported clade formed by *Boaedon*, *Lycodonomorphus*, and *Lamprophis* (78/92). The South African *Lamprophis* and *Lycodonomorphus* were further retrieved as sister taxa with robust support values (92/97). Polytypic *Lycophidion* (20%), *Gonionotophis* (40%), *Bothrophthalmus* (100%), *Boaedon* (83%), *Lycodonomorphus* (50%), and *Lamprophis* (57%) were unambiguously monophyletic. *Pseudoboodon* was represented by only one of the four species (25%). *Chamaelycus* and *Dendrolycus* were the only known lamprophiid genera not sampled.

#### Pseudoxyrhophiidae

Our sampling of Pseudoxyrhophiidae included 20 of 22 genera (91%) and 55 of the 89 species (62%) recognized by Uetz et al. [33] (Fig 11). New sequences were generated for 10 species (S3 Table). Socotran *Ditypophis vivax* and a robust clade formed by the African mainland *Amplorhinus* and *Duberria* (93/98) were retrieved as successive sister groups to the remaining pseudoxyrhophiids. However, this arrangement received ambiguous support (64/77). The remaining Malagasy pseudoxyrhophiids formed a strongly supported clade (76/95) composed of one ambiguously and two robustly supported clades. The robustly supported clades comprised two different assemblages: 1) a group that included the genera *Parastenophis*, *Leioheterodon*, *Langaha*, *Micropisthodon*, *Ithycyphus*, *Madagascarophis*, *Phisalixella*, and *Lycodryas* (Clade P1; 96/99), and 2) another group composed by *Dromicodryas*, *Thamnosophis*, *Elapotinus*, *Liopholidophis*, *Pseudoxyrhopus*, *Heteroliodon*, and *Liophidium* (Clade P2; 90/98). The ambiguously supported clade was composed by the genera *Compsophis* and *Alluaudina* (69/97), which clustered with no statistical support with the latter larger clade of Malagasy pseudoxyrhophiids (Clade P2). Interrelationships within Clades P1 and P2 were well established in general, with support values above 80 in both FBP and SHL, except for a few clades. Within Clade P1, *Leioheterodon* and *Parastenophis* clustered with ambiguous support (66/86). This group appeared as the sister taxon of a strongly supported clade (88/99) that included the remaining six genera of Clade P1. The latter included two strongly supported clades, one with *Langaha*, *Leioheterodon*, and *Ithycyphus* (99/100), and the other with *Madagascarophis*, *Phisalixella*, and *Lycodryas* (98/99). *Phisalixella* and *Lycodryas* formed a strongly supported clade (99/98), that was the sister group of *Madagascarophis*. Within Clade P2, *Dromicodryas* and *Thamnosophis* were robustly united (99/100) as were *Elapotinus*, *Liopholidophis*, *Pseudoxyrhopus*, *Heteroliodon*, and *Liophidium* (91/92). *Elapotinus* was strongly placed as the sister taxon to a clade formed by *Liopholidophis*, *Pseudoxyrhopus*, *Heteroliodon*, and *Liophidium*, which also received strong support (81/91). *Liophidium* was retrieved as the sister group to a strongly supported clade (80/88) formed by the monophyletic genus *Liopholidophis* and a paraphyletic *Pseudoxyrhopus* that included *Heteroliodon occipitalis*, which clustered with moderate support with *Pseudoxyrhopus microps* (82/79).

Except for *Pseudoxyrhopus* that appeared paraphyletic in respect to *Heteroliodon*, all polytypic genera were retrieved as monophyletic with robust to unambiguous support values. Available sampling for each one of these genera was as follows: *Duberria* (50%), *Leioheterodon* (100%), *Ithycyphus* (40%), *Madagascarophis* (75%), *Phisalixella* (75%), *Lycodryas* (80%), *Compsophis* (57%), *Dromicodryas* (100%), *Tamnosophis* (100%), *Liopholidophis* (38%), *Liophidium* (80%). Only *Brygophis* and *Pararhadinaea* among pseudoxyrhophiid genera were missing in our analysis.

#### Elapidae

Our analysis included 47 of the 55 genera (85%) and 196 of the 361 (54%) of the recognized species of Elapidae (Figs 12–14). We added two previously unsequenced species (*Dendroaspis viridis*, *Naja oxiana*) and incorporated new or available sequences for 18 species. Higher-level interrelationships within that large and medically important assemblage of venomous snakes was far from resolved (Figs 12–14). The only robustly supported higher-level clade of elapids known so far was the Australo-Melanesian radiation (84/100, Figs 13 and 14), which includes both Australo-Melanesian terrestrial and marine forms and will be treated here as the “Hydrophiine radiation” (also recognized as the subfamily “Oxyuraninae” [104]). All other higher-level affinities retrieved here were either ambiguously supported or not supported at all. Our analysis provided evidence of polyphyly of *Calliophis*, *Bungarus*, *Toxicocalamus*, and paraphyly of *Suta*, *Parasuta*, *Hoplocephalus*.

The genus *Calliophis* (50%) was retrieved as the sister-group of all remaining elapids, but its monophyly was not recovered. *Calliophis bivirgata* did not cluster with the strongly supported clade formed by the remaining species of *Calliophis* (98/98, Fig 12), but rather was more closely related to all the remaining elapids than to its congeners. However, non-monophyly of the genus is not confirmed yet, because the phylogenetic position of *C*. *bivirgata* is not supported (56/56, Fig 12).

Excluding *Calliophis*, all other genera of Elapidae grouped into three ambiguously supported clades (Fig 12): Clade E1 (21/92) contained *Bungarus flaviceps* and the genera *Sinomicrurus* (60%), *Micruroides* (monotypic), and *Micrurus* (24%); Clade E2 (64/99) held *Ophiophagus*, *Hemibungarus*, *Dendroaspis*, *Walterinnesia*, *Aspidelaps*, *Hemachatus*, and *Naja*; and Clade E3 (30/96) included *Elapsoidea*, *Bungarus* (except *B*. *flaviceps*), *Laticauda*, and the hydrophiine radiation. Afro-Asian/Australo-Melanesian Clades E2 and E3 clustered together albeit ambiguously (18/78).

Within Clade E1, the genera *Sinomicrurus* and *Micruroides* were retrieved as successive sister taxa to *Micrurus*, although with ambiguous supports (64/92 and 67/92, respectively). Polytypic *Sinomicrurus* and *Micrurus* were retrieved as strongly monophyletic (96/96 and 99/100, respectively). All the deep groupings within Clades E2 and E3 were poorly or ambiguously supported, although several monophyletic suprageneric clades were well supported within them.

Within Clade E2, the following genera grouped in two strongly to robustly supported clades (Fig 12): 1) *Walterinnesia*, *Aspidelaps*, *Hemachatus*, and *Naja* (94/100); 2) *Hemachatus* and *Naja* (86/97). The affinities of *Ophiophagus*, *Hemibungarus*, and *Dendroaspis* within clade E2 were not resolved. Although *Walterinnesia* and *Aspidelaps* formed sister-taxa within a robustly supported clade that included *Hemachatus* and *Naja*, their sister-group affinities remained uncertain (43/22).

Clade E3 had the following six well supported clades (Figs 12–14): 1) *Simoselaps* and *Brachyurophis* (91/100); 2) *Acanthophis* and *Pseudechis* (73/98); 3) *Elapognathus*, *Rhinoplocephalus*, *Cryptophis*, and paraphyletic *Suta* and *Parasuta* (96/100); 4) *Rhinoplocephalus* and *Cryptophis* (100/100); 5) *Oxyuranus* and *Pseudonaja* (100/100); and 6) *Echiopsis*, *Drysdalia*, *Austrelaps*, *Tropidechis*, *Notechis*, *Hoplocephalus*, *Paroplocephalus*, *Hemiaspis*, *Emydocephalus*, *Aipysurus*, *Parahydrophis*, *Ephalophis*, *Hydrophis* (91/100). The latter group corresponded to a viviparous radiation of hydrophiines in which *Echiopsis* rooted at the base of the tree but with unclear affinities to two clearly monophyletic lineages. One robust lineage (98/99) included *Drysdalia*, *Austrelaps*, and *Tropidechis* + *Notechis* as successive sister taxa to a paraphyletic *Hoplocephalus* that included *Paroplocephalus*. All sister-group hypotheses within this lineage were supported robustly (FBP/SHL > 90), and the *Tropidechis* + *Notechis* sister group relationship was unambiguous. The second lineage of this viviparous radiation was strongly supported (72/95) and retrieved *Hemiaspis*, *Emydocephalus* + *Aipysurus*, *Parahydrophis* + *Ephalophis*, and *Hydrelaps* as successive sister taxa to the speciose *Hydrophis*. All hypotheses of sister-group relationships received strong or robust support values. *Emydocephalus* + *Aipysurus* and *Parahydrophis* + *Ephalophis* were unambiguously and robustly supported (90/100), respectively.

Among polytypic elapid genera, monophyly was well established for *Dendroaspis* (78/100; 100%), *Aspidelaps* (98/99; 100%), *Elapsoidea* (100/100; 30%), *Laticauda* (81/98; 75%), *Furina* (100/100; 40%), *Aspidomorphus* (100/100; 100%), *Demansia* (100/100; 21%), *Acanthophis* (100/100; 56%), *Pseudechis* (97/61; 67%), *Oxyuranus* (100/99; 100%), *Drysdalia* (99/100; 100%), *Austrelaps* (100/100; 67%), *Hemiaspis* (100/100; 100%), *Aipysurus* (100/100; 67%), and *Hydrophis* (100/100; 72%). The polytypic genera that did form a clade, but had ambiguous or no support values at all were as follow: *Naja* (51/46; 83%), *Simoselaps* (29/35; 100%), *Brachyurophis* (62/77; 86%), *Vermicella* (49/76; 83%), *Pseudonaja* (61/80; 78%), and *Emydocephalus* (61/28; 67%). Polytypic elapid genera that were retrieved as paraphyletic are as follows: *Suta* (50%) and *Parasuta* (33%) in respect to each other, *Hoplocephalus* (67%) in respect to *Paroplocephalus* (monotypic).

*Toxicocalamus* (17%), *Calliophis* (50%), and *Bungarus* (71%) were retrieved as polyphyletic. Polytypic genera for which there was only one representative species sampled were as follows: *Walterinnesia* (50%), *Cacophis* (25%), *Elapognathus* (50%), *Cryptophis* (20%), *Denisonia* (50%). *Antaioserpens*, *Kolpophis*, *Loveridgelaps*, *Ogmodon*, *Parapistocalamus*, *Pseudohaje*, *Salomonelaps*, and *Thalassophis* were not sampled.

#### Pseudoxenodontidae

Pseudoxenodontidae was represented by four species belonging to the two genera allocated in the family (100%)–*Plagiopholis* and *Pseudoxenodon*–representing 40% of its known diversity (Fig 15; S4 Table). We incorporated new or available sequences for three species sampled in this analysis. This unambiguously supported family retrieved a robustly supported monophyletic genus *Pseudoxenodon* (100/95) as the sister group of the genus *Plagiopholis*.

#### Dipsadidae

Dipsadidae was represented in our matrix by 78 of 96 genera (78%) and 239 of 764 species (31%) recognized by Uetz et al. [33] (Figs 15–17). Within subfamilies [23], our analysis evaluated 22 of 27 genera (81%) and 67 of 396 species (17%) of Dipsadinae, 50 of 57 genera (88%) and 161 of 342 species (47%) of Xenodontinae, and all three genera (100%) and four of the five species (80%) of Carphophiinae. We also included three of nine genera (33%) and seven of 21 species (20%) recognized by Zaher et al. [23] and Grazziotin et al. [21] as Dipsadidae *incertae sedis*. The family was recovered as a robust clade (95/97) with a basal dichotomy between the Asiatic genus *Thermophis* and a strongly supported clade that included all New World species of Dipsadidae (Clade D1; 84/99). Asiatic *Stichophanes ningshaanensis*, which probably is the closest sister group of all New World dipsadids [127], was not included because no sequences were available in GenBank when the data matrix was finalized [128]. Within New World dipsadids, only Xenodontinae and Dipsadinae were monophyletic (Figs 15–17) but with ambiguous support values (58/96 and 65/98, respectively). They grouped together in a robustly supported clade (Clade D2; 92/99). This clade included those species referred to traditionally as South (Xenodontinae) and Central (Dipsadinae) American radiations of dipsadids [129,130]. In contrast, the North American radiation and subfamily Carphophiinae were recovered as paraphyletic and polyphyletic, respectively (Fig 15). Polyphyly of Carphophiinae was caused by the position of *Diadophis*, which clustered poorly with Clade D2 in an ambiguously supported clade (28/85), instead of grouping with *Carphophis* and *Contia*. Furthermore, carphophiines did not group with the clade formed by *Farancia* and *Heterodon* (Fig 15), causing the North American radiation to be paraphyletic. Within this radiation polytypic *Heterodon* (60%) and *Contia* (100%) were unambiguously monophyletic, and *Farancia* (100/99; 100%) was robustly monophyletic.

Most hypotheses of relationships within Dipsadinae had moderate or weak support (Fig 15). Imantodini received moderate support (70/81) and was only ambiguously supported as the sister group of Nothopsini [127]. The tribe Dipsadini also received ambiguous support, as did its sister group relationship with the clade composed by *Geophis* and *Atractus*. Monophyly of the recently redefined Diaphorolepidini [131] could not be assessed in our analysis since we sampled only one species for the tribe. We also did not test the monophyly of nine polytypic genera of Dipsadinae, because we included only one species for each of them. These are as follow: *Adelphicos* (17%), *Amastridium* (50%), *Coniophanes* (6%), *Hydromorphus* (50%), *Ninia* (10%), *Sibon* (6%), *Synophis* (13%) *Trimetopon* (17%) and *Tropidodipsas* (14%). *Chersodromus*, *Diaphorolepis*, *Plesiodipsas*, *Rhadinella* and *Urotheca* were not included in our analysis.

Of the nine polytypic genera sampled, six were recovered as non-monophyletic as follows: *Geophis* (14%) was paraphyletic in relation to *Atractus* (7%); *Dipsas* (18%) and *Sibynomorphus* (36%) were paraphyletic in relation to each other; *Tretanorhinus* (50%), *Leptodeira* (83%), and *Imantodes* (50%) were recovered as polyphyletic, with *Imantodes inornatus* and *Leptodeira nigrofasciata* grouping together ambiguously (34/82). Additionally, *Atractus* and *Hypsiglena* (78%) were ambiguously supported. Only *Rhadinaea* (10%) retrieved strong support.

All but one of the tribes of Xenodontinae [21,23] were monophyletic. Tribes Tropidrodryadini, Pseudoboini, Hydrodynastini, Xenodontini, and Psomophiini were retrieved with unambiguous support, while Elapomorphini (89/100), Echinantherini (91/94), Philodryadini (95/98), Hydropsini (96/100), and Saphenophiini (99/100) were recovered as robust clades. Monophyly of the tribe Tachymenini was only moderately supported (79/100) in our analysis. Only Alsophiini was not monophyletic because *Arrhyton* grouped with *Uromacer* and the tribe Xenodontini, yet with no support (Fig 16). The clade formed by Uromacer and Xenodontini received ambiguous support (51/83). Relationships among the tribes were generally poorly supported, except for the clade formed by Philodryadini and Tropidodryadini that was recovered with strong support (73/97). The monospecific tribe Caaeteboini grouped together with the genus *Xenopholis* in an ambiguously supported clade (28/86). The monospecific Conophiini was ambiguously recovered (36/71) as the sister group of *Crisantophis nevermanni*, and both taxa grouped with *Manolepis putnami* in an unsupported clade (13/34). *Thamnodynastes* (26%) was the only genus of Xenodontinae that was not recovered as monophyletic since *T*. *pallidus* did not group with the other species of *Thamnodynastes* (Fig 17). The following polytypic genera were unambiguously or robustly monophyletic: *Borikenophis* (67%), *Cubophis* (83%), *Erythrolamprus* (32%), *Hydrodynastes* (67%), *Magliophis* (100%), *Tropidodryas* (100%), *Apostolepis* (19%), *Arrhyton* (88%), *Echinanthera* (33%), *Hypsirhynchus* (75%), *Ialtris* (50%), *Lygophis* (75%), *Philodryas* (64%), *Pseudalsophis* (67%), *Pseudoboa* (50%), *Psomophis* (100%), *Uromacer* (100%), *Xenodon* (92%), and *Xenopholis* (67%).

The following genera received strong or moderate support: *Helicops* (29%), *Oxyrhopus* (50%), *Phalotris* (31%), *Alsophis* (67%), *Mussurana* (67%), and *Siphlophis* (57%). Although retrieved as monophyletic, *Taeniophallus* (44%) was the only unsupported xenodontine genus in our ML tree (59/42).

Monophyly of the following 12 genera could not be tested because sampling included one species only: *Boiruna* (50%), *Calamodontophis* (50%), *Clelia* (14%), *Conophis* (33%), *Elapomorphus* (50%), *Hydrops* (33%), *Phimophis* (33%), *Pseudoeryx* (50%), *Rodriguesophis* (33%), *Tachymenis* (17%), and *Tomodon* (33%). Polytypic *Emmochliophis*, *Eutrachelophis*, *Saphenophis*, and monotypic *Amnesteophis*, *Coronelaps*, *Ditaxodon*, and *Lioheterophis* were not sampled.

#### Natricidae

We sampled 26 genera (68%) and 89 species (38%) of Natricidae [33] (Fig 18). Natricidae was recovered as a robust clade (92/100) showing an early divergence between two major clades. Moderately supported (72/81) Clade N1 (Fig 18) comprised ten Old World and five New World genera distributed throughout the Paleartic and Neartic realms, respectively. Robustly supported (98/100) Clade N2 (Fig 18) comprised 12 Old World genera distributed throughout the Paleartic, Indo-Malayan, Australasian and Afrotropical realms.

Clade N1 included New World *Thamnophis*, *Adelophis*, *Nerodia*, *Tropidoclonion*, *Regina*, *Clonophis*, *Virginia*, *Haldea*, *Liodytes*, and *Storeria*, and Old World *Natrix*, *Opisthotropis*, *Sinonatrix*, and *Aspidura*. Sri-Lankan *Aspidura* was retrieved as the sister-group of the remaining Old World and New World genera, which clustered together robustly (90/97). Eastern Paleartic *Sinonatrix* and *Opisthotropis* formed a clade without support (60/55), while the western Paleartic *Natrix* and ten New World Neartic genera had a robust association (96/100). The New World natricid radiation composed of *Adelophis*, *Clonophis*, *Haldea*, *Liodytes*, *Nerodia*, *Regina*, *Storeria*, *Thamnophis*, *Tropidoclonion*, and *Virginia* (Fig 18) had robust support (95/100).

Within Clade N1, the Neartic *Aspidura* (57%), *Sinonatrix* (75%), *Opisthotropis* (19%), and *Natrix* (100%) were recovered as unambiguously monophyletic. *Storeria* (60%) was also retrieved as a monophyletic clade with a robust support (99/99). Among the remaining 10 genera, *Tropidoclonion*, *Virginia*, *Haldea*, and *Clonophis* were monotypic; *Adelophis* (50%) was sampled for only one species, and thus monophyly could not be assessed; the polytypic genera *Liodytes* (100%), *Thamnophis* (81%), *Nerodia* (90%) and *Regina* (100%) were not retrieved as monophyletic. Although *Liodytes* formed a strongly supported clade with *Haldea*, *Virginia*, and *Clonophis* (86/95), it was paraphyletic with respect to the latter three genera (Fig 18). However, *Liodytes alleni* clustered strongly (81/96) with a statistically unsupported (59/55) clade formed by *Haldea*, *Virginia*, and *Clonophis*, suggesting the effect of rogue taxa within that assemblage. On the other hand, *Virginia* and *Clonophis* clustered strongly (89/92) together. *Thamnophis* was retrieved as paraphyletic, with *Adelophis* clustering as the sister group of *T*. *melanogaster*, nested deep within an unambiguously supported clade composed by eight other species of *Thamnophis*. *Nerodia floridana* and *N*. *cyclopion* grouped together but formed the sister group of *Tropidoclonion* + *Regina gahamii*, instead of grouping with the other *Nerodia* (Fig 18). The remaining species of *Nerodia* formed a robustly supported clade that was the sister group of *Regina septemvittata*, thus rendering *Regina* polyphyletic (Fig 18).

The second major assemblage of Natricidae–Clade N2 –included *Afronatrix*, *Amphiesma*, *Atretium*, *Balanophis*, *Hebius*, *Lycognathophis*, *Macropisthodon*, *Natriciteres*, *Rhabdophis*, *Trachischium*, *Tropidonophis*, and *Xenochrophis*, and was retrieved with three robustly or unambiguous monophyletic components. The first component was robustly supported (99/99) and included the Indo-Malayan *Trachischium* and *Hebius*. The second component was also robustly supported (90/94), being composed by the Indo-Malayan *Macropisthodon*, the Afrotropical *Afronatrix* and *Natriciteres*, and insular *Lycognathophis* from the Seychelles Archipelago. The third, unambiguously supported component was composed by the Indo-Malayan *Amphiesma*, *Xenochrophis*, and *Balanophis*, the Australasian *Tropidonophis*, and the Indo-Malayan/Paleartic *Rhabdophis* and *Atretium*. The clades formed by *Afronatrix*, *Macropisthodon*, *Natriciteres*, and *Lycognathophis*, as well as *Amphiesma*, *Atretium*, *Balanophis*, *Tropidonophis*, *Rhabdophis*, and *Xenochrophis* clustered together in a strongly supported clade of strictly Asiatic and Indo-Malayan components (83/88).

Monophyly of polytypic *Natriciteres* (33%) and *Hebius* (10%) were unambiguously and moderately (74/80) supported, respectively, while *Rhabdophis* (14%) was only ambiguously supported (75/31). *Atretium* (100%) and *Xenochrophis* (46%) were paraphyletic with respect to each other (Fig 18). *Afronatrix*, *Amphiesma*, *Balanophis*, and *Lycognathophis* were monotypic, and *Macropisthodon* (25%), *Trachischium* (20%), and *Tropidonophis* (5%) were represented by only one species, thus precluding a test of their monophyly.

Monotypic *Amphiesmoides*, *Anoplohydrus*, *Helophis*, *Isanophis*, *Parahelicops*, *Pararhabdophis*, and *Paratapinophis* and polytypic *Herpetoreas*, *Hologerrhum*, *Hydrablabes*, *Hydraethiops*, and *Limnophis* were not sampled herein.

#### Sibynophiidae, Calamariidae and Grayiidae

Sibynophiidae (95/99) and Grayiidae (99/100) were robustly monophyletic and Calamariidae (78/97) was strongly supported (Fig 19). The sampling for these families was strikingly unequal. The less diverse Sibynophiidae and Grayiidae were sampled comprehensively, but moderately diverse Calamariidae was sampled poorly (S4 Table). Our sample of Sibynophiidae corresponded to the two recognized genera (100%), *Scaphiodontophis* and *Sibynophis*, and six of the 11 species (55%). Grayiidae was sampled for three of the four (75%) species described so far for its single Afrotropical genus *Grayia*. On the other hand, our sample of Calamariidae corresponds to two of the seven genera (29%) and only three of the 89 species described (3%).

Within Sibynophiidae, Indo-Malayan *Sibynophis* (55%) received moderate support (72/88) and its species formed two unambiguously supported clades: 1) *S*. *triangularis*, *S*. *collaris* and *S*. *chinensis*; 2) *S*. *bistrigatus* and *S*. *subpunctatus*. We only sampled one species from Neotropical *Scaphiodontophis* (50%), and, thus, its monophyly was not tested.

*Grayia* (75%) was retrieved robustly (99/100). *Grayia smithii* clustered together with *G*. *ornata* in a moderately supported clade (93/78), which was retrieved as the sister group of *G*. *tholloni*.

Within Calamariidae, the two sampled species of *Calamaria* (3%) clustered together as the sister group of the only sampled species of *Pseudorabdion* (7%). The very incomplete sample sizes precluded adequate testing on the monophyly of the genera. We did not sample *Calamorhabdium*, *Collorhabdium*, *Etheridgeum*, *Macrocalamus*, and *Rabdion*.

#### Colubridae

We analyzed representatives from 78 genera (71%) and 275 species (37%) of colubrids (Figs 19–21; S4 Table). The family was recovered with ambiguous support (55/90) but presented a main basal division with two well-supported monophyletic groups: unambiguously supported Clade C1 formed by Asiatic Ahaetullinae genera *Ahaetulla*, *Chrysopelea*, and *Dendrelaphis*; and strongly supported Clade C2 (82/89) that included the remaining genera of Colubrinae. We retrieved within the Clade C2 the following well supported clades: 1) robustly supported Clade C3 (86/100) with mainly sub-Saharan *Thrasops*, *Dispholidus*, *Thelotornis*, *Hapsidophrys*, and *Philothamnus*; 2) strongly supported Clade C4 (72/96) holding mainly Paleartic (North African and Eurasian) *Bamanophis*, *Macroprotodon*, *Hemerophis*, *Hemorrhois*, *Spalerosophis*, *Platyceps*, *Dolichophis*, *Hierophis*, *Eirenis*, and the species *Hemerophis zebrinus*; 3) robustly supported Clade C5 (99/100) with the strictly Indo-Malayan *Ptyas* and *Cyclophiops major*; 4) robustly supported Clade C6 (83/94) having strictly Neotropical arboreal *Leptophis*, and *Oxybelis*; 5) robustly supported Clade C7 (90/93) with the strictly Neotropical racers *Mastigodryas* and *Drymoluber*; 6) robustly supported Clade C8 (99/99) with the Neotropical fossorial *Chilomeniscus*, *Sonora*, and *Chionactis*; 7) strongly supported Clade C9 (71/96) including the strictly sub-Saharan *Dipsadoboa* and *Crotaphopeltis*; 8) strongly supported Clade C10 (73/100) holding mostly Eurasiatic and Neartic *Archelaphe*, *Euprepiophis*, *Oreocryptophis*, *Orthriophis*, *Zamenis*, *Rhinechis*, *Oocatochus*, *Coronella*, *Elaphe*, *Senticolis*, *Scotophis*, *Minthonius*, *Pantherophis*, *Pituophis*, *Bogertophis*, *Rhinocheilus*, *Pseudelaphe*, *Arizona*, *Cemophora*, and *Lampropeltis*. Within Clade C10, strictly Neartic *Senticolis*, *Pantherophis*, *Pituophis*, *Bogertophis*, *Rhinocheilus*, *Pseudelaphe*, *Arizona*, *Cemophora*, and *Lampropeltis* formed a robust clade (Clade C11; 92/98) with respect to the remaining Eurasiatic genera belonging to Clade C10.

Although deeper branches within colubrids were generally poorly or not supported at all, most polytypic genera received very high support values. The following polytypic genera had strong to unambiguous support in our analysis: *Ahaetulla* (50%), *Bogertophis* (100%), *Chironius* (59%), *Chrysopelea* (60%), *Coelognathus* (71%), *Coluber* (25%), *Crotaphopeltis* (33%), *Dasypeltis* (58%), *Dendrelaphis* (14%), *Dendrophidion* (13%), *Dolichophis* (100%), *Drymobius* (50%), *Drymoluber* (67%), *Dryocalamus* (33%), *Eirenis* (70%), *Elaphe* (91%), *Euprepiophis* (100%), *Gonyosoma* (83%), *Hapsidophrys* (100%), *Hemorrhois* (100%), *Hierophis* (67%), *Macroprotodon* (100%), *Oligodon* (24%), *Opheodrys* (100%), *Orthriophis* (100%), *Oxybelis* (50%), *Philothamnus* (45%), *Pituophis* (100%), *Platyceps* (36%), *Spilotes* (100%), and *Thelotornis* (50%*)*.

*Spalerosophis* (33%), *Tantilla* (3%), and *Coronella* (67%) received moderate support. *Boiga* (30%), *Salvadora* (50%), *Telescopus* (14%), and *Lampropeltis* (71%) had poor or ambiguous support.

Paraphyletic assemblages were as follows: *Ptyas* (63%) in respect to *Cyclophiops*; *Mastigodryas* (21%) in respect to *Drymoluber*; *Gyalopion* (100%) in respect to *Ficimia*, *Pseudoficimia* and *Sympholis*; *Dipsadoboa* (20%) in respect to *Crotaphopeltis*; and *Pantherophis* (89%) in respect to *Pituophis*. Our analysis retrieved *Lycodon* (42%) (*sensu* [33]) as polyphyletic.

We did not sample the following 23 genera (23%): *Aeluroglena*, *Aprosdoketophis*, *Argyrogena*, *Chapinophis*, *Colubroelaps*, *Dryophiops*, *Elachistodon*, *Geagras*, *Leptodrymus*, *Lepturophis*, *Liopeltis*, *Meizodon*, *Muhtarophis*, *Pliocercus*, *Rhamnophis*, *Rhynchocalamus*, *Simophis*, *Stichophanes*, *Symphimus*, *Tantillita*, *Wallaceophis*, *Xenelaphis*, and *Xyelodontophis*.

### Divergence time estimation

**Fig 22:**
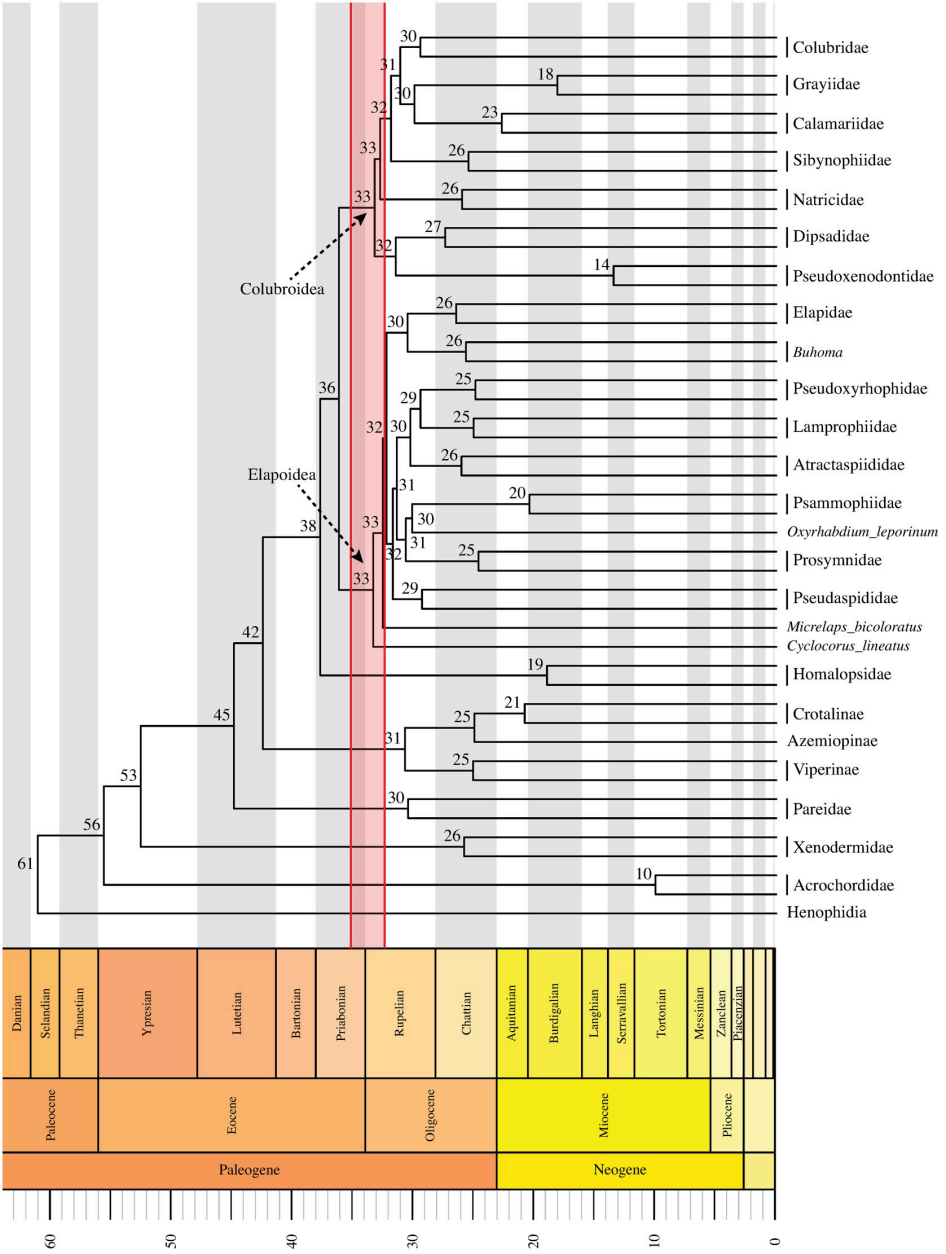
TreePL calibrated tree showing divergence time estimates for the major families of Colubroides. Values above branches represent estimated ages in millions of years. Red-shaded vertical bar corresponds to the possible range of the Eocene–Oligocene interval known as the “Grande Coupure”.

**Fig 23:**
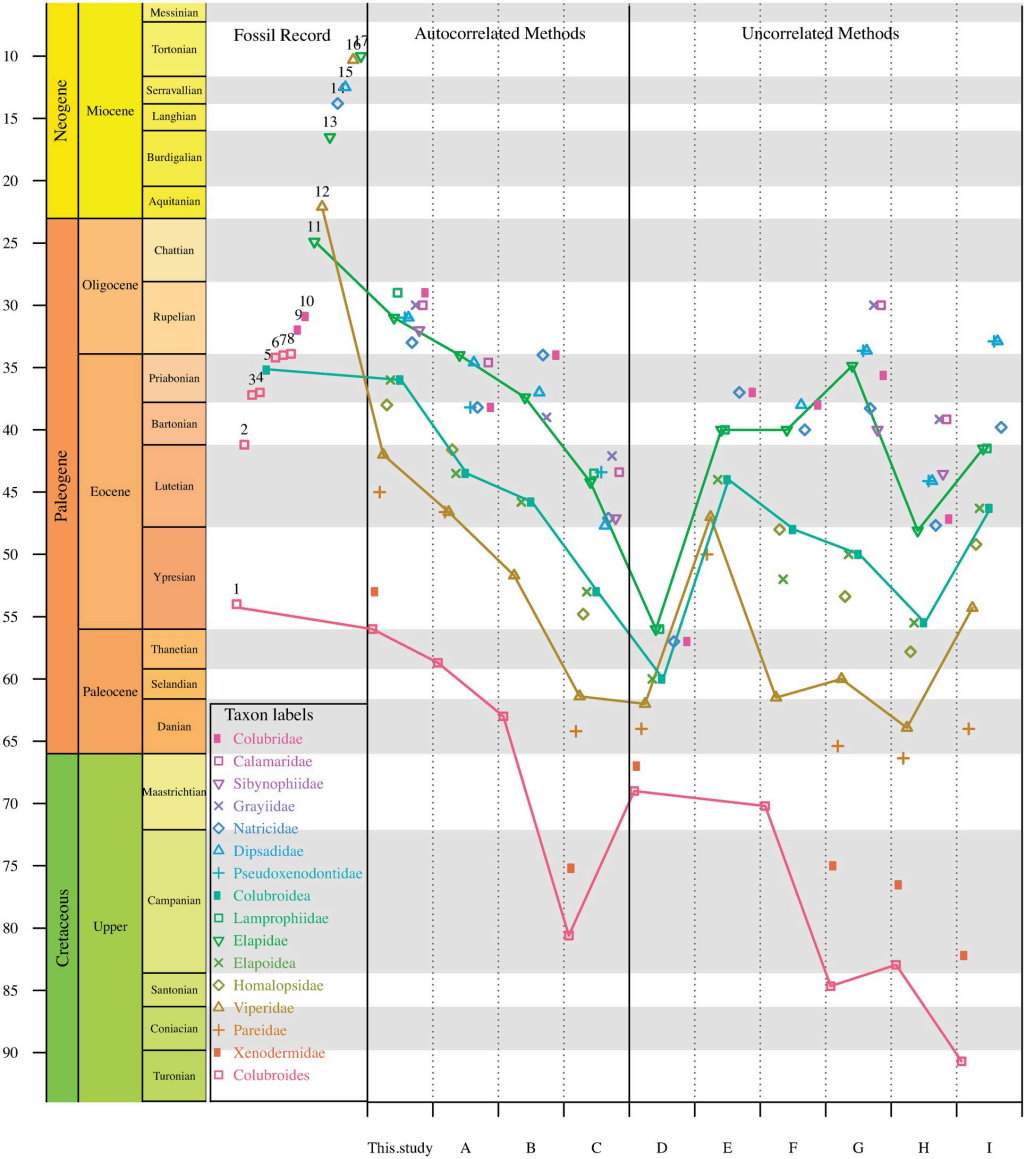
Comparative graph showing divergence time estimations for the main clades of Colubroides in some of the main studies published in the last decade. Numbers near symbols represent the following fossils (see S10 Table for more details): (1) *Procerophis sahnii*, 54 Mya; (2) Colubroidea *Incertae sedis*, 41.2 Mya; (3) Colubroidea *indet*, 37.2 Mya; (4) *Renenutet enmerwer*, 37 Mya; (5) Colubroidea *indet*, 35.2 Mya; (6) *Nebraskophis* sp, 34.2 Mya; (7) Colubrid *indet*, 34 Mya; (8) *Vectophis wardi*, 33.9 Mya; (9) *Texasophis galbreathi*, 32 Mya; (10) *Coluber cadurci*, 30.9 Mya; (11) Elapidae *indet*, 24.9 Mya; (12) *Vipera* cf. *V*. *antiqua*, Mya 22.1; (13) Elapidae *indet*, 16.5 Mya; (14) *Natrix longivertebrata*, 13.8 Mya; (15) *Paleoheterodon tiheni*, 12.5 Mya; (16) *Sistrurus* sp., 10.3 Mya; (17) *Incongruelaps iteratus*, 10 Mya. Letters represent the following contributions: (A) Burbrink and Pyron [132]; (B) Kelly et al. [22]; (C) Zheng and Wiens [26]; (D) Hsiang et al. (58); (E) Hsiang et al. (58); (F) Wuster et al. [133]; (G) Pyron and Burbrink [134]; (H) Alencar et al. [124]; (I) Vidal et al. [135].

The summary of our time-calibrated tree showing the main clades of Caenophidia is given in Fig 22. The complete time-calibrated tree in Newick format is provided in supporting information (S2 Fig). The cross-validation analysis implemented in treePL estimated a divergence time tree by using a smoothing parameter of 10. In our divergence time estimation, we obtained dates for the higher-level clades of Caenophidia that were younger than the dates estimated in recent studies (Fig 23).

A figure illustrating the patterns of cladogenesis throughout time for the whole tree of Caenophidia is given as supporting information (S4 Fig). We discuss these patterns of diversification along with the fossil record in the following section.

## Discussion

The considerable recent efforts to use molecular sequence data to resolve deep interrelationships of caenophidians [24,26,27,28,132–137] have differed substantially in the inversely related sampling of genes and taxa. Most data sets have either many taxa and few genes (MTFG) mostly from Sanger sequencing (e.g., 10 loci for 1343 species) [28], or few taxa and many genes (FTMG) built using next generation sequencing (e.g., 333 loci for 31 species) [137], and many discrepancies in results are likely due to this important difference. FTMG analyses tend to provide strong support for higher-level relationships that may be misleading because of inadequate sampling of key taxa. MTFG studies provide much more information about low-level relationships but tend to fail to provide well-supported resolution of deeper divergences due to the limited number of loci and short branches. An important exception is the hybrid approach of Zhang and Wiens [27] which added many (up to 52) extra genes for a subset (3,8%) of the terminals included in Pyron et al. [26] MTFG analyses.

A priori we might expect similar results from different MTFG studies because they tend to be based on similar data sets (most data retrieved from GenBank) and rely upon similar methods and models of sequence evolution. In contrast, there are considerable discrepancies between these studies throughout the caenophidian tree, suggesting that both taxon and gene samplings, including ours, are still very inadequate. In order to provide more robust grounds for discussion, we have combined SHL and FBP measures to distinguish inferred clades that are unsupported or ambiguously supported and those that are moderately to unambiguously supported (S2 Table). FBP and SHL are complimentary branch support measures [50], with SHL performing better with short branches while FBP searches are more efficient through all topologies [50,51]. Used in combination they can provide greater insights than reliance upon the use of only one of these measures enabling us to distinguish the seemingly more reliable inferred relationships that have congruent high support values from those with low or incongruent support values. Although this approach has limitations, we consider that it provides a sensibly conservative background for caenophidian systematics that can usefully inform possible taxonomic actions motivated by our developing understanding of phylogenetic relationships [27].

### How stable is the molecular Caenophidian tree?

Table 1 summarizes the number of clades within each higher-level and family-level clade on the tree, classified according to the seven categories of combined FBP-SHL support values defined in this study. Our combined clade support approach shows that among the 1264 clades composing the caenophidian tree (Figs 6–21; S1 Fig), only 20.9% (264) are unambiguously supported by both SHL and FBP values, while just over 15% (191) have no SHL or FBP support. Strongly to unambiguously supported clades sum up to 59.7% (755) of all clades recovered in the tree, leaving unsupported to ambiguously supported clades with 36.8% (466) while moderately supported clades represent only 3.4% (43) of all clades in the tree. The numbers of unsolved or questionable phylogenetic hypotheses of relationships within the available caenophidian tree is high and the widespread impression that both higher-level and lower-level colubroidean affinities and taxonomy are well-resolved is inaccurate and should at least be viewed with caution.

**Table 1:** Numbers of inferred clades in each category of combined FBP and SHL support values.

Taxa	no support (light grey)	poor/ambiguous (dark grey)	moderate (green)	strong (blue)	robust (orange)	unambiguous (red)	Total Nodes
**Acrochordidae**	0	0	0	1	0	1	2
**Xenodermidae**	0	0	1	1	0	2	4
**Pareidae**	1	1	0	2	7	0	11
**Viperidae**	27	38	8	25	70	78	246
**Viperinae**	11	6	4	9	16	23	69
**Crotalinae**	16	32	4	16	54	53	175
**Homalopsidae**	2	5	0	4	6	7	24
**Psammophiidae**	7	4	0	7	12	10	40
**Atractaspididae**	2	3	1	2	6	3	17
**Atractaspidinae**	2	1	1	0	2	0	6
**Aparallactinae**	0	2	0	2	3	3	10
**Lamprophiidae**	6	7	0	3	4	9	29
**Pseudoxyrhophiidae**	4	5	3	6	21	15	54
**Elapidae**	34	44	3	23	55	36	195
**Hydrophiinae**	20	28	1	14	30	27	120
**Pseudoxenodontidae**	0	0	0	0	2	1	3
**Dipsadidae**	42	76	8	31	52	29	238
**Carphophiinae**	0	1	0	0	0	1	2
**Dipsadinae**	16	25	2	7	8	8	66
**Xenodontinae**	26	47	6	23	40	18	160
**Natricidae**	9	9	6	14	31	19	88
**Sibynophiidae**	0	0	1	0	2	2	5
**Calamariidae**	0	0	0	1	0	1	2
**Grayiidae**	0	0	0	1	1	0	2
**Colubridae**	50	73	10	30	66	46	275
**Higher Levels**	5	8	2	1	3	3	22
**Pseudaspididae**	0	1	0	0	1	1	3
**Prosymnidae**	2	1	0	0	0	1	4
**Total**	**191**	**275**	**43**	**152**	**339**	**264**	**1264**
	15.1%	21,80%	3.4%	12%	26,80%	20.9%	

Clade support values based on the combination of FBP and SHL, classified in seven categories, as follows: red, unambiguous support (both methods recover values of 100%); orange, robust support (both methods recover values ≥ 90%, or ≥ 80% in one method and 100% in the other); blue, strong support (both methods recover values ≥ 80%, or values ≥ 70% in one method and ≥ 90% in the other); green, moderate support (both methods recover values ≥ 70% but do not reach values equal to previous categories); dark grey, ambiguous support (highly discrepant values, with < 70% in one method and ≥ 80% in the other) or poor support (values < 70% in one method and between 70% and 80% in the other method); light grey, unsupported (values < 70% for both methods).

However, unsupported to poorly/ambiguously supported clades have uneven distributions throughout the tree. Among the most diverse families, Dipsadidae has the highest percentage of questionable clades, with 49.5% (118) being unsupported or having poor or ambiguous support. Colubridae, Lamprophiidae, and Elapidae are slightly less unstable only, with 44.7% (123), 44.8% (13), and 40.0% (78) of their clades having poor or ambiguous support. In contrast, although similarly diverse, relationships within Viperidae are better supported, with only 26.4% of all clades being unsupported to poorly/ambiguously supported. The remaining, less diverse, Homalopsidae (29.2%), Psammophiidae (27.5%), Atractaspididae (29.4%), Natricidae (20.5%), Pseudoxyrhophiidae (16.6%), and Pareidae (18.2%) are similar, with only 16 to 30% of their internal clades being statistically questionable.

Within Dipsadidae, unsupported to poorly/ambiguously supported clades are concentrated in the subfamily Dipsadinae (62.1%), suggesting that synonymyzations of *Sibynomorphus* with *Dipsas* or of *Geophis* with *Atractus* might still be premature. Similarly, the definition of new generic boundaries within Tachymenini must also await more robust inferences of relationships. Although the tribe Conophiini is retrieved in our analysis, including the genus *Crisantophis* [21,29], support values for that clade are unimpressive and an expanded molecular analysis of this group is needed in order to test its monophyly.

Within Colubridae, the non-monophyly of *Ptyas*, *Mastigodryas*, *Gyalopion*, *Dipsadoboa*, *Zamenis*, *Lycodon*, and *Pantherophis* requires a more thorough examination.

Within Viperidae, deeper relationships in viperine snakes receive ambiguous or no support at all. This coincides with recent molecular trees yielding substantially different results [24,26,27,28,124]. In contrast, most higher-level affinities of crotalines seem to be well resolved. Notwithstanding, *Causus* occupies an unresolved basal position within viperines. Pyron et al. [26] reported that it nests as the sister group of *Echis* with very high SHL, but Figueroa et al. [28] retrieved it as the sister-group of *Proatheris*, albeit with very low SHL.

Low clade support within Elapidae concentrates within the Australo-Melanesian radiation of marine elapid snakes (90/99), or Hydrophiinae, with 40.1% (48) of its clades receiving poor or ambiguous support.

### Independent morphological evidence

Key morphological attributes of colubroidean cranial, vertebral and hemipenial anatomies coincide with some of the higher-level clades in our molecular phylogeny. Description of most of these morphological characters were already addressed in the literature [1,29,30,138,139], but have been underused in the past due to the overall insipient knowledge of colubroidean affinities prior to the consolidation of large-scale molecular phylogenetic analyses. Some characters are unambiguously optimized in the molecular tree, but others support competing molecular topologies and suggest that further studies are necessary to test alternative hypotheses.

The three most recent large-scale molecular phylogenies [26,27,28] and our study invariably retrieve the following relationships with unambiguous support values: 1) a monophyletic Caenophidia (Acrochordoidea + Colubroides), 2) the families Xenodermidae, Pareidae, and Viperidae arising from basal splits within Colubroides, and 3) a monophyletic Colubroidea [23,26,28,137]. These studies also report well-supported clades representing the Xenodermidae (excluding *Oxyrhabdium*), Pareidae, Viperidae, Psammophiidae, Lamprophiidae, Pseudoxyrhophiidae (including *Duberria*), Atractaspididae (excluding *Micrelaps*), Elapidae (excluding *Homoroselaps*), Pseudaspididae, Pseudoxenodontidae, Dipsadidae, Natricidae, Calamariidae, Grayiidae, Sibynophiidae, and Colubridae. However, apart from these consensual results, affinities between these main clades, and between them and a number of genera with uncertain phylogenetic affinities remain controversial.

Diverging results in higher-level caenophidian affinities between Pyron et al. [26], Zheng and Wiens [27], Figueroa et al. [28], and this study concentrate mostly around the base of the colubroidean tree. These include the position of xenodermids (as sister group to acrochordids or to the remaining colubroideans), pareids (as sister group to Viperidae or to Endoglyptodonta), homalopsids (as sister group to Elapoidea or to Elapoidea + Colubroidea), and the interrelationships within elapoid and colubroid families.

#### Early diverging caenophidian lineages

The phylogenetic affinities of acrochordids have long been contentious, with conflicting morphological evidence suggesting their basal divergence within Alethinophidia [8] or a more nested position within Caenophidia [6,7,9,13,29,80,140]. Recently, acrochordids were unambiguously placed within alethinophidian snakes in both morphological and molecular analyses [23,26,58,141] and never retrieved as part of the basal split within alethinophidians. Notwithstanding, most analyses provide conflicting signals for the position of acrochordids with respect to colubroideans, the former being retrieved either as the sister-group of colubroideans [23,24,132,137,141] or nested within the latter as the sister-group of xenodermids [17,26] or even as the sister-group of Colubriformes (i.e., excluding xenodermids from the latter) [58,142]. On the other hand, all recent analyses rejected unanimously an association between acrochordids and Natricidae [80] or Homalopsidae [143].

**Fig 24:**
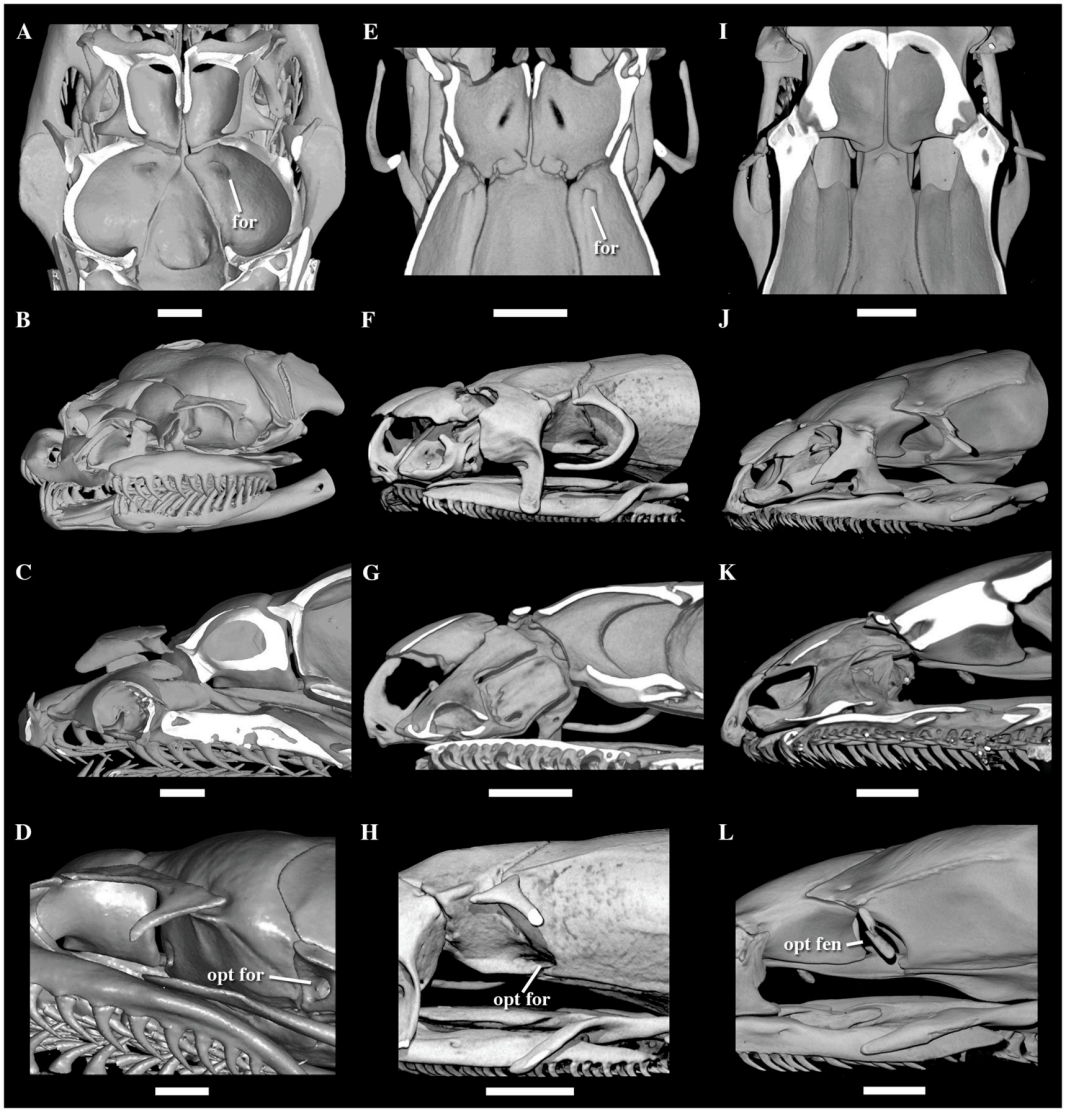
Skulls of *Acrochordus granulatus* (A-D), *Fimbrios klossi* (E-H), and *Xylophis perroteti* (I-L). Three-dimensional surface and cutaway views based on high resolution X-ray computed tomography. A, E, I, dorsal three-dimensional cutaway views along the frontal axis; B, F, J, oblique three-dimensional surface views; C, G, K, left lateral three-dimensional cutaway views along the sagittal axis; D, H, L, left lateral three-dimensional surface views. Legends: *for*., optic foramina; *opt*. *fen*., optic fenestra. Scale bar = 1mm.

**Fig 25:**
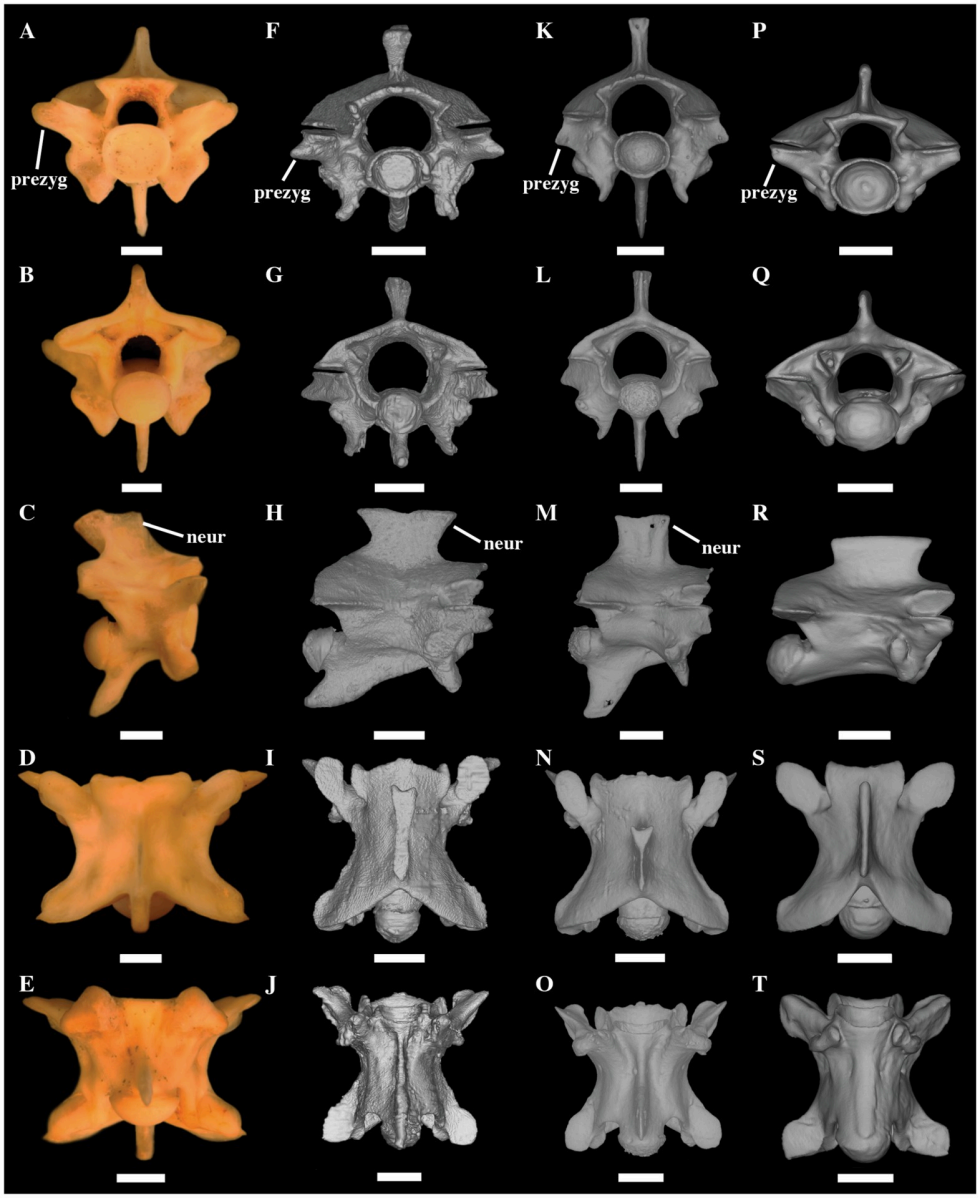
Mid- to posterior trunk vertebrae of *Acrochordus javanicus* (A-E), *Achalinus rufescens* (F-J), *Fimbrios klossi* (K-O), and *Pareas* sp. (P-T). Photographs (A-E) and three-dimensional surface views based on high resolution X-ray computed tomography. A, F, K, P, anterior views; B, G, L, Q, posterior views; C, H, M, R, right lateral views; D, I, N, S, dorsal views; E, J, O, T, ventral views. Legends: prezyg., prezygapophysial process; neur., neural spine. Scale bar = 2 mm (A-E) and 1 mm (F-T).

Three strikingly conspicuous morphological characters shared by acrochordids and the xenodermids *Achalinus*, *Xenodermus*, and *Fimbrios* support a sister-group relationship between them: 1) a distinct foramen for the optic nerve that opens on the anteriormost ventrolateral surface of the parietal (Fig 24; Figs A–C in S1 Appendix); 2) a foramen of unknown function in the dorsal surface of the laterosphenoid [140]; and 3) a vertically oriented blade-like prezygapophyseal accessory process (Fig 25; Fig A in S2 Appendix). Here, we report for the first time vertically oriented blade-like prezygapophyseal accessory processes in Xenodermids (Fig 25; Fig A in S2 Appendix). *Acrochordus* and *Xenodermus* also share an unusual karyotypic formula of 2n = 32 chromosomes, which might represent a fourth character shared exclusively by acrochordids and xenodermids [144–146].

On the other hand, our molecular results place xenodermids as the sister group of the remaining colubroideans [23,26,137] with unambiguous support (100/100). Additionally, our analysis includes new sequences for *Fimbrios*, which corroborate the study of Teynié et al. [147] and reinforce our topology.

**Fig 26:**
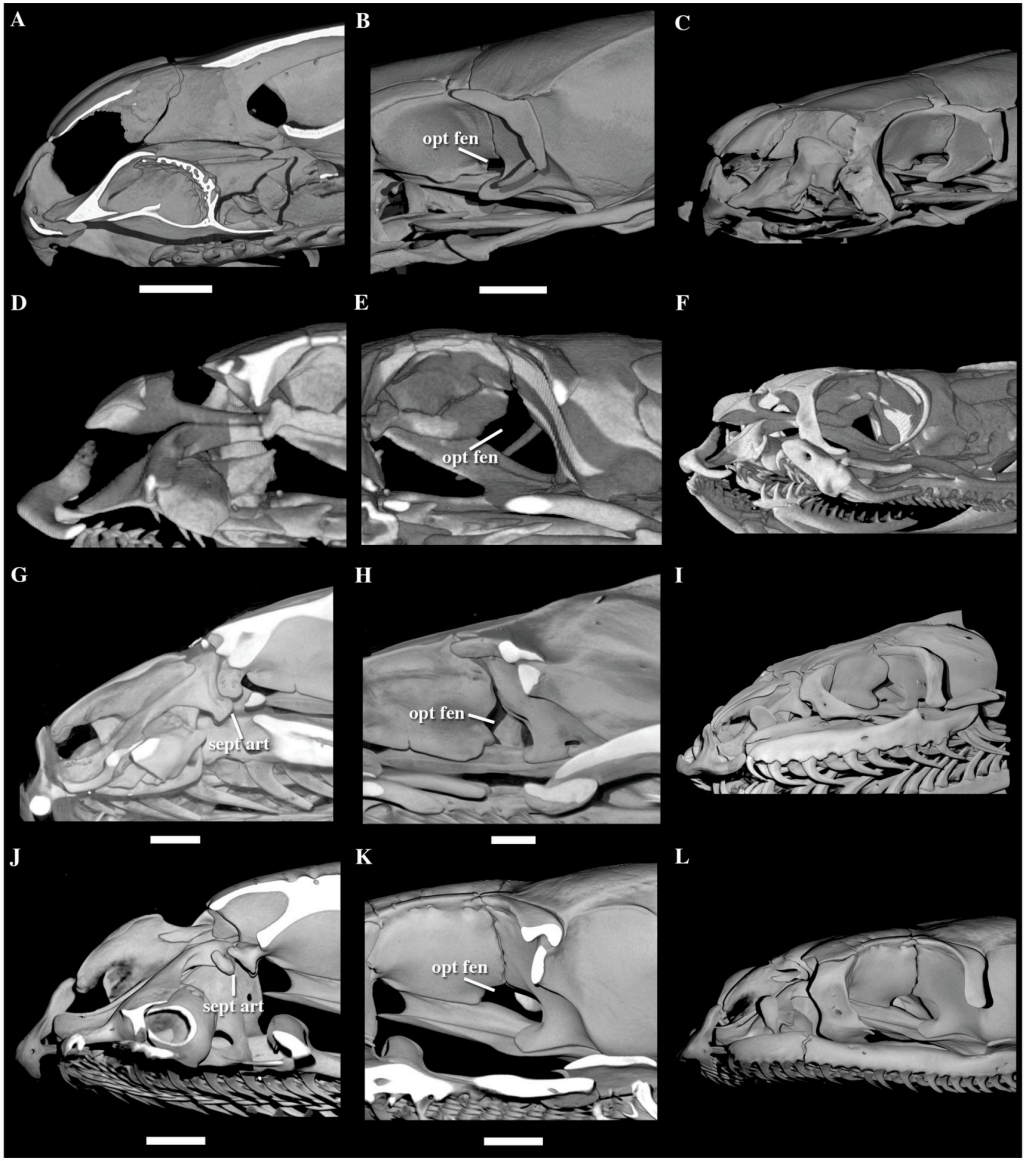
Skulls of *Pareas moellendorffi* (A-C), *Causus rhombeatus* (D-F), *Enhydris chinensis* (G-I), and *Afronatrix anoscopa* (J-L). Three-dimensional surface and cutaway views based on high resolution X-ray computed tomography. A, D, G, J left lateral three-dimensional cutaway views along the sagittal axis; B, E, H, K, left lateral three-dimensional surface views; C, F, I, L, oblique three-dimensional surface views. Legends: sept. art., septomaxillary articulation; opt. fen., optic fenestra. Scale bar = 1 mm.

**Fig 27:**
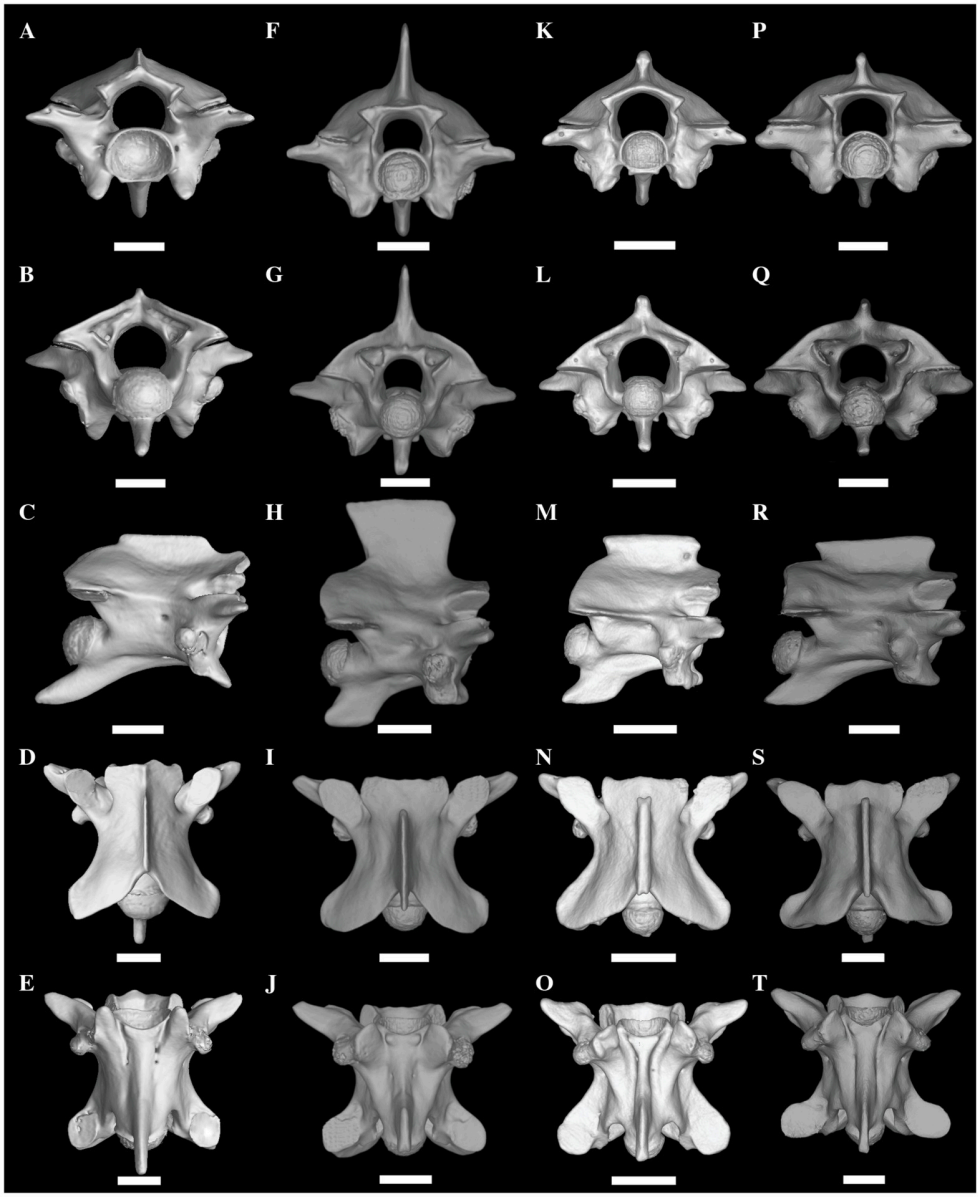
Mid- to posterior trunk vertebrae of *Causus difilippi* (A-E), *Cerberus rynchops* (F-J), *Cyclocorus lineatus* (K-O), and *Sinomicrurus macclellandi* (P-T). Three-dimensional surface and cutaway views based on high resolution X-ray computed tomography. A, F, K, P, anterior views; B, G, L, Q, posterior views; C, H, M, R, right lateral views; D, I, N, S, dorsal views; E, J, O, T ventral views. A-E, scale bar = 5 mm; F-J, scale bar = 2mm; K-O, scale bar = 1mm; P-T, scale bar = 1mm.

Two uniquely derived morphological characters support the clade Colubroides with the exclusion of acrochordids: 1) expanded costal cartilages [148,149]; and 2) a deeply curved (concave) border of the septomaxilla delimiting the opening of the vomeronasal organ and contacting the vomer laterally to the latter in ventral view [30]. This hypothesis suggests that the three characters shared by acrochordids and xenodermids either evolved independently in these two groups or they are synapomorphies of the clade Caenophidia that were secondarily lost in the ancestor of Colubriformes. Two additional derived characters–a uniformly blade-like neural spine reaching the roof of the zygosphene (Fig 25), and the septomaxillae contacting the frontals (Fig 26)–have been usually thought to represent synapomorphies of the clade Colubroides to the exclusion of acrochordids [30,31]. However, our observations reveal that *Achalinus*, *Xenodermus*, and *Fimbrios* also lack both characters (Figs 24 and 25; Figs B and C in S1 Appendix; Fig A in S2 Appendix, respectively), which likely represent synapomorphies of the clade Colubriformes (Figs 26 and 27) (see below). Despite the large amount of evidence at hand, the phylogenetic affinities of acrochordids and xenodermids within caenophidians remain contentious and deserve further investigation.

Although pareids and homalopsids are clearly positioned within the clade Colubriformes, their precise phylogenetic affinities within that group were still controversial. In both large-scale FTMG and MTFG molecular analyses, pareids appear either as the sister group of Viperidae or as the sister group of Endoglyptodonta, while homalopsids are positioned either as the sister group of Elapoidea [19,23,136,137] or as the sister group of Elapoidea + Colubroidea [26,28]. Our results contradict both Pyron et al. [26] and Figueroa et al. [28] since we recover Pareidae as the sister-group of the robustly supported clade Endoglyptodonta (99/99), while the Homalopsidae are retrieved as the sister-group of the moderately supported clade formed by elapoids and colubroids (83/79) (Figs 1 and 2). Results in which homalopsids are the sister-group of elapoids [26,28] may find some support through independent morphological evidence since the former group shares an overal similar hemipenial morphology with the Pseudoxhyrhophiidae [29], including lobes covered by densely packed, diminute spinules (Figs G-I, M, N in S3 Appendix). However, other elapoid lineages have a very uniform and distinct hemipenial morphology (S3 Appendix), suggesting that homalopsids and pseudoxyrhophiids evolved their hemipenial similarities independently.

**Fig 28:**
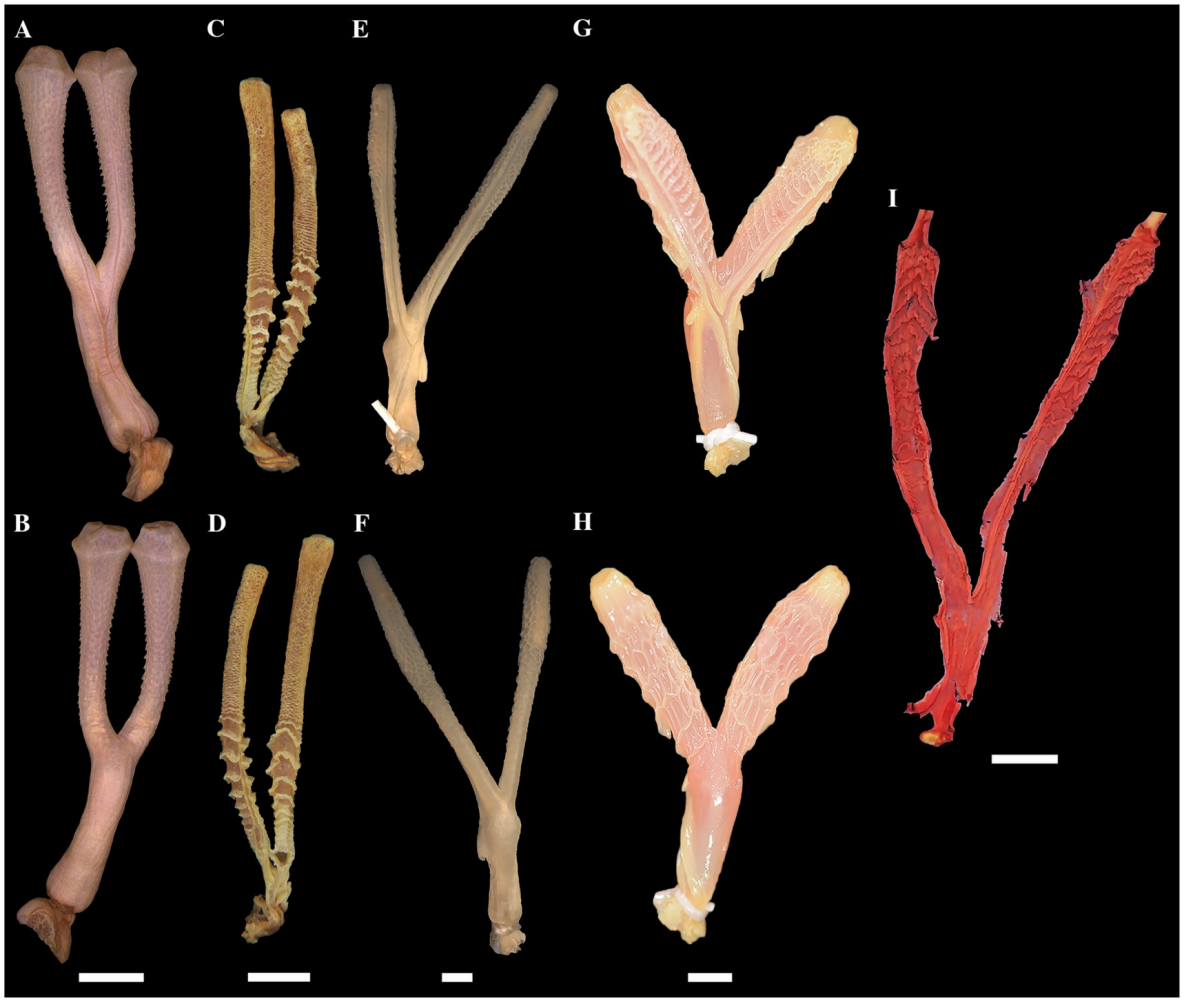
Hemipenes of *Acrochordus javanicus* (A-B), *Achalinus rufescens* (C-D), *Pareas monticola* (E-F), *Asthenodipsas malaccanus* (G-H), and *Xylophis perroteti* (I). A, C, E, G, sulcate views; B, D, F, H, asulcate views. A, B, E, F, G, H, fully everted and expanded; C, D, fully everted and partially expanded; I, opened through a longitudinal slit, spread flat, and dyed with alizarin red. A-B, scale bar = 5 mm; C-D, scale bars = 2 mm; E-F, scale bar = 1 mm; G-H, scale bar = 1 mm; I, scale bar = 5 mm.

**Fig 29:**
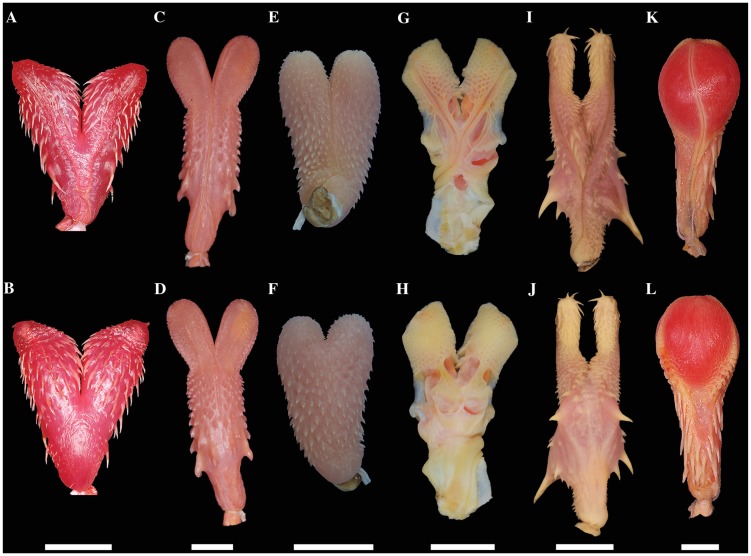
Hemipenes of *Porthidum nasutum* (A-B), *Brachyorrhos albus* (C-D), *Atractaspis fallax* (E-F), *Oreocalamus hanitschi* (G-H), *Grayia ornata* (I-J), and *Spilotes sulphureus* (K-L). A, C, E, G, I, K, sulcate views; B, D, F, H, J, L, asulcate views. A-L completely everted and expanded. A-D, Scale bars = 5 mm; E-F, scale bar = 10 mm; G-H, scale bar = 5 mm; I-J, Scale bars = 10 mm.

Our Colubriformes has been unambiguously recovered in FTMG analyses [136,137], and is supported by four uniquely derived morphological characters: 1) a blade-like, uniformly thin neural spine that invades the roof of the zygosphene (Figs 25 and 27); 2) an optic fenestra floored by the parasphenoid (the frontal suboptic process that contacts the parietal ventrally to the ophthalmic nerve is lost) (Figs 24 and 26) [1]; 3) septomaxillae contacting the prefrontals (Figs 24 and 26) [6,29,30]; and 4) well-defined calyces arranged in rows on the hemipenial lobes (Figs 28 and 29) (see also S1–S3 Appendices). The medial process of the septomaxilla appears to project posteriorly and contact the frontals only in the clade Colubriformes.

Differing from colubriforms but similar to the condition in acrochordids and tropidophiids (Fig 24; Fig A in S1 Appendix), the medial process of the septomaxilla in *Achalinus*, *Xenodermus*, and *Fimbrios* does not contact the frontals, but rather expands in a blade-like process that contacts the nasal dorso-posteriorly (Figs B and C in S1 Appendix). In *Xylophis*, the medial process of the septomaxilla approaches the colubriform condition since it does not expand in a dorso-posteriorly directed lamina but forms a finger-like process that extends posteriorly without reaching the frontals (Fig 24). The septomaxilla of pareids contacts broadly the ventral surface of the frontal [30] (Fig 26; Fig D in S1 Appendix), while the septomaxillary-frontal contact appears to be reduced or absent in most viperids, except in *Azemiops*, *Atheris*, and *Causus*, in which it is definitely present (Fig 26; Fig E in S1 Appendix). *Azemiops* and *Causus* are the sister groups to the two main lineages of crotalines and viperines, respectively, and, for this reason, we consider that the contact between the two bones was secondarily lost in both subfamilies, probably due to the specialization that occured in the snout and venom delivery system of viperids [30]. The septomaxillary-prefrontal contact further evolved as a complex prokinetic joint [30] in homalopsids, elapoids, and colubroids, representing instead a uniquely derived feature of that clade (see below).

The optic nerve usually emerges from the braincase between the frontal and parietal bones in snakes. In its plesiomorphic condition, the frontal and parietal meet below the optic nerve (e.g., *Tropidophis nigriventris*; Fig A in S1 Appendix), whereas in its derived condition this contact is lost and the parasphenoid participates broadly in the ventral border of the optic foramen (Fig 26) [1,150]. The xenodermids *Achalinus*, *Fimbrios*, and *Xenodermus* retain the plesiomorphic condition, while *Xylophis* shares the derived condition present in colubriforms (Fig 24; Figs B and C in S1 Appendix).

Calyces are secondarily lost many times within higher colubriforms, and more conspicuously in all elapoids, natricids, and in several less inclusive lineages within colubroids. However, well-defined calyces are definitively present in pareids, viperids, pseudoxenodontids, colubrids, dipsadids, calamariids, and sibynophiids (Figs 28 and 29; S3 Appendix) [29]. Calyces are absent in xenodermids, which retain thin-walled flounces or spines as lobular ornamentations [29] (Fig 28; Figs A and B in S3 Appendix). Among the examined hemipenes of xenodermids, *Achalinus* is the only exception in which the more distal part of the lobes is ornamented by a dense arrangement of closely packed longitudinal flounces that tend to form small calyces (Fig 28; Fig B in S3 Appendix). The condition in *A*. *rufescens* appears to be independently acquired within xenodermids. The hemipenis of “basal” caenophidians retains a similar overall morphology that includes a centrolinar sulcus, extremely long and ornamented cylindrical lobes and short unornamented hemipenial body with a sulcus spermaticus dividing centrolinearly (Fig 28). This overall morphology is not lost in higher endoglyptodont lineages (Fig 29).

**The genus *Xylophis***—Although not included in our molecular analysis, we investigated the skull, vertebrae, and one hemipenis of *Xylophis perroteti*. Two recent molecular analyses suggested that it might be either nested inside natricids or the sister-group of pareids [151,152]. As shown above, *X*. *perroteti* retains two of the four putative colubriform morphological synapomorphies (1 and 2) and none of the xenodermid shared derived features, supporting the view that the genus does not belong to the latter family. Unlike xenodermids and acrochordids, *Xylophis* lacks a vertically oriented blade-like prezygapophyseal accessory processes and the distinct parietal and laterosphenoid foramina characteristic of the latter two groups. Furthermore, *Xylophis* shares with Colubriformes the presence of a uniformly thin neural spine reaching the roof of the zygosphene and an optic fenestra floored by the parasphenoid (Fig 24). These uniquely derived features support the inclusion of *Xylophis* in the clade Colubriformes. The hemipenis of *Xylophis* differs significantly from the hemipenial morphology of endoglyptodont lineages (Figs 28 and 29; S3 Appendix). It is deeply bilobed, with extremely long, cylindrical lobes covered with longitudinal flounces, and a short unornamented hemipenial body with a sulcus spermaticus dividing centrolinearly near the base of the crotch (Fig 28; Fig D in S3 Appendix). The overall hemipenial morphology of *Xylophis* is very similar to those of Pareidae and Xenodermidae (Fig 28; Figs A–D in S3 Appendix). All pareids and xenodermids examined share with *Xylophis* extremely long cylindrical lobes, a short unornamented hemipenial body, and a centrolineal sulcus spermaticus. However, when compared to Xenodermidae, *Xylophis* differs from *Fimbrios* and *Xenodermus* by the absence of lobular spines (well developed in *Fimbrios*), and shares with *Achalinus* longitudinally disposed flounces surrounding the lobes. Similarly, differences are also observed with Pareidae hemipenial morphology. Although there is an indication of flounces on the hemipenis of *Asthenodipsas*, *Xylophis* differs from this genus and also from *Aplopeltura* and *Pareas* by the absence of calyces on the lobes.

The deeply bilobed hemipenial condition is not exclusive of xenodermids. It also occurs in all members of “basal” caenophidian lineages (*Acrochordus*, xenodermids, and pareids). Although xenodermids and pareids are included in Colubroides, they share with *Acrochordus* the lack of ornamentations covering their hemipenial body [29]. In contrast, all endoglyptodont colubroideans, with some exceptions due to secondary changes, bear a spiny hemipenial body. These two conditions may be plesiomorphic for Caenophidia (including *Acrochordus*) that persists in “basal” colubroideans, being secondarily lost in the clade Endoglyptodonta (with only some exceptions; e.g., *Xenodon merremi*).

The morphological evidence described above supports a deep divergence of *Xylophis* within the clade Colubriformes, while it is morphologically and ecologically distinct from its possible sister group, the Pareidae [152], warranting familial recognition as a separate assemblage of non-endoglyptodont colubroideans. While this study was in review, Deepak et al. [153] provided a molecular phylogenetic study retrieving *Xylophis* as the sister-group of Pareidae, outside the Endoglyptodont clade, and described a new subfamily Xylophiinae to accommodate the genus. Deepak et al.’s [153] conclusions are supported by Ruane and Austin’s [152] previous molecular results and our own morphological evidence.

#### Endoglyptodont lineages

**Fig 30:**
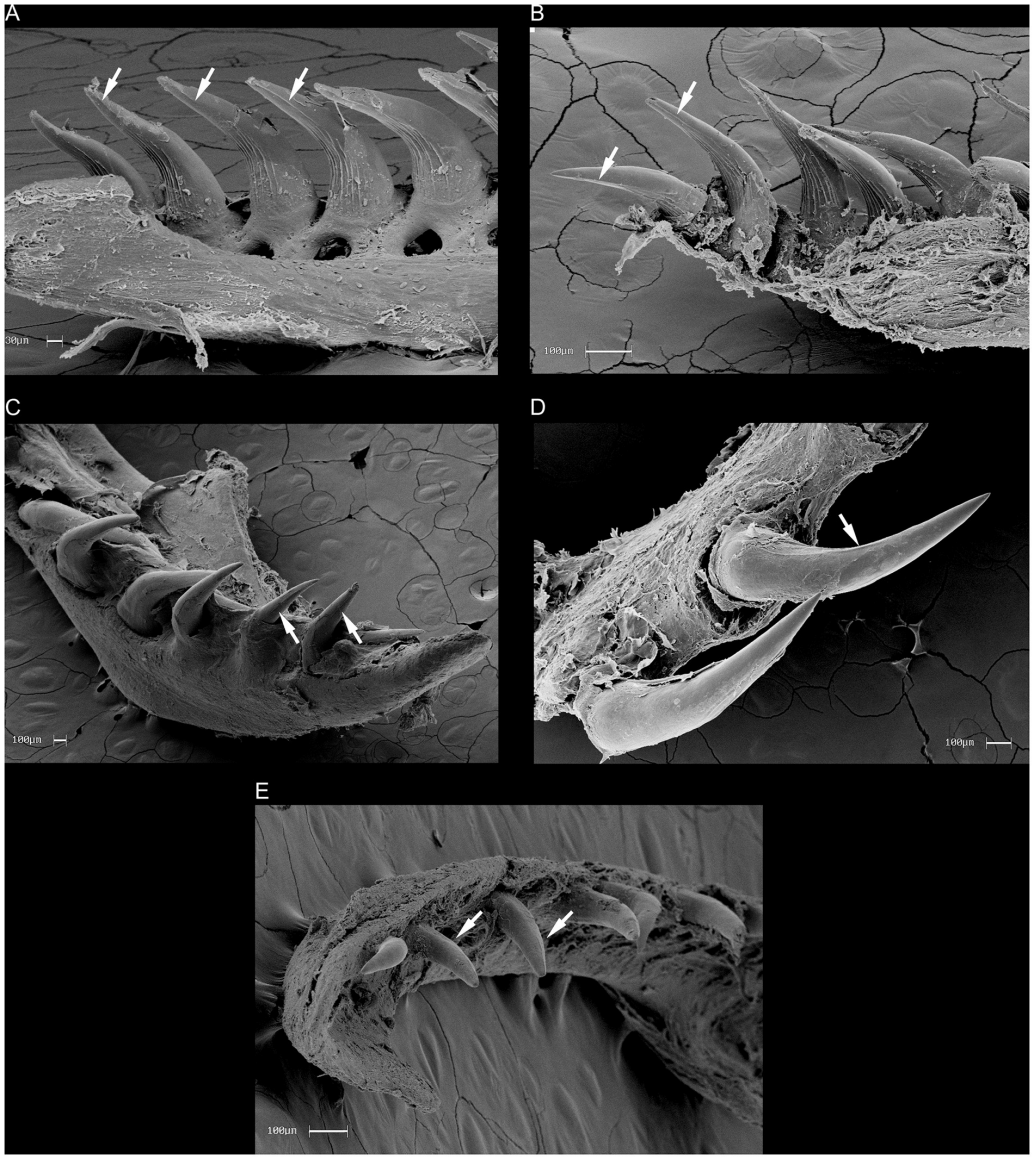
Scanning electron microscopy of maxillary teeth in Acrochordidae, Xenodermidae, Pareidae, and Xylophiinae. (A) right posterior maxillary teeth of *Achalinus formosanus* in lingual view; (B) right posterior maxillary teeth of *Achalinus rufescens* in lingual view; (C) right posterior maxillary teeth of *Acrochordus granulosus* in labial view; (D) left posterior maxillary teeth of *Pareas* sp. in lingual view; (E) right posterior maxillary teeth of *Xylophis perroteti* in labial view. White arrows are pointing to the lateral (labial) and medial (lingual) ridges in the posterior maxillary teeth.

Recovery of a robust Endoglyptodonta (99/99) necessitates rejection of Figueroa et al.’s [28] hypothesis of sister-group relationship between Pareidae and Viperidae (Fig 1A). Endoglyptodonts are also unambiguously supported in FTMG analyses [136,137] and are corroborated by at least three uniquely derived morphological characters: 1) sulcate maxillary teeth functionally associated with a serous dental gland [23,154]; 2) posterior maxillary teeth with anterior and posterior ridges (Fig 30); and 3) spines present on (ornamenting) the hemipenial body (Fig 29; Figs E–AD in S3 Appendix). Although typically sulcate tooth morphologies occur in early diverging lineages of endoglyptodonts (viperids and homalopsids) and are secondarily lost within elapoids and colubroids, all endoglyptodonts share the presence of anterior and posterior ridges in their posterior maxillary teeth. Here, we confirmed that the plesiomorphic condition of lateral and medial ridges in the posterior maxillary teeth are retained in acrochordids, xenodermids, pareids, and *Xylophis* (Fig 30). Thus, the appearance of distinct anterior and posterior maxillary ridges in posterior maxillary teeth is a synapomorphy of the clade Endoglyptodonta instead of Colubroides. Anterior and posterior maxillary ridges seem to be intimately correlated with the appearance of venom injecting sulcate teeth, suggesting that the specialized posterior maxillary dental lamina from which fangs develop [155] is actually absent in these early diverging colubroidean lineages. Similarly, hemipenial body spines are known to occur only in endoglyptodonts among snakes (except in some uropeltids and scolecophidians). They occur uniformly in viperids, homalopsids and higher endoglyptodont lineages, and are lost secondarily in a variety of taxa within elapoids and colubroids. They are absent in acrochordids, xenodermids, pareids, and *Xylophis* (Figs 28 and 29; S3 Appendix) [23]. Endoglyptodonts may also share a fourth hemipenial synapomorphy illustrated by an expansion of the hemipenial body and a sharp reduction in length of the hemipenial lobes, the latter normally not exceeding twice the size of the former (with few exceptions, such as *Pseudaspis cana*). In contrast, acrochordids, xenodermids, pareids, and *Xylophis* uniformly retain very elongated, narrow tubular lobes and short hemipenial bodies (Fig 28; S3 Appendix) [23].

All recent FTMG and MTFG molecular analyses have unambiguously resolved the clade formed by homalopsids, elapoids, and colubroids [19,23,26,28,136,137]. Homalopsids and higher endoglyptodonts are characterized by a complex prokynetic joint attachment of the snout with the braincase, which is established by the medial flange of the septomaxillae that forms a widened condylar process that articulates with a cotylar process on the anteroventral edge of the frontal (Fig 26G and 26J) [30]. As mentioned previously, a septomaxillary-frontal contact is absent in acrochordids and xenodermids (Fig 24A and 24C), while pareids and basal viperids retain a simple contact that does not form distally widened processes (Fig 26A and 26D). Within viperids, a contact between the septomaxillae and frontals occurs in the “basal” taxa *Causus* and *Azemiops*. *Causus* retains an insipient articular facet that probably evolved independently from higher endoglyptodonts [30] (Fig 26D; Fig E in S1 Appendix). Differently from *Causus*, *Atheris* retains a simple contact between the septomaxillae and frontals [30]. The septomaxillae do not reach the frontals in most crotalines and viperines [30]. On the other hand, Cundall and Irish [30] described a complex prokynetic articular joint in a wide variety of colubroideans, and we confirmed its presence in all homalopsids and the majority of elapoids and colubroids examined here (Figs F–AA in S1 Appendix). A few notable exceptions are the cyclocorid *Oxyrhabdium*, the atractaspidids *Macrelaps*, *Aparallactus* and *Atractaspis*, the lamprophiids *Chamaelycus* and *Lycophidion*, the natricids *Atretium* and *Aspidura*, and the calamariids *Macrocalamus* and *Oreocalamus* (S1 Appendix). Both accounts suggest that an expanded prokynetic joint is a synapomorphy that evolved in the most recent common ancestor of homalopsids, elapoids, and colubroids, and have been lost or reduced in a few unrelated lineages, like atractaspidids and calamariids among others [30]. Although an incipient joint occurs in some “basal” viperids, such as *Causus* and *Atheris*, it appears that viperids never evolved the complex prokynetic joint of homalopsids, colubroids, and elapoids, but retained a septomaxillary-frontal contact [30].

Both higher endoglyptodont superfamilies Elapoidea and Colubroidea are retrieved with robust support values in all recent FTMG and MTFG molecular analyses, including our study [19,23,26,28,136,137]. Despite such consensus from independent molecular sources, an important conclusion that emerges is that higher-level phylogenetic affinities between the main families of elapoids and colubroids are far from resolved. Virtually no significant support values exist for any of the hypothesized higher-level affinities within these two derived endoglyptodont superfamilies, except for a moderately supported clade formed by the families Sibynophiidae, Calamariidae, Grayiidae, and Colubridae (Fig 1A). No independent morphological evidence is known to support this clade.

#### Endoglyptodonts of uncertain affinities

In addition to the unstable familial affinities within colubroids and elapoids, a number of poorly-known genera are persistently recovered outside the main recognized families, in statistical unsupported phylogenetic positions, further hidering any attempt to provide a stable phylogenetic hypothesis for endoglyptodonts.

Recently, Weinell and Brown [36] resolved a long-standing debate regarding the phylogenetic affinities of poorly known *Cyclocorus*, *Oxyrhabdium*, *Hologerrhum*, and *Myersophis*. *Oxyrhabdium* was first sequenced by Lawson et al. [18], and its affinities remained uncertain [22,23,26]. *Cyclocorus* was independently sequenced by us (this study) and Weinell and Brown [36]. However, our limited sampling resolves *Cyclocorus* and *Oxyrhabdium* as two unrelated elapoid lineages with uncertain affinities, while the molecular analysis of Weinell and Brown [36] retrieved them along with *Hologerrhum* and *Myersophis* in a well-supported, entirely endemic elapoid clade of Philippine snakes. Weinell and Brown [36] accommodated these four genera in their newly erected subfamily Cyclocorinae (here viewed as family Cyclocoridae, new combination), resolving the taxonomic status of our two apparently unrelated rogue taxa *Cyclocorus* and *Oxyrhabdium*. Although monophyly of cyclocorids seems to be now well established, their sister-group relationship within Elapoidea remains unresolved and in need of further investigation [36].

Despite recent contributions, *Buhoma*, *Micrelaps*, *Psammodynastes*, and *Oreocalamus* remain as rogue taxa in all recent molecular analyses (Figs 1–3). In addition to these sequenced genera with uncertain affinities, several other genera that have not been sequenced yet have been treated recently as *incertae sedis* [22,23,26], including *Blythia*, *Elapoidis*, *Gongylosoma*, *Helophis*, *Iguanognathus*, *Montaspis*, *Poecilopholis*, *Rhabdops*, and *Tetralepis*. Among these, *Poecilopholis*, *Iguanognathus*, and *Tetralepis* remain known from one or a few individuals only.

Nagy et al. [156] considered African *Helophis* to be almost indistinguishable from *Hydraethiops* and, thus, allocated it to the family Natricidae. We concur with and follow these authors. Similarly, Asiatic *Blythia* and *Rhabdops* seem to belong to Natricidae since they share with other members of the family the presence of hypapophyses throughout the precloacal vertebrae and hemipenes with an undivided sulcus spermaticus and spinose throughout, with minute spinules disposed distally, becoming gradually larger towards the base of the organ [157]. South African endemic *Montaspis* was thought to belong to the Pseudoxyrhophiidae [22,158,159]. However, contrary to Lambiris [159] who found no close morphological affinities between the hemipenial morphology of *M*. *gilvomaculata* and other southern African “colubrids”, we find that the hemipenis of *Montaspis* closely resembles the pattern of most lamprophiids, and specifically those of *Lamprophis* and *Chamaelycus* (Fig K in S3 Appendix). These taxa share organs uniformly covered by large spines arranged in more or less transverse rows and covered on their proximal third by an enlarged sheath of tissue that forms a fringed pattern around the lobes and body. The hemipenis of *Monstapis* also shares with other lamprophiids a deeply divided centrolineal sulcus spermaticus that bifurcates on the proximal half of the hemipenial body. Differently from the condition described in *Montaspis* and lamprophiids, the hemipenes of pseudoxyrhophiids lack fringes associated with the larger body spines and, with few exceptions (e.g., *Pseudoxyrhopus tritaeniatus* and *Duberria lutrix*), have lobes ornamented by distinct diminute spinules that are often densely packed (Figs A-N in S3 Appendix) [29]. Thus, we consider *Montaspis* to belong to the elapoid continental African radiation of Lamprophiidae rather than being more closely related to the Malagasy pseudoxyrhophiid radiation.

Among the remaining genera of unclear affinities listed above, we added previously unavailable sequences of *Oreocalamus* to our analysis, and retrieved it nested inside the clade Colubroidea (see Discussion below). We also analyzed hemipenial and cranial materials of *Gongylosoma*, *Elapoidis*, *Cyclocorus*, *Oxyrhabdium*, *Oreocalamus*, *Iguanognathus*, and the recently described genus *Colubroelaps*, which appears to be incorrectly allocated to the family Colubridae [33,34]. The morphological data retrieved from these genera provides an opportunity to reevaluate their phylogenetic affinities and taxonomic status within Colubroides.

***Gongylosoma***—The genus includes five poorly known and rare species that occur throughout Southeast Asia and the Greater Sunda Islands [33,34]. Species of that genus were frequently considered to belong to the colubrid genera *Ablabes* [160] or *Liopeltis* [157], until Leviton pointed out important morphological differences within the genus, and transferred three species to *Gongylosoma* (*G*. *baliodeira*, *G*. *longicaudum*, *G*. *scriptum*). Both *Gongylosoma* and *Liopeltis* have been referred to the family Colubridae recently (ex Colubrinae) without further clarification [34]. Our analysis of the hemipenes of *Gongylosoma balioderum* and *Liopeltis frenatus* (Fig AC in S3 Appendix) confirms this allocation, and further corroborates the observations made by Smith [157] and Leviton [161] on their hemipenial distinctiveness. Both hemipenes retain a typically colubrid condition, with a simple sulcus spermaticus that opens in the distal tip of a single lobe, the hemipenial lobe of *L*. *frenatus* being ornamented by large, uniformly distributed, papillate calyces while the lobe of *G*. *balioderum* is covered by large papillae without calyces. The proximal half of the organ is ornamented by large and medium-sized spines in *L*. *frenatus*, and sparcely distributed spinules in *G*. *balioderum*. The latter species also bears deep, conspicuous folds that border the sulcus and extend to the asulcate surface, forming an infolding around the proximal surface of the body. Smith [157] described a very similar condition on a dissected hemipenis of *G*. *scriptum*.

***Elapoidis***—Except for Wallach et al. [34], who assigned the genus *Elapoidis* Boie, 1827 to the Natricidae without any explanation, recent workers have considered it to have uncertain affinities within colubroideans [26,29]. Examination of the hemipenis of one specimen of *Elapoidis fusca* (Fig T in S3 Appendix) corroborates the allocation of the genus to the Natricidae. The hemipenis of *Elapoidis* and natricids share the derived condition of a highly centripetal sulcus spermaticus that opens on the apical surface of the lobular crotch, with the divergent branches forming a nude area that expands on the apical surface of two reduced lobes. The hemipenis is also ornamented only with small to medium-sized spines distributed throughout the organ, as occurs in all natricids, and with a unique large-sized spine disposed at the base of the hemipenial body, like in most natricids. Apart form the nude apical area, the lobes are ornamented by uniformly distributed minute spines. Additionally, the genus *Elapoidis* retains well-developed hypapophyses throughout the posterior trunk vertebrae [162]. The combination of these characters is only known to occur in natricid snakes.

***Cyclocorus* and *Oxyrhabdium***—Although Weinell and Brown [36] clarified the phylogenetic affinities of these two genera, there is virtually no information on their cranial, vertebral and hemipenial morphology. Here, we provide information on the skull and hemipenis of both genera, and the vertebrae of *Cyclocorus*. Our examination of the hemipenes reveals that both conform to the elapoid morphology (i.e., ornamented only with spinules and spines). However, they are strikingly distinct from each other and do not offer a clue about their close affinities. *Cyclocorus* has an extremely long and narrow, unilobed hemipenis that lacks any specialized ornamentation, being sparcely covered by spinules and retaining a single sulcus spermaticus. In contrast, *Oxyrhabdium modestum* has deeply bilobed hemipenes, with a centrolineal sulcus spermaticus, and is covered by uniform and sparcely distributed small spines. *Cyclocorus lineatus* has a typical colubriformes precloacal vertebral morphology, with well-developed hypapophyses, laterally tapering prezygapophyseal processes, a low uniformly thin, blade-like neural spine that reaches the zygosphenal tectum, distinct para- and diapophyseal articular facets projecting ventro-laterally, round condyle, and well-defined cotylar ventrolateral processes. The presence of expanded blade-like hypapophyses conforms with the generalized elapoid morphotype. Vertebrae of *Oxyrhabdium modestum* were not available for study.

***Oreocalamus***—We provide the first assessment of the skull and hemipenis of this genus. *Oreocalamus* has a peculiar hemipenis that ressembles the organ of *Calamaria* in its overall morphology, with a pair of short, but deeply bilobed and rounded lobes ornamented by symmetrically disposed calyces, centrifugal sulcus spermaticus, and spineless (mostly nude) hemipenial body. However, it differs from *Calamaria* by the presence of a large pair of pockets on the asulcate surface of the hemipenial body and deeply defined capitular grooves at the base of the lobes (lobes are bicapitulated). Both *Oreocalamus* and *Calamaria* have typically colubroid vertebral morphologies with an elongate centrum that lacks hypapophyses (Fig G in S2 Appendix). Finally, *Oreocalamus* and *Macrocalamus* appear to have lost secondarily the septomaxillary-frontal articulation while *Calamaria* retains a reduced articulation (Fig B in S1 Appendix). Further, *Macrocalamus* and *Oreocalamus* have a similar prefrontal morphology, with an unusually expanded lateral process projecting from the dorsal half of the prefrontal and tapering anteriorly (Fig X in S1 Appendix). Although *Oreocalamus hanitschi* was retrieved nested inside Colubridae [17], a group with exclusively unilobed hemipenes, its phylogenetic position is not statistically supported in our molecular tree, and it might be equally possible for this species to cluster with Calamariidae since none of the branches that separate them received significant combined SHL/BH support values (Fig 19). Therefore, in face of the hemipenial and osteological similarities shared between *Oreocalamus*, *Calamaria*, and *Macrocalamus*, we assign it to the Calamariidae instead of the Colubridae.

***Colubroelaps***—The monotypic genus *Colubroelaps* was described by Orlov et al. [163] to accomodate a small fossorial snake from southern Vietnam. They provisionally included the new genus in the family Colubridae (their subfamily Colubrinae) based mainly on the absence of hypapophyses on the posterior trunk vertebrae and of a diastema and sulcate teeth on the posterior end of the maxillae. However, the skull morphology of the type specimen shows that *C*. *nguyenvansangi* has hinged teeth like sibynophiids (Fig W in S1 Appendix). Among colubroidean snakes, *Liophidium* and *Iguanognathus* also retain hinged teeth [25], but differently from the latter two genera, *Colubroelaps* shares with sibynophiids the presence of a distally broadened, plate-like maxillary process of the palatine, absence of a choanal process of the palatine, a long tubular dorsally-curved compound bone, reduced mandibular fossa, vestigial splenial and angular bones, and a posterior dentigerous process of the dentary separated from the compound bone and forming a projected free ending process that diverges from the main mandibular axis (Figs V and W in S1 Appendix). Also, like *Sibynophis* and *Scaphiodontophis*, the maxilla of *Colubroelaps* projects freely posteriorly to the maxillary-ectopterygoid contact, but without forming an elongated “dentigerous process” [25]. The combination of these derived characters shared by *Sibynophis*, *Scaphiodontophis*, and *Colubroelaps* supports the inclusion of the latter genus in the family Sibynophiidae. The absence of hypapophyses on the posterior trunk vertebrae of *C*. *nguyenvansangi* seems to contradict the present allocation because sibynophiids retain well-developed hypapophyses throuhought the trunk vertebrae (Fig G in S2 Appendix). However, we suspect that posterior hypapophyses are reduced due to the fossorial habits of that species.

***Iguanognathus***—The genus *Iguanognathus* was tentatively allocated in the families Colubridae and Natricidae in the past, despite the lack of any compelling evidence supporting either hypotheses. Like sibynophiids, *Iguanognathus* has hinged teeth [164], which would suggest a possible close relationship with that family. However, apart from the presence of hinged teeth, *Iguanognathus* does not share any of the other cranial specializations typical of sibynophiids [25], as discussed above. Instead, *Iguanognathus* retains an aligned posterior dentigerous process of the dentary, well developed, functional splenial and angular bones, a choanal process, and a posteriorly curved tapering maxillary process of the palatine. Additionally, unlike sibynophiids, *Iguanognathus* lacks hypapophyses on the posterior precloacal vertebrae [165] which also rules out its belonging in the Natricidae. For these reasons, we prefer to consider this genus a Colubridae *incertae sedis*.

### A time-calibrated tree and the fossil record of Colubroides

#### Previous age estimates of Colubroides

Divergence time estimates have been increasingly discussed in molecular studies of snake evolution in recent years [26,61,69,58,132,133,166]. Although they have been used to detail the tempo and mode of evolution of the group, these studies have sometimes inferred substantially different dates for major events in colubroid diversification. As Fig 23 shows, nine recent studies provide disparate dates for the origin of most higher-level clades of colubroideans [22,58,124, 132–135,166]. Our divergence time estimates are mostly concordant with those of Burbrink and Pyron [132] but are significantly younger than the dates estimated by some other studies [26,58]. Although there are differences in which fossils were used for calibrations, and in the numbers of genes and taxa, the different results in Fig 23 mostly reflect expected differences between analyses based on autocorrelated and uncorrelated molecular clocks [167]. Studies in which time estimations were generated by penalized likelihood algorithms (e.g. treePL) tend to keep the same general pattern and same temporal cladogenic order. Comparing only the studies using autocorrelated methods [26,22,132] and our own (Fig 23), we observe that a difference among time estimations for one specific clade implies differences in the entire cladogenic process, that can be younger or older as a whole, but can never be younger for some clades and at the same time older for others, or vice-versa. In contrast, time estimations generated by uncorrelated methods [58,124,133,134] and a Bayesian autocorrelation method [135] tend to be more variable with respect to the general pattern, presenting cladogenic events that are ordered different among each study. As an example, a previous analysis of the family Viperidae using an uncorrelated method [124] in BEAST resulted in much older cladogenic events for the family as a whole (Fig 23). These differences likely result from the different methods of divergence time estimation used by Alencar et al. [124] and the present study.

Such disparate results suggest that inferred dates of divergence should be treated with caution, and that the available fossil evidence is paramount to an accurate description of the evolutionary trends of a group. Therefore, we integrate our estimated divergence dates with the fossil record in an attempt to reach more balanced conclusions regarding the evolutionary events underlying the origin and diversification of extant colubroidean families.

Despite their differences, some general trends emerge from the eight studies illustrated in Fig 23. Nine of the ten studies estimated an early divergence time for the ancestor of Colubroideans (i.e., the split between Colubroidea and Elapoidea) which, together, span an interval of approximately 35 My, from the upper Cretaceous (Turonian) to the upper Paleocene (Thanetian). Six of these studies place the origin of the group within the Cretaceous while three retrieve a Paleocene origin. The former hypothesis of a Cretaceous origin of the group is concordant with the presence of alleged colubroidean vertebral remains in the Cenomanian of the Wadi Milk Formation of Sudan [168]. However, the more complete material described by Rage and Werner [168] belongs to the enigmatic Caenophidian family Russellophiidae, a group known only from vertebral remains and only tentatively assigned to the clade Colubroides. The other colubroidean vertebrae were considered of indeterminate Colubroidean affinities due to their fragmentary condition [168], lacking preserved parts with unambiguous derived colubroidean traits and rendering their assignment to this group questionable [169]. Although a late Cretaceous origin of the group seems likely, more definitive evidence of Cretaceous colubroidean records is lacking.

#### Colubroidean early divergence

The colubroidean fossil record is mostly composed of disarticulated vertebrae that are difficult to assign to any extant family because vertebral characters alone are of limited value when it comes to diagnosing most colubroidean clades [31,113]. Despite this limitation, colubroidean precloacal vertebrae were recognized until recently by the following suite of derived characters, known to occur in combination only in colubroidean snakes [31,68,109,169]: an elongate centrum, well-developed prezygapophyseal accessory processes, a blade-like neural spine that extends anteriorly onto the zygosphene and remains uniformly thin anteroposteriorly (as opposed to an expanded posterior margin of the neural spine), well-developed subcotylar tubercles (or cotylar ventrolateral processes), distinct dia- and parapophyseal articular facets of the synapophysis, prominent hypapophyses on middle and posterior trunk vertebrae, and paracotylar foramina.

Among these characters, the uniformly thin blade-like neural spine that extends onto the roof of the zygosphene appears to be invariably present in all colubroideans, and unique to the group. However, our observations reveal that extant xenodermids lack an uniformly blade-like neural spine that reaches the roof ot the zygosphene (Fig 25; Fig A in S2 Appendix) [30,31]. Although *Achalinus*, *Xenodermus*, and *Fimbrios* tend to retain a blade-like posterior margin, the neural spine never invades the roof of the zygosphene anteriorly [30,31] (Fig 25; Fig A in S2 Appendix). Therefore, we consider this character to represent a putative synapomorphy of the clade Colubriformes, with important implications in the definition of the minimum age used as calibration point for the base of our estimated colubroidean divergence time tree.

Among extinct putative caenophidian families Russellophiidae, Nigerophiidae, and Anomalophiidae, only the latter seems to retain a similar neural spine morphology [31], and might well represent an early colubriform lineage. However, the enigmatic nature of these three families, known only from sparce vertebral material, precludes any unambiguous allocation to the colubroidean radiation.

Therefore, the earliest records of allegedly uncontested colubroidean vertebrae from the Lower to Upper Eocene [68,109,169–175] are more accurately assigned to the clade Colubriformes instead.

Our time calibrated tree places the divergence of stem-colubroideans at ~ 56 Mya, near the Paleocene/Eocene boundary, while stem-colubriforms diverged at ~ 53 Mya within the Ypresian (Fig 22). Early fossil records of definitive colubriforms are concordant with this date, with the oldest unequivocal record being of *Procerophis sahnii* [68] from the early Ypresian of India, with an age of 54 Mya. (Fig 22) [68,71,72]. The other known Eocene colubriform records are all from the middle/upper Eocene: an unnamed colubriform from the middle Eocene of Namibia (41.2 Mya) [174]; an unnamed colubriform from the middle Eocene of Myanmar (37.2 Mya) [169]; *Renenutet enmerwer* from the middle Eocene of Egypt (37 Mya) [172]; a vertebra referred to *Nebraskophis* from the upper Eocene of Hardie Mine, USA (34.2 Mya) [171]; and an unnamed colubriform from the upper Eocene of Thailand (34 Mya) [175]. Because the vertebrae of *Vectophis wardi* and *Headonophis harrisoni* from the upper Eocene of the Isle of Wight, England (33.9 Mya) [170,173] retain a robust and posteriorly expanded neural spine that does not invade the zygosphenal roof anteriorly, we treated them as caenophidians of uncertain affinities instead of belonging to the clades Colubroides or Colubriformes.

Molecular evidence supports an early Paleogene divergence of colubroideans in Asia [132,134], but they may have been present already in Africa in the early Upper Cretaceous [168]. The presence of a definitive colubriform snake in the Lower Eocene of Namibia [174], and the recent finding of *Renenutet enmerwer* in the Upper Eocene of Egypt [172] along with an already well established colubroidean fauna in the Lower Oligocene of Tanzania [94], indicates that the group had already diversified in Africa by the Eocene. Additionally, the presence of diversilly significant number of colubriform records in India during the Eocene, including the oldest undisputed colubriform snake, along with the African records discussed above, suggest that colubroideans may have diversified much earlier in Gondwana prior to its dispersal throughout Laurasia [172]. In that context, the controversial presence of colubroidean snakes in the Cenomanian of Sudan [69], which extends the divergence timing of the group to the early Upper Cretaceous, seems to become a plausible hypothesis [168]. However, additional findings are needed to fill the implied ghost lineage of approximately 40 million years, from the Upper Cretaceous sediments of the Wadi Milk Formation of Sudan to the Lower Eocene undisputed colubriform record of India.

### The Eocene-Oligocene transition and the diversification of present-day Colubroid and Elapoid lineages

The early Oligocene was marked by a much cooler and more temperate global climate than the warm "greenhouse" conditions that characterized most of the Cretaceous and early Cenozoic [176,177]. The impoverished global diversity in the Oligocene that resulted from the Eocene-Oligocene extinctions is also observed in the fossil record of snakes around the world [32]. Our time calibrated tree for colubroideans illustrates this trend, with a relatively low number of cladogenetic events dated prior to the Oligocene-Miocene boundary (Fig 22; S4 Fig). However, these Oligocene cladogenetic events were key for the establishment of the present-day colubroidean snake fauna. While an early divergence of basal colubroidean lineages is estimated to have occurred within the Eocene, all extant families of Colubroidea and Elapoidea that compose most of the present-day endoglyptodont fauna are estimated to have originated rapidly within the early Oligocene interval, between ~ 33 and 28 Mya. (Figs 22 and 23; S4 Fig). Diversification dates retrieved here are consistent with the major terrestrial faunal turnover recorded around the world and associated with the overall climate shift at the Eocene-Oligocene boundary. This trend is consistent with the sudden appearance in the European fossil record of the derived colubrid vertebral morphotype with an elongated centrum, long prezygapophyseal accessory processes, distinct epizygapophyseal spines, and a uniformly narrow haemal keel (lacking hypapophyses), as illustrated by *Coluber cadurci* [31, 69,110,109]. The subsequent, mainly Miocene diversification of extant Colubroidean families, is also highly consistent with the known Neogene colubroidean fossil record [32,178,179].

Among “basal” colubroidean lineages estimated to have diverged within the Eocene (Fig 22), the Xenodermidae, Pareidae, and Homalopsidae still lack a fossil record, while the first unequivocal viperid record is only early Miocene of age (MN1) [79], contrasting significantly with our estimated timescale. Among Paleogene fossil caenophidian snakes, *Thaumastophis missiaeni* approaches the xenodermid vertebral morphology in having vertically oriented blade-like prezygapophyseal accessory processes and a lightly built and elongate vertebral morphology [68]. However, the combination of these two characters, along with the absence of well-developed hypapophyses (present in xenodermids), and the presence of parazygantral foramina (shared with acrochordids) and a blade-like neural spine invading the zygosphenal tectum (shared with colubriformes) precludes its assignment to any of the three colubroidean families cited above, or to the acrochordids [68]. The lack of a well-established fossil record for the Xenodermidae, Pareidae, and Homalopsidae during that interval of early colubroidean evolution hampers any attempt to determine in more details the pattern of early divergence of the group. Notwithstanding, according to our divergence estimates, it can be hypothesized that appearance of grooved venomous teeth and the consequent diversification of higher endoglyptodont lineages occurred within the Eocene, prior to the large-scale faunal turnover that characterizes the Eocene-Oligocene transition [176,177,180–183]. Indeed, our time calibrated tree indicates that stem-Xenodermidae diverged at ~ 52.6 Mya while stem-Pareidae and stem-Endoglyptodonta diverged at ~ 45 Mya. Stem-Viperidae and stem-Homalopsidae also diverged within the Eocene, at ~ 42.5 Mya and 38 Mya, respectively.

#### Viperidae fossil record and divergence time estimates

Although viperids are abundant in the fossil record, most are confined to the Neogene, consist of isolated vertebrae, and are assigned to extant taxa [79,89]. The oldest record of a viperid known so far is *Provipera boettgeri*, described by Kinkelin [184] based on an isolated fang from the early Miocene of Germany (MN1; ~ 21 to 23 Mya) (see Rage [31] for the validity of the name). Viperids are also recorded in the early Miocene of Southern Asia (equivalent to MN 3) [185] and North America (late Arikareean) [186], early or middle Miocene of Central Asia [187], and middle Miocene of northern Africa (equivalent to MN 7+8) [188]. The first unquestionable crotaline was reported by Ivanov [88] from the middle Miocene of Gritsev in Ukraine (MN 9). Szyndlar and Rage [79,85] provided a detailed review of the known Neogene fossil record of the family. As shown by these authors, the fossil record of viperids does not help clarify the early divergence of the family, since most fossils are associated with extant taxa from derived lineages [79,85], as shown in our own phylogenetic tree (Figs 6–9).

Our time calibrated tree suggests that the origin of crown-Viperidae occurred in the early Oligocene, at ~ 30.7 Mya, while the basal split between viperine and causine subfamilies on the one hand, and crotaline and azemiopine subfamilies, on the other hand, occurred within the late Oligocene, at approximately 26 and 25 Mya, respectively (Fig 22). As such, although expected, no fossil viperids have been recorded yet in the Paleogene, resulting in an interval of approximately 20 Mya between their hypothesized early divergence in the Eocene and the first known, early Miocene, unequivocal fossil of the family [79,85]. Additionally, since the sister-group relationship between Asiatic/American crotalines and African/Eurasiatic viperines is mainly symmetric, no conclusion can be reached on the geographic area of origin of the family (apart from excluding the New World).

#### Elapoid fossil record and divergence time estimates

Within Elapoidea, only the Elapidae and, possibly, Pseudoxyrhophiidae have a fossil record [31,32,94]. The oldest known record of an unequivocal elapid comes from the late Oligocene of the Nsungwe Formation, Tanzania, which bears sediments of ~ 25 Mya [94]. McCartney et al. [94] also report a distinct caudal vertebra from the same locality in Tanzania that bears the unusual feature of a single hemal keel instead of paired hemapophyses. According to the authors, the extant genus *Duberria* also exhibits a similar condition, being the only known pseudoxyrhophiid snake so far that lacks hemapophyses but retains a well-developed hemal keel. Although *Duberria*'s caudal morphology ressembles the caudal vertebra described by McCartney et al. [94] as "Colubroid Morphotype C", the authors rightly refrain from assigning the latter to Pseudoxyrhophiidae.

The oldest record of an Australian elapid consists of a vertebra attributed to a hydrophiine found in sediments of Riversleigh dated from the upper Oligocene or lower Miocene (24–23 Mya) [103]. According to Scanlon et al. [103] the vertebra is morphologically very similar to the extant genus *Laticauda*. However, these authors refrain in allocating it to the latter genus given the limited information afforded by one isolated vertebra.

The first record of elapids in Europe comes from the lower Miocene of France (MN 4; [32]). Elapids are further abundantly documented throughout the Miocene and the Pliocene of Europe [115], persisting in that continent until the upper Pliocene when they became extinct [189–191]. According to Szyndlar and Rage [191], most European fossil elapids are assignable to extant *Naja*. This genus is also recorded in the middle Miocene of northern Africa (13.8 Mya; MN70 [188,191]). The relatively abundant skull material found associated with elapid vertebrae in the Neogene of Europe tend to corroborate the view that most of these large Neogene elapids were either closely related to or nested within *Naja* [115,190,192].

Isolated posterior trunk vertebrae from the middle Miocene of North America (upper Barstovian) and Europe (Astaracian, MN7) were assigned to extant *Micrurus* [193], as *Micrurus* sp. and *Micrurus gallicus*, respectively. Referral to the family Elapidae is based on having a low and recurved hypapophysis, a low anteroposteriorly elongated neural spine, and a poorly vaulted neural arch. However, these characters may correlate well with fossorial habits [193], and, thus, their assignment to the *Micrurus* is questionable. No compelling evidence distinguishes the vertebral morphology of *Micrurus* from other Asian and Neotropical coral snake genera. According to Rage and Holman [193], the few vertebrae referred to *Micrurus* from the Miocene of Nebraska (USA) and la Grive (France) are comparable to extant *Micrurus fulvius*, and thus should be referred to this genus. Our observations of the vertebral morphology of South American *Micrurus* shows a very distinctive morphology from that of *Micrurus fulvius*, suggesting that the vertebral morphology of the genus is much more diverse than previously thought. A detailed description of the vertebral morphology of speciose New World *Micrurus* and its closely related North American and Asiatic genera *Micruroides*, *Leptomicrurus*, *Calliophis*, and *Sinomicrurus* is necessary to confidently support the assignment of these Miocene records to any known extant genus, and especially to *Micrurus*.

Sub-Saharan elapids from the end of the Paleogene raise doubts about the well-accepted hypothesis of an Asian origin for the group [2,22,103]. According to McCartney et al. [94], the presence of elapids in the Nsungwe formation indicates two possible scenarios: a rapid initial phase of dispersion of the family from Asia to Africa before the end of the Oligocene or, alternatively, an origin of the family in Africa rather than Asia. The elapids from Nsungwe and the hydrophiine from Riversleigh help reduce the gap between the fossil record and the most recent molecular estimates (Fig 23). The presence of a hydrophiine in the late Oligocene or early Miocene of Australia further supports the hypothesis of a dispersal and colonization of the Australian continent in the late Oligocene [194].

Our time calibrated tree suggests an early divergence of stem-elapids within the early Oligocene at ~ 30.5 Mya, with the main crown-Elapidae lineages diversifying during the late Oligocene at ~ 26.5 Mya (Fig 22; S4 Fig). Although estimates of the time of divergence of Elapidae seem to favour an early Oligocene origin (Fig 22), available molecular phylogenies (including this one) and the fossil record do not yet allow inference of the biogeographic origin of the group. While the discovery of elapids in the upper Oligocene of sub-Saharan Africa and the unambiguous position of the family within the African elapoid radiation favour an African origin, the basal-most positions of successive Asian coral-snake lineages in recent molecular phylogenies tends to favor the opposite hypothesis of an Asian origin of the group. The lack of support for deeper elapid relationship fails to provide a robust support for either hypothesis (Figs 10–12).

Apart from the uncertainties involving the debate on an Asian or African origin, it has been commonly thought that the family dispersed to the West Paleartic, Australian (via Melanesia), and Neartic/Neotropical (via the Bering strait) regions independently [2,32,103,115,183,194]. Scanlon et al. [103] provided robust paleontological evidence supporting the hypothesis of an over-water dispersal to Australia of the ancestor of the hydrophiine radiation, close to the Oligocene–Miocene boundary. The dispersal into the West Paleartic in the lower Miocene of forms belonging to or closely related to the extant genus *Naja* is also well documented [2,32,115,190–192]. In contrast, the occurrence of extant genus *Micrurus* in the Miocene of France is questionable due to the absence of vertebral diagnostic features that distinguish members of this genus from the Asiatic coral snake radiation. Its record in the Miocene of North America also needs further corroboration since Holman [195] used only two extant species of *Micrurus* (*M*. *fulvius* and *M*. *affinis*) and *Micruroides euryxanthus* for comparison.

Our time calibrated tree places the early divergence of stem-hydrophiines at ~ 23 Mya, at the Oligocene–Miocene boundary, and the ancestor of the New World radiation of coral snakes (stem-micrurines) diverged from the Old World coral snakes at ~ 21 Mya in the early Miocene. These results support the hypotheses of a late Oligocene over-sea dispersal and colonization of the Australian continent by the ancestor of hydrophiines [103] and early Miocene terrestrial colonization of North America by the ancestor of New World coral snakes.

#### Colubroid fossil record and divergence time estimates

The fossil record of Colubroidea is much more extensive than that of elapoids but is mostly confined to the Neogene. Their vertebral morphology remains poorly known, and an accurate evaluation of the fossils assigned to this superfamily or to specific colubroid families remains far from being resolved. Pseudoxenodontids, calamariids, sibynophiids, and grayiids have not been recorded so far in the fossil record. In contrast, “colubrid” and natricid fossils are abundant and have been recorded throughout the Neogene of North America and Europe [31,175,179]. Colubrids and dipsadids are also well-represented in the Neogene of North America [178].

Although Miocene and Pliocene records of colubroids are straightforward, Paleogene records are more elusive and most of them are of uncertain assignment. Here we follow Smith [109] in assigning the vertebrae from the upper Eocene of the Medicine Pole Hills of the Chadron Formation in North Dakota to Colubroidea since they retain a “racer-like” vertebral morphology consistent with those of the North American racer clade of colubrids [109]. This record represents the oldest known Colubroidea so far.

The divergence between Colubroidea and Elapoidea in our time calibrated tree is estimated at ~ 36 Mya, lying close to the boundary between the Eocene and Oligocene (Fig 22) and in accordance with the appearance of colubroids in the late Eocene of North America (Chadronian NALMA) [109]. Similar to elapoid families in our time calibrated tree, ancestors of extant families of colubroids diverged during the early Oligocene, between ~ 30 to 33 Mya. These dates are also in agreement with the first emergence of the typical colubrid vertebral morphotype in the early Oligocene of France, documented by *Coluber cadurci* from the Phosphorites of Quercy (Mammal Paleogene Reference Unit MP 22). Colubrid precloacal vertebrae can be distinguished from all other colubroidean family by the presence of the following combination of derived features: an elongated centrum, long prezygapophyseal accessory processes, distinct epizygapophyseal spines, and an uniformly narrow haemal keel (lacking hypapophyses). Slightly younger records of putative natricids from the Phosphorites of Quercy are difficult to allocate due to their fragmentary condition and their overall similarities with the vertebral morphology of several extant elapoid families. Holman [178] and Szyndlar [179] detailed the Neogene colubroid records in North America and Europe, respectively.

## Conclusions

The traditional meaning of the superfamily Colubroidea [18,26,26,28] no longer accommodates our growing knowledge of the phylogenetic affinities [18,19,23,25,26,27,28] and morphological disversity and disparities in the group [8,11,23,25,29,30]. Despite some pleas against any change on the traditional usage of the names “Colubroidea” and “Colubridae” [196], the new morphological evidence provided here reinforces the need for a series of taxonomic changes to accommodate new phylogenetic and morphological knowledge. Xenodermids, pareids, and xylophiids represent ancient caenophidian lineages that are phylogenetically and morphologically distinct from the endoglyptodont radiation. All three lineages lack the dental specializations that gave rise to an advanced venom delivery system characteristic of endoglyptodonts, thus breaking a universally accepted definition of colubroids as representing the truly venomous snake radiation. Xenodermids further lack some vertebral and cranial features that are commonly used to determine colubroid fossil remains and share with acrochordoids cranial and vertebral specializations that are virtually absent in the remaining caenophidian lineages.

Our observations on caenophidian vertebral morphology, especially in xenodermids, were particularly useful in redefining the fossil record commonly used as calibration points in molecular phylogenies (e.g., the oldest colubroidean remains–*Procerophis sahni*–is here placed as a calibration point for the early divergence between colubriformes and xenodermids instead of the traditional divergence between xenodermids and acrochordids). By reviewing and reinterpreting the relevant fossil record, our treePL analysis was able to highlight previously unnoticed correlation between the early diversification of colubroid and elapoid major lineages and the Eocene-Oligocene transition. Our divergence dates are in general younger than most previous studies (Fig 23) and, although many authors would suspect that disparate dates result from distinct methodological approaches, we suggest that most of these differences are due primarily to the effect of highly heterogeneous usages of the fossil record rather than of distinct methodological procedures. More importantly, our results highlight the need for more detailed anatomical studies in combination with a more careful usage of the fossil record [197].

Similarly, comparative morpho-functional or behavioural studies with caenophidians, that are dependent on previously published, molecular phylogenies as frameworks, should seek more accurate, combined evidence of branch support to evaluate their evolutionary scenarios. Our statistical exploration of three different support methods indicates that TBE and SHL are constantly higher and less conservative than FBP values. Based on the discrepancies among these methods, we reinforce the combined use of different support values to identify nodes that are not well supported or ambiguously supported. Such approach can help highlight weakly supported clades and/or the presence of rogue terminals in phylogenetic datasets. Our study revealed that a large number of colubroidean clades are still either poorly or ambiguously supported and should be treated with caution.

## Supporting information

S1 Table Distinct classification schemes discussed in this study. Number of Phylogenetic levels in each classification scheme are as follow: 1–3 = higher-levels, 4 = superfamily, 5 = family, 6 = subfamily; families are listed in bold.
(XLSX)

S2 Table Categories of combined clade support. Graphic illustration for combined clade support values when comparing FBP, SHL, and TBE metrics, classified in seven categories, as follows: 1) Red, unambiguously supported; 2) Orange, robustly supported; 3) blue, strongly supported; 4) green, moderately supported; 5) dark grey, ambiguously supported; 6) dark grey, poorly supported; 7) light grey, unsupported (see text for discussion).
(XLSX)

S3 Table List of accession numbers. List of all the terminal taxa and sequences by gene used in the present study, including best partitions, accession numbers of sequences retrieved from GenBank and new sequences produced for this study.
(XLSX)

S4 Table Number and percentage of species and genera sequenced by family. Comparisons between the number of families, genera and species of Colubroides used in the present study and by Figueroa et al. [28] (2016).
(DOCX)

S5 Table Numbers and percentages for genera and species. Number of species by genus of Colubroides sampled in this study; and a list of all species of Colubroides following Uetz et al. [33], indicating which species was sampled in our study (spreadsheet 2).
(XLSX)

S6 Table List of sequences from GenBank considered questionable and/or problematical. List of accession numbers, with genes names, current identification in GenBank, and probable correct identification for questionable and/or problematic sequences of snakes available in GenBank.
(DOC)

S7 Table List of taxa recognized in this study but not listed by Uetz and Hosek (2017). List with rationale for the species recognized in the present study, but not listed in Uetz et al. [33].
(DOCX)

S8 Table List of Primers used in this study. List with sequences for the pairs of primers used to amplify the gene fragments used in the present study.
(XLSX)

S9 Table Numbers of clades in each category of combined FBP, TBE, and SHL support values. Clade support values based on the combination of (A) FBP/SHL, (B) FBP/TBE, (C) and TBE/SHL, classified in seven categories, as follows: red, unambiguous support (both methods recover values of 100%); orange, robust support (both methods recover values ≥ 90%, or ≥ 80% in one method and 100% in the other); blue, strong support (both methods recover values ≥ 80%, or values ≥ 70% in one method and ≥ 90% in the other); green, moderate support (both methods recover values ≥ 70% but do not reach values equal to previous categories); dark grey, ambiguous support (highly discrepant values, with < 70% in one method and ≥ 80% in the other) or poor support (values < 70% in one method and between 70% and 80% in the other method); light grey, unsupported (values < 70% for both methods).
(XLSX)

S10 Table Representative fossil snakes from the Cenozoic. List of fossil snakes from the Paleogene and Neogene with authorship, stratigraphic occurrence and locality.
(XLSX)

S1 Fig Full RAxML tree. Maximum likelihood tree of Colubroides containing 1263 terminals. Color of the squares follow the categories of combined clade support as described in S2 Table. Numbers inside de squares on the nodes of the full tree represent the bootstrap and SHL values retrieved. Diamonds on each tip represent the percentage of the data for each terminal generated in this study: white, 0%; light gray, between 1% and 50%; dark gray, between 50% and 99%; black, 100%. Terminals in red represent additional samples in relation to Pyron et al. [26].
(PDF)

S2 Fig Full treePL tree. Data matrix and calibrated tree resulting from the treePL analysis of Colubroides, including the outgroups and containing 1278 terminals (1263 Colubroides and 15 outgroups).
(TRE)

S3 Fig Full RAxML tree. Maximum likelihood species-level phylogeny of Colubroides including comparisons among values of FBP, SHL, and TBE support metrics. Numbers inside de squares on the nodes of the full tree represent the TBE values retrieved.
(PDF)

S4 Fig treePL zoomed trees. Zoomed, large-scale calibrated tree resulting from the treePL analysis showing the pattern of cladogenic events through time.
(PDF)

S1 Appendix Skulls. Skull morphology of representatives of colubroidean families illustrating the naso-frontal joint and optic foramen/fenestra. Figure A, Tropidophiidae: *Tropidophis nigriventris* (AMNH 81182); Acrochordidae: *Acrochordus granulatus* (ZMB 9444). Figure B, Xenodermidae: *Achalinus spinalis* (AMNH 34621), *Fimbrios klossi* (BMNH 1946.1.15.88). Figure C, Xenodermidae: *Xenodermus javanicus* (FMNH 158613); Xylophiidae: *Xylophis perroteti* (BMNH 1955.1.3.10). Figure D, Pareidae: *Pareas moellendorffi* (AMNH 27770), *Apopeltura boa* (BMNH 47.12.30). Figure E, Viperidae: *Azemiops kharini* (ZMB 69985), *Bothrops neuwiedi* (MZUSP 1476), *Causus rhombeatus* (FMNH 74241), *Vipera ursinii* (MZUSP 8230). Figure F, Homalopsidae: *Bitia hydroides* (FMNH 229568), *Brachyorrhos albus* (FMNH 142322), *Enhydris chinensis* (AMNH 33870), *Fordonia leucobalia* (AMNH 107179). Figure G, Homalopsidae: *Homalopsis buccata* (MNHN 1963.728); Psammophiidae: *Malpolon monspessulanus* (AMNH 140768), *Mimophis mahfalensis* (UMMZ 209653). Figure H, Psammophiidae: *Psammophylax variabilis* (AMNH 73213), *Rhamphiophis oxyrhynchus* (AMNH 16890), *Psammophis phillipsi* (AMNH 67750). Figure I, Cyclocoridae: *Cyclocorus lineatus* (MNHN 1900.413), *Oxyrhabdium modestus* (FMNH 53386); Atractaspididae: *Aparallactus modestus* (AMNH 50545). Figure J, Atractaspididae: *Atractaspis bibronii* (AMNH 82073), *Homoroselaps lacteus* (LSUMZ 57229), *Macrelaps microlepidotus* (FMNH 205860), *Polemon christyi* (FMNH 219913). Figure K, Lamprophiidae: *Bothrolycus ater* (AMNH 11976), *Chamaelycus fasciatus* (BMNH 1909.4.29.3), *Dipsadoboa weileri* (AMNH 12472), *Lamprophis olivaceus* (AMNH 12003). Figure L, Lamprophiidae: *Lycodonomorphus rufulus* (AMNH 140284), *Lycophidion capense* (AMNH 63771), *Gonionotophis capensis* (AMNH 73208), *Pseudoboodon lemniscatus* (MNHN 1905.179). Figure M, Pseudoxyrhophiidae: *Alluaudina bellyi* (UMMZ 201605), *Dromicodryas quadrilineatus* (UMMZ 209290), *Duberria lutrix* (UMMZ 154361), *Heteroliodon occipitalis* (UMMZ 218178). Figure N, Pseudoxyrhophiidae: *Ithycyphus miniatus* (UMMZ 201615), *Langaha madagascariensis* (UMMZ 218193), *Liophidium torquatum* (UMMZ 209437), *Pseudoxyrhopus tritaeniatus* (UMMZ 203649). Figure O, Elapidae: *Bungarus caeruleus* (AMNH 87483); *Calliophis intestinalis* (BMNH 1880.9.10.15), *Micrurus narduccii* (MZUSP 8370), *Naja naja* (AMNH 86912). Figure P, Elapidae: *Notechis scutatus* (ZMB 7930), *Toxicocalamus loriae* (AMNH 95581); Pseudoxenodontidae: *Pseudoxenodon stricticaudatus* (AMNH 34674). Figure Q, Natricidae: *Afronatrix anoscopa* (MNHN 1986.1618), *Aspidura trachyprocta* (AMNH 120251), *Atretium schistosum* (AMNH 85509), *Lycognathophis seychellensis* (UMMZ 195836). Figure R, Natricidae: *Natriciteres fuliginoides* (MNHN 1987.1552), *Natrix maura* (AMNH 115697), *Sinonatrix annularis* (AMNH 115693), *Xenochrophis cerogaster* (AMNH 89276). Figure S, Dipsadidae: *Apostolepis* cf. *nelsonjorgei* (MZUSP 20636), *Atractus maculatus* (IB 40003), *Conophis pulcher* (AMNH 117934), *Contia tenuis* (UMMZ 133519–1). Figure T, Dipsadidae: *Farancia abacura* (KU 214419), *Geophis hoffmanni* (AMNH 113561), *Helicops pastazae* (AMNH 49143), *Heterodon nasicus* (MNHN 1993.1625). Figure U, Dipsadidae: *Philodryas mattogrossensis* (AMNH 141377), *Sibon sartorii* (LSUMZ 23243), *Tachymenis peruviana* (KU 135193), *Urotheca multilineata* (AMNH 98284). Figure V, Dipsadidae: *Xenopholis scalaris* (AMNH 60799); Sibynophiidae: *Scaphiodontophis annulatus* (MZUSP 5971), *Sibynophis subpunctatus* (AMNH 96073). Figure W, Sibynophiidae: *Colubroelaps nguyenvansangi* (ZISP/IEBR 25682). Figure X, Calamariidae: *Calamaria gervaisi* (AMNH 36744), *Macrocalamus lateralis* (LSUMZ 45407); *Oreocalamus hanitschi* (BMNH 1929.12.22.106). Figure Y, Grayiidae: *Grayia smithii* (AMNH 140428); Colubridae: *Boiga dendrophila* (AMNH 116014), *Coluber constrictor* (FMNH 135284). Figure Z, Colubridae: *Dendrelaphis papuensis* (AMNH 107175), *Ptyas mucosus* (AMNH 83993), *Scaphiophis albopunctatus* (AMNH 104101), *Senticolis triaspis* (AMNH 110625). Figure AA, Colubridae: *Spilotes pullatus* (IBSP 4955); Colubridae incertae sedis: *Iguanognathus werneri* (BMNH 1946.1.6.34). Figure AB, Elapoidea incertae sedis: *Buhoma depressiceps* (BMNH 1907.5.22.10), *Micrelaps muelleri* (HUJR 8009). Scale bar = 1 mm.
(PDF)

S2 Appendix Vertebrae. Posterior trunk vertebral morphology of representatives of colubroidean families. Figure A, Acrochordidae: *Acrochordus javanicus* (USNM 297404), scale bar = 2 mm; Xenodermidae: *Achalinus rufescens* (BMNH 1946.1.12.37), scale bar = 1 mm; *Fimbrios klossi* (BMNH 1946.1.15.88), scale bar = 1 mm; Pareidae: *Pareas* sp. (MZUSP 12186), scale bar = 1 mm. Figure B, Viperidae: *Causus difilippi* (MZUSP 18668), scale bar = 5 mm; *Vipera ursinii* (MZUSP 8230), scale bar = 5 mm; *Azemiops feae* (ROM 36976), scale bar = 1mm; *Bothrops jararaca* (MZUSP 14425), scale bar = 2mm. Figure C, Homalopsidae: *Cerberus rynchops* (MZUSP 9569), scale bar = 2mm; *Homalopsis buccata* (MZUSP 11483), scale bar = 1mm. Psammophiidae: *Psammophis lineolatus* (MZUSP 8221), scale bar = 1mm; *Mimophis mahfalensis* (MZSUP 12188), scale bar = 2mm. Figure D, Pseudoxyrhophiidae: *Madagascarophis colubrinus* (BMNH 89.8.28.23), scale bar = 2mm; *Ditypophis vivax* (BMNH_99.12.5.125), scale bar = 1mm; Lamprophiidae: *Boaedon fuliginosus* (MZUSP 8167), scale bar = 2mm; *Crotaphopeltis hotamboeia* (MZUSP 19602), scale bar = 1mm. Figure E, Atractaspididae: *Atractaspis irregulares* (MZUSP 10826), scale bar = 1mm; *Homoroselaps lacteus* (LSUMZ 57229), scale bar = 1mm. Elapidae: *Sinomicrurus macclellandi* (ROM 37113), scale bar = 1mm; *Naja naja* (UMMZ 181137), scale bar = 1mm. Figure F, Elapidae: *Micrurus corallinus* (MZUSP 13112), scale bar = 1mm; Cyclocoridae: *Cyclocorus lineatus* (BMNH 96.3.30.78), scale bar = 1mm; Natricidae: *Natrix natrix* (MZUSP 2514), scale bar = 2mm; *Natriciteres olivacea* (MZUSP 2083), scale bar = 1mm. Figure G, Sibynophiidae: *Scaphiodontophis annulatus* (MZUSP 5971), scale bar = 2mm; Grayiidae: *Grayia smithii* (MZUSP 8130), scale bar = 1mm; *Grayia tholloni* (MZUSP 8135), scale bar = 2mm; Calamariidae: *Oreocalamus hanitschi* (BMNH 1929.12.22.106), scale bar = 1mm. Figure H, Colubridae: *Chironius bicarinatus* (MZUSP 13860), scale bar = 2mm; *Spilotes pullatus* (MZUSP 13845), scale bar = 2mm; *Oxybelis aeneus* (MZUSP 13028), scale bar = 2mm; *Mastigodryas boddaerti* (MZUSP 13052), scale bar = 2mm. Figure I, Colubridae: *Simophis rhinostoma* (MZUSP 13858), scale bar = 2mm; Dipsadidae: *Heterodon platirhinos* (MZUSP 2991), scale bar = 2mm; *Farancia abacura* (MZUSP 2953), scale bar = 2mm; *Carphophis amoenus* (MZUSP 8183), scale bar = 1mm. Figure J, Dipsadidae: *Synophis lasallei* (MZUSP 7713), scale bar = 1mm; *Nothopsis rugosus* (MZUSP 7490), scale bar = 1mm; *Dipsas indica* (IBSP 40137), scale bar = 1mm; *Atractus serranus* (MZUSP 17937), scale bar = 1mm. Figure K, Dipsadidae: *Boiruna maculata* (MZUSP 703), scale bar = 2mm; *Helicops angulatus* (MZUSP 14234), scale bar = 2mm; *Philodryas nattereri* (MZUSP 13039), scale bar = 2mm; *Oxyrhopus clathratus* (MZUSP 14010), scale bar = 2mm.
(PDF)

S3 Appendix Hemipenes. Hemipenial morphology of representatives of colubroidean families. Figure A, Acrochordidae: *Acrochordus javanicus* (LSUMZ 34406) completely everted and filled, scale bar = 5 mm; Xenodermidae: *Xenodermus javanicus* (FMNH 138678) partially everted, partially filled, and dyed with alizarin red, scale bar = 1 mm. Figure B, Xenodermidae: *Achalinus rufescens* (BMNH 1983.193) completely everted and partially filled, scale bar = 2 mm; *Fimbrios klossi* (BMNH 1965.2.639) opened through a longitudinal slit, one lobe partially filled, scale bar = 1 mm; Pareidae: *Pareas monticola* (BMNH 1909.3.9.19) completely everted and filled, scale bar = 1 mm. Figure C, Pareidae: *Asthenodipsas malaccanus* (BMNH 1924.10.23.7) completely everted and filled; *Aplopeltura boa* (BMNH 94.6.30.63) completely everted and filled; scale bars = 2 mm. Figure D, Xylophiidae: *Xylophis perroteti* (BMNH 1955.1.3.10) opened through a longitudinal slit, spread flat, and dyed with alizarin red, scale bar = 5 mm. Figure E, Viperidae: *Porthidum nasutum* (MZUSP 7480) completely everted and filled; *Vipera ammodytes* (MZUSP 8223) completely everted and filled; scale bars = 5 mm. Figure F, Viperidae: *Bothrops neuwiedi* (MZUSP 11851) completely everted and filled; *Causus bilineatus* (MNHN 1993.5992) completely everted and filled; scale bars = 5 mm. Figure G, Homalopsidae: *Homalopsis buccata* (MNHN 1963.728) completely everted and filled; *Brachyorrhos albus* (FMNH 142324) completely everted and filled; scale bars = 5 mm. Figure H, Homalopsidae: *Fordonia leucobalia* (AMNH 107179) completely everted, filled, and dyed with alizarin red; *Bitia hydroides* (FMNH 229568) completely everted and filled; scale bars = 5 mm. Figure I, Homalopsidae: *Erpeton tentaculatum* (AMNH 8850) completely everted and filled, scale bar = 5 mm. Psammophiidae: *Mimophis mahfalensis* (UMMZ 209646) completely everted and partially filled, scale bar = 2 mm; Atractaspididae: *Polemon christyi* (FMNH 219912) completely everted and filled, scale bar = 5 mm. Figure J, Atractaspididae: *Atractaspis fallax* (AMNH 102298) completely everted and filled, scale bar = 10 mm; *Macrelaps microlepidotus* (FMNH 205863) completely everted and filled, scale bar = 5 mm. Figure K, Cyclocoridae: *Cyclocorus lineatus* (MNHN 1900.411) opened through a longitudinal slit, spread flat, and dyed with alizarin red, scale bar = 5 mm; *Oxyrhabdion modestum* (FMNH 68907) completely everted, filled, and dyed with alizarin red, scale bar = 5 mm. Figure L, Lamprophiidae: *Lamprophis fuliginosus* (MNHN 1994.8111) completely everted and filled, scale bar = 5 mm; *Chamaelycus fasciatum* (BMNH 1909.4.29.2–3) completely everted and filled, scale bar = 2 mm; *Lycodonomorphus rufulus* (AMNH 140283) completely everted and filled, scale bar = 5 mm. Figure M, Lamprophiidae: *Lycophidion semicinctus* (MNHN 1995.3474) completely everted, filled, and dyed with alizarin red; *Mehelya capensis* (AMNH 73208) completely everted and filled; *Pseudoboodon lemniscatus* (MNHN 1905.185) completely everted and filled; scale bars = 5 mm. Figure N, Pseudoxyrhophiidae: *Dromicodryas bernieri* (UMMZ 218166) completely everted and filled, scale bar = 5 mm; *Duberria lutrix* (AMNH 115639) completely everted, filled, and dyed with alizarin red, scale bar = 3 mm; *Alluaudina bellyi* (UMMZ 209239) completely everted and filled, scale bar = 2 mm. Figure O, Pseudoxyrhophiidae: *Pseudoxyrhopus tritaeniatus* (UMMZ 195854) completely everted and filled; *Liophidium torquatum* (UMMZ 209430) completely everted and filled; scale bars = 5 mm. Figure P, Elapidae: *Naja melanoleuca* (BMNH 1959.1.7.69) completely everted and filled; *Micrurus frontalis* (IBSP 44331) completely everted and filled; scale bars = 5 mm. Figure Q, Elapidae: *Austrelaps superbus* (BMNH 1926.12.25.113) completely everted and filled; *Bungarus candidus* (BMNH 1937.11) completely everted and filled; scale bars = 5 mm. Figure R, Natricidae: *Atretium schistosum* (AMNH 85505) completely everted, filled, and dyed with alizarin red; *Lycognathophis seychellensis* (UMMZ 167994) completely everted, filled, and dyed with alizarin red; *Afronatrix anoscopus* (AMNH 142404) completely everted, filled, and dyed with alizarin red; scale bars = 2 mm. Figure S, Natricidae: *Xenochrophis vittatus* (BMNH 71.7.20.195–6) completely everted and filled; *Natriciteres olivacea* (AMNH 11905) completely everted and filled; *Sinonatrix annularis* (AMNH 84530) completely everted, filled, and dyed with alizarin red; scale bars = 2 mm. Figure T, Natricidae: *Aspidura trachyprocta* (AMNH 120248) completely everted and partially filled (no scale); *Elapoidis fusca* (MNHN 1895.55) completely everted and filled, scale bar = 2 mm. Figure U, Pseudoxenodontidae: *Pseudoxenodon macrops* (AMNH 34649) completely everted and filled; Dipsadidae: *Conophis pulcher* (MNHN 5981) completely everted and filled; scale bars = 5 mm. Figure V, Dipsadidae: *Contia tenius* (UMMZ 133370) completely everted and filled, scale bar = 2 mm; *Urotheca decipiens* (KU 103892) completely everted and filled, scale bar = 5 mm. Figure W, Dipsadidae: *Oxyrhopus occipitalis* (AMNH 129255) completely everted, filled, and dyed with alizarin red; *Farancia erythrogramma* (KU 197245) completely everted, filled, and dyed with alizarin red. Scale bars = 5 mm. Figure X, Dipsadidae: *Tachymenis chilensis* (MZUSP 8239) completely everted, filled, and dyed with alizarin red, scale bar = 2 mm; *Heterodon nasicus* (MNHN 3636) completely everted and filled, scale bar = 5 mm; *Philodryas olfersii* (IBSP 63455) completely everted, filled, and dyed with alizarin red, scale bar = 5 mm. Figure Y, Sibynophiidae: *Sibynophis chinensis* (AMNH 34102) completely everted and filled, scale bar = 2 mm; *Scaphiodontophis annulatus* (KU 191073) completely everted and filled, scale bar = 2 mm; Calamariidae: *Pseudorabdion longiceps* (BMNH 1969.1866) completely everted and partially filled, scale bar = 5 mm. Figure Z, Calamariidae: *Calamaria lumbricoidis* (BMNH 1928.2.18.26) completely everted and partially filled, scale bar = 5 mm; *Calamaria linnaei* (AMNH 31943) completely everted and partially filled, scale bar = 1 mm; *Oreocalamus hanitschi* (BMNH 1929.12.22.106) completely everted and partially filled, scale bar = 5 mm. Figure AA, Grayiidae: *Grayia ornata* (BMNH 98.3.25.3) completely everted and filled; Colubridae: *Pantherophis guttatus* (USNM 523605) completely everted and filled; *Spilotes sulphureus* (IBSP 68260) completely everted and filled. Scale bars = 10 mm. Figure AB, Colubridae: *Dispholidus typus* (AMNH 23110) completely everted and filled; *Hierophis viridiflavus* (MNHN 1978.414) completely everted and filled; *Boiga pulverulenta* (MNHN 1967.437) completely everted and filled; scale bars = 10 mm. Figure AC, Colubridae: *Ptyas korros* (AMNH 84460) completely everted and filled, scale bar = 10 mm; *Gongylosoma baliodeirus* (MNHN 1989.199) completely everted and filled, scale bar = 3 mm; *Liopeltis frenatus* (MNHN 1928.75) completely everted and filled, scale bar = 5 mm. Figure AD, Elapoidea Incertae sedis: *Buhoma depressiceps* (MNHN 1991.1740) completely everted, partially filled, and dyed with alizarin red, scale bar = 1 mm.
(PDF)
