# Correction: Large-scale molecular phylogeny, morphology, divergence-time estimation, and the fossil record of advanced caenophidian snakes (Squamata: Serpentes)

**DOI:** 10.1371/journal.pone.0217959

**Published:** 2019-05-31

**Authors:** Hussam Zaher, Robert W. Murphy, Juan Camilo Arredondo, Roberta Graboski, Paulo Roberto Machado-Filho, Kristin Mahlow, Giovanna G. Montingelli, Ana Bottallo Quadros, Nikolai L. Orlov, Mark Wilkinson, Ya-Ping Zhang, Felipe G. Grazziotin

There are errors in the Author Contributions. The correct contributions are: Conceptualization: HZ RWM FGG. Data curation: HZ RG KM FGG. Formal analysis: HZ RG MW FGG. Funding acquisition: HZ YPZ. Investigation: HZ MW FGG. Methodology: HZ MW FGG. Project administration: HZ YPZ. Resources: HZ FGG. Software: HZ FGG. Supervision: HZ RWM YPZ FGG. Validation: HZ RWM JCA RG PRMF GGM ABQ NLO MW YPZ FGG. Visualization: HZ JCA RG PRMF KM GGM ABQ NLO MW YPZ FGG. Writing–original draft: HZ JCA RG PRMF GGM ABQ MW FGG. Writing–review & editing: HZ RWM JCA RG PRMF KM GGM ABQ NLO MW YPZ FGG.
