# Supplementary material for: Large-scale molecular phylogeny, morphology, divergence-time estimation, and the fossil record of advanced caenophidian snakes (Squamata: Serpentes)
Source: PLoS One. 2019 May 10;14(5):e0216148. doi: 10.1371/journal.pone.0216148 (PMC6512042; doi:10.1371/journal.pone.0216148)
Supplement: S4 Table — Comparisons between the number of families, genera and species of Colubroides used in the present study and by Figueroa et al. [28] (2016). (DOCX) [file pone.0216148.s004.docx]

|  | **Statistics for genera** | | | **Statistics for species** | | |
| --- | --- | --- | --- | --- | --- | --- |
| **Families from Uetz et al.*** | **Total** | **This study** | | **Total** | **This study** | |
|  | **#** | **#** | **%** | **#** | **#** | **%** |
| Colubroides: Incertae Sedis | 9 | 2 | 22 | 15 | 2 | 13 |
| Xenodermidae | 6 | 4 | 67 | 18 | 5 | 28 |
| Pareidae | 3 | 3 | 100 | 20 | ***12*** | 60 |
| Viperidae: Viperinae | 13 | 12 | 92 | 100 | 70 | 70 |
| Viperidae: Crotalinae | 21 | 21 | 100 | 237 | 176 | 74 |
| Viperidae: Azemiopinae | 1 | 1 | 100 | 2 | 1 | 50 |
| Homalopsidae | 28 | 16 | 57 | 53 | 25 | 47 |
| Elapoidea: Incertae Sedis | 7 | 6 | 86 | 26 | 12 | 46 |
| Elapidae | 55 | 47 | 85 | 361 | ***196*** | 54 |
| Atractaspididae: Aparallactinae | 9 | 6 | 67 | 46 | 12 | 26 |
| Atractaspididae: Atractaspidinae | 2 | 2 | 100 | 23 | 7 | 30 |
| Lamprophiidae | 12 | 10 | 83 | 72 | 30 | 42 |
| Psammophiidae | 8 | 8 | 100 | 52 | ***41*** | 79 |
| Pseudoxyrhophiidae | 22 | 20 | 91 | 89 | ***55*** | 62 |
| Colubroidea: Incertae Sedis | 1 | 0 | 0 | 1 | 0 | 0 |
| Sibynophiidae: Scaphiodontophiinae | 1 | 1 | 100 | 2 | 1 | 50 |
| Sibynophiidae: Sibynophiinae | 1 | 1 | 100 | 9 | 5 | 56 |
| Calamariidae | 7 | 2 | 29 | 89 | 3 | 3 |
| Grayiidae | 1 | 1 | 100 | 4 | 3 | 75 |
| Colubridae | 102 | 79 | 77 | 736 | ***275*** | 37 |
| Natricidae | 38 | 26 | 68 | 231 | 89 | 39 |
| Pseudoxenodontidae | 2 | 2 | 100 | 10 | 4 | 40 |
| Dipsadidae: Incertae Sedis | 9 | 3 | 33 | 21 | 7 | 33 |
| Dipsadidae: Dipsadinae | 27 | 22 | 81 | 396 | ***67*** | 17 |
| Dipsadidae: Xenodontinae | 57 | 50 | 88 | 342 | 161 | 47 |
| Dipsadidae: Carphophiinae | 3 | 3 | 100 | 5 | 4 | 80 |
|  |  |  |  |  |  |  |
| **Total** | 444 | 347 | 78 | 2960 | ***1263*** | 42 |
|  |  |  |  |  |  |  |
| Colubroidea | 248 | 189 | 76 | 1846 | ***619*** | 34 |
| Sibynophiidae | 2 | 2 | 100 | 11 | 6 | 55 |
| Dipsadidae | 96 | 78 | 81 | 764 | ***239*** | 31 |
| Elapoidea | 115 | 99 | 86 | 669 | ***353*** | 53 |
| Atractaspididae | 11 | 8 | 73 | 69 | 19 | 28 |
| Viperidae | 35 | 34 | 97 | 339 | ***247*** | 73 |

* Total number of genera and species for each family are based on Uetz et al.[33]. Our data matrix includes 1263 terminal taxa (species) of Colubroides. However, 20 of our terminals are not recognized by Uetz et al. [33], which explains the difference in numbers of species sequenced listed above for some families (different values in bold and italicized). See S5 Table for more details.
