# Supplementary material for: Large-scale molecular phylogeny, morphology, divergence-time estimation, and the fossil record of advanced caenophidian snakes (Squamata: Serpentes)
Source: PLoS One. 2019 May 10;14(5):e0216148. doi: 10.1371/journal.pone.0216148 (PMC6512042; doi:10.1371/journal.pone.0216148)
Supplement: S6 Table — List of accession numbers, with genes names, current identification in GenBank, and probable correct identification for questionable and/or problematic sequences of snakes available in GenBank. (DOC) [file pone.0216148.s006.doc]

Our reasoning to select sequences from GenBank was based on five basic steps, as follows:

1. We downloaded all sequences for a particular locus for each species of Colubroides. If two or more sequences were present for the same species we chose the longest one to represent the species.
2. We checked for complete species identification and we did not use sequences identified as *confer* (cf*.*) or *affinis* (aff.).
3. We submitted all selected sequences to a BLAST search (<https://blast.ncbi.nlm.nih.gov/Blast.cgi>) and we evaluated the results until the 10^th^ genetically closest sequence as a proxy for contamination, misidentification or mislabeling of GenBank samples.
4. Additionally, we ran a quick RAxML analyses for each locus and we interpreted the relative position of each sequence searching for unusual results (e.g. sequences positioned in different families).
5. GenBank sequences presenting aberrant results as being related to (phylogeneticaly, RAxML) or similar to (genetically, BLAST) taxonomically distant taxa were removed for our matrix.

The table below indicates the sequences of Colubroides form GenBank that were classified as contaminated, misidentified or mislabeled. The probable re-identification with the accession number and percentage of identity is also provided.

| **Accession** | **Gene** | **Name in our tree** | **Named in NCBI as:** | **Probable re-identification** | **Accession** | **Ident** | **Author(s)** |
| --- | --- | --- | --- | --- | --- | --- | --- |
| AF236809 | cytb |  | *Philodryas aestiva* | *Callithrix emiliae* | L44587 | 99% | Araujo, et al (unpublished) |
| D31616 | 12S | *Calliophis macclellandi* | *Sinomicrurus macclellandi* | *Protobothrops mucrosquamatus* | AY289220 | 96% | Eguchi, (unpublished) |
| D31615 | 12S |  | *Hemibungarus japonicus* | *Protobothrops mucrosquamatus* | AY763187 | 95% | Eguchi, (unpublished) |
| Z46443 | 12S |  | *Rhamphiophis oxyrhynchus* | *Rhamphiophis rostratus* | FJ404126 | 100% | Heise, et al (1995) |
| D31610 | 12S |  | *Emydocephalus ijimae* | *Protobothrops mucrosquamatus* | AY223653 | 94% | Eguchi, (unpublished) |
| D31617 | cytb |  | *Laticauda semifasciata* | *Nerodia taxispilota* | AF402914 | 85% | Eguchi, (unpublished) |
| AY046573 | 12S |  | *Oxyrhopus rhombifer* | *Waglerophis merremi* | GQ457840 | 100% | Sineriz, et al (unpublished) |
| AY058927 | c-mos |  | *Demansia atra* | *Eunectes murinus* | AY099964 | 100% | Slowinski and Lawson, (unpublished) |
| AY058929 | c-mos |  | *Drysdalia coronata* | *Sanzinia madagascariensis* | AY099982 | 100% | Slowinski and Lawson, (unpublished) |
| Z46463 | 12S |  | *Chironius carinatus* | *Ptyas mucosus* | AY122828 | 93% | Heise, et al (1995) |
| DQ112075 | cytb | *Hapsidophrys smaragdina* | *Gastropyxis smaragdina* | *Coronella girondica* | AF471088 | 94% | Lawson, et al (2005) |
| AY223594 | cytb |  | *Bothrocophias microphthalmus* | *Bothrops bilineata* | AY223591 | 100% | Parkinson, et al (2002) |
| AY662612 | RAG1 |  | *Ramphotyphlops braminus^1^* | *Acontias meleagris* | AY662639 | 87% | Townsend, (unpublished) |
| AF428033 | cytb |  | *Dipsadoboa werneri* | *Buhoma procterae* | AY612001 | 100% | Gravlund, (2002) |
| GQ895876 | cytb | *Erythrolamprus reginae* | *Liophis reginae* | Nested within Dipsadinae, as the sister group of *Ninia atrata* |  |  | Pyron et al., (2011)^2^ |
| GQ895876 | c-mos | *Erythrolamprus reginae* | *Liophis reginae* | Nested within Dipsadinae |  |  | Pyron et al., (2011)^2^ |
| GQ895891 | cytb |  | *Thamnodynastes pallidus* | Nested within Dipsadinae, as the sister group of *Sibynomorphus mikanii* |  |  | Pyron et al., (2011)^2^ |
| GQ895832 | c-mos |  | *Thamnodynastes pallidus* | Nested within Dipsadinae, as the sister group of *Sibynomorphus mikanii* |  |  | Pyron et al., (2011)^2^ |
| GQ895878 | cytb |  | *Manolepis putnami* | ? |  |  | Pyron et al., (2011)^2^ |
|  | c-mos |  | *Manolepis putnami* | ? |  |  | Pyron et al., (2011)^2^ |
| GQ895891 | cytb |  | *Conophis vittatus* | Nested within Sonorini, as the sister group of *Conopsis biserialis* |  |  | Pyron et al., (2011)^2^ |
| GQ895806 | c-mos |  | *Conophis vittatus* | Same sequence as *Conopsis biserialis* |  |  | Pyron et al., (2011)^2^ |
| GQ895859 | cytb |  | *Clelia clelia* | Nested within Xenodontini, as the sister group of *Erythrolamprus miliaris* |  |  | Pyron et al., (2011)^2^ |
| GQ895803 | c-mos |  | *Clelia clelia* | Nested within Xenodontini |  |  | Pyron et al., (2011)^2^ |
| GQ895879 | cytb |  | *Ninia sebae* | Nested within Pseudoboini, as the sister group of *Drepanoides anomalus* |  |  | Pyron et al., (2011)^2^ |
| GQ895821 | c-mos |  | *Ninia sebae* | Nested within Pseudoboini, as the sister group of *Clelia clelia* |  |  | Pyron et al., (2011)^2^ |
| GQ895895 | cytb | *Xenodon merremi* | *Waglerophis merremi* | Nested within Pseudoboini, as the sister group of *Siphlophis cervinus* |  |  | Pyron et al., (2011)^2^ |
| GQ895836 | cytb | *Xenodon merremi* | *Waglerophis merremi* | Nested within Pseudoboini, inside the genus *Siphlophis* |  |  | Pyron et al., (2011)^2^ |
| GQ895878 | cytb |  | *Pliocercus elapoides* | Same sequence as *Manolepis putnami* |  |  | Pyron et al., (2011)^2^ |
| GQ895821 | c-mos |  | *Pliocercus elapoides* | Not clustering with any group ^3^ |  |  | Pyron et al., (2011)^2^ |
| GQ895894 | cytb |  | *Siphlophis compressus* | Nested within Tachymenini, as the sister group of *Thamnodynastes strigatus* |  |  | Pyron et al., (2011)^2^ |
| GQ895835 | c-mos |  | *Siphlophis compressus* | Nested within Tachymenini |  |  | Pyron et al., (2011)^2^ |
| GQ895861 | cytb |  | *Conopsis nasus* | Nested within Xenodontinae, as the sister group of *Psomophis* |  |  | Pyron et al., (2011)^2^ |
| GQ895805 | c-mos |  | *Conopsis nasus* | Sister group of *Calamaria* ^4^ |  |  | Pyron et al., (2011)^2^ |
| DQ902138 | cytb |  | *Zamenis longissimus^5^* |  |  |  | Burbrink & Lawson (2007) |
| DQ902114 | cytb |  | *Zamenis longissimus^5^* |  |  |  | Burbrink & Lawson (2007) |
| DQ902221 | nd2 |  | *Zamenis longissimus^5^* | Ptyas korros |  | 96.00% | Burbrink & Lawson (2007) |
| DQ902293 | nd4 |  | *Zamenis longissimus^5^* |  |  |  | Burbrink & Lawson (2007) |
| DQ902072 | cmos |  | *Zamenis longissimus^5^* |  |  |  | Burbrink & Lawson (2007) |
| GQ457773 | 16S |  | Siphlophis pulcher | Siphlophis longicaudatus |  |  |  |
| GQ457834 | 12S |  | *Siphlophis pulcher* | Siphlophis longicaudatus |  |  |  |
| GQ457894 | c-mos |  | *Siphlophis pulcher* | Siphlophis longicaudatus |  |  |  |

^1^ Sequence AY662612 of *Rhamphotyphlops braminus* is significantly distinct from other NCBI sequences of *R. braminus* (AY487410, AY444062, GU902633), showing several shared polymorphisms with sequences of scincid lizards deposited in NCBI

^2^ Sequence deposited in NCBI but not used by Pyron et al. (2011).

^3^ Sequence removed from our analysis, following a conservative approach (see text for an explanation).
