# Supplementary material for: Large-scale molecular phylogeny, morphology, divergence-time estimation, and the fossil record of advanced caenophidian snakes (Squamata: Serpentes)
Source: PLoS One. 2019 May 10;14(5):e0216148. doi: 10.1371/journal.pone.0216148 (PMC6512042; doi:10.1371/journal.pone.0216148)
Supplement: S7 Table — List with rationale for the species recognized in the present study, but not listed in Uetz et al. [33]. (DOCX) [file pone.0216148.s007.docx]

| *Apostolepis sanctaeritae* | Considered a synonym of A. cearensis by Ferrarezzi et al. (2005). However, A. ammodites is a junior synonym of A. sanctaeritae. For this reason, we recognize the validity of this species. |
| --- | --- |
| *Borikenophis prymnus* | A valid species according to Grazziotin et al. (2012). |
| *Bothrops colombiensis* | A valid species according to Wuster et al. (2002). Available evidence suggests this is a valid species (Oliveira et al., in prep.). |
| *Bothrops isabelae* | A valid species according to Wallach et al. (2014). Available evidence suggests this is a valid species (Oliveira et al., in prep.). |
| *Crotalus exsul* | Available sequences of C. exsul differ significantly from those of C. ruber added in this study. For this reason, we preferred to keep both species as valid terminal taxa (but see, Opinion 1960, Bulletin of Zoological Nomenclature 57(3), 189–190). |
| *Crotalus tortugensis* | A valid species according to Murphy et al. (2002) and Grismer (2002). |
| *Crotalus unicolor* | A valid species according to McCranie (1986) (but see, Campbell and Lamar, 1989). Available sequences of C. unicolor are distinct from those available to C. durissus. For this reason, we prefer to keep both speice as valid terminal taxa. |
| *Crotalus vegrandis* | A valid species according to Rivas et al. (2012). |
| *Dipsas neivai* | A valid species according to Cicchi et al. (2007) and available evidence at hand (Martinez et al., in prep.) |
| *Echis multisquamatus* | A possible valid species according to Uetz and Hosek (2014). |
| *Gloydius qinlingensis* | A valid species according to Xu et al. (2012). |
| *Gonionotophis unicolor* | A valid species according to Kelly et al. (2011) (but see, Benedetto and Broadley, 2014). |
| *Hydrophis ocellatus* | A valid species according to Kharin (2012) and Sanders et al. (2013). |
| *Lycodryas sanctijohannis* | A valid species according to Hawlitschek et al. (2011). |
| *Pareas macularius* | A valid species according to Sang et al. (2009). |
| *Pareas tonkinensis* | Considered a synonym of Pareas hamptoni by Vogel (2010). However, available sequences for both species are distinct. We thus retain both species as valid terminal taxa. |
| *Platyceps atayevi* | A valid species according to Nagy et al. (2004). (but see, Schatti et al., 2005). |
| *Platyceps rogersi* | A valid species according to Nagy et al. (2004) and Amr and Disi (2011). |
| *Platyceps rubriceps* | A valid species according to Nagy et al. (2004) (but see Amr and Disi, 2011). |
| *Sibynomorphus garmani* | A valid species according to Grazziotin et al. (2012) and Zaher et al. (2014). |
| *Thamnophis errans* | A valid species according to Rossman et al. (1996), Rossman e Burbrink (2005) and Ahumada-Carrillo et al. (2014). |
| *Vipera nikolskii* | A valid species according to McDiarmid et al. (1999) and Venchi e Sindaco (2006) (but see Zienko et al., 2010). |
| *Xenodon werneri* | A valid species according to Zaher (1999), Grazziotin et al. (2012), and Myers and McDowell (2014). |
