# Supplementary material for: Large-scale molecular phylogeny, morphology, divergence-time estimation, and the fossil record of advanced caenophidian snakes (Squamata: Serpentes)
Source: PLoS One. 2019 May 10;14(5):e0216148. doi: 10.1371/journal.pone.0216148 (PMC6512042; doi:10.1371/journal.pone.0216148)
Supplement: S3 Fig — Maximum likelihood species-level phylogeny of Colubroides including comparisons among values of FBP, SHL, and TBE support metrics. Numbers inside de squares on the nodes of the full tree represent the TBE values retrieved. (PDF) [file pone.0216148.s013.pdf]

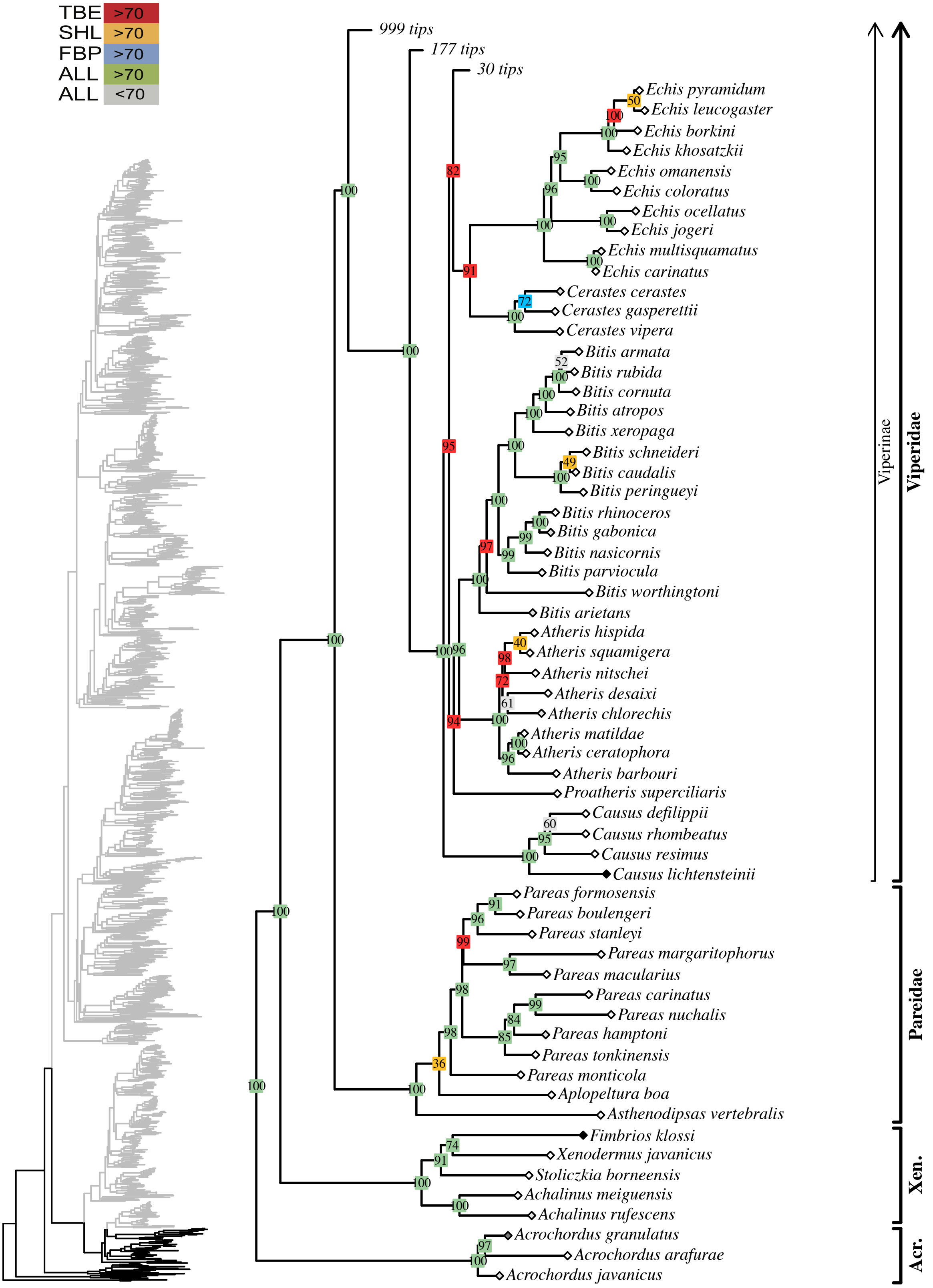
TBE >70
SHL >70
FBP >70
ALL >70
ALL <70
999 tips
177 tips
30 tips
Echis pyramidum
Echis leucogaster
Echis borkini
Echis khosatzkii
Echis omanensis
Echis coloratus
Echis ocellatus
Echis jogeri
Echis multisquamatus
Echis carinatus
Cerastes cerastes
Cerastes gasperettii
Cerastes vipera
Bitis armata
Bitis rubida
Bitis cornuta
Bitis atropos
Bitis xeropaga
Bitis schneideri
Bitis caudalis
Bitis peringueyi
Bitis rhinoceros
Bitis gabonica
Bitis nasicornis
Bitis parviocula
Bitis worthingtoni
Bitis arietans
Atheris hispida
Atheris squamigera
Atheris nitschei
Atheris desaixi
Atheris chlorechis
Atheris matildae
Atheris ceratophora
Atheris barbouri
Proatheris superciliaris
Causus defilippii
Causus rhombeatus
Causus resimus
Causus lichtensteinii
Pareas formosensis
Pareas boulengeri
Pareas stanleyi
Pareas margaritophorus
Pareas macularius
Pareas carinatus
Pareas nuchalis
Pareas hamptoni
Pareas tonkinensis
Pareas monticola
Aplopeltura boa
Asthenodipsas vertebralis
Fimbrios klossi
Xenodermus javanicus
Stoliczkia borneensis
Achalinus meiguensis
Achalinus rufescens
Acrochordus granulatus
Acrochordus arafurae
Acrochordus javanicus
Viperinae
Viperidae
Pareidae
Xen.
Acr.

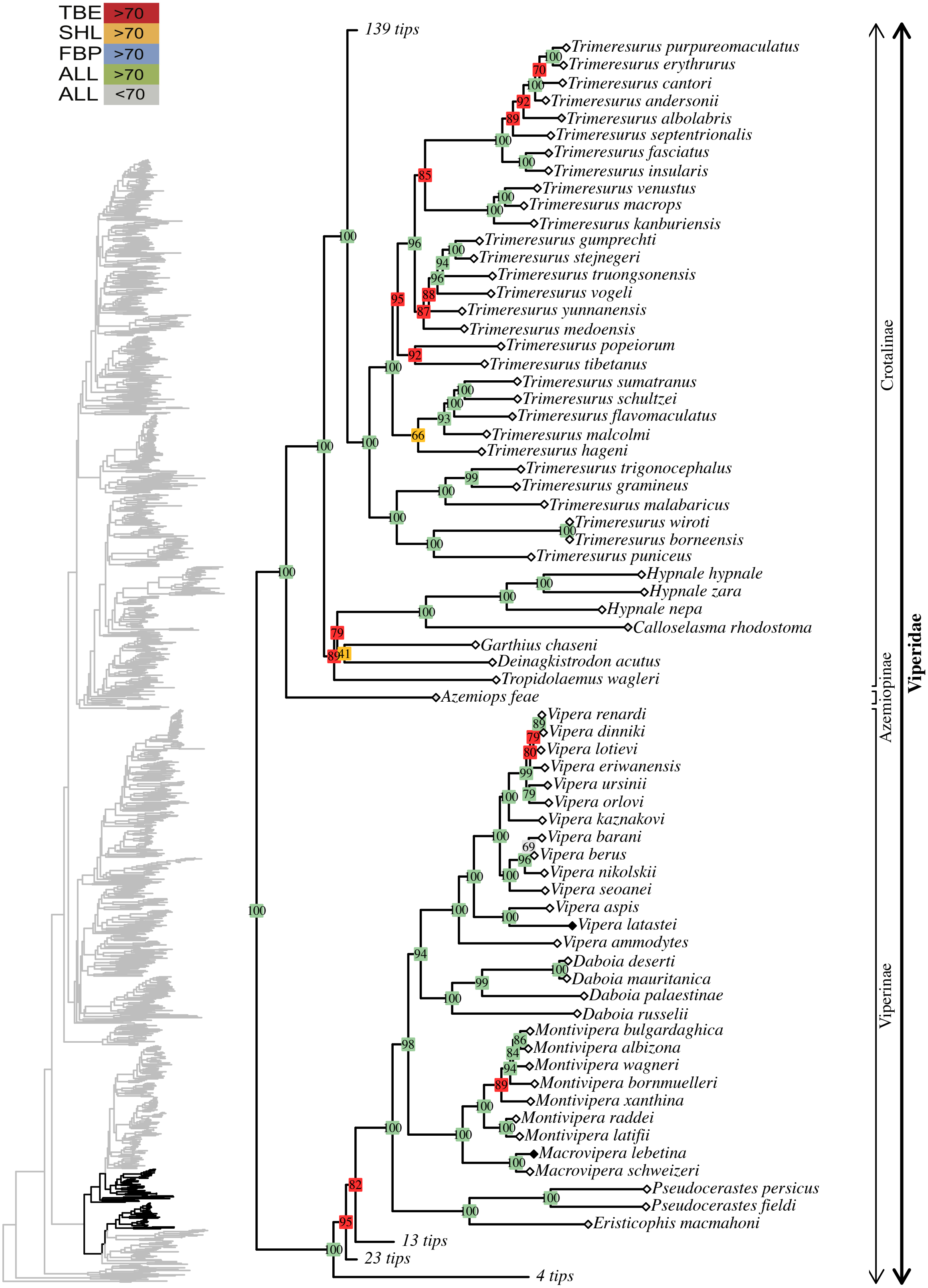

TBE >70
SHL >70
FBP >70
ALL >70
ALL <70
139 tips
Trimeresurus purpureomaculatus
Trimeresurus erythrurus
Trimeresurus cantori
Trimeresurus andersonii
Trimeresurus albolabris
Trimeresurus septentrionalis
Trimeresurus fasciatus
Trimeresurus insularis
Trimeresurus venustus
Trimeresurus macrops
Trimeresurus kanburiensis
Trimeresurus gumprechti
Trimeresurus stejnegeri
Trimeresurus truongsonensis
Trimeresurus vogeli
Trimeresurus yunnanensis
Trimeresurus medoensis
Trimeresurus popeiorum
Trimeresurus tibetanus
Trimeresurus sumatranus
Trimeresurus schultzei
Trimeresurus flavomaculatus
Trimeresurus malcolmi
Trimeresurus hageni
Trimeresurus trigonocephalus
Trimeresurus gramineus
Trimeresurus malabaricus
Trimeresurus wiroti
Trimeresurus borneensis
Trimeresurus puniceus
Hypnale hypnale
Hypnale zara
Hypnale nepa
Calloselasma rhodostoma
Garthius chaseni
Deinagkistrodon acutus
Tropidolaemus wagleri
Azemiops feae
Vipera renardi
Vipera dinniki
Vipera lotievi
Vipera eriwanensis
Vipera ursinii
Vipera orlovi
Vipera kaznakovi
Vipera barani
Vipera berus
Vipera nikolskii
Vipera seoanei
Vipera aspis
Vipera latastei
Vipera ammodytes
Daboia deserti
Daboia mauritanica
Daboia palaestinae
Daboia russelii
Montivipera bulgardaghica
Montivipera albizona
Montivipera wagneri
Montivipera bornmuelleri
Montivipera xanthina
Montivipera raddei
Montivipera latifii
Macrovipera lebetina
Macrovipera schweizeri
Pseudocerastes persicus
Pseudocerastes fieldi
Eristicophis macmahoni
13 tips
23 tips
4 tips
Crotalinae
Azemiopinae
Viperinae
Viperidae

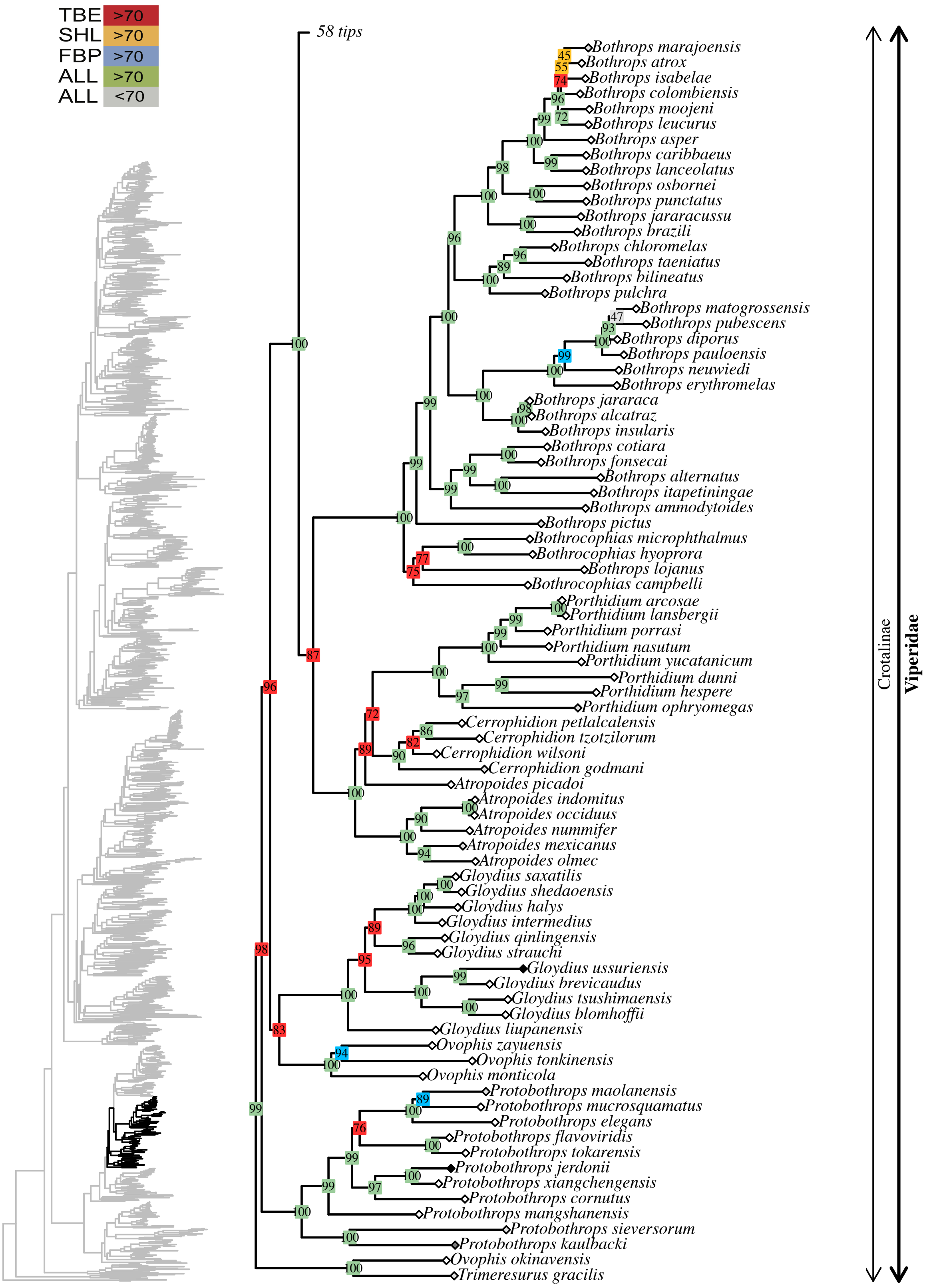

TBE >70
SHL >70
FBP >70
ALL >70
ALL <70
58 tips
Bothrops marajoensis
Bothrops atrox
Bothrops isabelae
Bothrops colombiensis
Bothrops moojeni
Bothrops leucurus
Bothrops asper
Bothrops caribbaeus
Bothrops lanceolatus
Bothrops osbornei
Bothrops punctatus
Bothrops jararacussu
Bothrops brazili
Bothrops chloromelas
Bothrops taeniatus
Bothrops bilineatus
Bothrops pulchra
Bothrops matogrossensis
Bothrops pubescens
Bothrops diporus
Bothrops pauloensis
Bothrops neuwiedi
Bothrops erythromelas
Bothrops jararaca
Bothrops alcatraz
Bothrops insularis
Bothrops cotiara
Bothrops fonsecai
Bothrops alternatus
Bothrops itapetiningae
Bothrops ammodytoides
Bothrops pictus
Bothrocophias microphthalmus
Bothrocophias hyoprora
Bothrops lojanus
Bothrocophias campbelli
Porthidium arcosae
Porthidium lansbergii
Porthidium porrasi
Porthidium nasutum
Porthidium yucatanicum
Porthidium dunni
Porthidium hespere
Porthidium ophryomegas
Cerrophidion petlalcalensis
Cerrophidion tzotzilorum
Cerrophidion wilsoni
Cerrophidion godmani
Atropoides picadoi
Atropoides indomitus
Atropoides occiduus
Atropoides nummifer
Atropoides mexicanus
Atropoides olmec
Gloydius saxatilis
Gloydius shedaoensis
Gloydius halys
Gloydius intermedius
Gloydius qinlingensis
Gloydius strauchi
Gloydius ussuriensis
Gloydius brevicaudus
Gloydius tsushimaensis
Gloydius blomhoffii
Gloydius liupanensis
Ovophis zayuensis
Ovophis tonkinensis
Ovophis monticola
Protobothrops maolanensis
Protobothrops mucrosquamatus
Protobothrops elegans
Protobothrops flavoviridis
Protobothrops tokarensis
Protobothrops jerdonii
Protobothrops xiangchengensis
Protobothrops cornutus
Protobothrops mangshanensis
Protobothrops sieversorum
Protobothrops kaulbacki
Ovophis okinavensis
Trimeresurus gracilis
Crotalinae
Viperidae

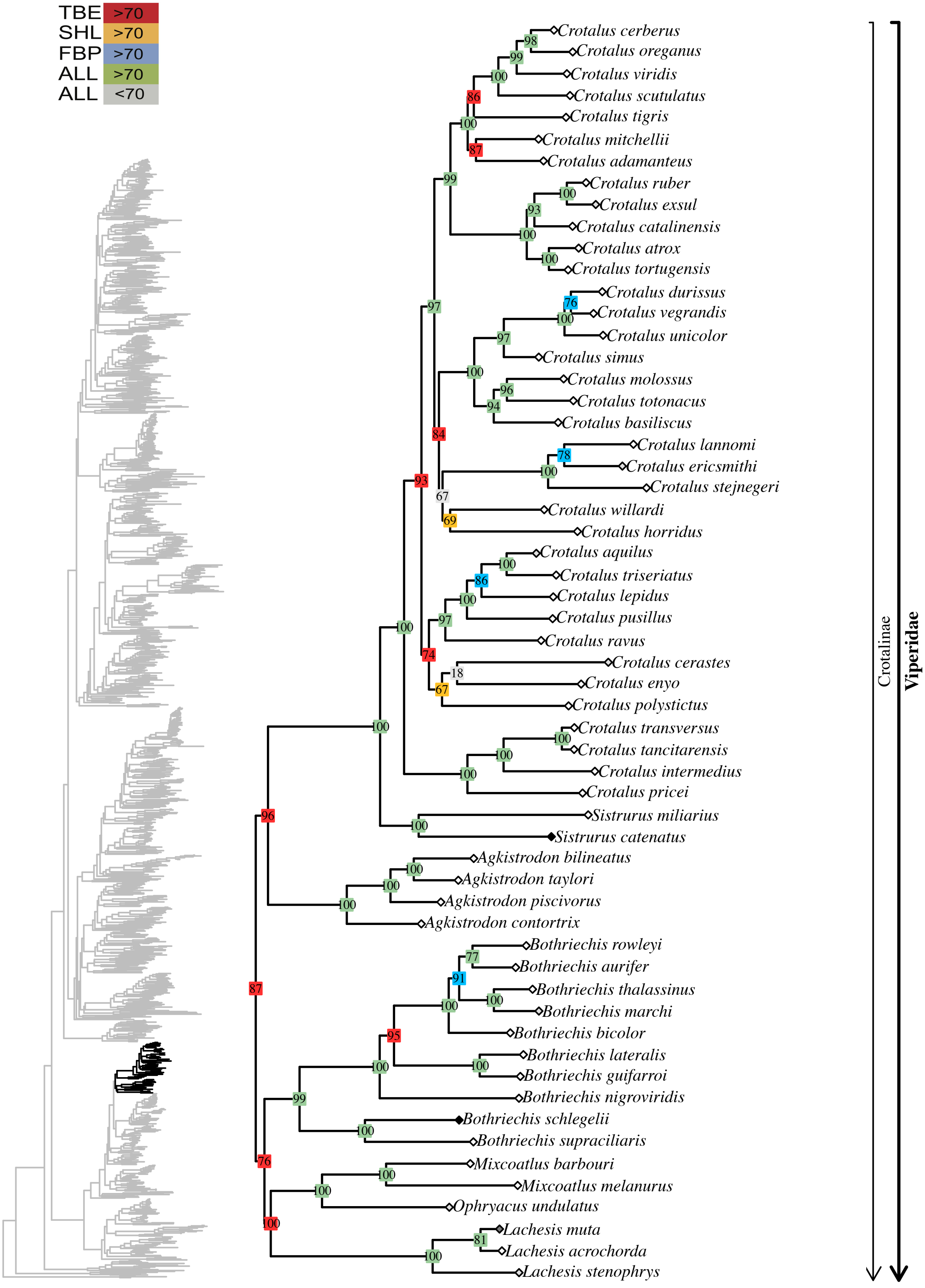

TBE >70
SHL >70
FBP >70
ALL >70
ALL <70
Crotalus cerberus
Crotalus oreganus
Crotalus viridis
Crotalus scutulatus
Crotalus tigris
Crotalus mitchellii
Crotalus adamanteus
Crotalus ruber
Crotalus exsul
Crotalus catalinensis
Crotalus atrox
Crotalus tortugensis
Crotalus durissus
Crotalus vegrandis
Crotalus unicolor
Crotalus simus
Crotalus molossus
Crotalus totonacus
Crotalus basiliscus
Crotalus lannomi
Crotalus ericsmithi
Crotalus stejnegeri
Crotalus willardi
Crotalus horridus
Crotalus aquilus
Crotalus triseriatus
Crotalus lepidus
Crotalus pusillus
Crotalus ravus
Crotalus cerastes
Crotalus enyo
Crotalus polystictus
Crotalus transversus
Crotalus tancitarensis
Crotalus intermedius
Crotalus pricei
Sistrurus miliarius
Sistrurus catenatus
Agkistrodon bilineatus
Agkistrodon taylori
Agkistrodon piscivorus
Agkistrodon contortrix
Bothriechis rowleyi
Bothriechis aurifer
Bothriechis thalassinus
Bothriechis marchi
Bothriechis bicolor
Bothriechis lateralis
Bothriechis guifarroi
Bothriechis nigroviridis
Bothriechis schlegelii
Bothriechis supraciliaris
Mixcoatlus barbouri
Mixcoatlus melanurus
Ophryacus undulatus
Lachesis muta
Lachesis acrochorda
Lachesis stenophrys
Crotalinae
Viperidae

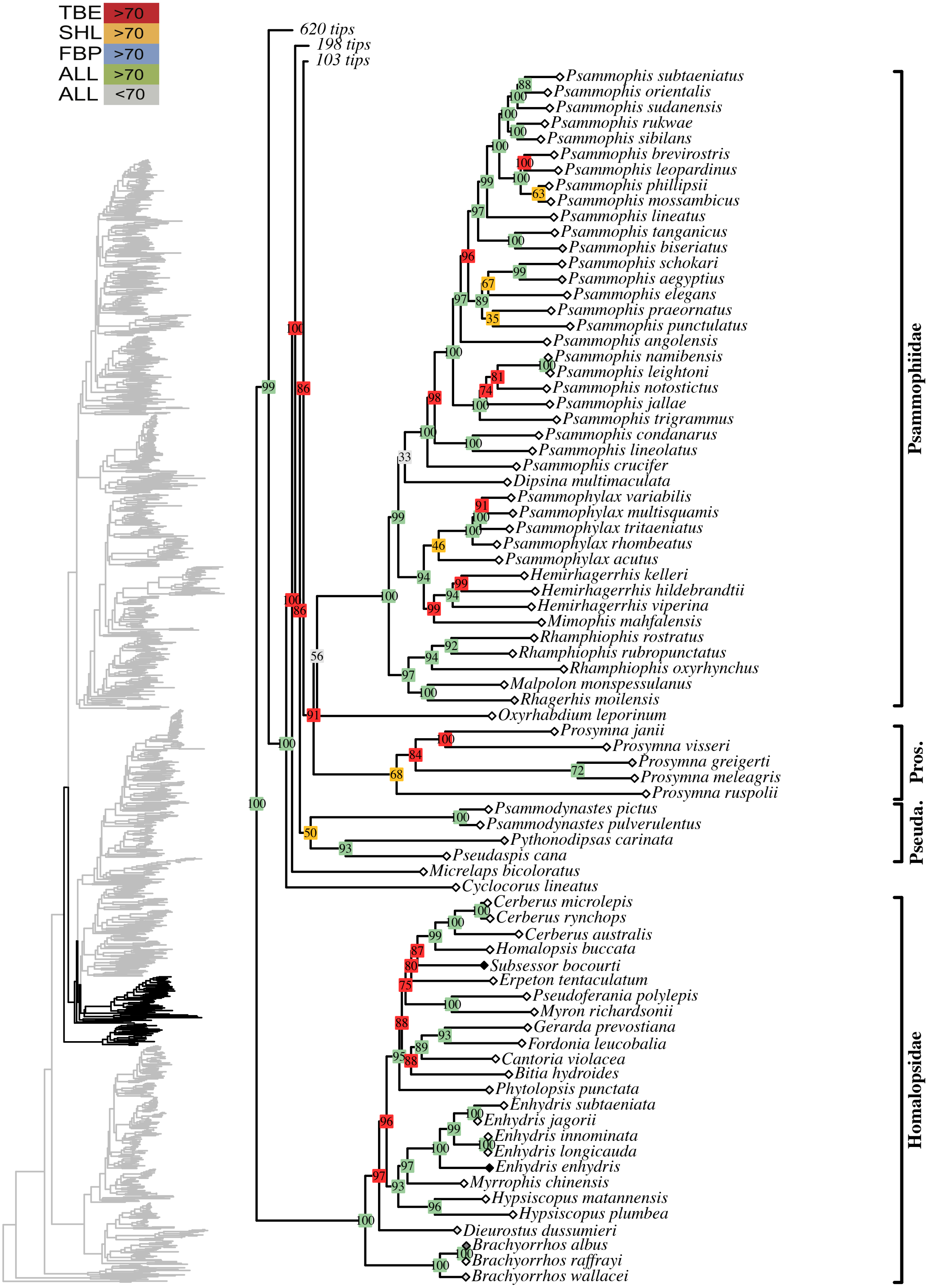

TBE >70
SHL >70
FBP >70
ALL >70
ALL <70
620 tips
198 tips
103 tips
Psammophis subtaeniatus
Psammophis orientalis
Psammophis sudanensis
Psammophis rukwae
Psammophis sibilans
Psammophis brevirostris
Psammophis leopardinus
Psammophis phillipsii
Psammophis mossambicus
Psammophis lineatus
Psammophis tanganicus
Psammophis biseriatus
Psammophis schokari
Psammophis aegyptius
Psammophis elegans
Psammophis praeornatus
Psammophis punctulatus
Psammophis angolensis
Psammophis namibensis
Psammophis leightoni
Psammophis notostictus
Psammophis jallae
Psammophis trigrammus
Psammophis condanarus
Psammophis lineolatus
Psammophis crucifer
Dipsina multimaculata
Psammophylax variabilis
Psammophylax multisquamis
Psammophylax tritaeniatus
Psammophylax rhombeatus
Psammophylax acutus
Hemirhagerrhis kelleri
Hemirhagerrhis hildebrandtii
Hemirhagerrhis viperina
Mimophis mahfalensis
Rhamphiophis rostratus
Rhamphiophis rubropunctatus
Rhamphiophis oxyrhynchus
Malpolon monspessulanus
Rhagerhis moilensis
Oxyrhabdium leporinum
Prosymna janii
Prosymna visseri
Prosymna greigerti
Prosymna meleagris
Prosymna ruspolii
Psammodynastes pictus
Psammodynastes pulverulentus
Pythonodipsas carinata
Pseudaspis cana
Micrelaps bicoloratus
Cyclocorus lineatus
Cerberus microlepis
Cerberus rynchops
Cerberus australis
Homalopsis buccata
Subsessor bocourti
Erpeton tentaculatum
Pseudoferania polylepis
Myron richardsonii
Gerarda prevostiana
Fordonia leucobalia
Cantoria violacea
Bitia hydroides
Phytolopsis punctata
Enhydris subtaeniata
Enhydris jagorii
Enhydris innominata
Enhydris longicauda
Enhydris enhydris
Myrrophis chinensis
Hypsiscopus matannensis
Hypsiscopus plumbea
Dieurostus dussumieri
Brachyorrhos albus
Brachyorrhos raffrayi
Brachyorrhos wallacei
Psammophiidae
Pros.
Pseuda.
Homalopsidae

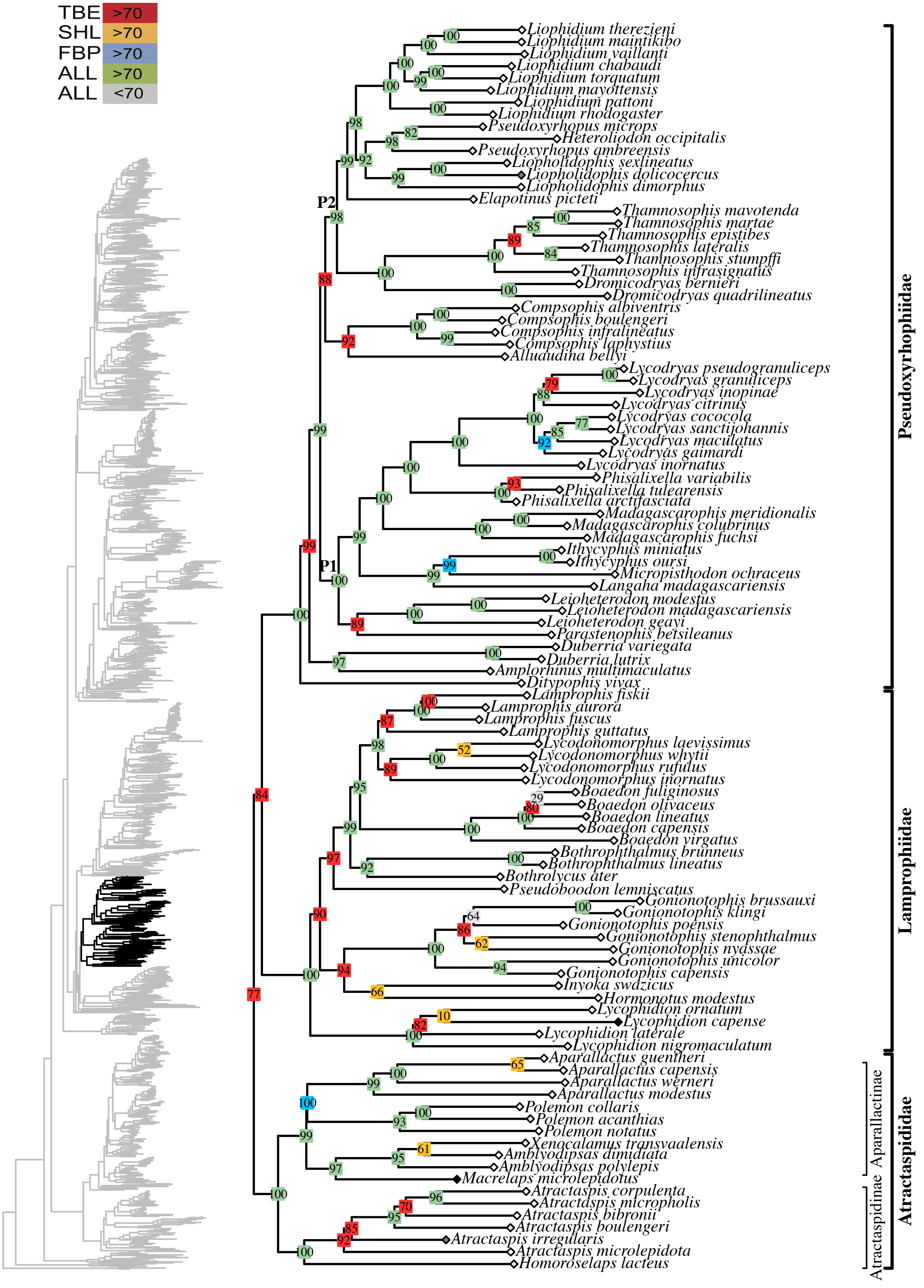

TBE >70
SHL >70
FBP >70
ALL >70
ALL <70
P2
P1
Liophidium therezieni
Liophidium maintikibo
Liophidium vaillanti
Liophidium chabaudi
Liophidium torquatum
Liophidium mayottensis
Liophidium pattoni
Liophidium rhodogaster
Pseudoxyrhopus microps
Heteroliodon occipitalis
Pseudoxyrhopus ambreensis
Liopholidophis sexlineatus
Liopholidophis dolicocercus
Liopholidophis dimorphus
Elapotinus picteti
Thamnosophis mavotenda
Thamnosophis martae
Thamnosophis epistibes
Thamnosophis lateralis
Thamnosophis stumpffi
Thamnosophis infrasignatus
Dromicodryas bernieri
Dromicodryas quadrilineatus
Compsophis albiventris
Compsophis boulengeri
Compsophis infralineatus
Compsophis laphystius
Alluaudina bellyi
Lycodryas pseudogranuliceps
Lycodryas granuliceps
Lycodryas inopinae
Lycodryas citrinus
Lycodryas cococola
Lycodryas sanctijohannis
Lycodryas maculatus
Lycodryas gaimardi
Lycodryas inornatus
Phisalixella variabilis
Phisalixella tulearensis
Phisalixella arctifasciata
Madagascarophis meridionalis
Madagascarophis colubrinus
Madagascarophis fuchsi
Ithycyphus miniatus
Ithycyphus oursi
Micropisthodon ochraceus
Langaha madagascariensis
Leioheterodon modestus
Leioheterodon madagascariensis
Leioheterodon geayi
Parastenophis betsileanus
Duberria variegata
Duberria lutrix
Amplorhinus multimaculatus
Ditypophis vivax
Lamprophis fiskii
Lamprophis aurora
Lamprophis fuscus
Lamprophis guttatus
Lycodonomorphus laevissimus
Lycodonomorphus whytii
Lycodonomorphus rufulus
Lycodonomorphus inornatus
Boaedon fuliginosus
Boaedon olivaceus
Boaedon lineatus
Boaedon capensis
Boaedon virgatus
Bothrophthalmus brunneus
Bothrophthalmus lineatus
Bothrolycus ater
Pseudoboodon lemniscatus
Gonionotophis brussauxi
Gonionotophis klingi
Gonionotophis poensis
Gonionotophis stenophthalmus
Gonionotophis nyassae
Gonionotophis unicolor
Gonionotophis capensis
Inyoka swazicus
Hormonotus modestus
Lycophidion ornatum
Lycophidion capense
Lycophidion laterale
Lycophidion nigromaculatum
Aparallactus guentheri
Aparallactus capensis
Aparallactus werneri
Aparallactus modestus
Polemon collaris
Polemon acanthias
Polemon notatus
Xenocalamus transvaalensis
Amblyodipsas dimidiata
Amblyodipsas polylepis
Macrelaps microlepidotus
Atractaspis corpulenta
Atractaspis micropholis
Atractaspis bibronii
Atractaspis boulengeri
Atractaspis irregularis
Atractaspis microlepidota
Homoroselaps lacteus
Pseudoxyrhophiidae
Lamprophiidae
Aparallactinae
Atractaspidinae
Atractaspididae

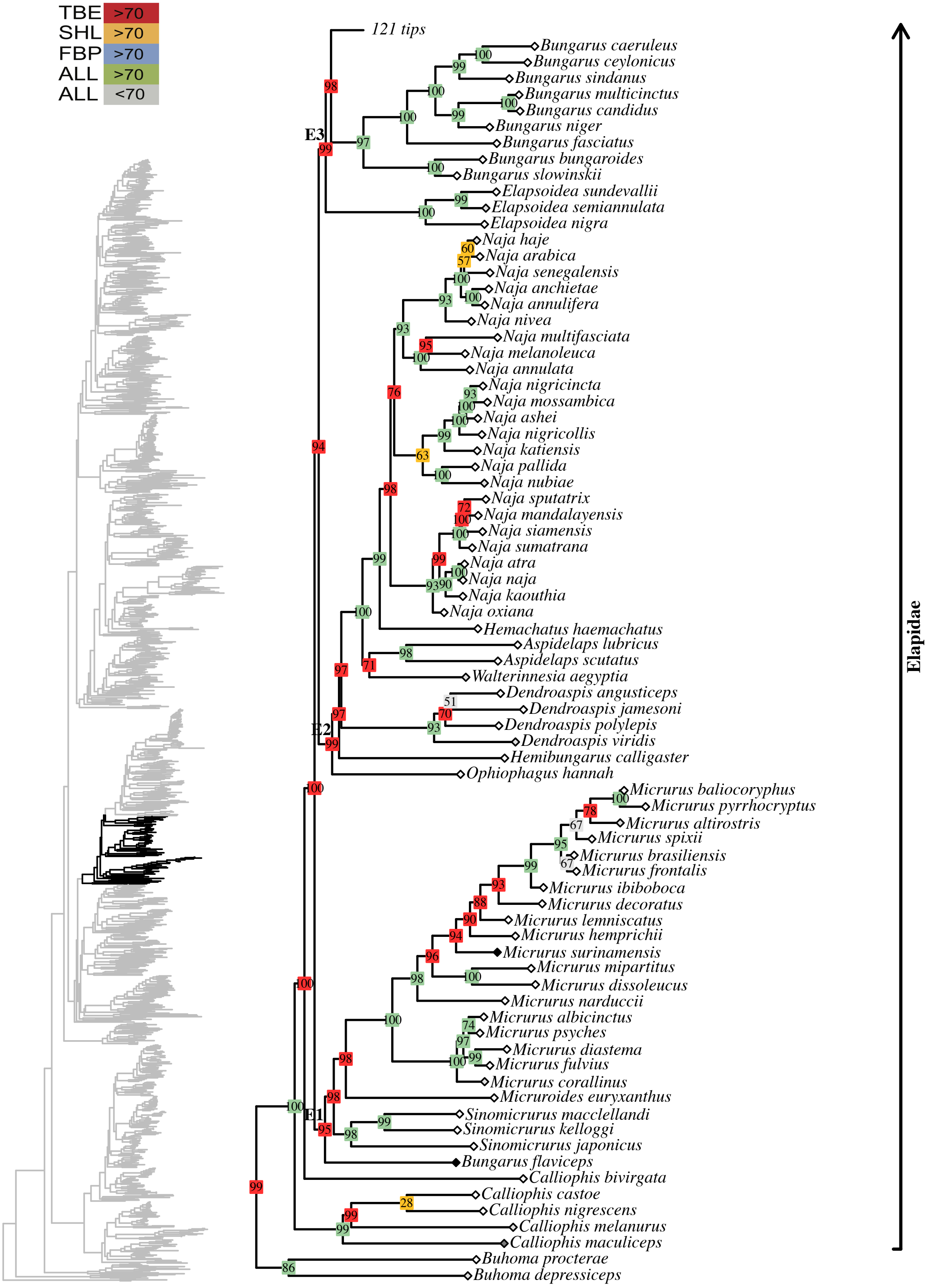

TBE >70
SHL >70
FBP >70
ALL >70
ALL <70
121 tips
E3
E2
E1
Bungarus caeruleus
Bungarus ceylonicus
Bungarus sindanus
Bungarus multicinctus
Bungarus candidus
Bungarus niger
Bungarus fasciatus
Bungarus bungaroides
Bungarus slowinskii
Elapsoidea sundevallii
Elapsoidea semiannulata
Elapsoidea nigra
Naja haje
Naja arabica
Naja senegalensis
Naja anchietae
Naja annulifera
Naja nivea
Naja multifasciata
Naja melanoleuca
Naja annulata
Naja nigricincta
Naja mossambica
Naja ashei
Naja nigricollis
Naja katiensis
Naja pallida
Naja nubiae
Naja sputatrix
Naja mandalayensis
Naja siamensis
Naja sumatrana
Naja atra
Naja naja
Naja kaouthia
Naja oxiana
Hemachatus haemachatus
Aspidelaps lubricus
Aspidelaps scutatus
Walterinnesia aegyptia
Dendroaspis angusticeps
Dendroaspis jamesoni
Dendroaspis polylepis
Dendroaspis viridis
Hemibungarus calligaster
Ophiophagus hannah
Micrurus baliocoryphus
Micrurus pyrrhocryptus
Micrurus altirostris
Micrurus spixii
Micrurus brasiliensis
Micrurus frontalis
Micrurus ibiboboca
Micrurus decoratus
Micrurus lemniscatus
Micrurus hemprichii
Micrurus surinamensis
Micrurus mipartitus
Micrurus dissoleucus
Micrurus narduccii
Micrurus albicinctus
Micrurus psyches
Micrurus diastema
Micrurus fulvius
Micrurus corallinus
Micruroides euryxanthus
Sinomicrurus macclellandi
Sinomicrurus kelloggi
Sinomicrurus japonicus
Bungarus flaviceps
Calliophis bivirgata
Calliophis castoe
Calliophis nigrescens
Calliophis melanurus
Calliophis maculiceps
Buhoma procterae
Buhoma depressiceps
Elapidae

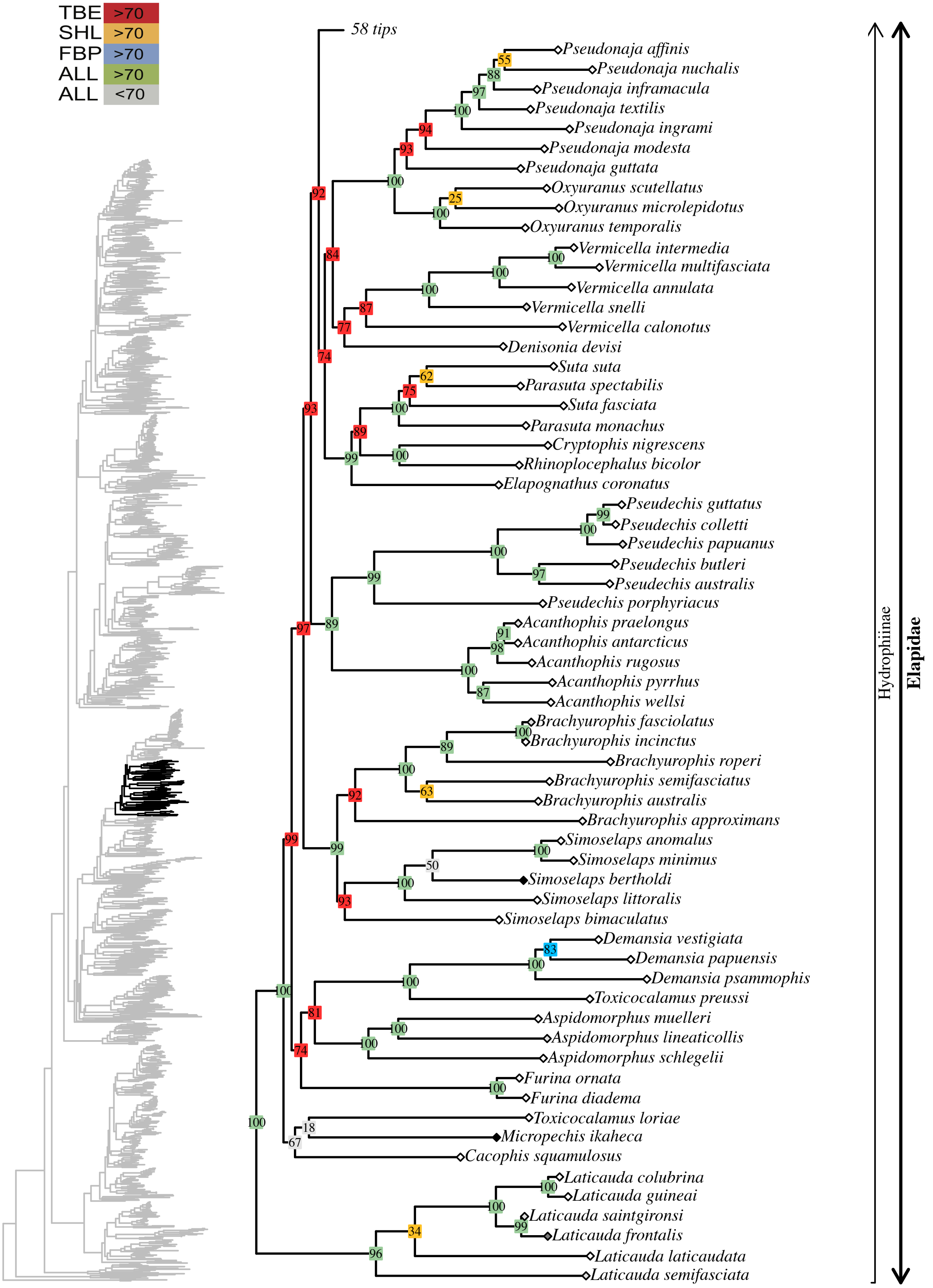

TBE >70
SHL >70
FBP >70
ALL >70
ALL <70
58 tips
Pseudonaja affinis
Pseudonaja nuchalis
Pseudonaja inframacula
Pseudonaja textilis
Pseudonaja ingrami
Pseudonaja modesta
Pseudonaja guttata
Oxyuranus scutellatus
Oxyuranus microlepidotus
Oxyuranus temporalis
Vermicella intermedia
Vermicella multifasciata
Vermicella annulata
Vermicella snelli
Vermicella calonotus
Denisonia devisi
Suta suta
Parasuta spectabilis
Suta fasciata
Parasuta monachus
Cryptophis nigrescens
Rhinoplocephalus bicolor
Elapognathus coronatus
Pseudechis guttatus
Pseudechis colletti
Pseudechis papuanus
Pseudechis butleri
Pseudechis australis
Pseudechis porphyriacus
Acanthophis praelongus
Acanthophis antarcticus
Acanthophis rugosus
Acanthophis pyrrhus
Acanthophis wellsi
Brachyurophis fasciolatus
Brachyurophis incinctus
Brachyurophis roperi
Brachyurophis semifasciatus
Brachyurophis australis
Brachyurophis approximans
Simoselaps anomalus
Simoselaps minimus
Simoselaps bertholdi
Simoselaps littoralis
Simoselaps bimaculatus
Demansia vestigiata
Demansia papuensis
Demansia psammophis
Toxicocalamus preussi
Aspidomorphus muelleri
Aspidomorphus lineaticollis
Aspidomorphus schlegelii
Furina ornata
Furina diadema
Toxicocalamus loriae
Micropechis ikaheca
Cacophis squamulosus
Laticauda colubrina
Laticauda guineai
Laticauda saintgironsi
Laticauda frontalis
Laticauda laticaudata
Laticauda semifasciata
Hydrophiinae
Elapidae

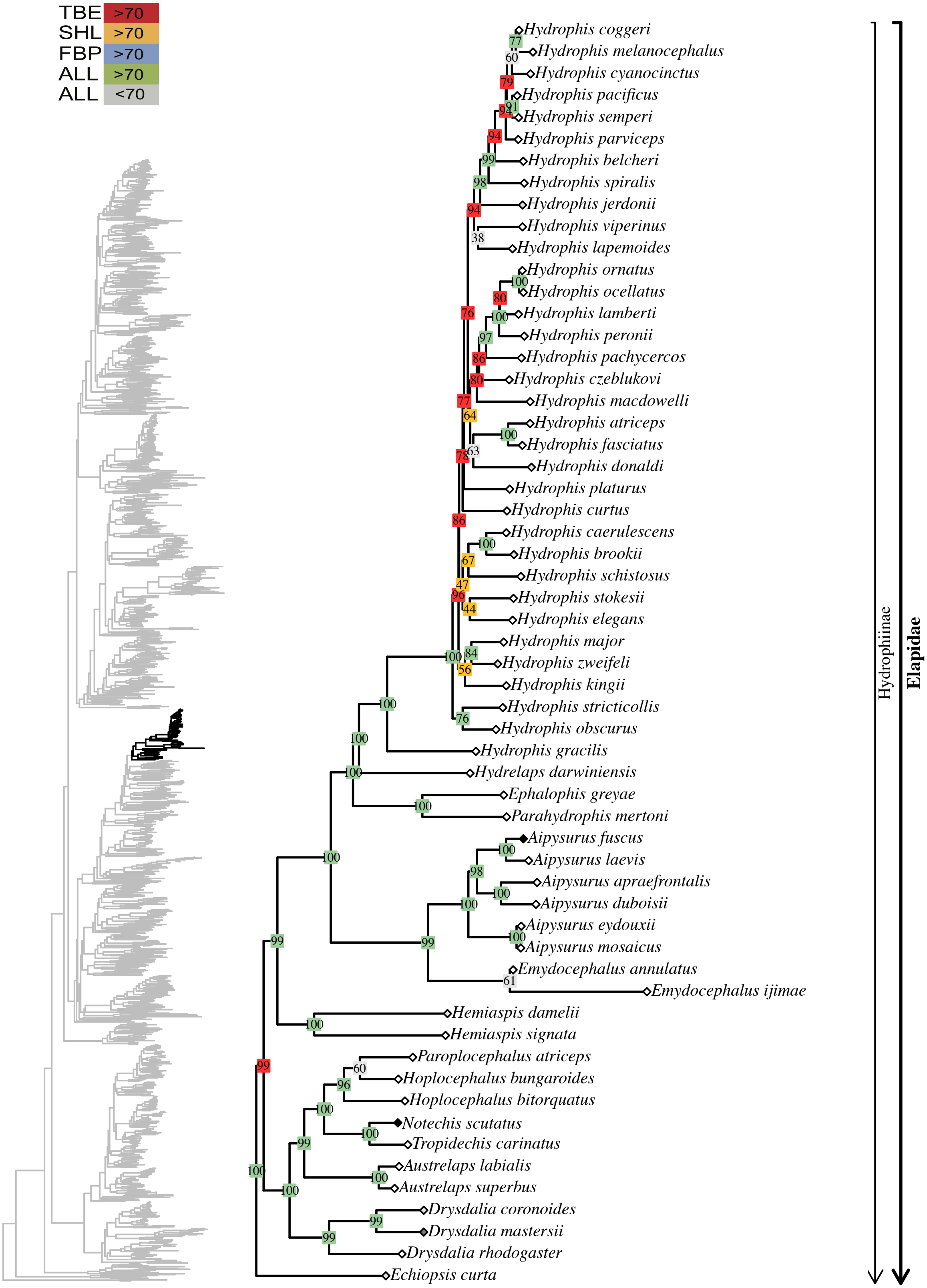

TBE >70
SHL >70
FBP >70
ALL >70
ALL <70
Hydrophis coggeri
Hydrophis melanocephalus
Hydrophis cyanocinctus
Hydrophis pacificus
Hydrophis semperi
Hydrophis parviceps
Hydrophis belcheri
Hydrophis spiralis
Hydrophis jerdonii
Hydrophis viperinus
Hydrophis lapemoides
Hydrophis ornatus
Hydrophis ocellatus
Hydrophis lamberti
Hydrophis peronii
Hydrophis pachycercos
Hydrophis czeblukovi
Hydrophis macdowelli
Hydrophis atriceps
Hydrophis fasciatus
Hydrophis donaldi
Hydrophis platurus
Hydrophis curtus
Hydrophis caerulescens
Hydrophis brookii
Hydrophis schistosus
Hydrophis stokesii
Hydrophis elegans
Hydrophis major
Hydrophis zweifeli
Hydrophis kingii
Hydrophis stricticollis
Hydrophis obscurus
Hydrophis gracilis
Hydrelaps darwiniensis
Ephalophis greyae
Parahydrophis mertoni
Aipysurus fuscus
Aipysurus laevis
Aipysurus apraefrontalis
Aipysurus duboisii
Aipysurus eydouxii
Aipysurus mosaicus
Emydocephalus annulatus
Emydocephalus ijimae
Hemiaspis damelii
Hemiaspis signata
Paroplocephalus atriceps
Hoplocephalus bungaroides
Hoplocephalus bitorquatus
Notechis scutatus
Tropidechis carinatus
Austrelaps labialis
Austrelaps superbus
Drysdalia coronoides
Drysdalia mastersii
Drysdalia rhodogaster
Echiopsis curta
Hydrophiinae
Elapidae

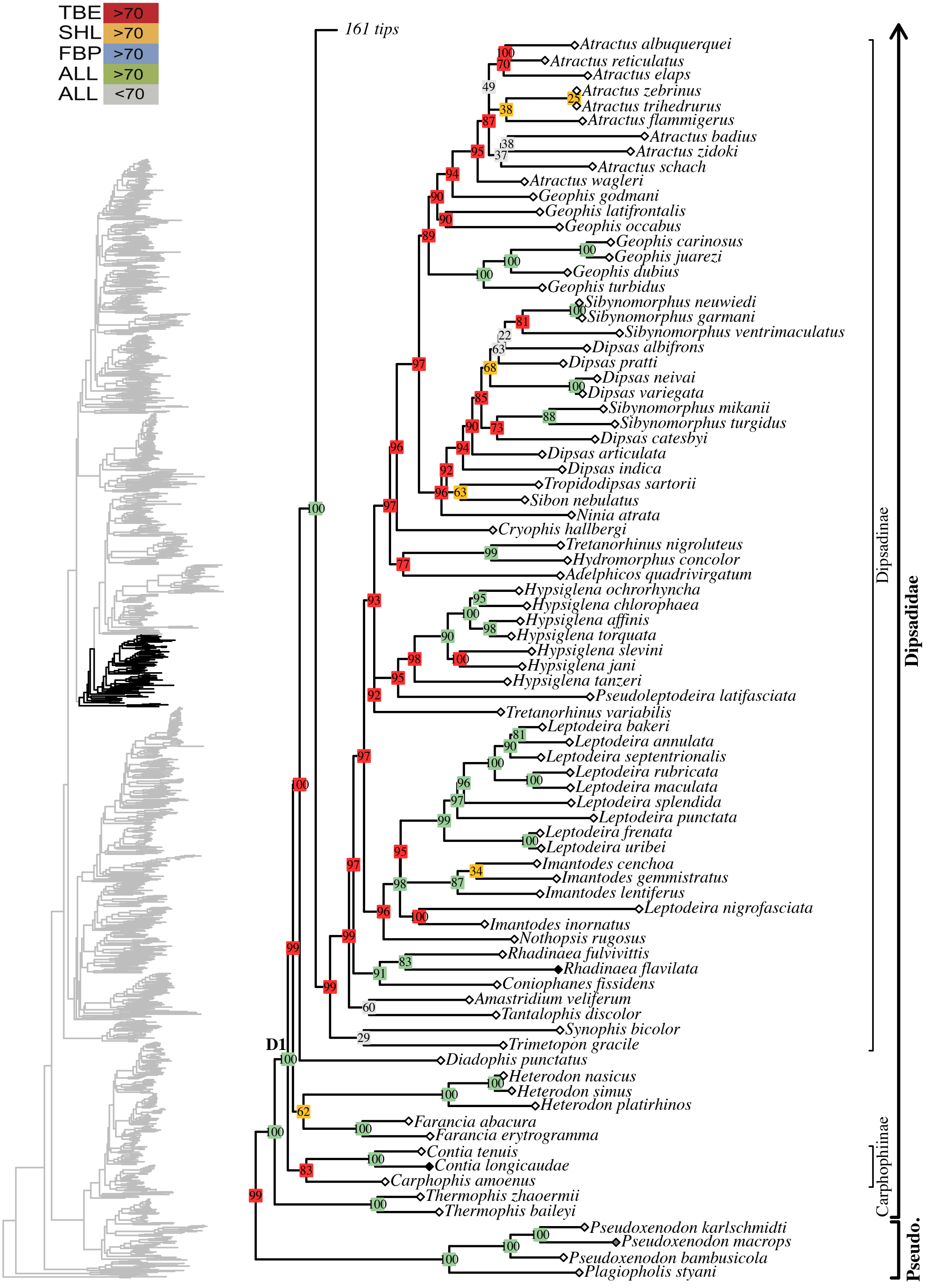

TBE >70
SHL >70
FBP >70
ALL >70
ALL <70
161 tips
Atractus albuquerquei
Atractus reticulatus
Atractus elaps
Atractus zebrinus
Atractus trihedrurus
Atractus flammigerus
Atractus badius
Atractus zidoki
Atractus schach
Atractus wagleri
Geophis godmani
Geophis latifrontalis
Geophis occabus
Geophis carinosus
Geophis juarezi
Geophis dubius
Geophis turbidus
Sibynomorphus neuwiedi
Sibynomorphus garmani
Sibynomorphus ventrimaculatus
Dipsas albifrons
Dipsas pratti
Dipsas neivai
Dipsas variegata
Sibynomorphus mikanii
Sibynomorphus turgidus
Dipsas catesbyi
Dipsas articulata
Dipsas indica
Tropidodipsas sartorii
Sibon nebulatus
Ninia atrata
Cryophis hallbergi
Tretanorhinus nigroluteus
Hydromorphus concolor
Adelphicos quadrivirgatum
Hypsiglena ochrorhyncha
Hypsiglena chlorophaea
Hypsiglena affinis
Hypsiglena torquata
Hypsiglena slevini
Hypsiglena jani
Hypsiglena tanzeri
Pseudoleptodeira latifasciata
Tretanorhinus variabilis
Leptodeira bakeri
Leptodeira annulata
Leptodeira septentrionalis
Leptodeira rubricata
Leptodeira maculata
Leptodeira splendida
Leptodeira punctata
Leptodeira frenata
Leptodeira uribei
Imantodes cenchoa
Imantodes gemmistratus
Imantodes lentiferus
Leptodeira nigrofasciata
Imantodes inornatus
Nothopsis rugosus
Rhadinaea fulvivittis
Rhadinaea flavilata
Coniophanes fissidens
Amastridium veliferum
Tantalophis discolor
Synophis bicolor
Trimetopon gracile
Diadophis punctatus
Heterodon nasicus
Heterodon simus
Heterodon platirhinos
Farancia abacura
Farancia erytrogramma
Contia tenuis
Contia longicaudae
Carphophis amoenus
Thermophis zhaoermii
Thermophis baileyi
Pseudoxenodon karlschmidti
Pseudoxenodon macrops
Pseudoxenodon bambusicola
Plagiopholis styani
D1
Dipsadinae
Carphophiinae
Dipsadidae
Pseudo.

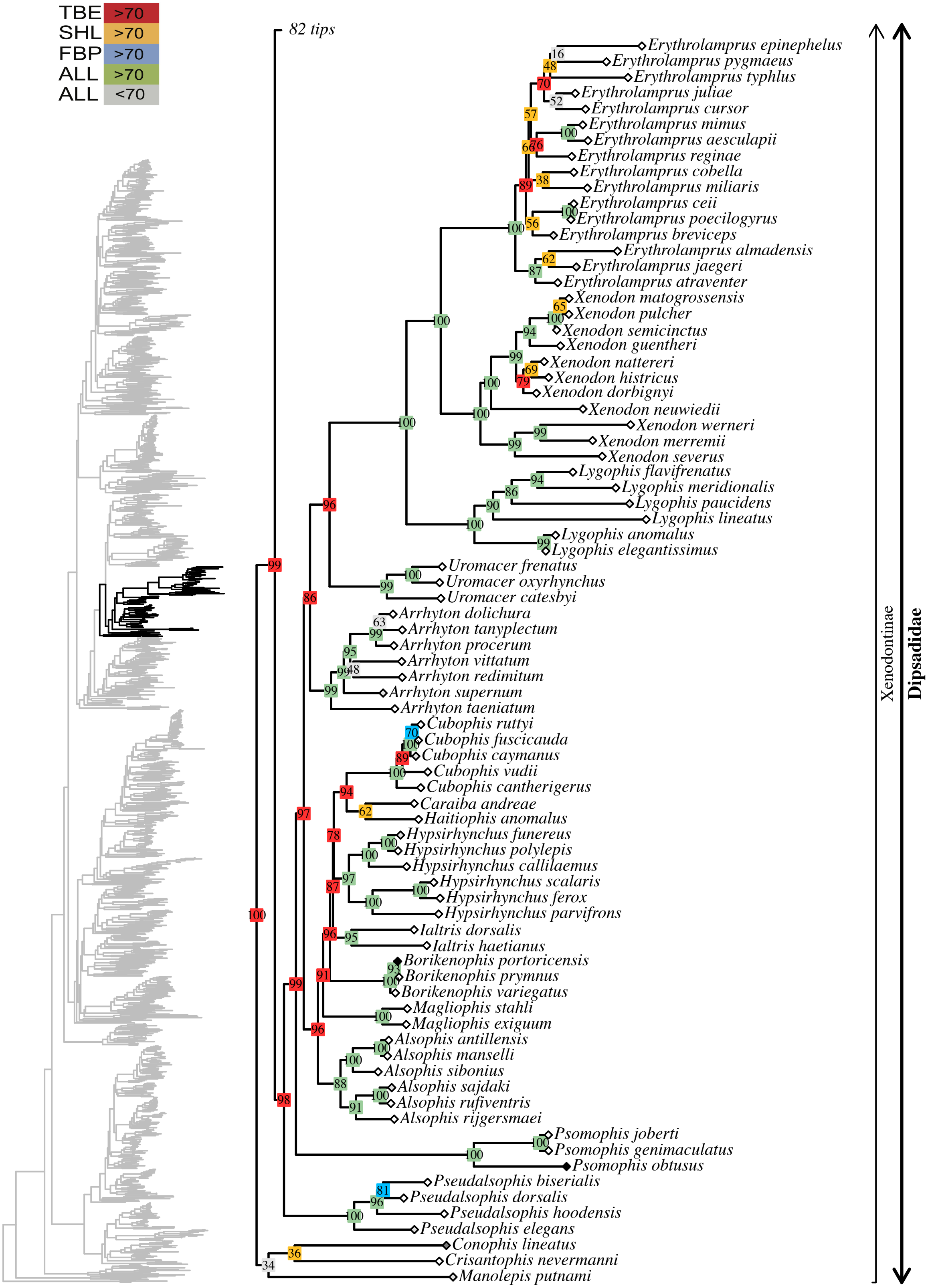

TBE >70
SHL >70
FBP >70
ALL >70
ALL <70
82 tips
Erythrolamprus epinephelus
Erythrolamprus pygmaeus
Erythrolamprus typhlus
Erythrolamprus juliae
Erythrolamprus cursor
Erythrolamprus mimus
Erythrolamprus aesculapii
Erythrolamprus reginae
Erythrolamprus cobella
Erythrolamprus miliaris
Erythrolamprus ceii
Erythrolamprus poecilogyrus
Erythrolamprus breviceps
Erythrolamprus almadensis
Erythrolamprus jaegeri
Erythrolamprus atraventer
Xenodon matogrossensis
Xenodon pulcher
Xenodon semicinctus
Xenodon guentheri
Xenodon nattereri
Xenodon histricus
Xenodon dorbignyi
Xenodon neuwiedii
Xenodon werneri
Xenodon merremii
Xenodon severus
Lygophis flavifrenatus
Lygophis meridionalis
Lygophis paucidens
Lygophis lineatus
Lygophis anomalus
Lygophis elegantissimus
Uromacer frenatus
Uromacer oxyrhynchus
Uromacer catesbyi
Arrhyton dolichura
Arrhyton tanyplectum
Arrhyton procerum
Arrhyton vittatum
Arrhyton redimitum
Arrhyton supernum
Arrhyton taeniatum
Cubophis ruttyi
Cubophis fuscicauda
Cubophis caymanus
Cubophis vudii
Cubophis cantherigerus
Caraiba andreae
Haitiophis anomalus
Hypsirhynchus funereus
Hypsirhynchus polylepis
Hypsirhynchus callilaemus
Hypsirhynchus scalaris
Hypsirhynchus ferox
Hypsirhynchus parvifrons
Ialtris dorsalis
Ialtris haetianus
Borikenophis portoricensis
Borikenophis prymnus
Borikenophis variegatus
Magliophis stahli
Magliophis exiguum
Alsophis antillensis
Alsophis manselli
Alsophis sibonius
Alsophis sajdaki
Alsophis rufiventris
Alsophis rijgersmaei
Psomophis joberti
Psomophis genimaculatus
Psomophis obtusus
Pseudalsophis biserialis
Pseudalsophis dorsalis
Pseudalsophis hoodensis
Pseudalsophis elegans
Conophis lineatus
Crisantophis nevermanni
Manolepis putnami
Xenodontinae
Dipsadidae

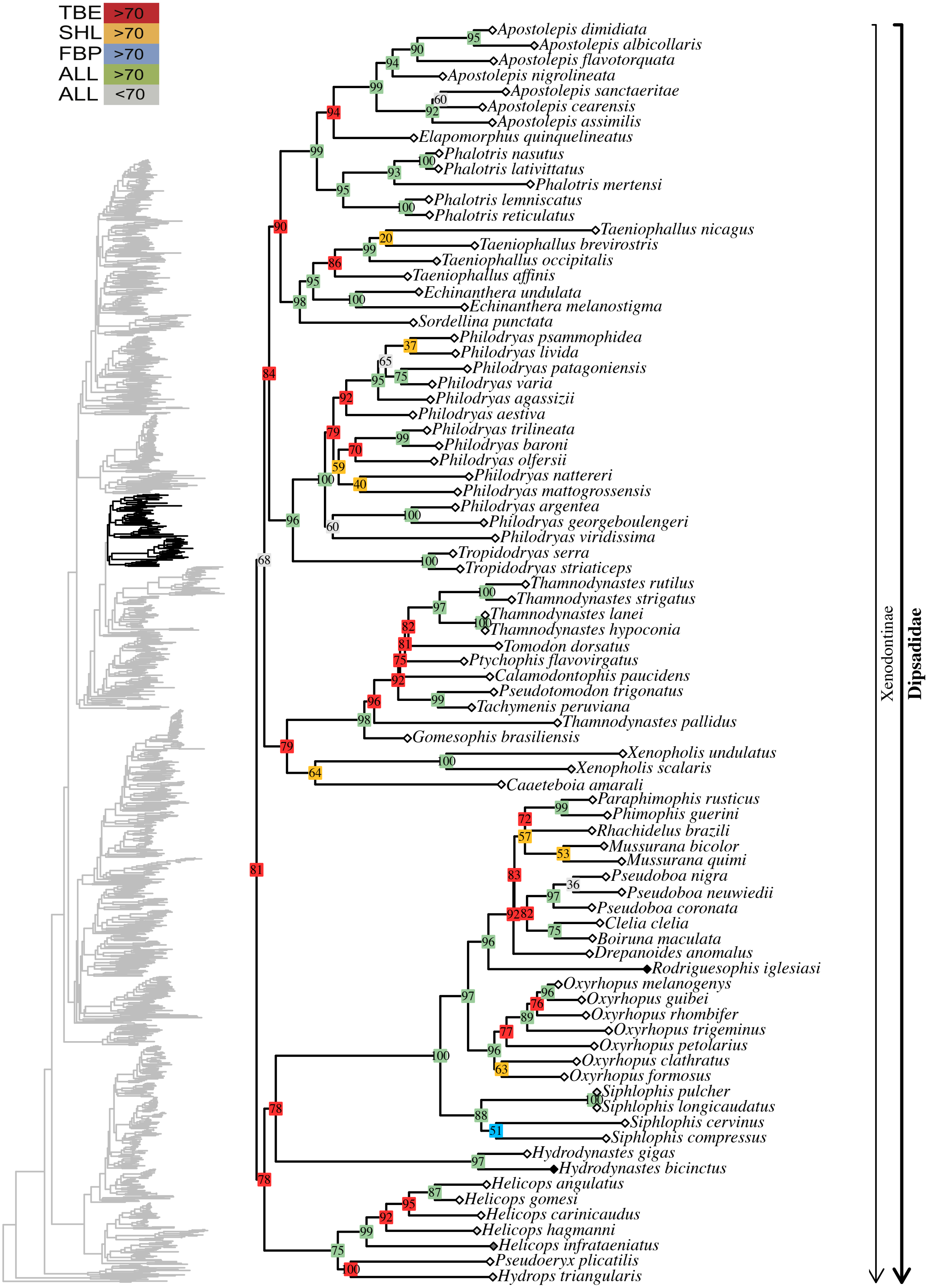
TBE >70
SHL >70
FBP >70
ALL >70
ALL <70
Apostolepis dimidiata
Apostolepis albicollaris
Apostolepis flavotorquata
Apostolepis nigrolineata
Apostolepis sanctaeritae
Apostolepis cearensis
Apostolepis assimilis
Elapomorphus quinquelineatus
Phalotris nasutus
Phalotris lativittatus
Phalotris mertensi
Phalotris lemniscatus
Phalotris reticulatus
Taeniophallus nicagus
Taeniophallus brevirostris
Taeniophallus occipitalis
Taeniophallus affinis
Echinanthera undulata
Echinanthera melanostigma
Sordellina punctata
Philodryas psammophidea
Philodryas livida
Philodryas patagoniensis
Philodryas varia
Philodryas agassizii
Philodryas aestiva
Philodryas trilineata
Philodryas baroni
Philodryas olfersii
Philodryas nattereri
Philodryas mattogrossensis
Philodryas argentea
Philodryas georgeboulengeri
Philodryas viridissima
Tropidodryas serra
Tropidodryas striaticeps
Thamnodynastes rutilus
Thamnodynastes strigatus
Thamnodynastes lanei
Thamnodynastes hypoconia
Tomodon dorsatus
Ptychophis flavovirgatus
Calamodontophis paucidens
Pseudotomodon trigonatus
Tachymenis peruviana
Thamnodynastes pallidus
Gomesophis brasiliensis
Xenopholis undulatus
Xenopholis scalaris
Caaeteboia amarali
Paraphimophis rusticus
Phimophis guerini
Rhachidelus brazili
Mussurana bicolor
Mussurana quimi
Pseudoboa nigra
Pseudoboa neuwiedii
Pseudoboa coronata
Clelia clelia
Boiruna maculata
Drepanoides anomalus
Rodriguesophis iglesiasi
Oxyrhopus melanogenys
Oxyrhopus guibei
Oxyrhopus rhombifer
Oxyrhopus trigeminus
Oxyrhopus petolarius
Oxyrhopus clathratus
Oxyrhopus formosus
Siphlophis pulcher
Siphlophis longicaudatus
Siphlophis cervinus
Siphlophis compressus
Hydrodynastes gigas
Hydrodynastes bicinctus
Helicops angulatus
Helicops gomesi
Helicops carinicaudus
Helicops hagmanni
Helicops infrataeniatus
Pseudoeryx plicatilis
Hydrops triangularis
Xenodontinae
Dipsadidae

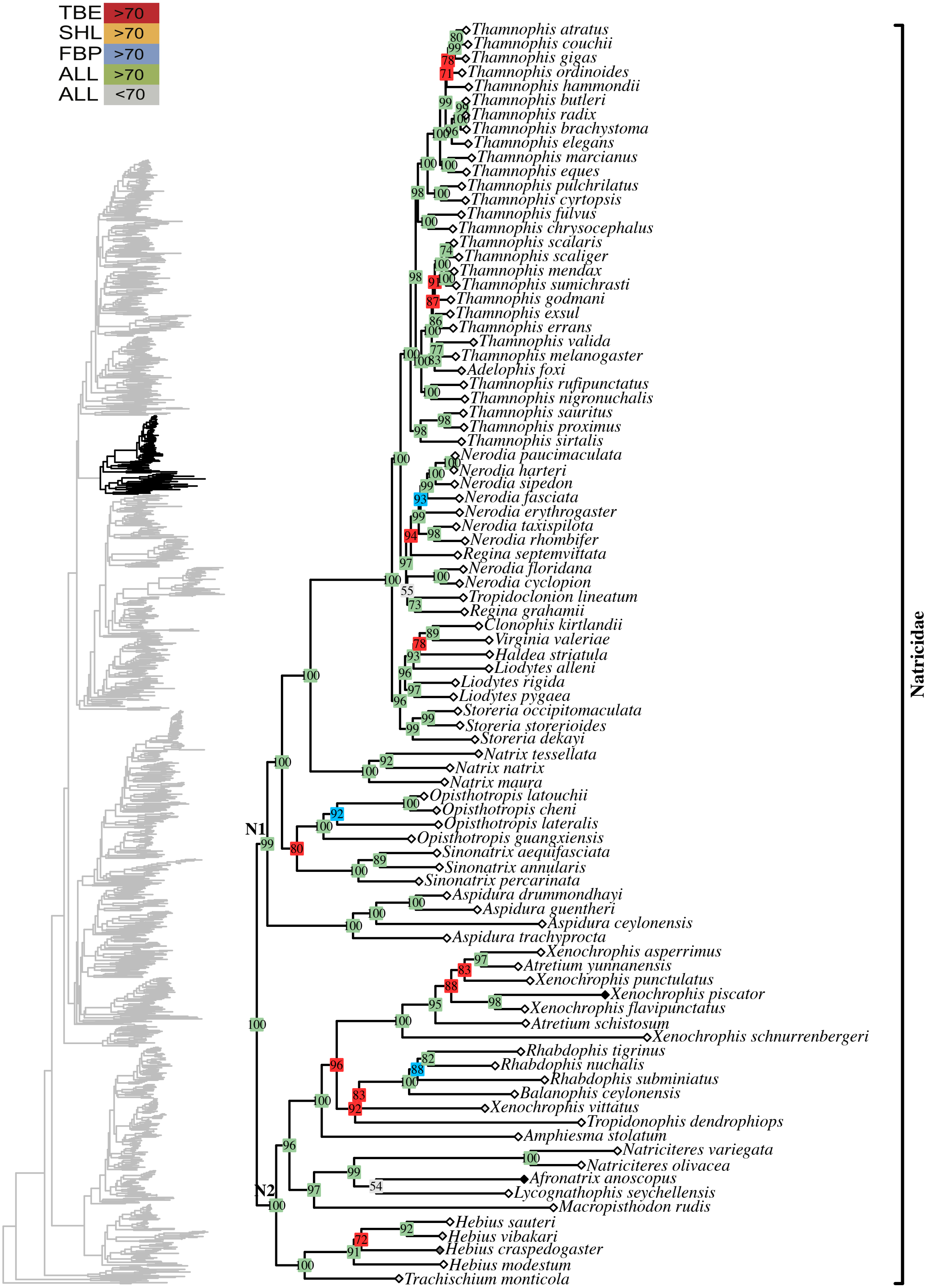
TBE >70
SHL >70
FBP >70
ALL >70
ALL <70
Thamnophis atratus
Thamnophis couchii
Thamnophis gigas
Thamnophis ordinoides
Thamnophis hammondii
Thamnophis butleri
Thamnophis radix
Thamnophis brachystoma
Thamnophis elegans
Thamnophis marcianus
Thamnophis eques
Thamnophis pulchrilatus
Thamnophis cyrtopsis
Thamnophis fulvus
Thamnophis chrysocephalus
Thamnophis scalaris
Thamnophis scaliger
Thamnophis mendax
Thamnophis sumichrasti
Thamnophis godmani
Thamnophis exsul
Thamnophis errans
Thamnophis valida
Thamnophis melanogaster
Adelophis foxi
Thamnophis rufipunctatus
Thamnophis nigronuchalis
Thamnophis sauritus
Thamnophis proximus
Thamnophis sirtalis
Nerodia paucimaculata
Nerodia harteri
Nerodia sipedon
Nerodia fasciata
Nerodia erythrogaster
Nerodia taxispilota
Nerodia rhombifer
Regina septemvittata
Nerodia floridana
Nerodia cyclopion
Tropidoclonion lineatum
Regina grahamii
Clonophis kirtlandii
Virginia valeriae
Haldea striatula
Liodytes alleni
Liodytes rigida
Liodytes pygaea
Storeria occipitomaculata
Storeria storerioides
Storeria dekayi
Natrix tessellata
Natrix natrix
Natrix maura
Opisthotropis latouchii
Opisthotropis cheni
Opisthotropis lateralis
Opisthotropis guangxiensis
Sinonatrix aequifasciata
Sinonatrix annularis
Sinonatrix percarinata
Aspidura drummondhayi
Aspidura guentheri
Aspidura ceylonensis
Aspidura trachyprocta
Xenochrophis asperrimus
Atretium yunnanensis
Xenochrophis punctulatus
Xenochrophis piscator
Xenochrophis flavipunctatus
Atretium schistosum
Xenochrophis schnurrenbergeri
Rhabdophis tigrinus
Rhabdophis nuchalis
Rhabdophis subminiatus
Balanophis ceylonensis
Xenochrophis vittatus
Tropidonophis dendrophiops
Amphiesma stolatum
Natriciteres variegata
Natriciteres olivacea
Afronatrix anoscopus
Lycognathophis seychellensis
Macropisthodon rudis
Hebius sauteri
Hebius vibakari
Hebius craspedogaster
Hebius modestum
Trachischium monticola
N1
N2
Natricidae

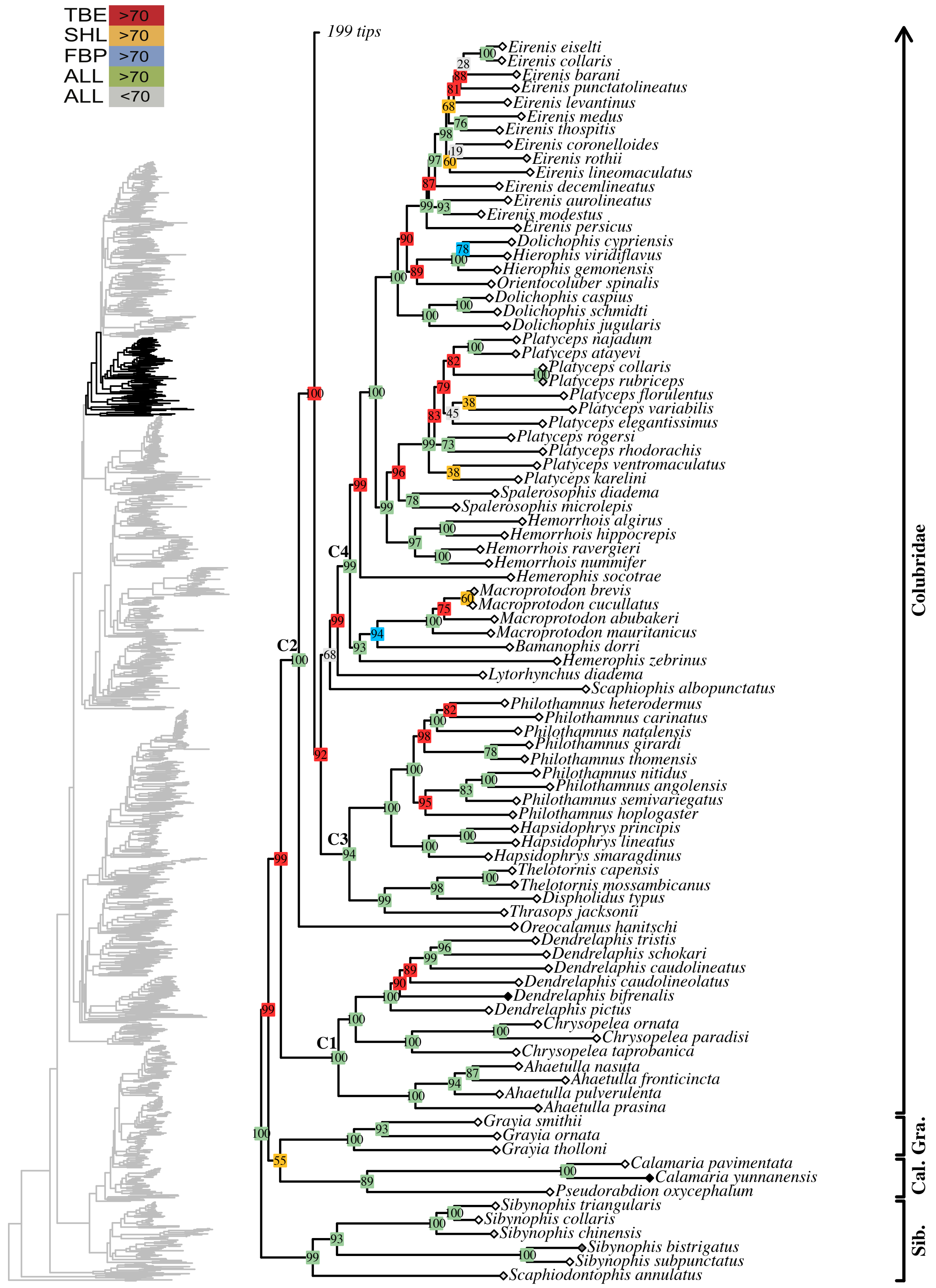
TBE >70
SHL >70
FBP >70
ALL >70
ALL <70
199 tips
Eirenis eiselti
Eirenis collaris
Eirenis barani
Eirenis punctatolineatus
Eirenis levantinus
Eirenis medus
Eirenis thospitis
Eirenis coronelloides
Eirenis rothii
Eirenis lineomaculatus
Eirenis decemlineatus
Eirenis aurolineatus
Eirenis modestus
Eirenis persicus
Dolichophis cypriensis
Hierophis viridiflavus
Hierophis gemonensis
Orientocoluber spinalis
Dolichophis caspius
Dolichophis schmidti
Dolichophis jugularis
Platyceps najadum
Platyceps atayevi
Platyceps collaris
Platyceps rubriceps
Platyceps florulentus
Platyceps variabilis
Platyceps elegantissimus
Platyceps rogersi
Platyceps rhodorachis
Platyceps ventromaculatus
Platyceps karelini
Spalerosophis diadema
Spalerosophis microlepis
Hemorrhois algirus
Hemorrhois hippocrepis
Hemorrhois ravergieri
Hemorrhois nummifer
Hemerophis socotrae
Macroprotodon brevis
Macroprotodon cucullatus
Macroprotodon abubakeri
Macroprotodon mauritanicus
Bamanophis dorri
Hemerophis zebrinus
Lytorhynchus diadema
Scaphiophis albopunctatus
Philothamnus heterodermus
Philothamnus carinatus
Philothamnus natalensis
Philothamnus girardi
Philothamnus thomensis
Philothamnus nitidus
Philothamnus angolensis
Philothamnus semivariegatus
Philothamnus hoplogaster
Hapsidophrys principis
Hapsidophrys lineatus
Hapsidophrys smaragdinus
Thelotornis capensis
Thelotornis mossambicanus
Dispholidus typus
Thrasops jacksonii
Oreocalamus hanitschi
Dendrelaphis tristis
Dendrelaphis schokari
Dendrelaphis caudolineatus
Dendrelaphis caudolineolatus
Dendrelaphis bifrenalis
Dendrelaphis pictus
Chrysopelea ornata
Chrysopelea paradisi
Chrysopelea taprobanica
Ahaetulla nasuta
Ahaetulla fronticincta
Ahaetulla pulverulenta
Ahaetulla prasina
Grayia smithii
Grayia ornata
Grayia tholloni
Calamaria pavimentata
Calamaria yunnanensis
Pseudorabdion oxycephalum
Sibynophis triangularis
Sibynophis collaris
Sibynophis chinensis
Sibynophis bistrigatus
Sibynophis subpunctatus
Scaphiodontophis annulatus
C1
C2
C3
C4
Colubridae
Gra.
Cal.
Sib.

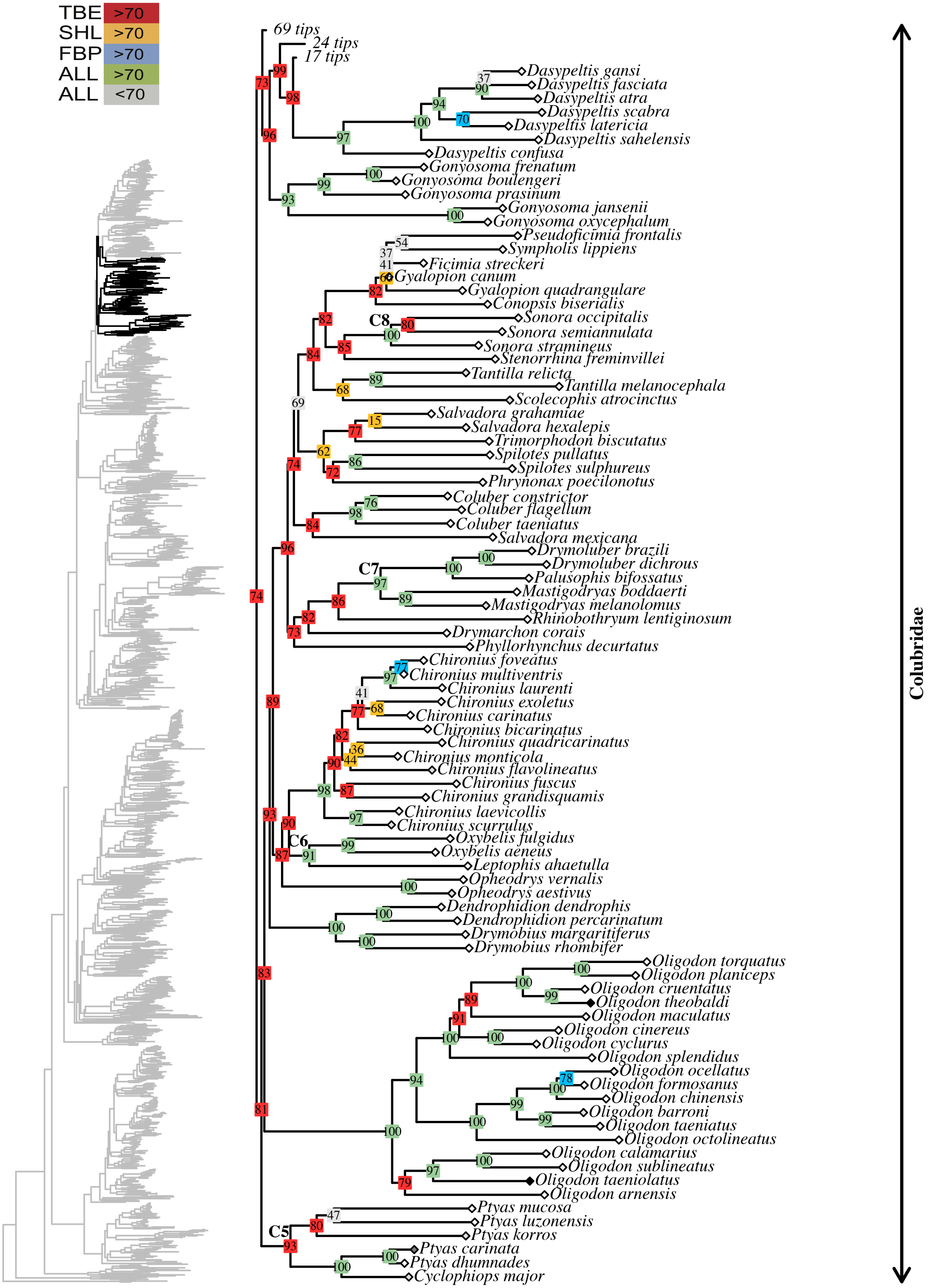

TBE >70
SHL >70
FBP >70
ALL >70
ALL <70
69 tips
24 tips
17 tips
Dasypeltis gansi
Dasypeltis fasciata
Dasypeltis atra
Dasypeltis scabra
Dasypeltis latericia
Dasypeltis sahelensis
Dasypeltis confusa
Gonyosoma frenatum
Gonyosoma boulengeri
Gonyosoma prasinum
Gonyosoma jansenii
Gonyosoma oxycephalum
Pseudoficimia frontalis
Sympholis lippiens
Ficimia streckeri
Gyalopion canum
Gyalopion quadrangulare
Conopsis biserialis
C8
Sonora occipitalis
Sonora semiannulata
Sonora stramineus
Stenorrhina freminvillei
Tantilla relicta
Tantilla melanocephala
Scolecophis atrocinctus
Salvadora grahamiae
Salvadora hexalepis
Trimorphodon biscutatus
Spilotes pullatus
Spilotes sulphureus
Phrynonax poecilonotus
Coluber constrictor
Coluber flagellum
Coluber taeniatus
Salvadora mexicana
C7
Drymoluber brazili
Drymoluber dichrous
Palusophis bifossatus
Mastigodryas boddaerti
Mastigodryas melanolomus
Rhinobothryum lentiginosum
Drymarchon corais
Phyllorhynchus decurtatus
Chironius foveatus
Chironius multiventris
Chironius laurenti
Chironius exoletus
Chironius carinatus
Chironius bicarinatus
Chironius quadricarinatus
Chironius monticola
Chironius flavolineatus
Chironius fuscus
Chironius grandisquamis
Chironius laevicollis
Chironius scurrulus
C6
Oxybelis fulgidus
Oxybelis aeneus
Leptophis ahaetulla
Opheodrys vernalis
Opheodrys aestivus
Dendrophidion dendrophis
Dendrophidion percarinatum
Drymobius margaritiferus
Drymobius rhombifer
Oligodon torquatus
Oligodon planiceps
Oligodon cruentatus
Oligodon theobaldi
Oligodon maculatus
Oligodon cinereus
Oligodon cyclurus
Oligodon splendidus
Oligodon ocellatus
Oligodon formosanus
Oligodon chinensis
Oligodon barroni
Oligodon taeniatus
Oligodon octolineatus
Oligodon calamarius
Oligodon sublineatus
Oligodon taeniolatus
Oligodon arnensis
C5
Ptyas mucosa
Ptyas luzonensis
Ptyas korros
Ptyas carinata
Ptyas dhumnades
Cyclophiops major
Colubridae

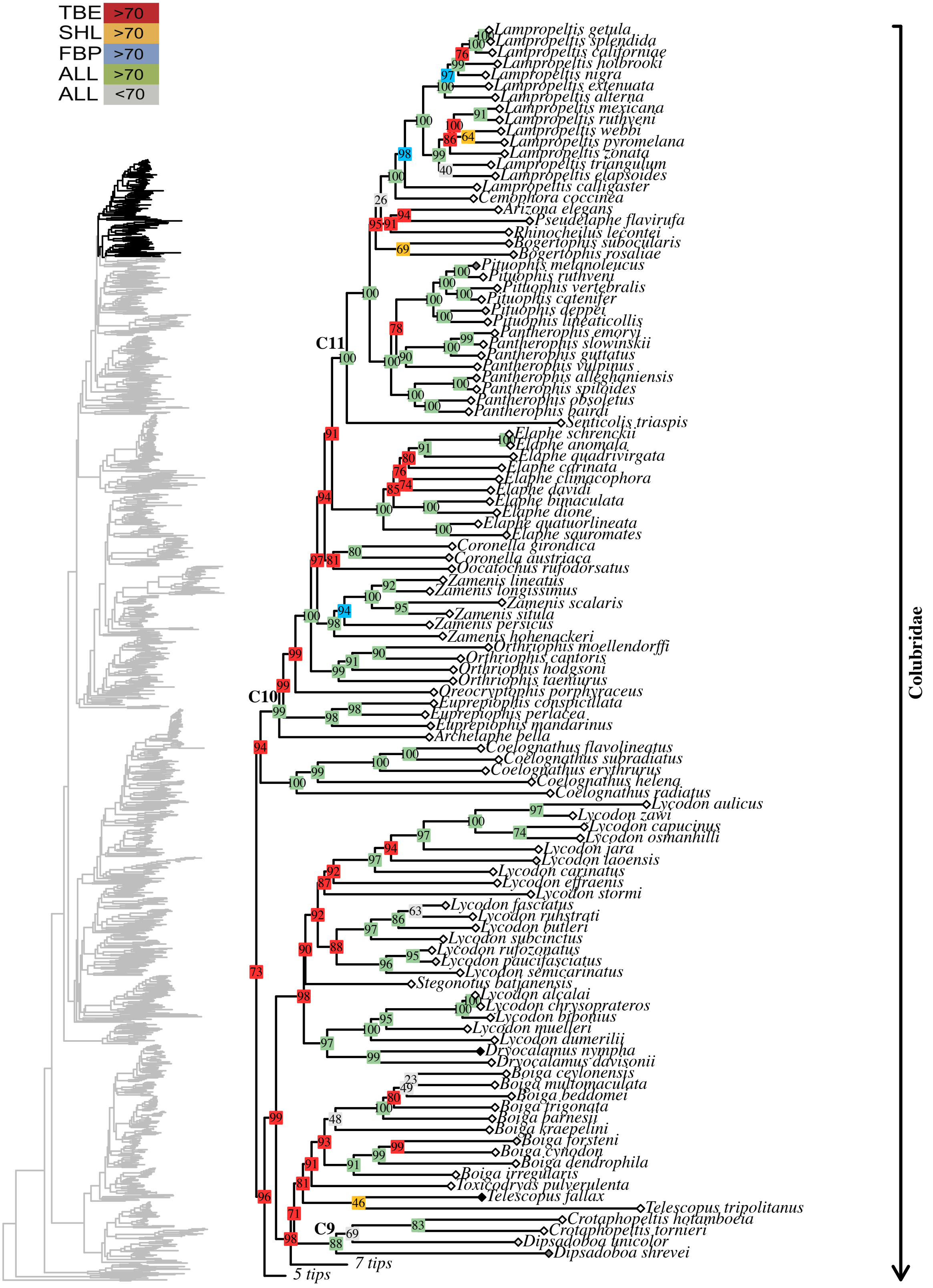
TBE >70
SHL >70
FBP >70
ALL >70
ALL <70
Colubridae
C11
C10
C9
Lampropeltis getula
Lampropeltis splendida
Lampropeltis californiae
Lampropeltis holbrooki
Lampropeltis nigra
Lampropeltis extenuata
Lampropeltis alterna
Lampropeltis mexicana
Lampropeltis ruthveni
Lampropeltis webbi
Lampropeltis pyromelana
Lampropeltis zonata
Lampropeltis triangulum
Lampropeltis elapsoides
Lampropeltis calligaster
Cemophora coccinea
Arizona elegans
Pseudelaphe flavirufa
Rhinocheilus lecontei
Bogertophis subocularis
Bogertophis rosaliae
Pituophis melanoleucus
Pituophis ruthveni
Pituophis vertebralis
Pituophis catenifer
Pituophis deppei
Pituophis lineaticollis
Pantherophis emoryi
Pantherophis slowinskii
Pantherophis guttatus
Pantherophis vulpinus
Pantherophis alleghaniensis
Pantherophis spiloides
Pantherophis obsoletus
Pantherophis bairdi
Senticolis triaspis
Elaphe schrenckii
Elaphe anomala
Elaphe quadrivirgata
Elaphe carinata
Elaphe climacophora
Elaphe davidi
Elaphe bimaculata
Elaphe dione
Elaphe quatuorlineata
Elaphe sauromates
Coronella girondica
Coronella austriaca
Oocatochus rufodorsatus
Zamenis lineatus
Zamenis longissimus
Zamenis scalaris
Zamenis situla
Zamenis persicus
Zamenis hohenackeri
Orthriophis moellendorffi
Orthriophis cantoris
Orthriophis hodgsoni
Orthriophis taeniurus
Oreocryptophis porphyraceus
Euprepiophis conspicillata
Euprepiophis perlacea
Euprepiophis mandarinus
Archelaphe bella
Coelognathus flavolineatus
Coelognathus subradiatus
Coelognathus erythrurus
Coelognathus helena
Coelognathus radiatus
Lycodon aulicus
Lycodon zawi
Lycodon capucinus
Lycodon osmanhilli
Lycodon jara
Lycodon laoensis
Lycodon carinatus
Lycodon effraenis
Lycodon stormi
Lycodon fasciatus
Lycodon ruhstrati
Lycodon butleri
Lycodon subcinctus
Lycodon rufozonatus
Lycodon paucifasciatus
Lycodon semicarinatus
Stegonotus batjanensis
Lycodon alcalai
Lycodon chrysoprateros
Lycodon bibonius
Lycodon muelleri
Lycodon dumerilii
Dryocalamus nympha
Dryocalamus davisonii
Boiga ceylonensis
Boiga multomaculata
Boiga beddomei
Boiga trigonata
Boiga barnesii
Boiga kraepelini
Boiga forsteni
Boiga cynodon
Boiga dendrophila
Boiga irregularis
Toxicodryas pulverulenta
Telescopus fallax
Telescopus tripolitanus
Crotaphopeltis hotamboeia
Crotaphopeltis tornieri
Dipsadoboa unicolor
Dipsadoboa shrevei
7 tips
5 tips
