# Supplementary figures and images for: Large-scale molecular phylogeny, morphology, divergence-time estimation, and the fossil record of advanced caenophidian snakes (Squamata: Serpentes)
Source: PLoS One. 2019 May 10;14(5):e0216148. doi: 10.1371/journal.pone.0216148 (PMC6512042; doi:10.1371/journal.pone.0216148)

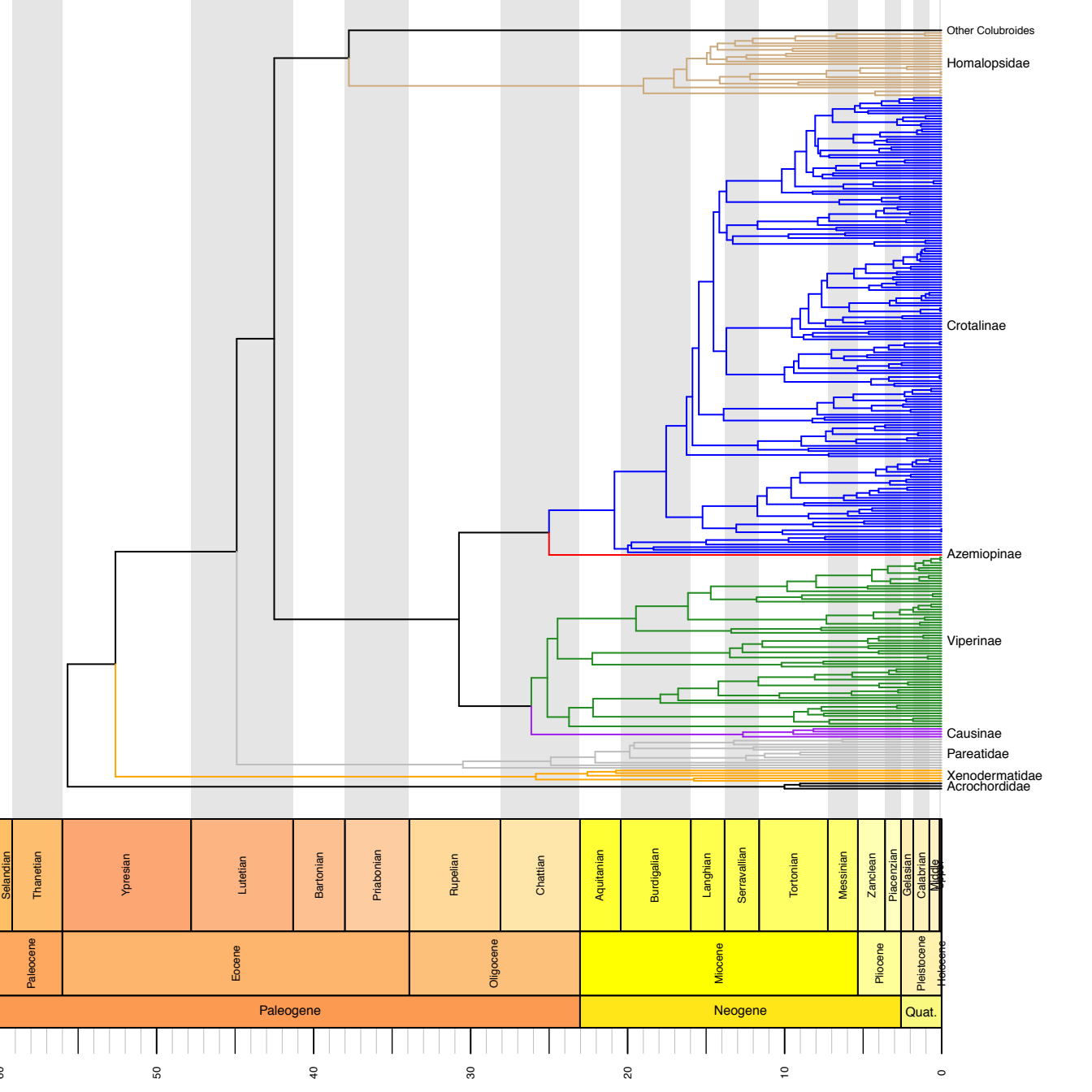

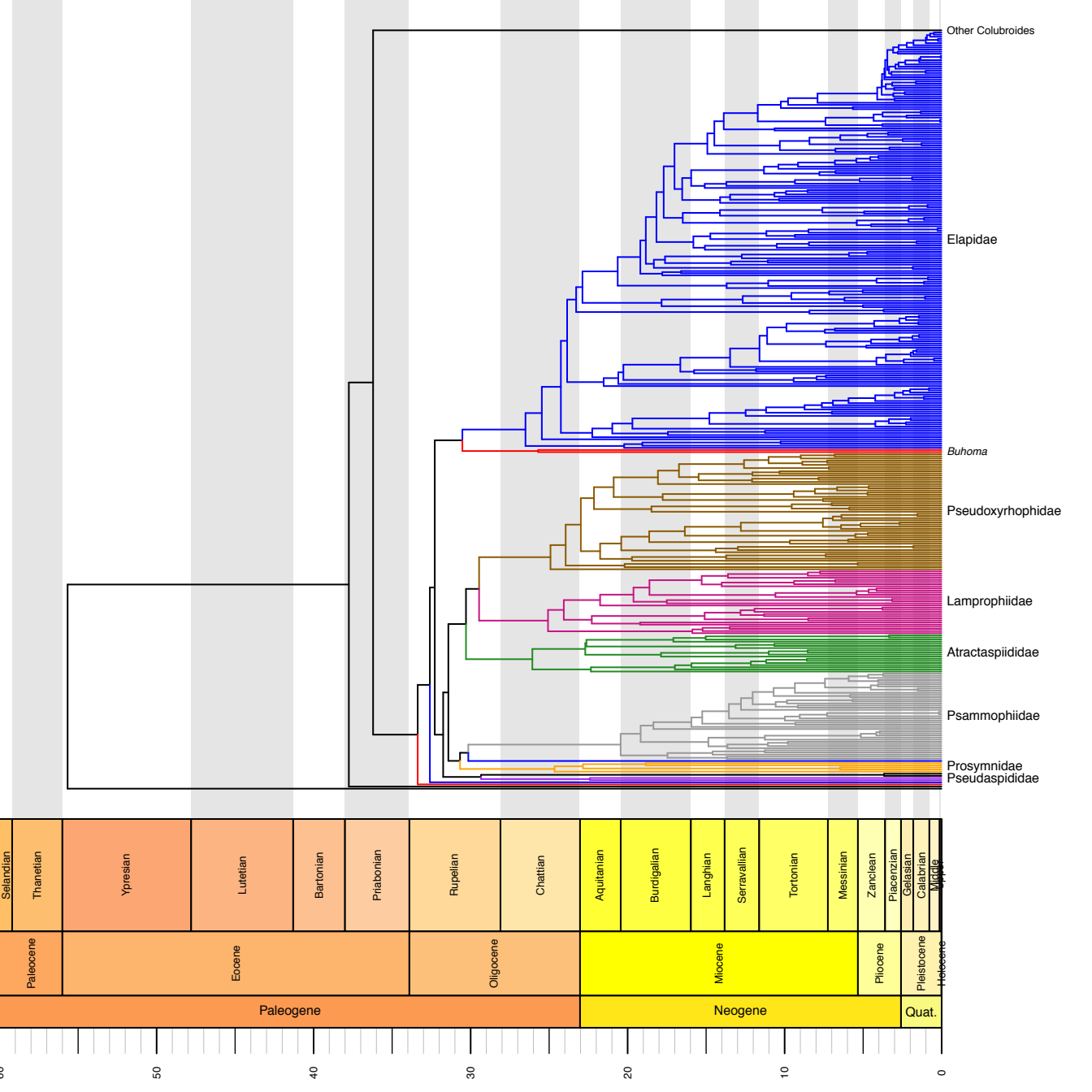

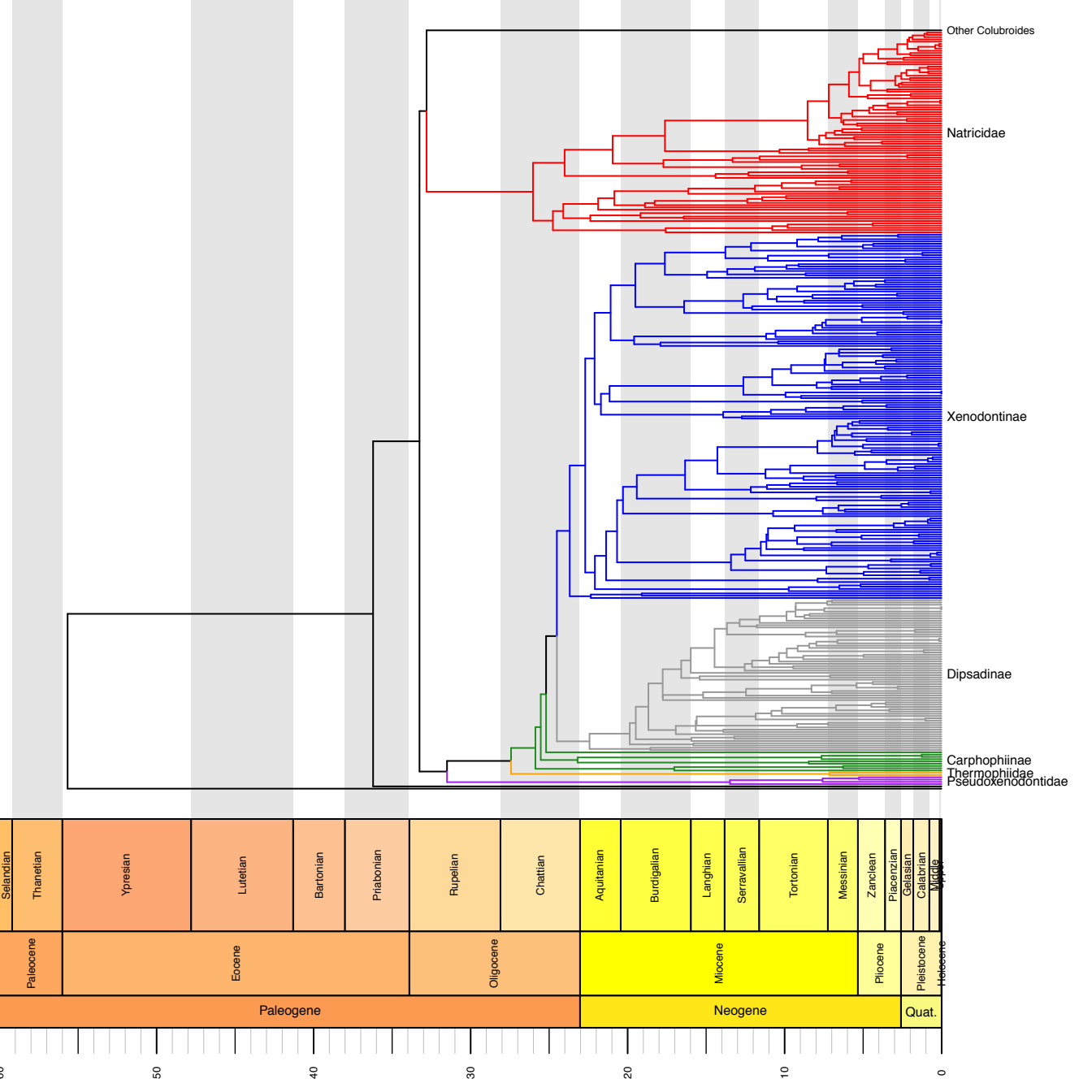

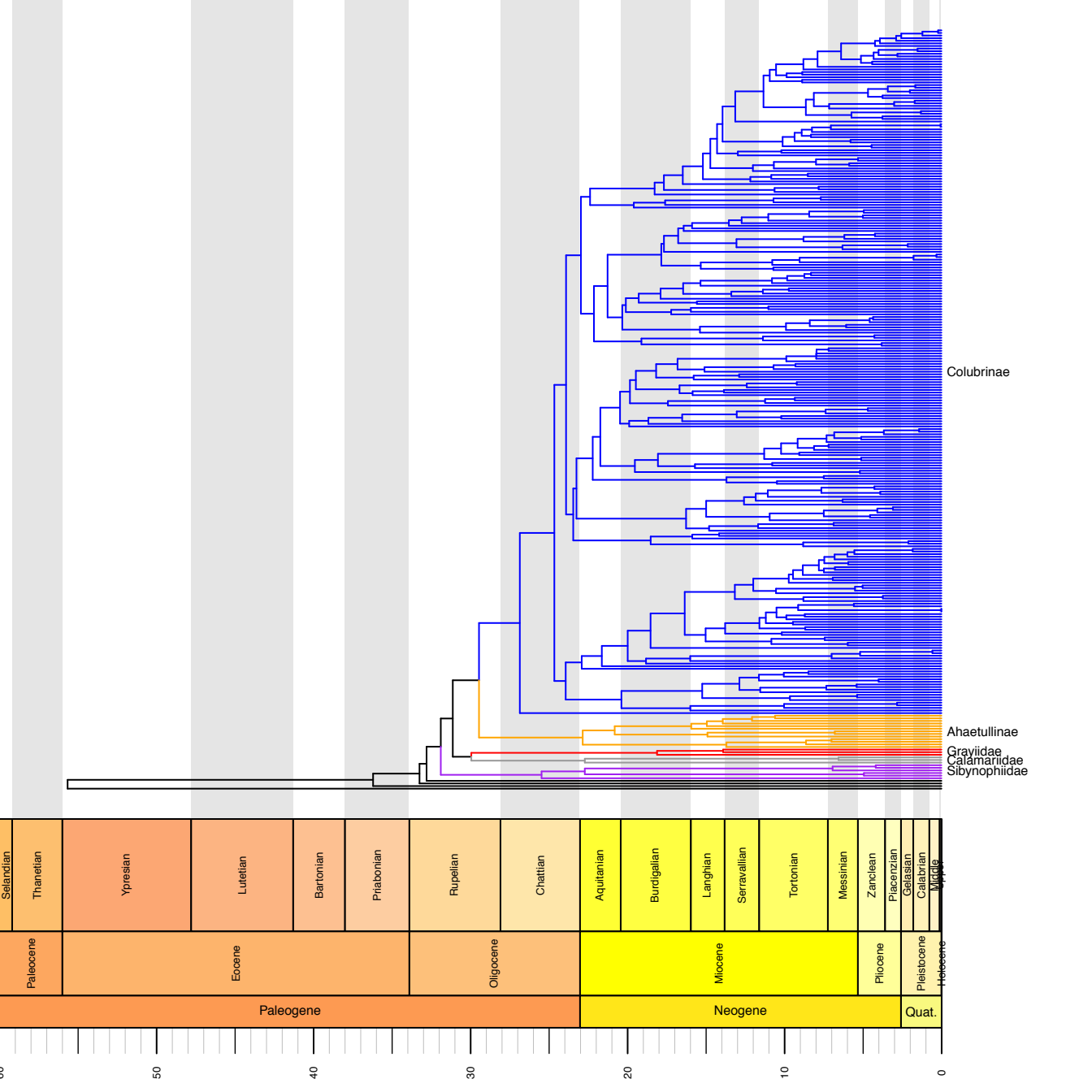

Supplement: S4 Fig — Zoomed, large-scale calibrated tree resulting from the treePL analysis showing the pattern of cladogenic events through time. (PDF) [file pone.0216148.s014.pdf]
