# Supplementary material for: Large-scale molecular phylogeny, morphology, divergence-time estimation, and the fossil record of advanced caenophidian snakes (Squamata: Serpentes)
Source: PLoS One. 2019 May 10;14(5):e0216148. doi: 10.1371/journal.pone.0216148 (PMC6512042; doi:10.1371/journal.pone.0216148)

S1 Appendix

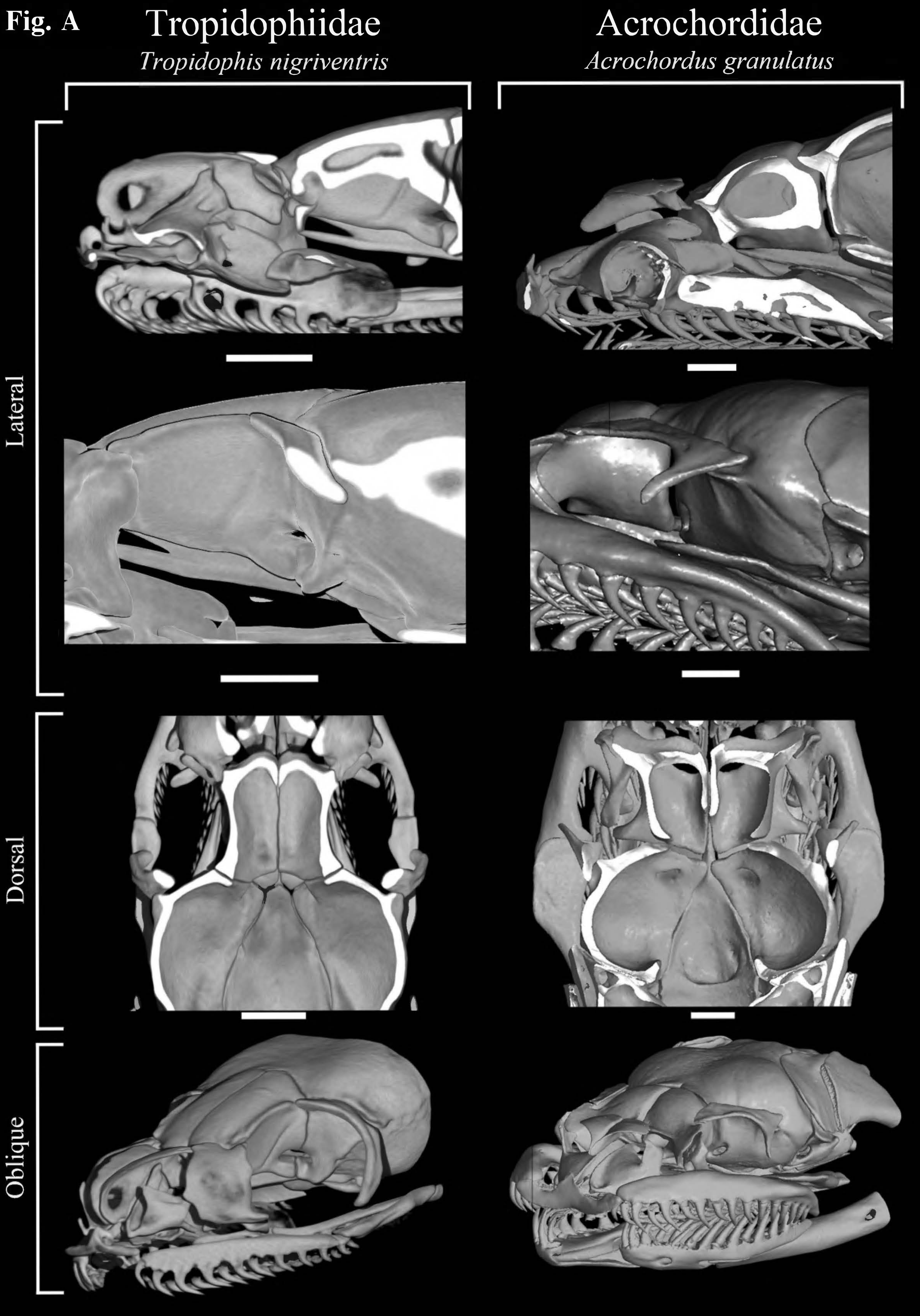

Fig. A
Tropidophiidae
Tropidophis nigriventris
Acrochordidae
Acrochordus granulatus
Lateral
Dorsal
Oblique

Fig. B

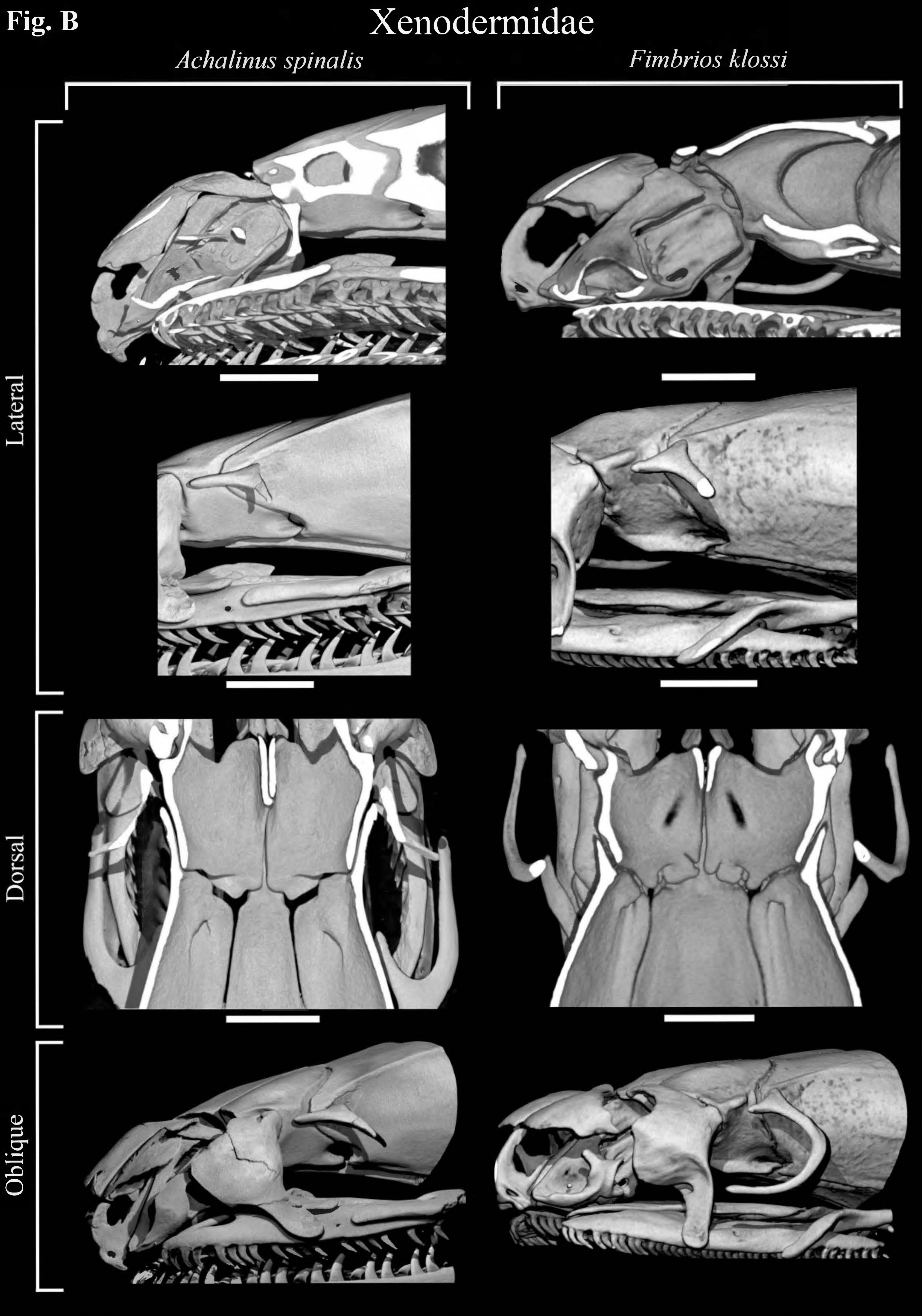
Xenodermidae
Achalinus spinalis
Fimbrios klossi
Lateral
Dorsal
Oblique

Fig. C

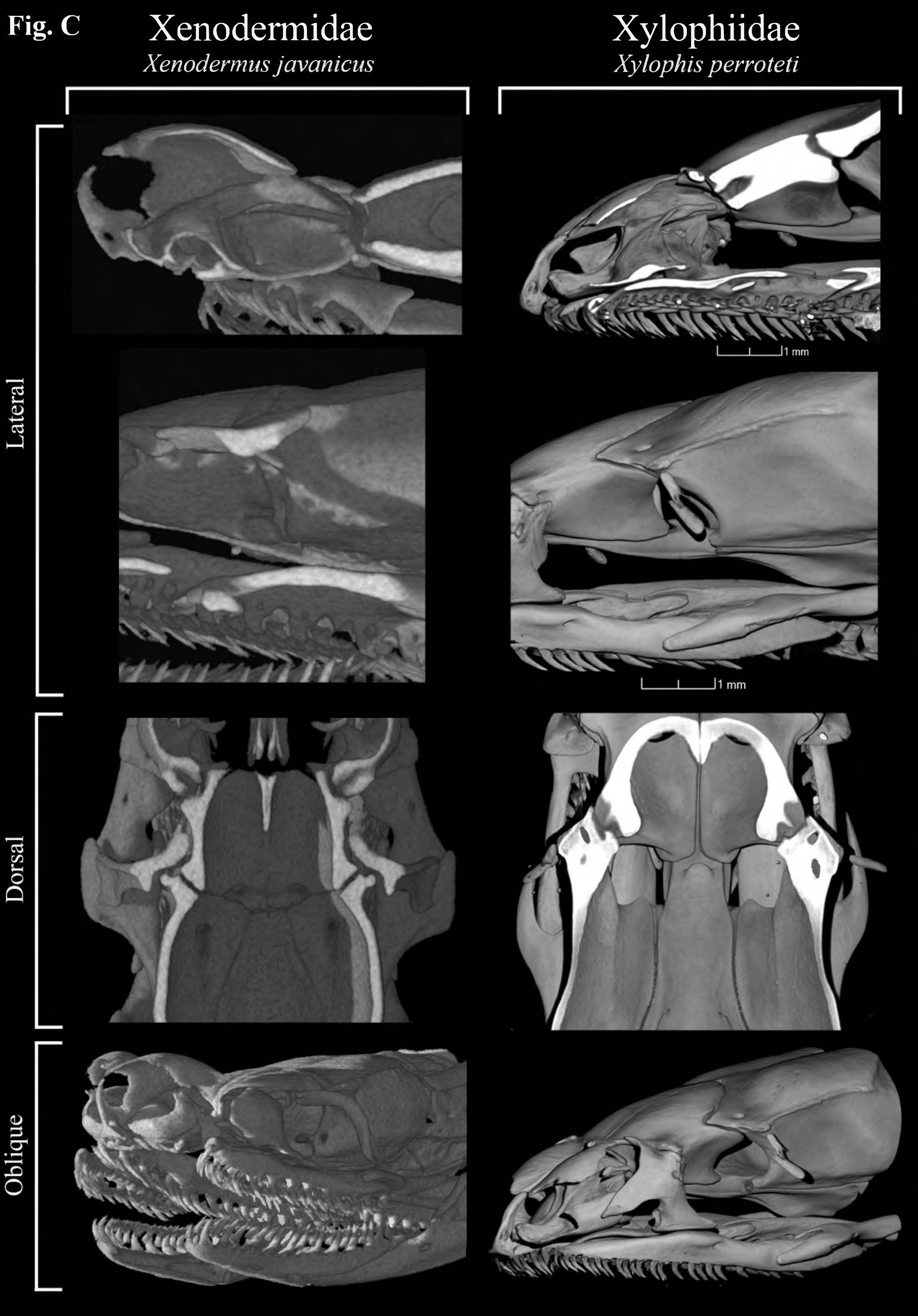

Xenodermidae
Xenodermus javanicus
Xylophiidae
Xylophis perroteti
Lateral
Dorsal
Oblique
1 mm
1 mm

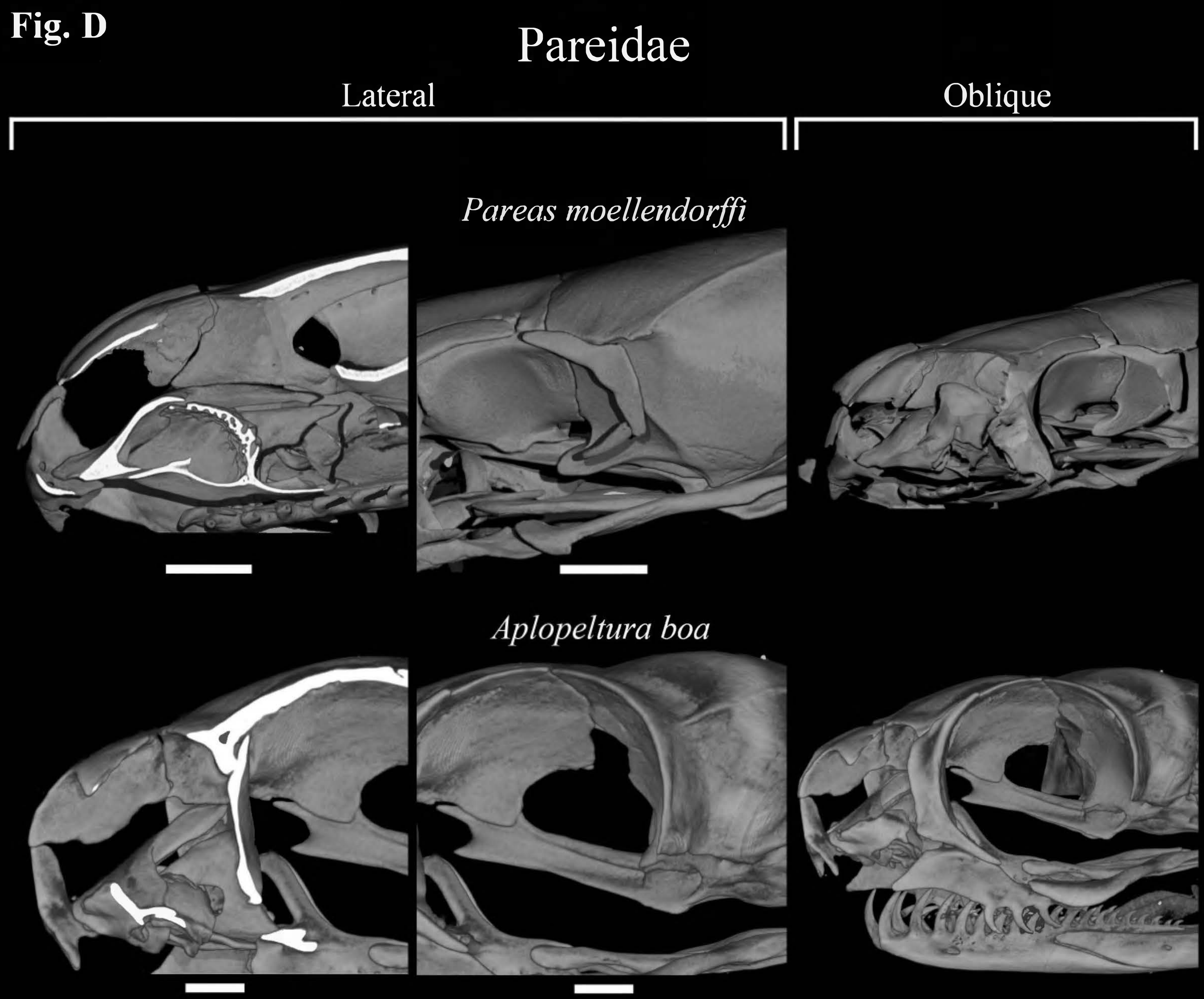
Fig. D
Pareidae
Lateral
Oblique
Pareas moellendorffi
Aplopeltura boa

Fig. E

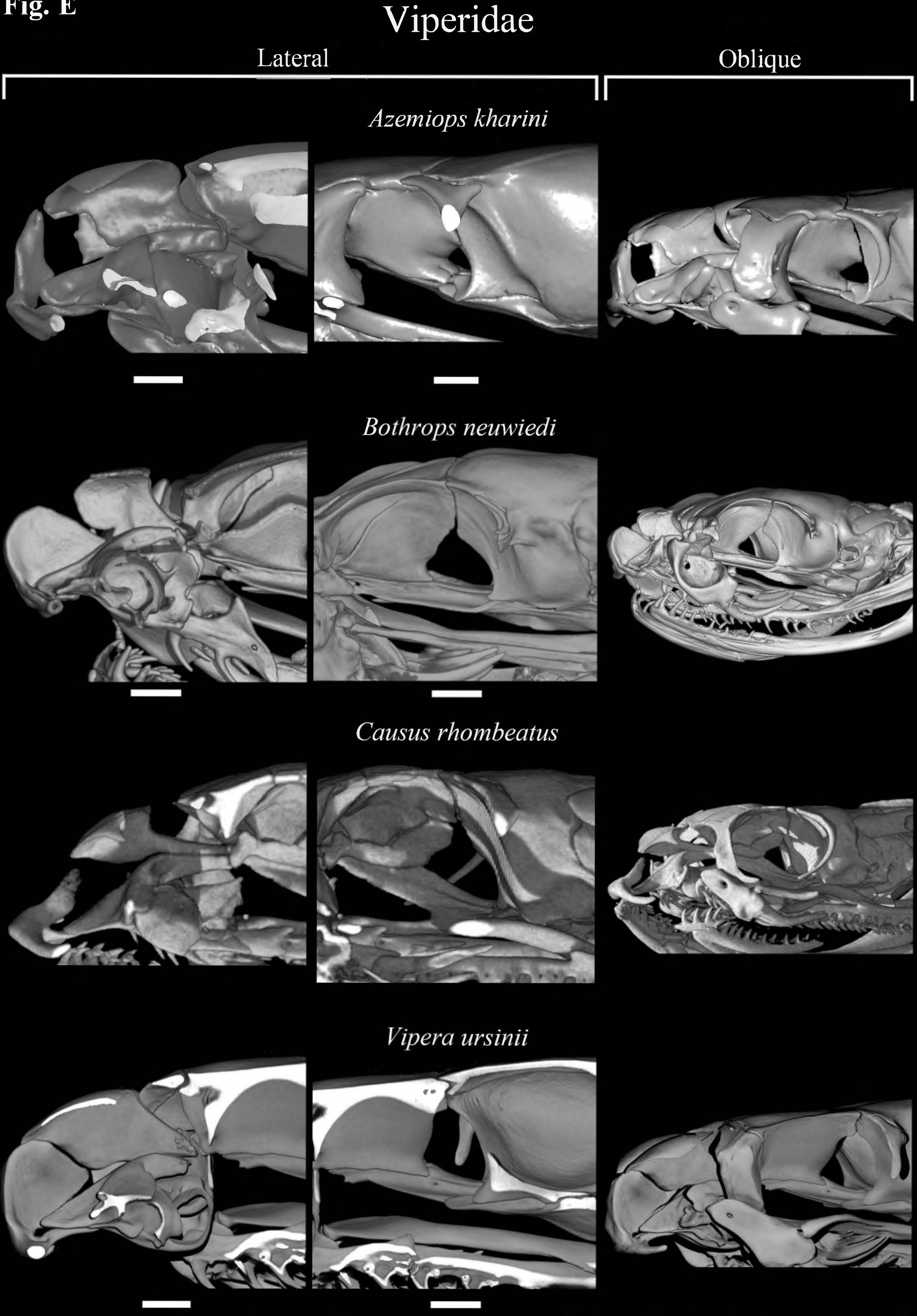
Viperidae
Lateral
Oblique
Azemiops kharini
Bothrops neuwiedi
Causus rhombeatus
Vipera ursinii

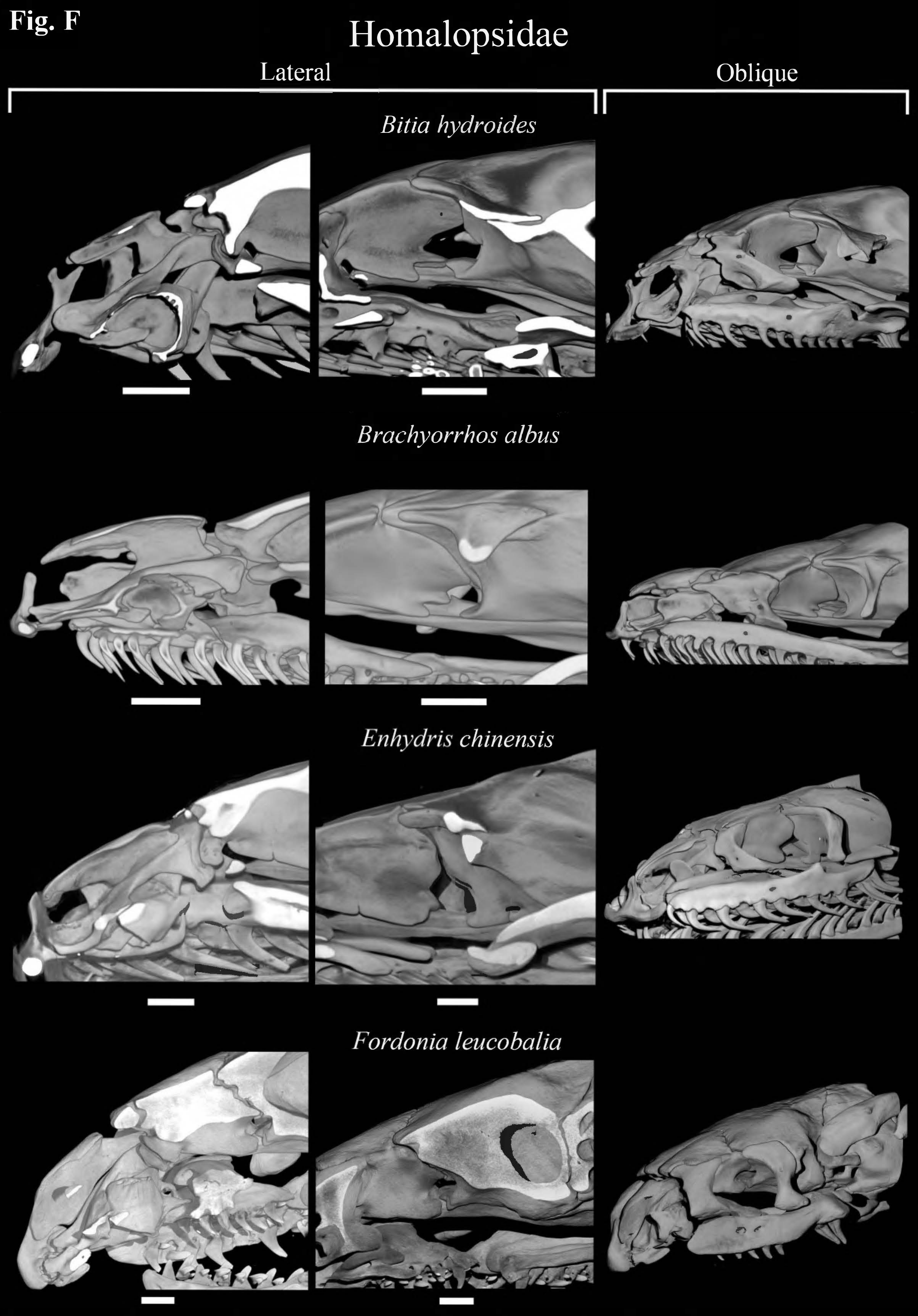
Fig. F
Homalopsidae
Lateral
Oblique
Bitia hydroides
Brachyorrhos albus
Enhydris chinensis
Fordonia leucobalia

Fig. G

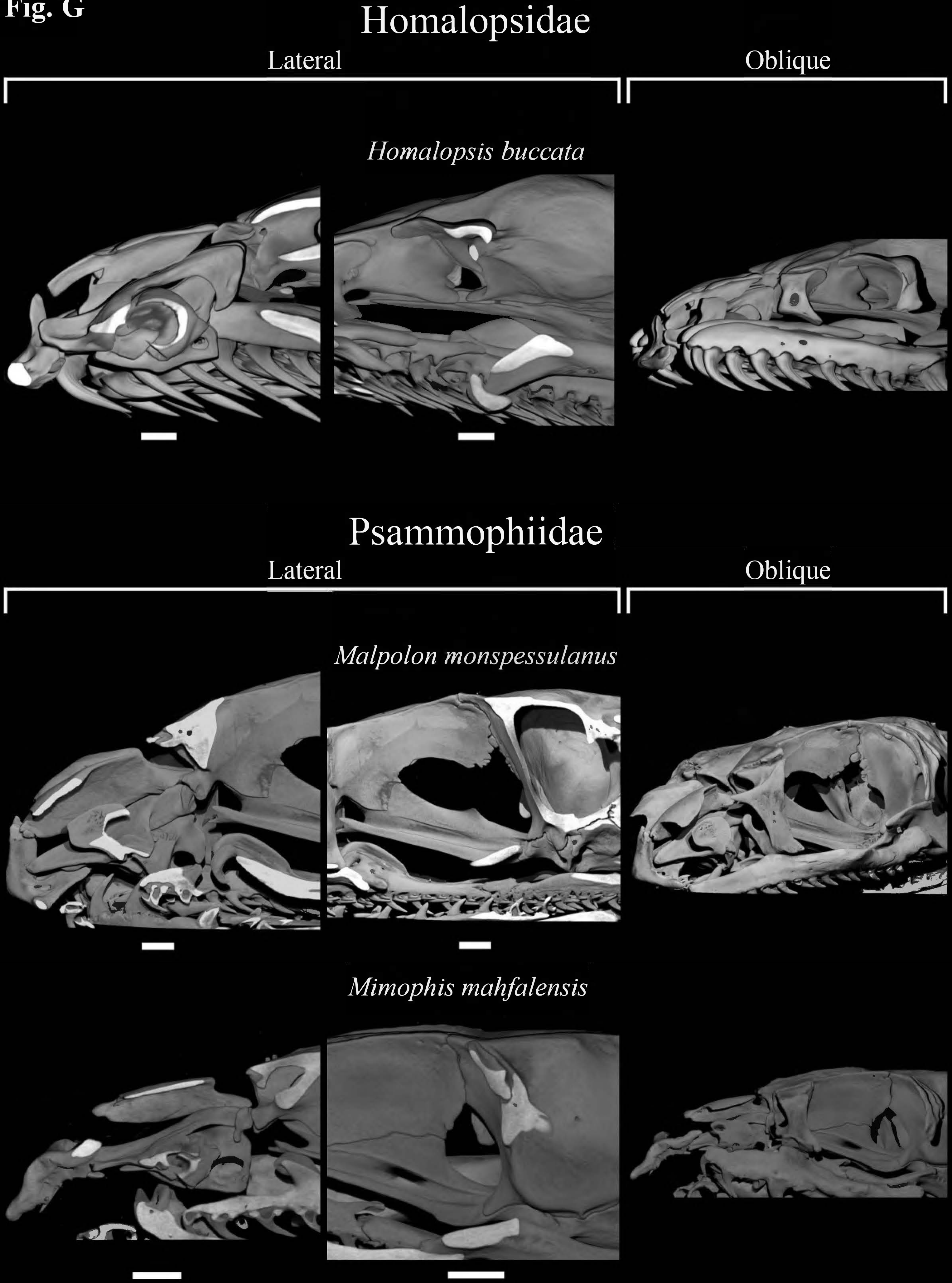
Homalopsidae
Lateral
Oblique
Homalopsis buccata
Psammophiidae
Lateral
Oblique
Malpolon monspessulanus
Mimophis mahfalensis

Fig. H

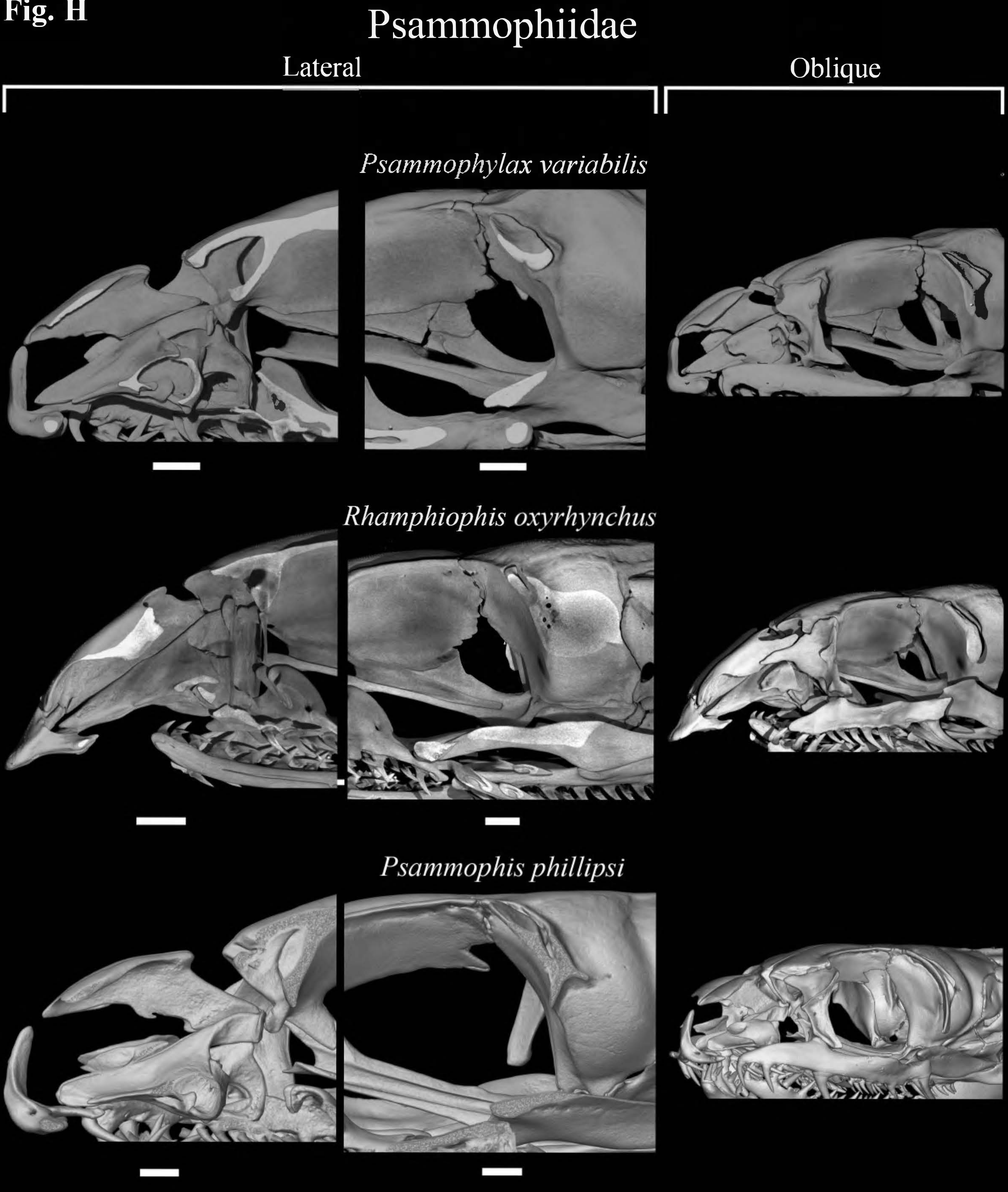
Psammophiidae
Lateral
Oblique
Psammophylax variabilis
Rhamphiophis oxyrhynchus
Psammophis phillipsi

Fig. I

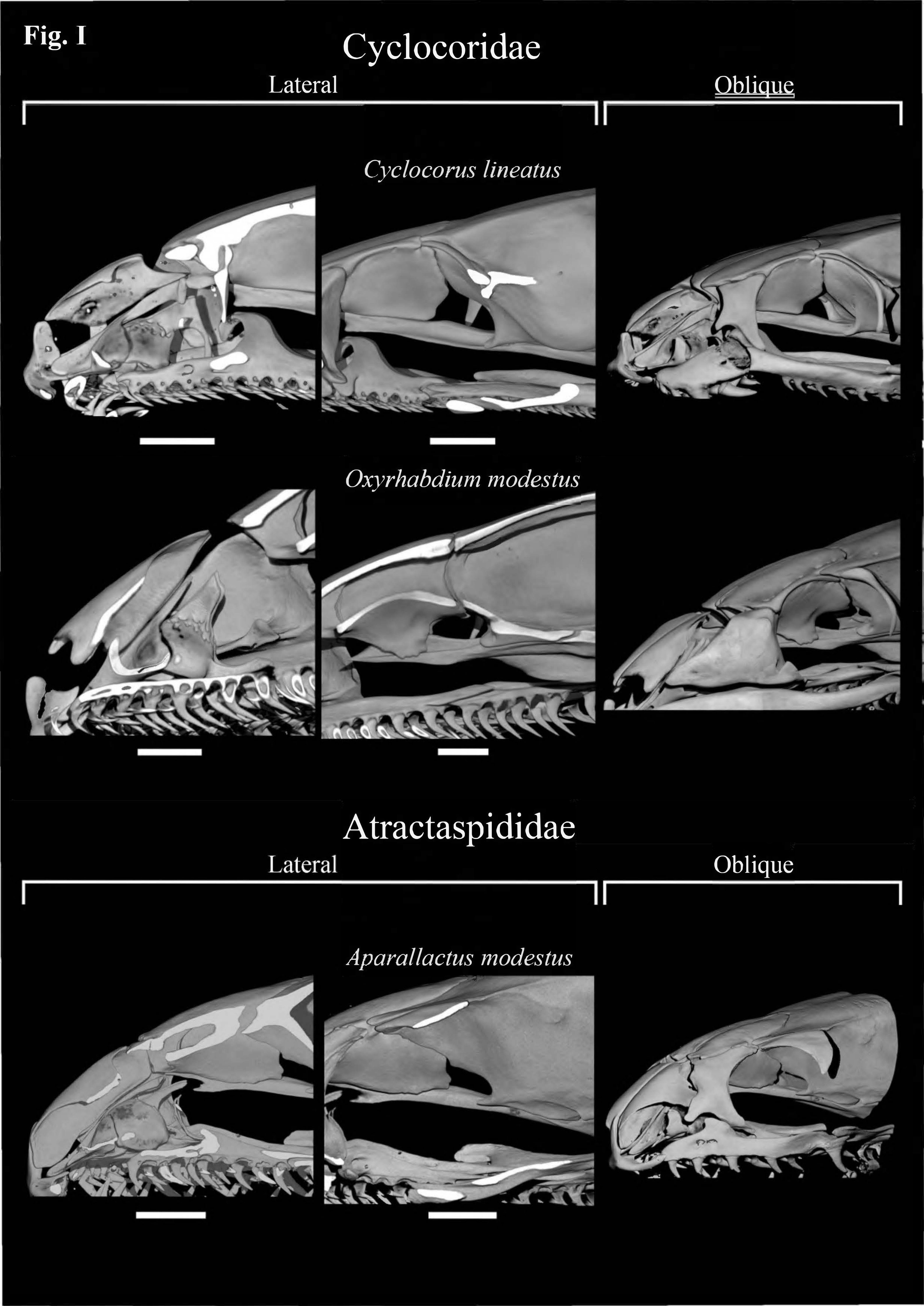
Cyclocoridae
Lateral
Oblique
Cyclocorus lineatus
Oxyrhabdium modestus
Atractaspididae
Lateral
Oblique
Aparallactus modestus

Fig. J

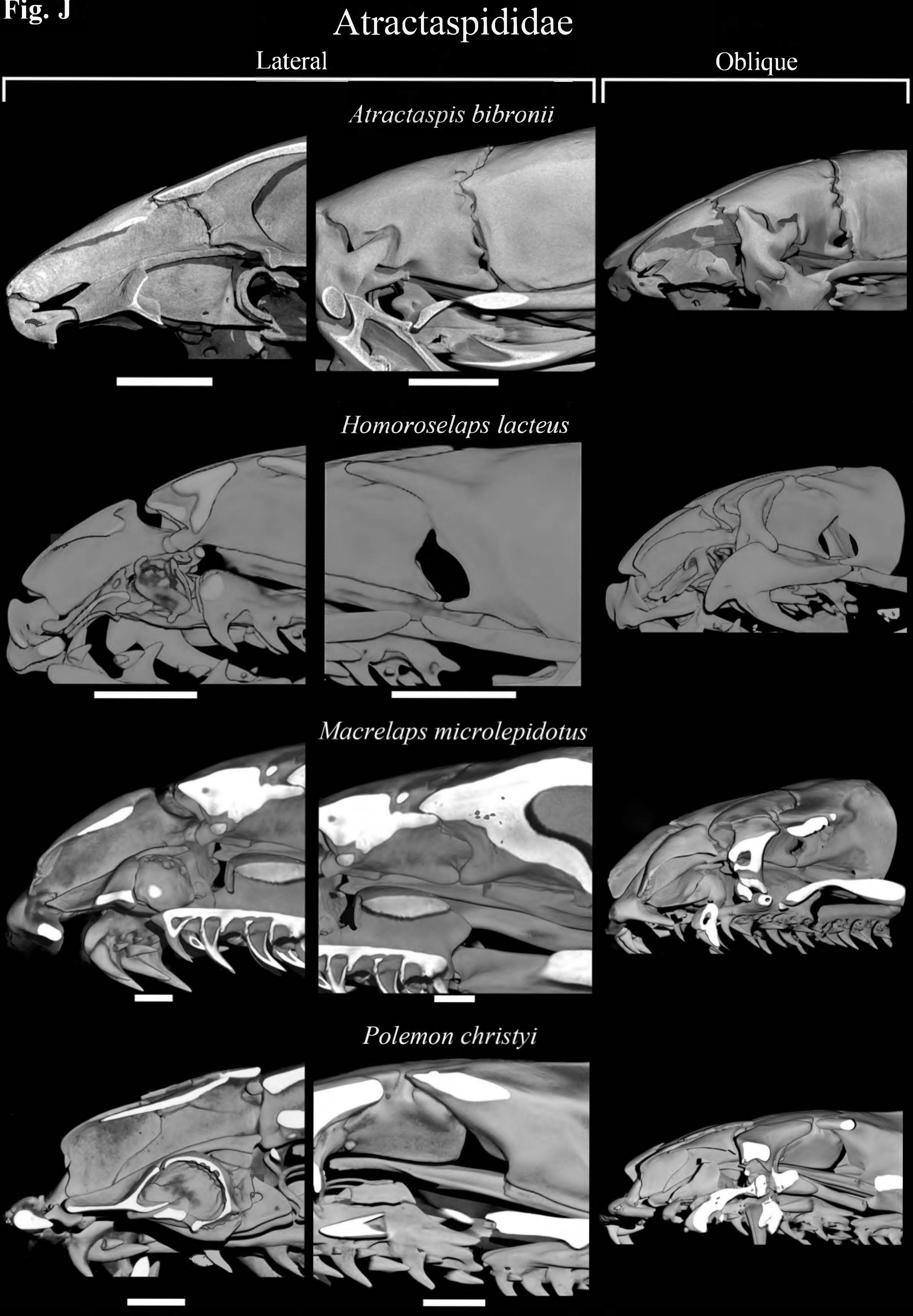
Atractaspididae
Lateral
Oblique
Atractaspis bibronii
Homoroselaps lacteus
Macrelaps microlepidotus
Polemon christyi

Fig. K

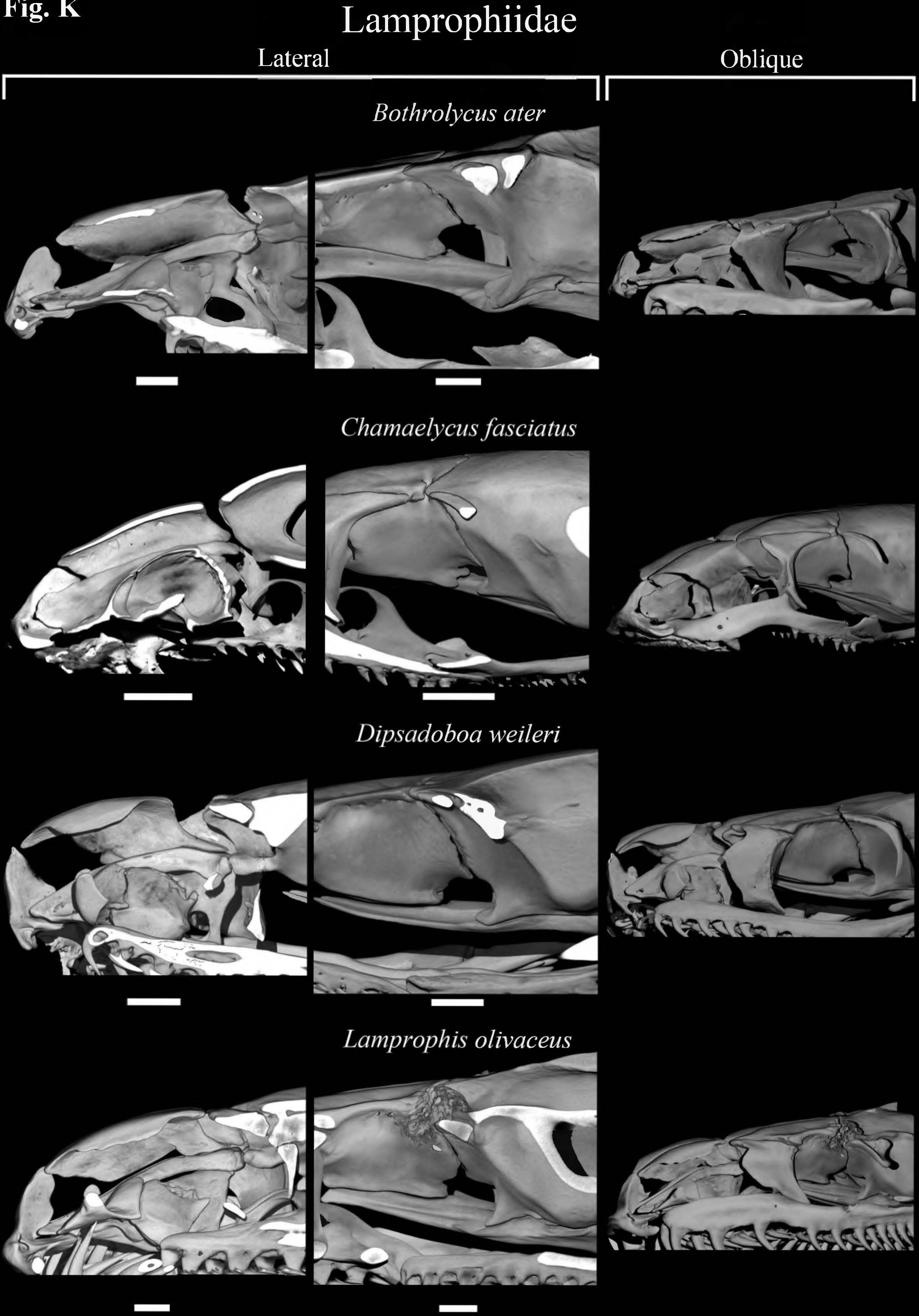
Lamprophiidae
Lateral
Oblique
Bothrolycus ater
Chamaelycus fasciatus
Dipsadoboa weileri
Lamprophis olivaceus

Fig. L

Lamprophiidae

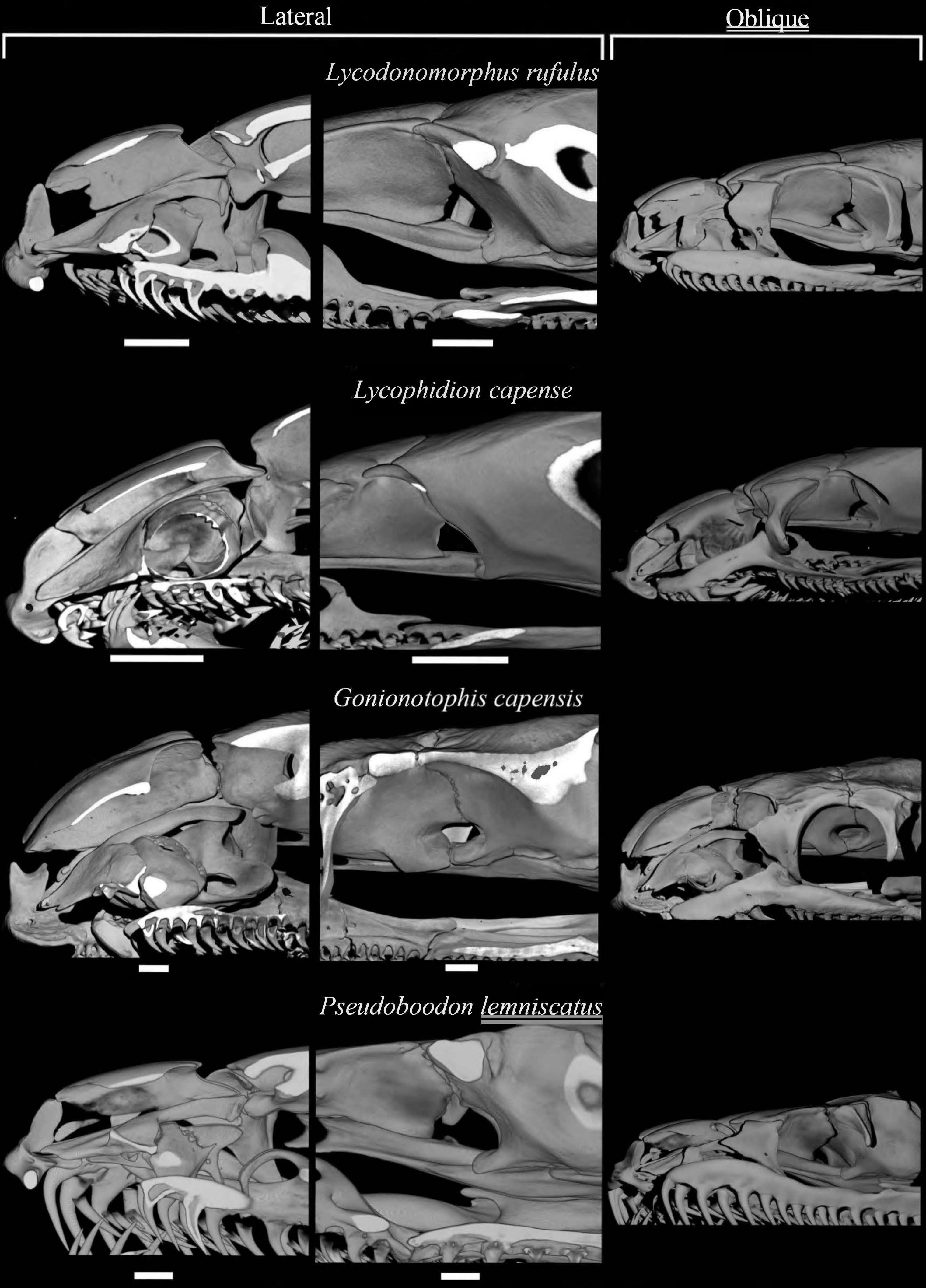

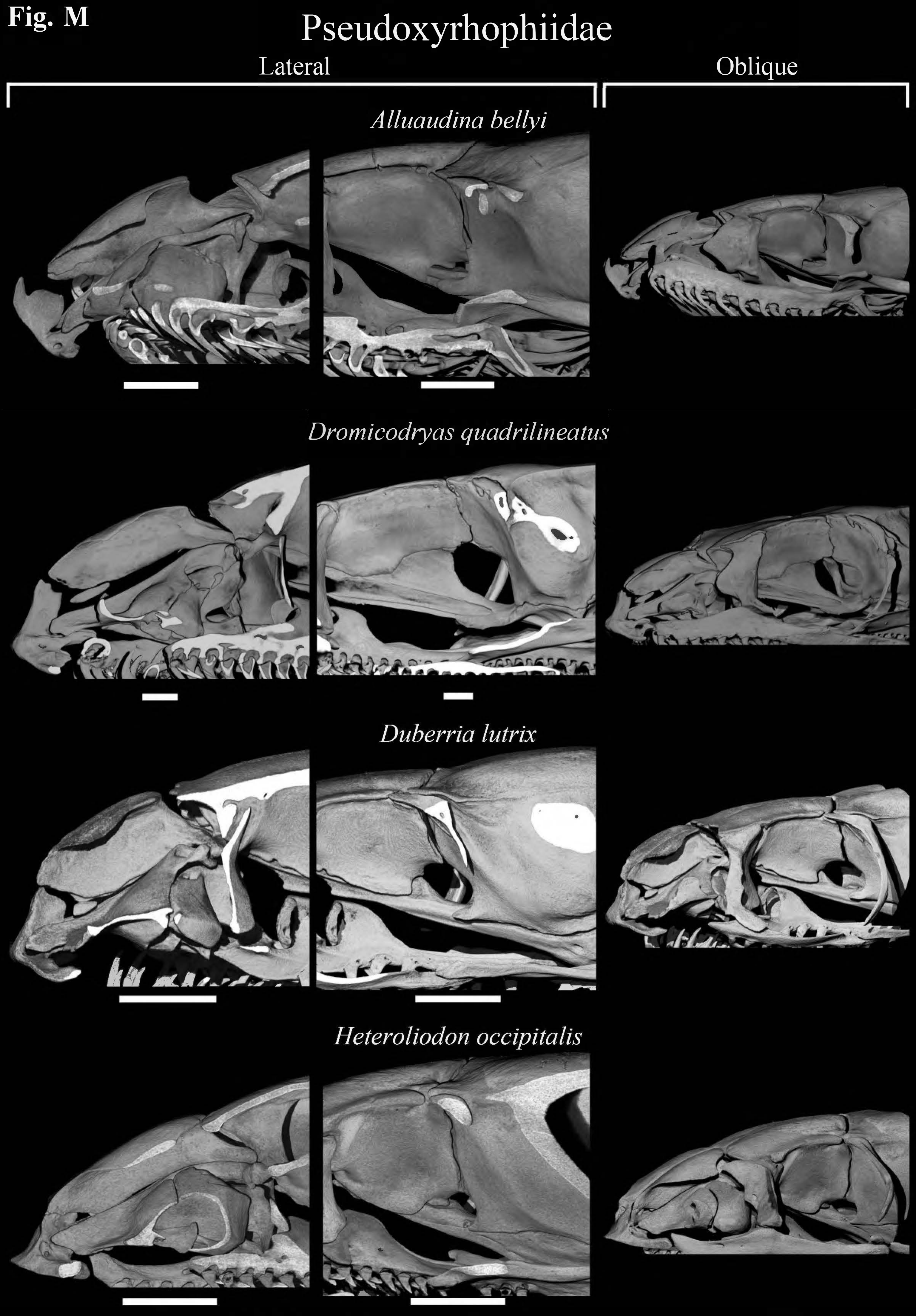
Fig. M
Pseudoxyrhophiidae
Lateral
Oblique
Alluaudina bellyi
Dromicodryas quadrilineatus
Duberria lutrix
Heteroliodon occipitalis

Fig. N

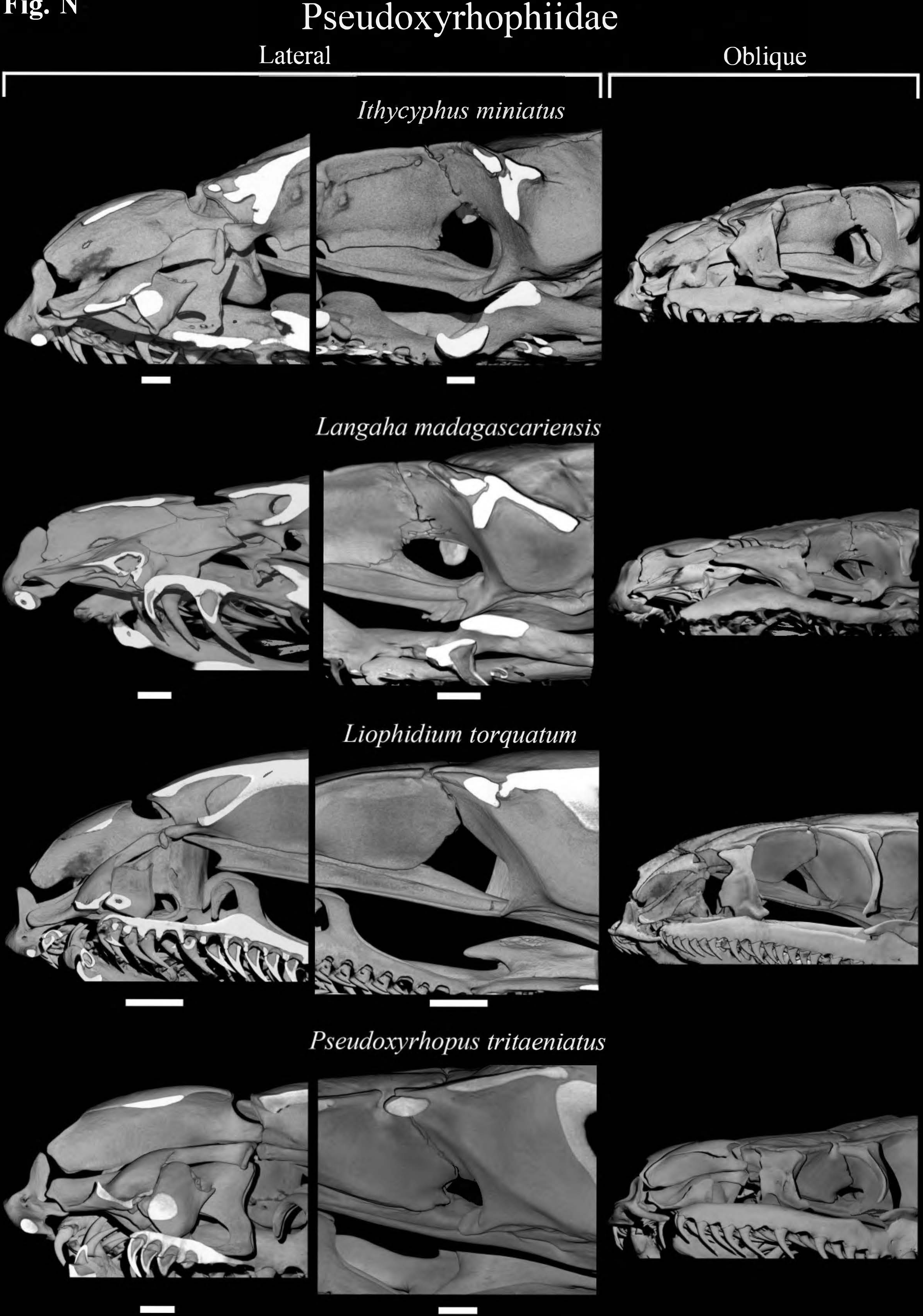
Pseudoxyrhophiidae
Lateral
Oblique
Ithycyphus miniatus
Langaha madagascariensis
Liophidium torquatum
Pseudoxyrhopus tritaeniatus

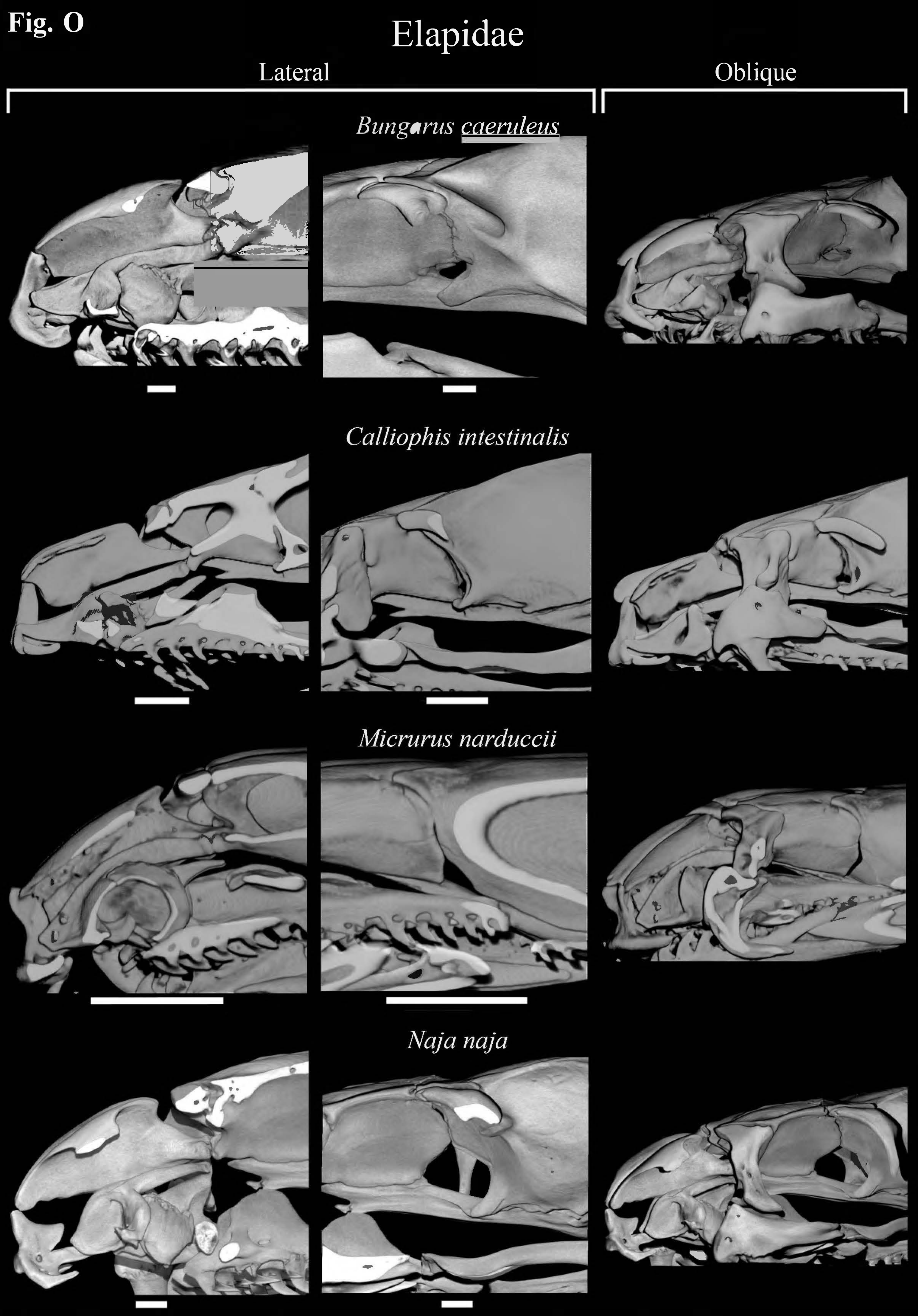
Fig. O
Elapidae
Lateral
Oblique
Bungarus caeruleus
Calliophis intestinalis
Micrurus narduccii
Naja naja

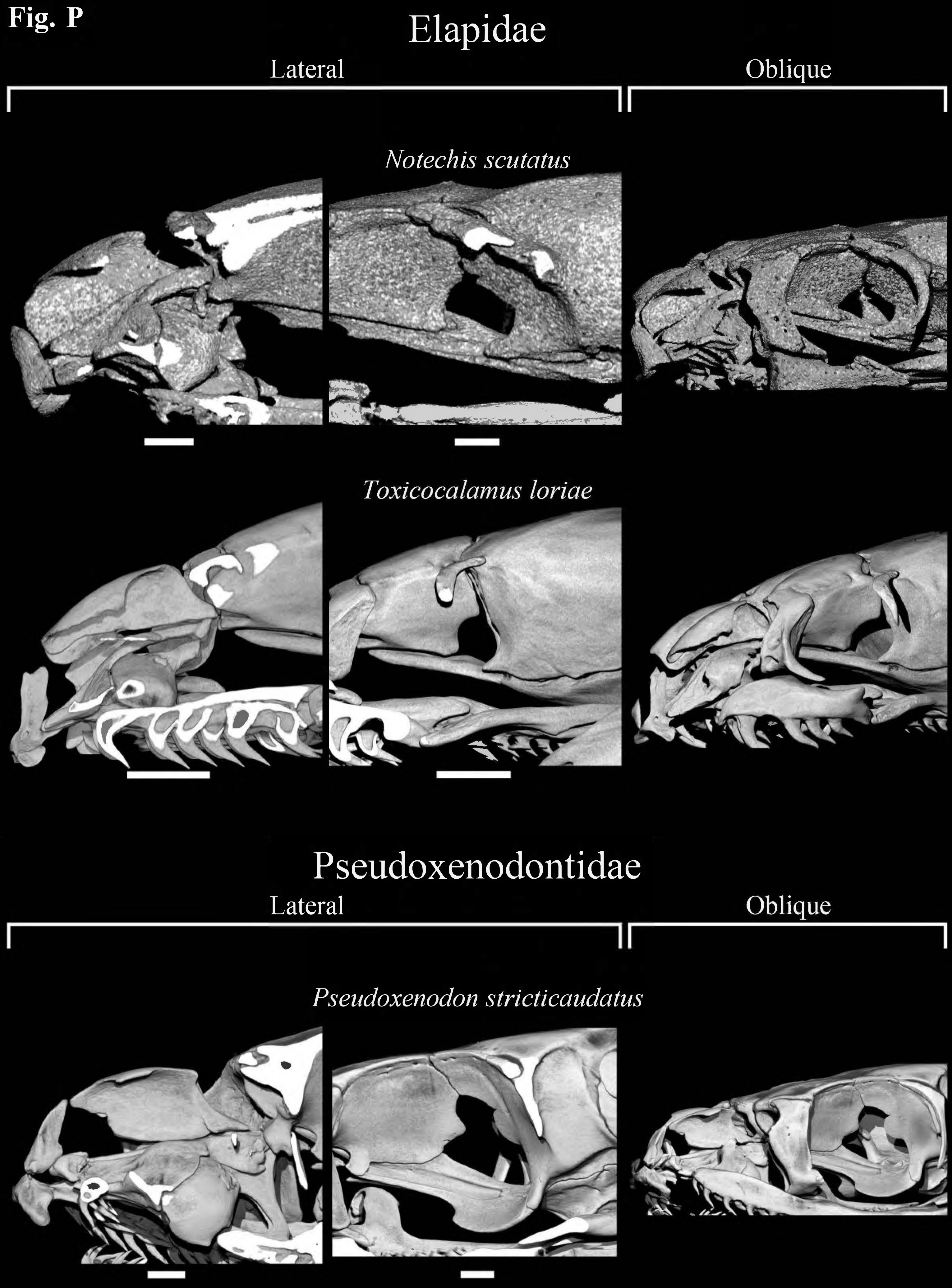
Fig. P
Elapidae
Lateral
Oblique
Notechis scutatus
Toxicocalamus loriae
Pseudoxenodontidae
Lateral
Oblique
Pseudoxenodon stricticaudatus

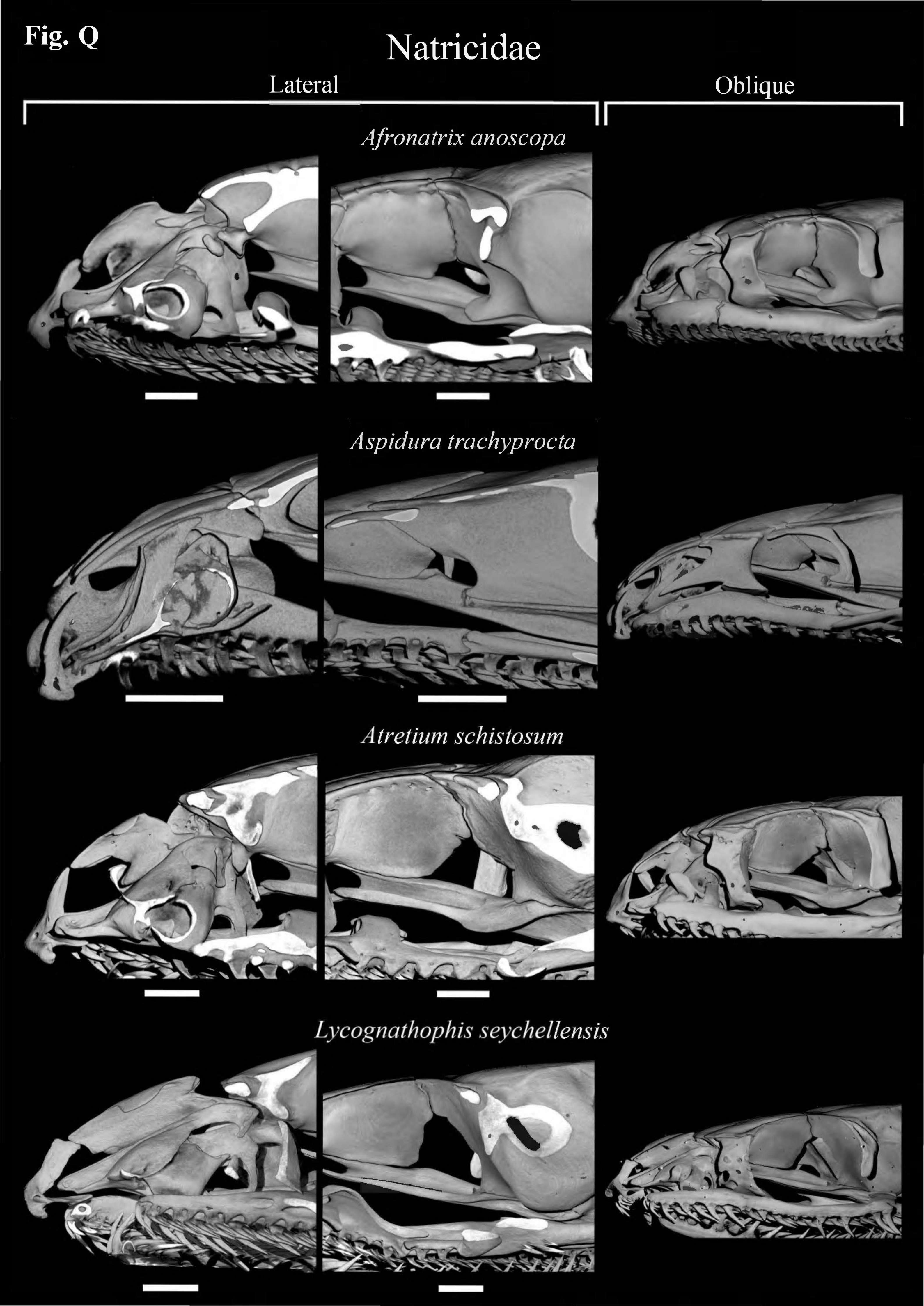
Fig. Q
Natricidae
Lateral
Oblique
Afronatrix anoscopa
Aspidura trachyprocta
Atretium schistosum
Lycognathophis seychellensis

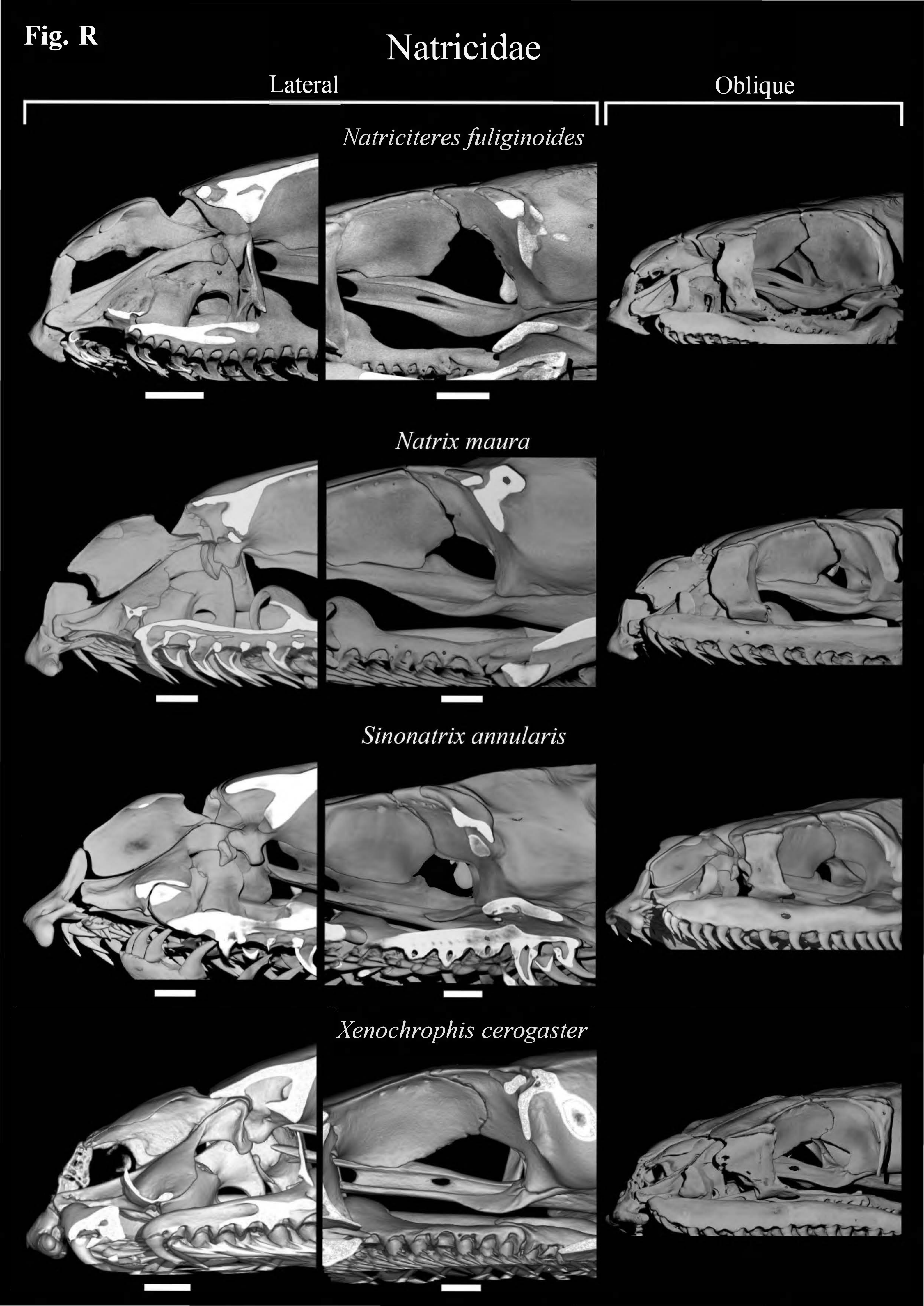
Fig. R
Natricidae
Lateral
Oblique
Natriciteres fuliginoides
Natrix maura
Sinonatrix annularis
Xenochrophis cerogaster

Fig. S

Dipsadidae

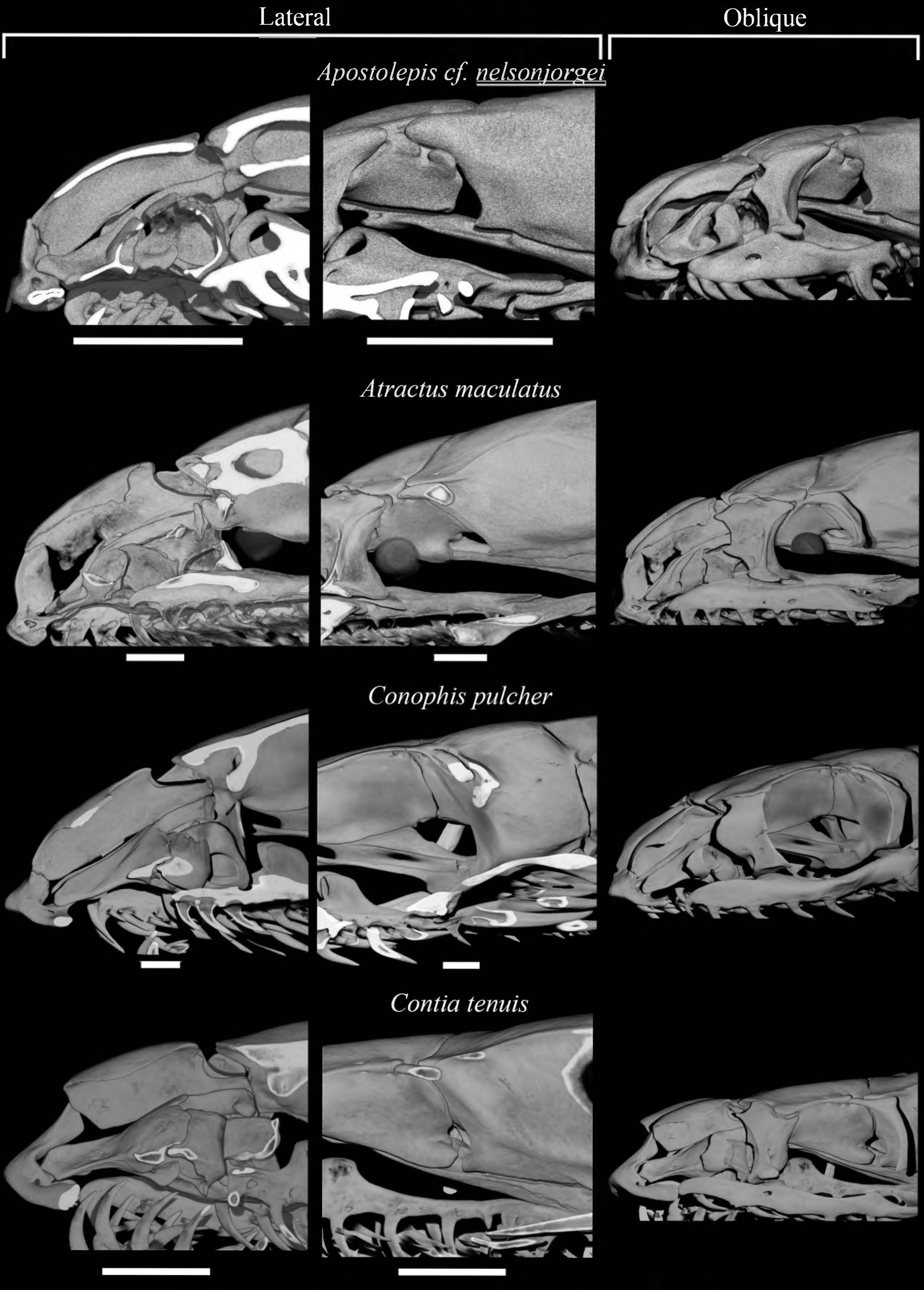

Fig. T

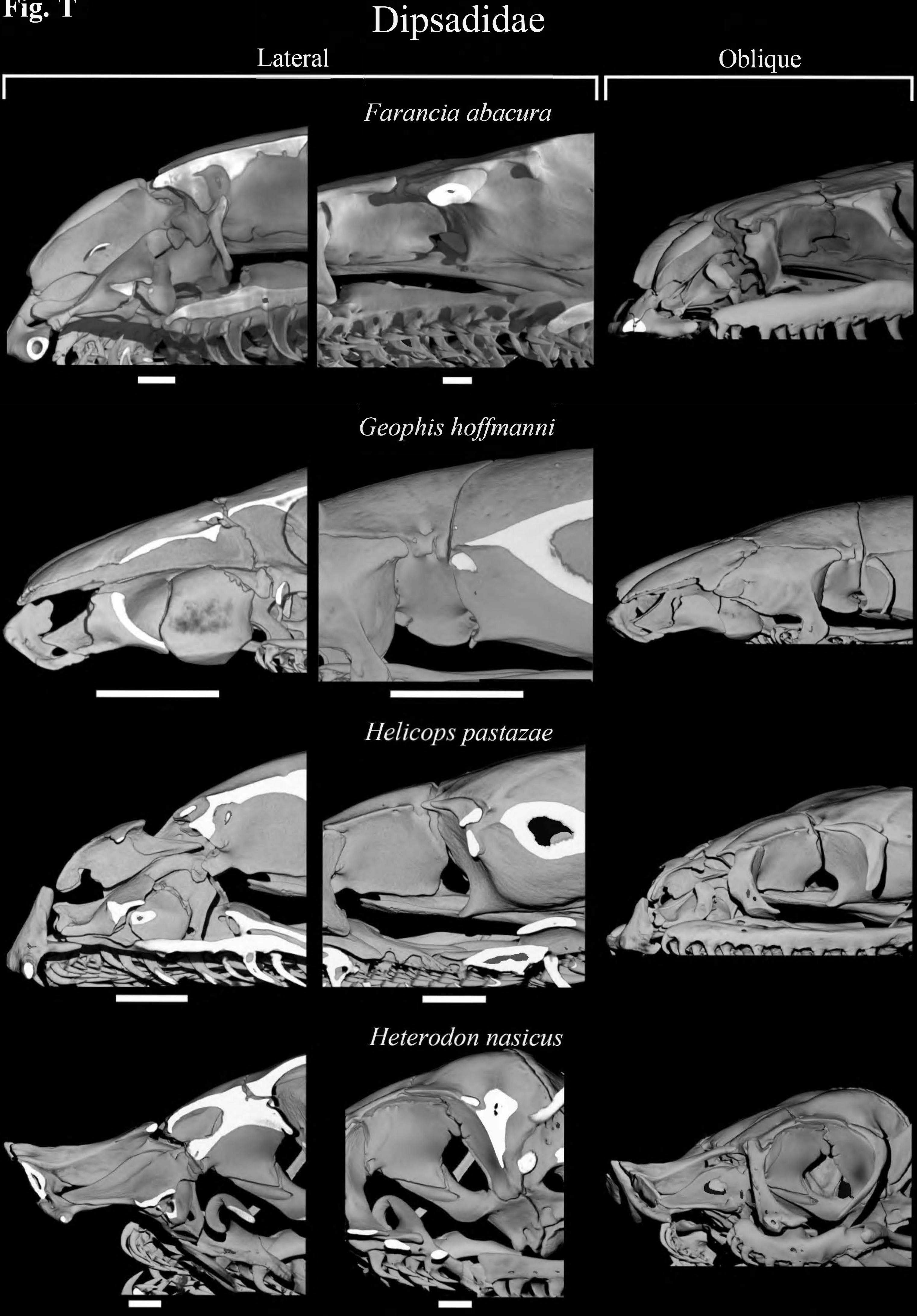
Dipsadidae
Lateral
Oblique
Farancia abacura
Geophis hoffmanni
Helicops pastazae
Heterodon nasicus

Fig. U

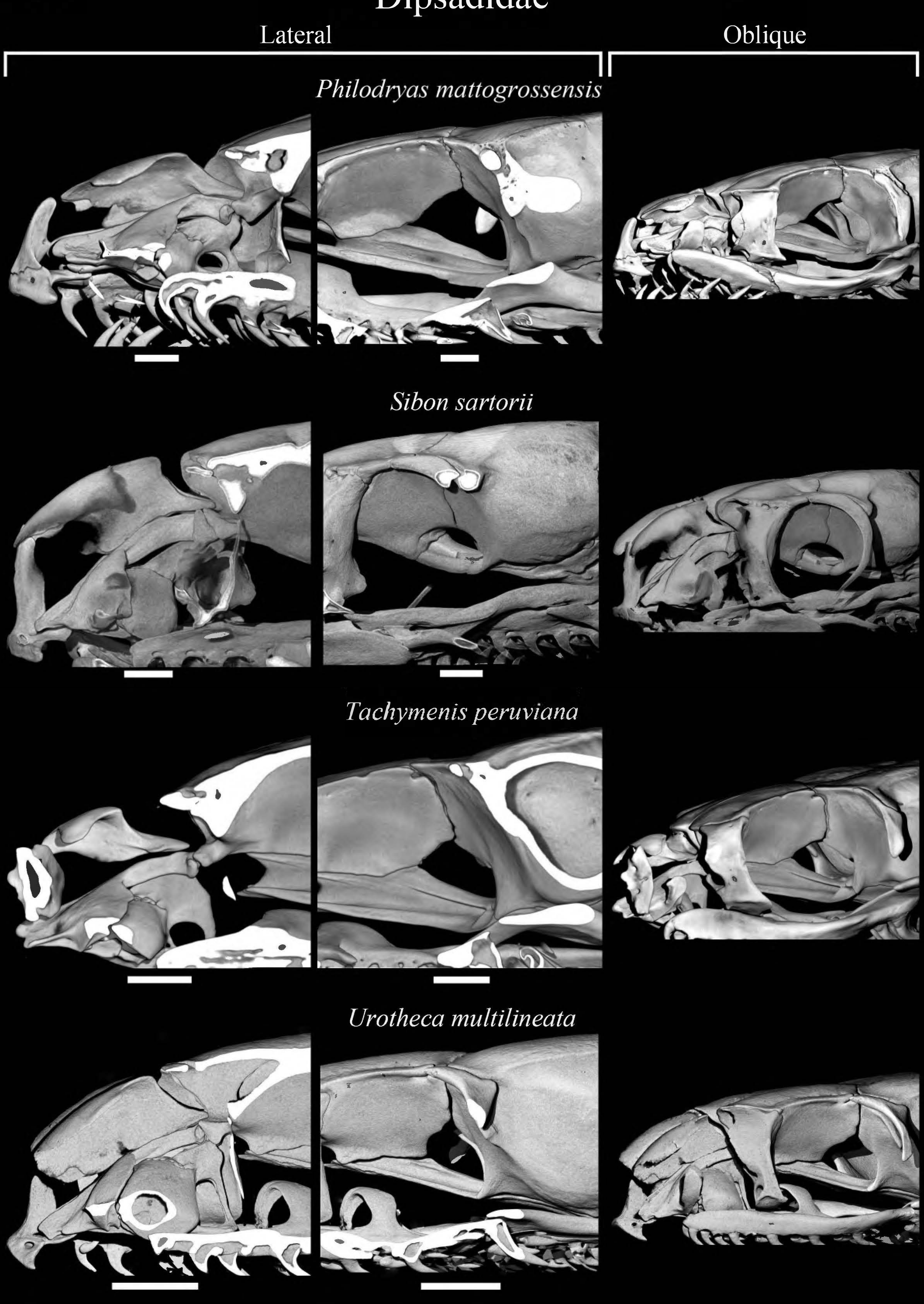

Dipsadidae
Lateral
Oblique
Philodryas mattogrossensis
Sibon sartorii
Tachymenis peruviana
Urotheca multilineata

Fig. V

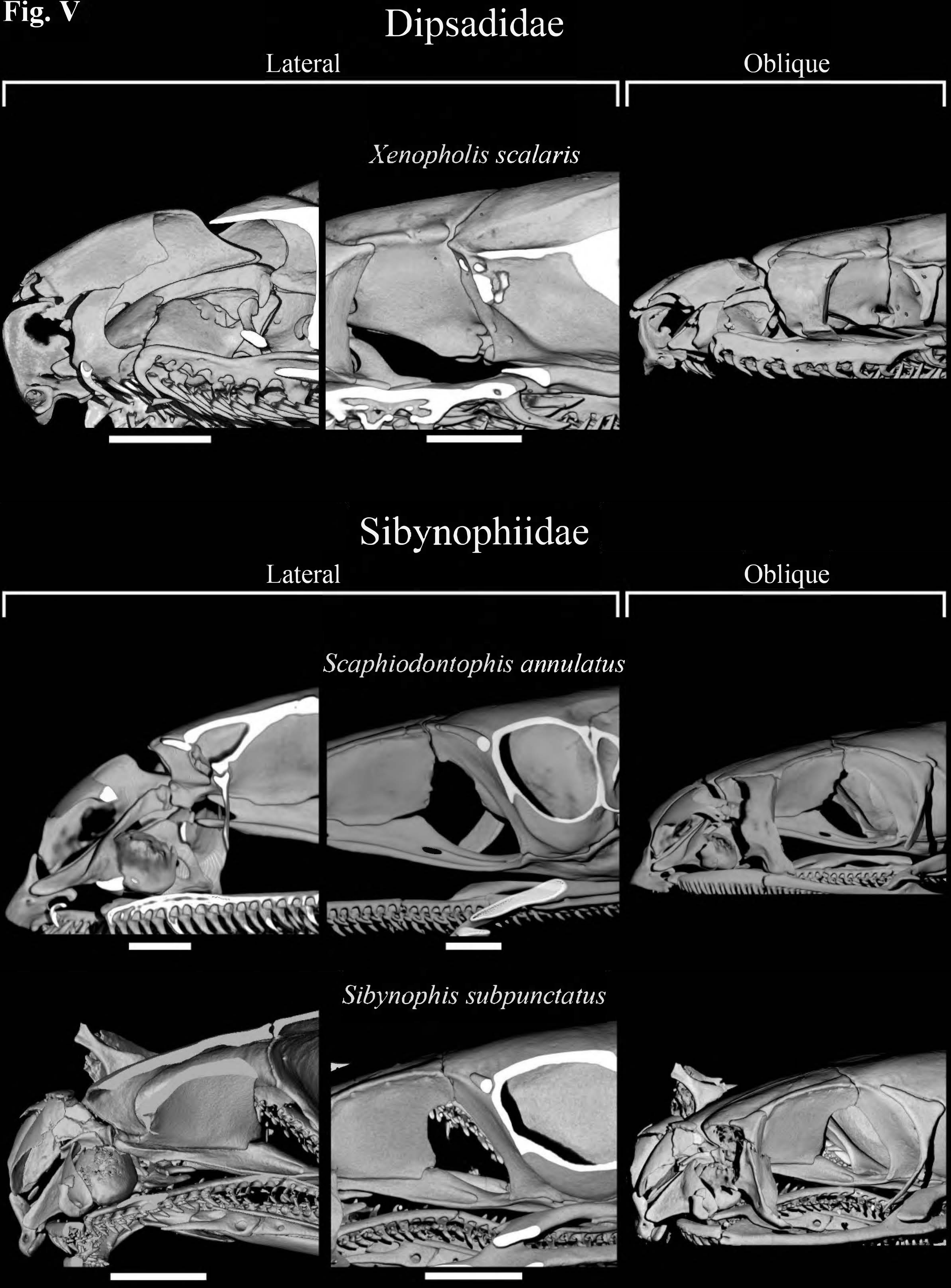
Dipsadidae
Lateral
Oblique
Xenopholis scalaris
Sibynophiidae
Lateral
Oblique
Scaphiodontophis annulatus
Sibynophis subpunctatus

Fig. W Sibynophiidae

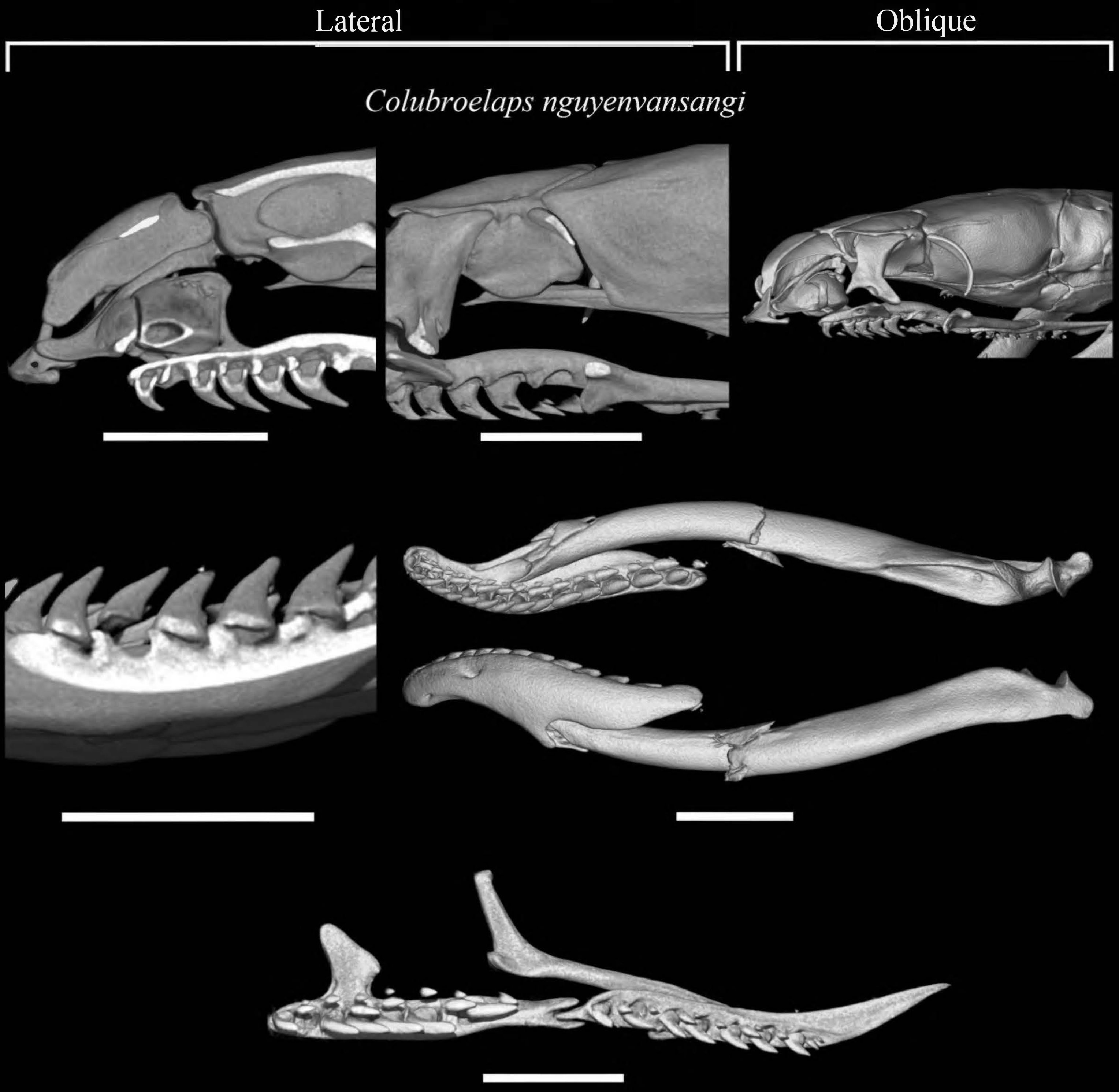

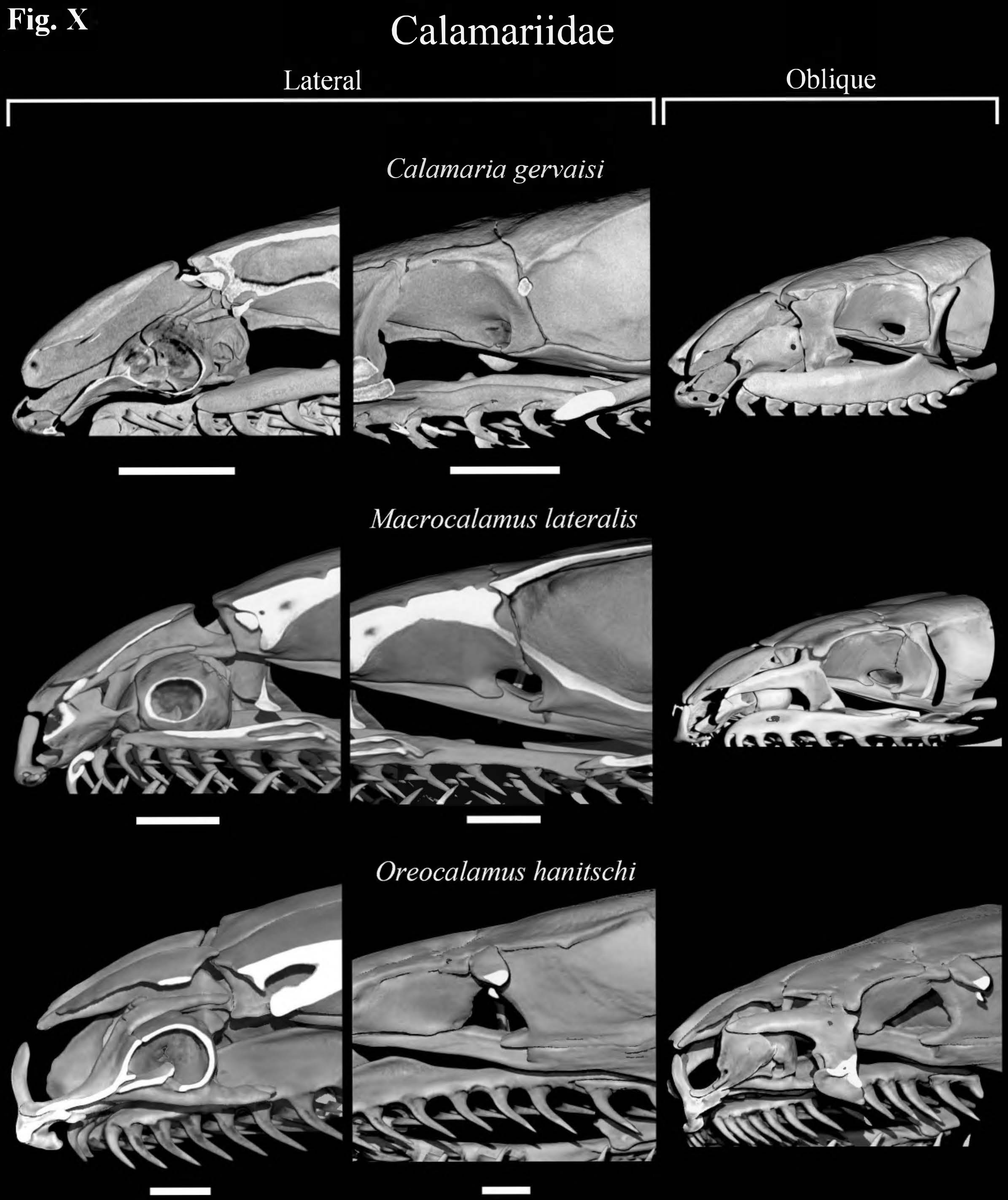
Fig. X
Calamariidae
Lateral
Oblique
Calamaria gervaisi
Macrocalamus lateralis
Oreocalamus hanitschi

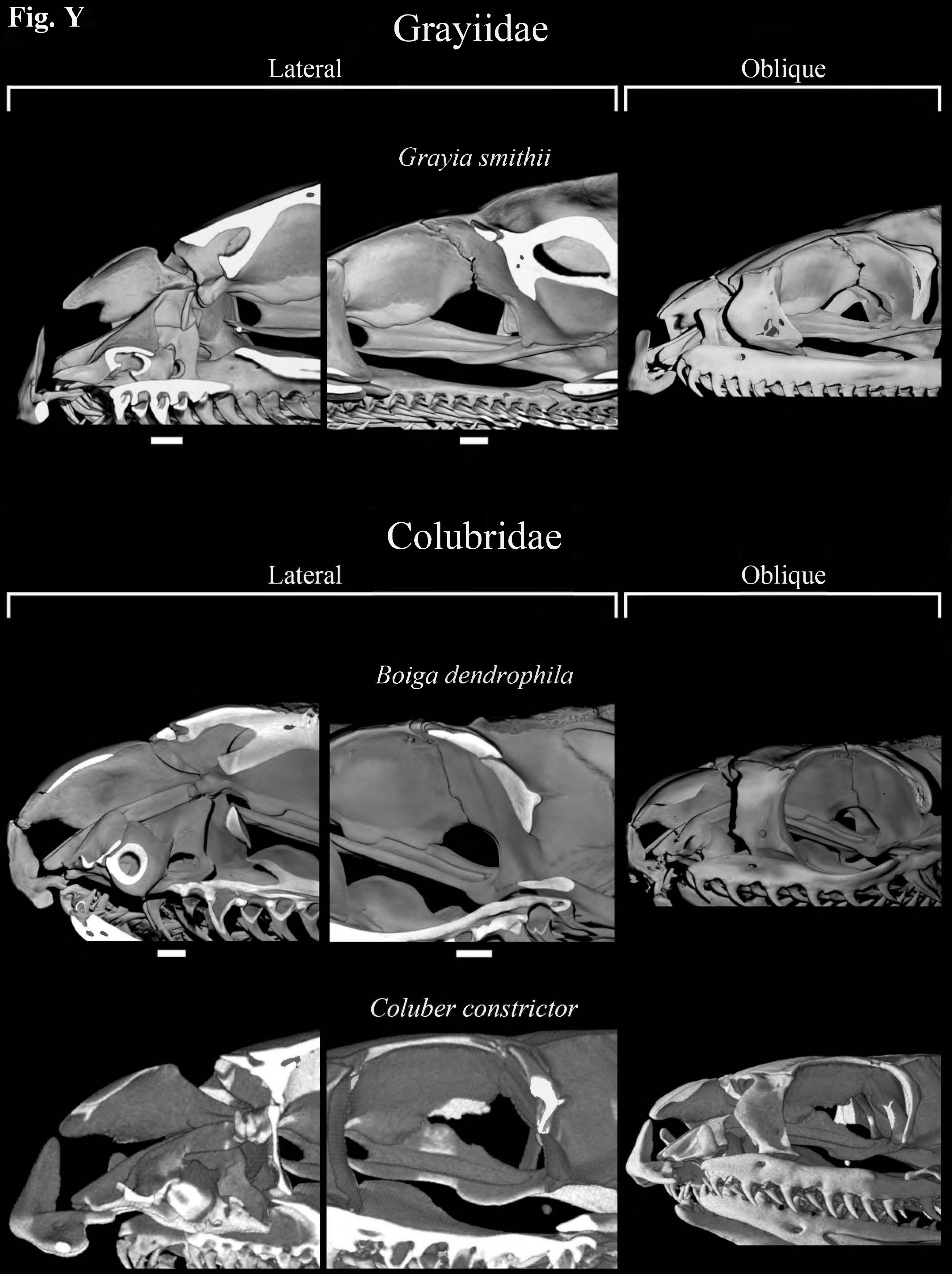
Fig. Y
Grayiidae
Lateral
Oblique
Grayia smithii
Colubridae
Lateral
Oblique
Boiga dendrophila
Coluber constrictor

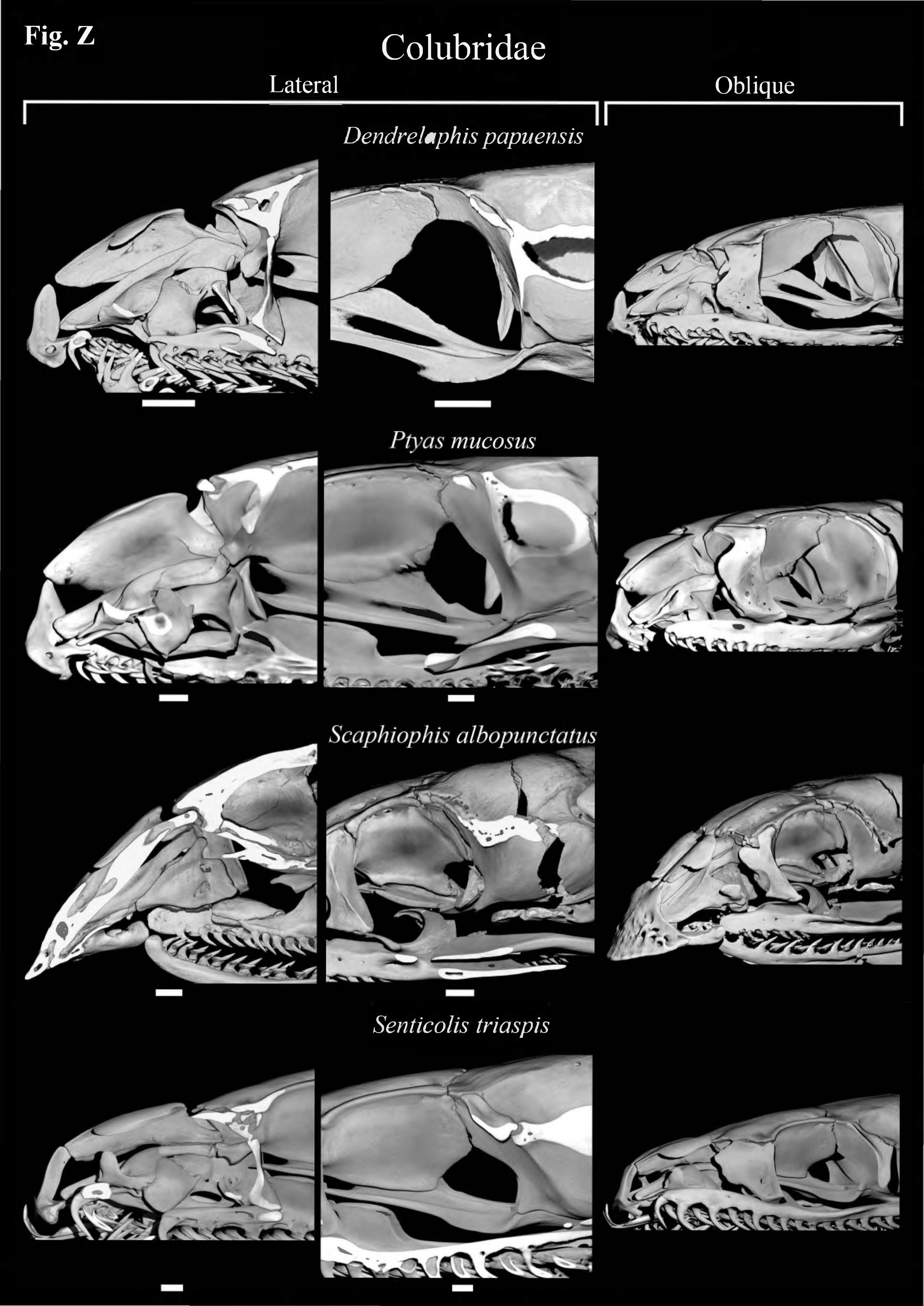
Fig. Z
Colubridae
Lateral
Oblique
Dendrelaphis papuensis
Ptyas mucosus
Scaphiophis albopunctatus
Senticolis triaspis

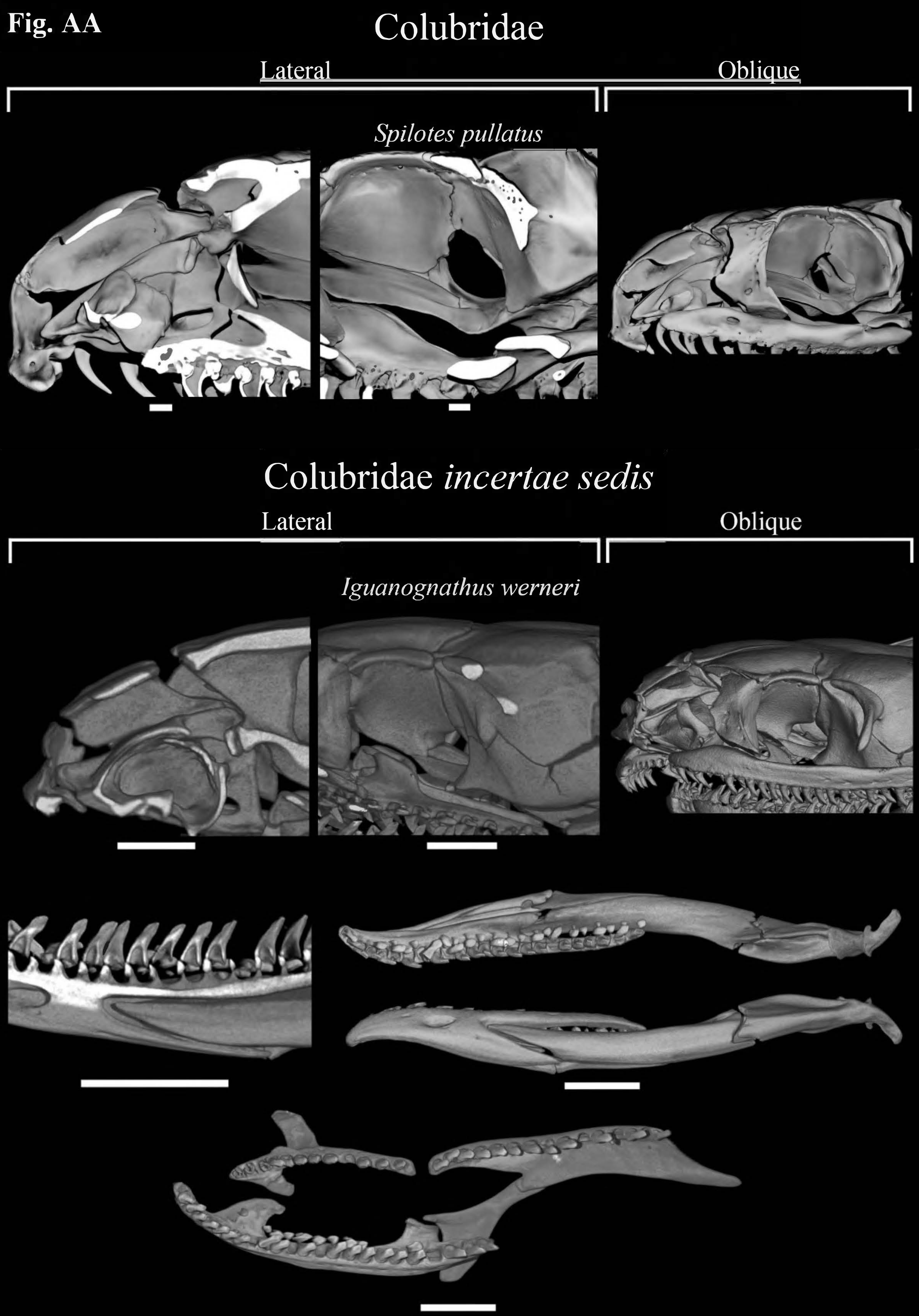
Fig. AA
Colubridae
Lateral
Oblique
Spilotes pullatus
Colubridae incertae sedis
Lateral
Oblique
Iguanognathus werneri

Fig. AB Elapoidea *incertae sedis*

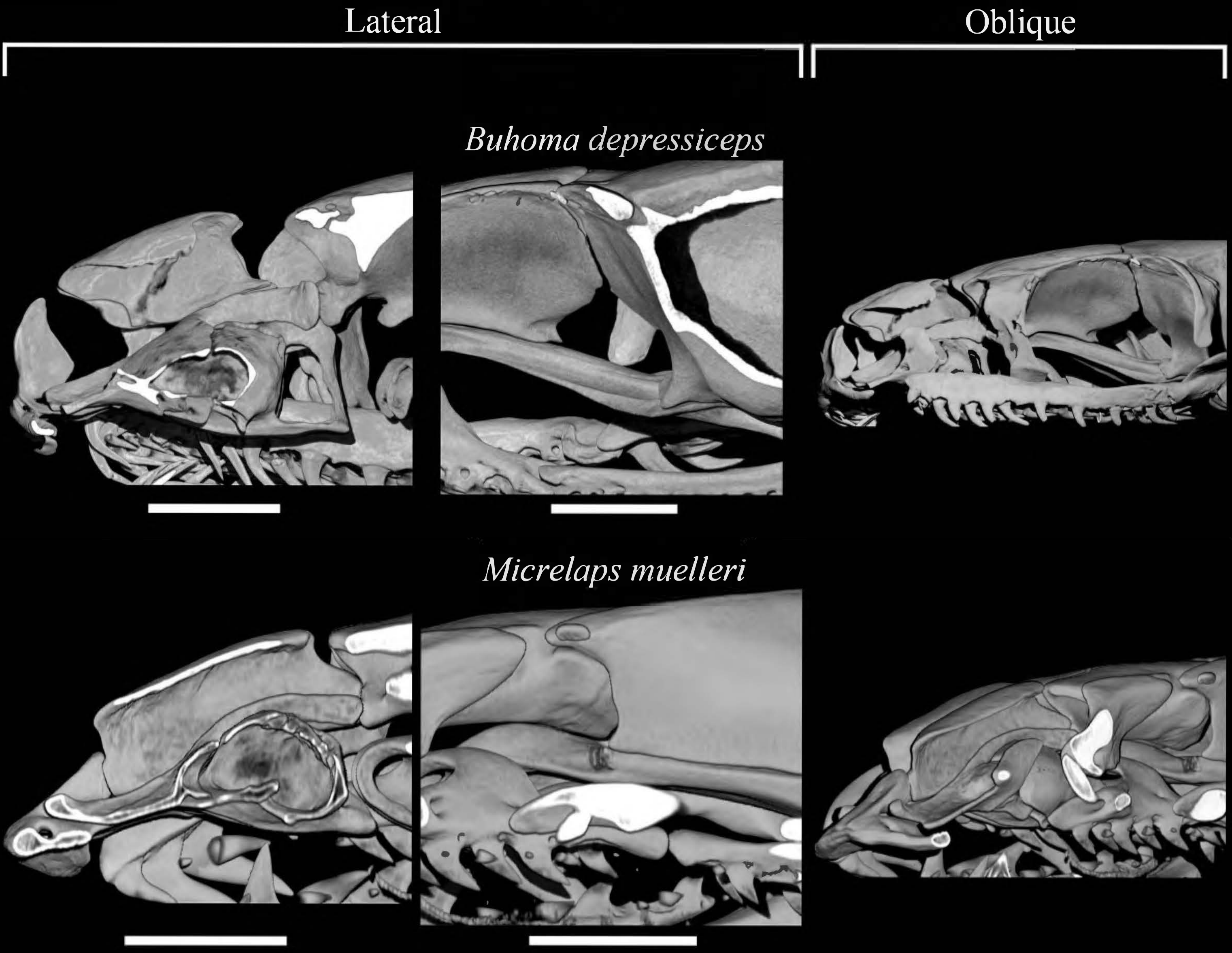

Supplement: S1 Appendix — Skull morphology of representatives of colubroidean families illustrating the naso-frontal joint and optic foramen/fenestra. Figure A, Tropidophiidae: Tropidophis nigriventris (AMNH 81182); Acrochordidae: Acrochordus granulatus (ZMB 9444). Figure B, Xenodermidae: Achalinus spinalis (AMNH 34621), Fimbrios klossi (BMNH 1946.1.15.88). Figure C, Xenodermidae: Xenodermus javanicus (FMNH 158613); Xylophiidae: Xylophis perroteti (BMNH 1955.1.3.10). Figure D, Pareidae: Pareas moellendorffi (AMNH 27770), Apopeltura boa (BMNH 47.12.30). Figure E, Viperidae: Azemiops kharini (ZMB 69985), Bothrops neuwiedi (MZUSP 1476), Causus rhombeatus (FMNH 74241), Vipera ursinii (MZUSP 8230). Figure F, Homalopsidae: Bitia hydroides (FMNH 229568), Brachyorrhos albus (FMNH 142322), Enhydris chinensis (AMNH 33870), Fordonia leucobalia (AMNH 107179). Figure G, Homalopsidae: Homalopsis buccata (MNHN 1963.728); Psammophiidae: Malpolon monspessulanus (AMNH 140768), Mimophis mahfalensis (UMMZ 209653). Figure H, Psammophiidae: Psammophylax variabilis (AMNH 73213), Rhamphiophis oxyrhynchus (AMNH 16890), Psammophis phillipsi (AMNH 67750). Figure I, Cyclocoridae: Cyclocorus lineatus (MNHN 1900.413), Oxyrhabdium modestus (FMNH 53386); Atractaspididae: Aparallactus modestus (AMNH 50545). Figure J, Atractaspididae: Atractaspis bibronii (AMNH 82073), Homoroselaps lacteus (LSUMZ 57229), Macrelaps microlepidotus (FMNH 205860), Polemon christyi (FMNH 219913). Figure K, Lamprophiidae: Bothrolycus ater (AMNH 11976), Chamaelycus fasciatus (BMNH 1909.4.29.3), Dipsadoboa weileri (AMNH 12472), Lamprophis olivaceus (AMNH 12003). Figure L, Lamprophiidae: Lycodonomorphus rufulus (AMNH 140284), Lycophidion capense (AMNH 63771), Gonionotophis capensis (AMNH 73208), Pseudoboodon lemniscatus (MNHN 1905.179). Figure M, Pseudoxyrhophiidae: Alluaudina bellyi (UMMZ 201605), Dromicodryas quadrilineatus (UMMZ 209290), Duberria lutrix (UMMZ 154361), Heteroliodon occipitalis (UMMZ 218178). Figure N, Pseudoxyrhophiidae: Ithycyphus [file pone.0216148.s015.pdf]
