# Supplementary material for: Large-scale molecular phylogeny, morphology, divergence-time estimation, and the fossil record of advanced caenophidian snakes (Squamata: Serpentes)
Source: PLoS One. 2019 May 10;14(5):e0216148. doi: 10.1371/journal.pone.0216148 (PMC6512042; doi:10.1371/journal.pone.0216148)
Supplement: S2 Appendix — Posterior trunk vertebral morphology of representatives of colubroidean families. Figure A, Acrochordidae: Acrochordus javanicus (USNM 297404), scale bar = 2 mm; Xenodermidae: Achalinus rufescens (BMNH 1946.1.12.37), scale bar = 1 mm; Fimbrios klossi (BMNH 1946.1.15.88), scale bar = 1 mm; Pareidae: Pareas sp. (MZUSP 12186), scale bar = 1 mm. Figure B, Viperidae: Causus difilippi (MZUSP 18668), scale bar = 5 mm; Vipera ursinii (MZUSP 8230), scale bar = 5 mm; Azemiops feae (ROM 36976), scale bar = 1mm; Bothrops jararaca (MZUSP 14425), scale bar = 2mm. Figure C, Homalopsidae: Cerberus rynchops (MZUSP 9569), scale bar = 2mm; Homalopsis buccata (MZUSP 11483), scale bar = 1mm. Psammophiidae: Psammophis lineolatus (MZUSP 8221), scale bar = 1mm; Mimophis mahfalensis (MZSUP 12188), scale bar = 2mm. Figure D, Pseudoxyrhophiidae: Madagascarophis colubrinus (BMNH 89.8.28.23), scale bar = 2mm; Ditypophis vivax (BMNH_99.12.5.125), scale bar = 1mm; Lamprophiidae: Boaedon fuliginosus (MZUSP 8167), scale bar = 2mm; Crotaphopeltis hotamboeia (MZUSP 19602), scale bar = 1mm. Figure E, Atractaspididae: Atractaspis irregulares (MZUSP 10826), scale bar = 1mm; Homoroselaps lacteus (LSUMZ 57229), scale bar = 1mm. Elapidae: Sinomicrurus macclellandi (ROM 37113), scale bar = 1mm; Naja naja (UMMZ 181137), scale bar = 1mm. Figure F, Elapidae: Micrurus corallinus (MZUSP 13112), scale bar = 1mm; Cyclocoridae: Cyclocorus lineatus (BMNH 96.3.30.78), scale bar = 1mm; Natricidae: Natrix natrix (MZUSP 2514), scale bar = 2mm; Natriciteres olivacea (MZUSP 2083), scale bar = 1mm. Figure G, Sibynophiidae: Scaphiodontophis annulatus (MZUSP 5971), scale bar = 2mm; Grayiidae: Grayia smithii (MZUSP 8130), scale bar = 1mm; Grayia tholloni (MZUSP 8135), scale bar = 2mm; Calamariidae: Oreocalamus hanitschi (BMNH 1929.12.22.106), scale bar = 1mm. Figure H, Colubridae: Chironius bicarinatus (MZUSP 13860), scale bar = 2mm; Spilotes pullatus (MZUSP 13845), scale bar = 2mm; Oxybelis aeneus (MZUSP 13028), scale bar = [file pone.0216148.s016.pdf]

S2 Appendix

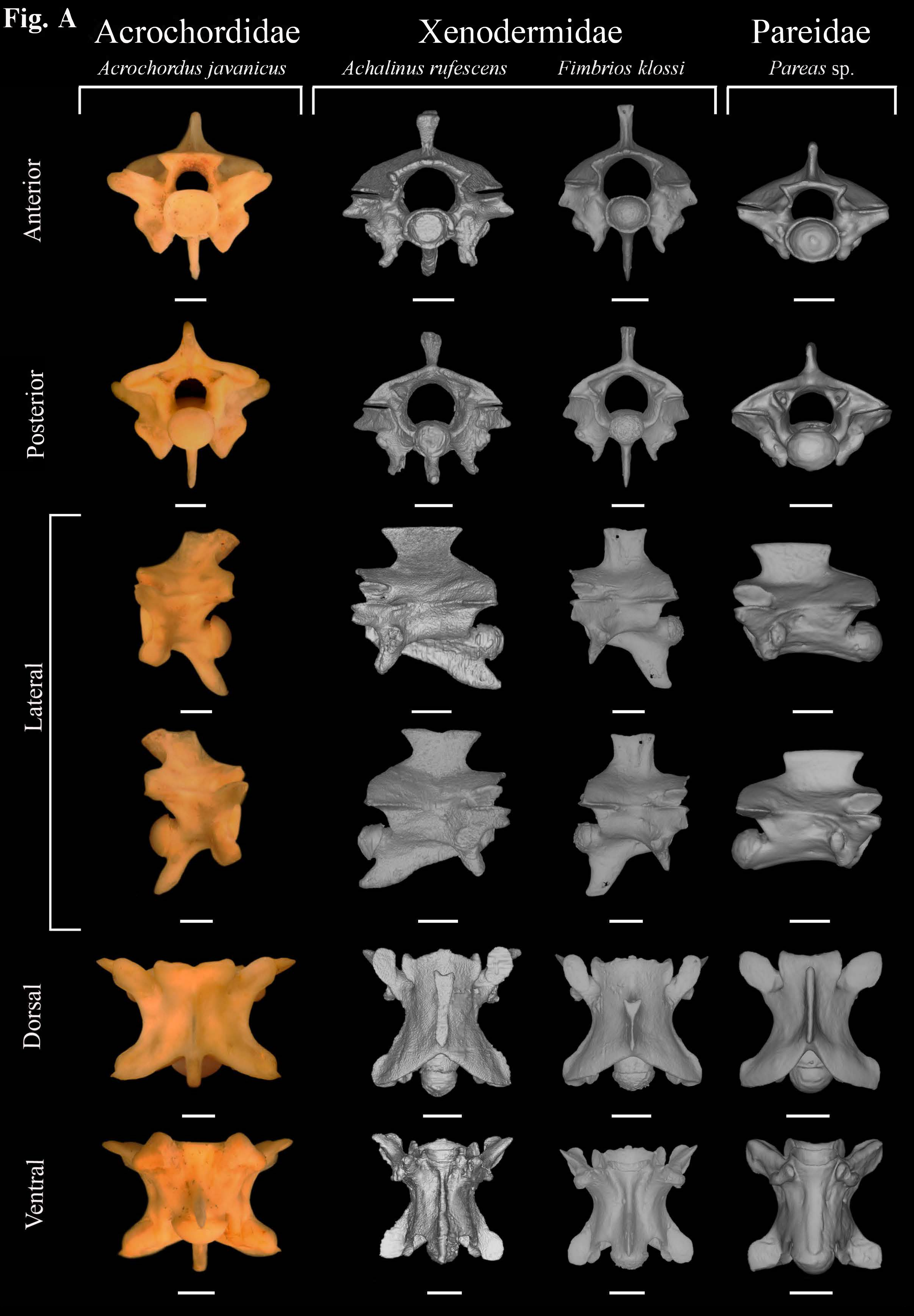
Fig. A
Acrochordidae
Xenodermidae
Pareidae
Acrochordus javanicus
Achalinus rufescens
Fimbrios klossi
Pareas sp.
Anterior
Posterior
Lateral
Dorsal
Ventral

Fig. B

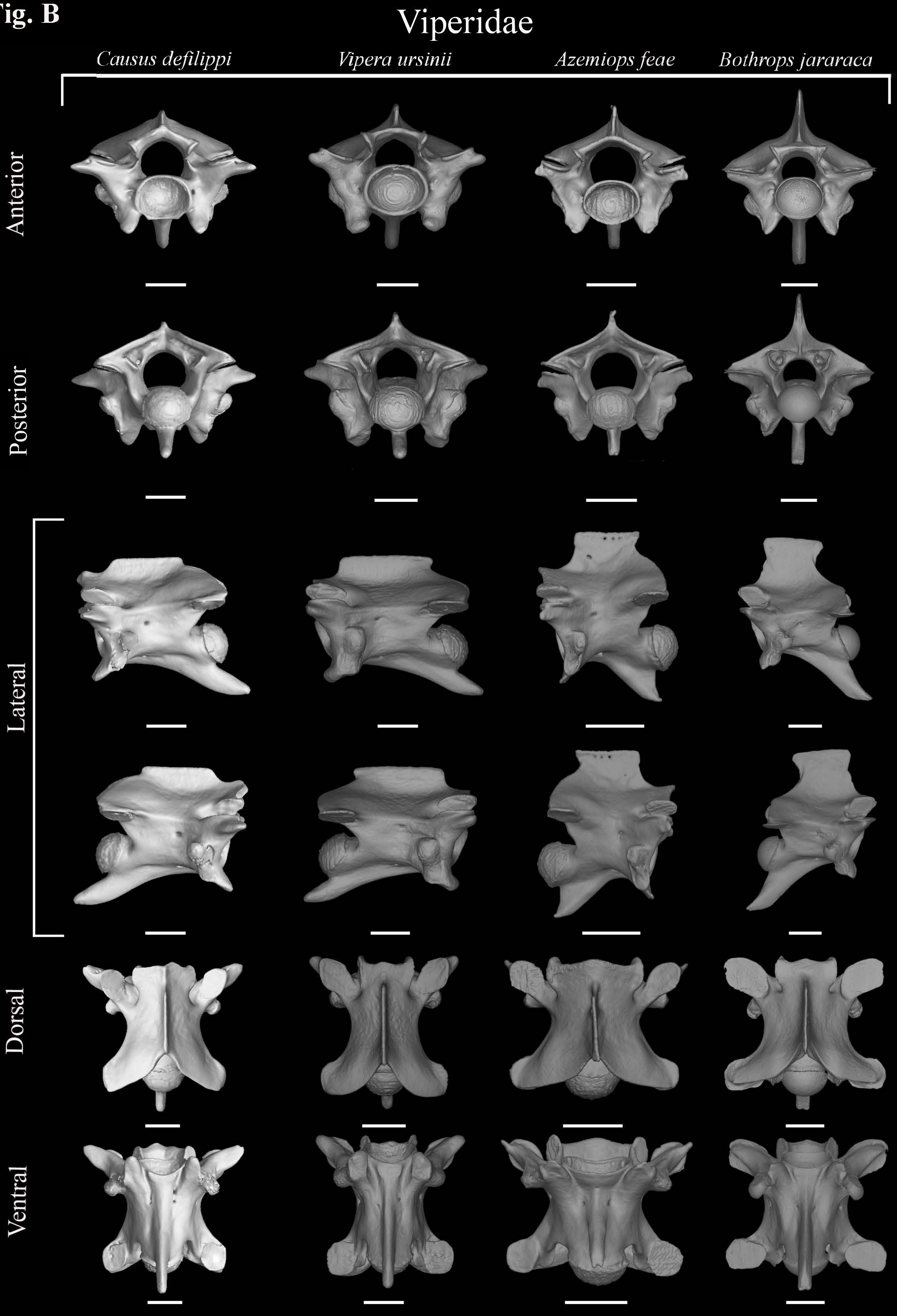
Viperidae
Causus defilippi
Vipera ursinii
Azemiops feae
Bothrops jararaca
Anterior
Posterior
Lateral
Dorsal
Ventral

Fig. C

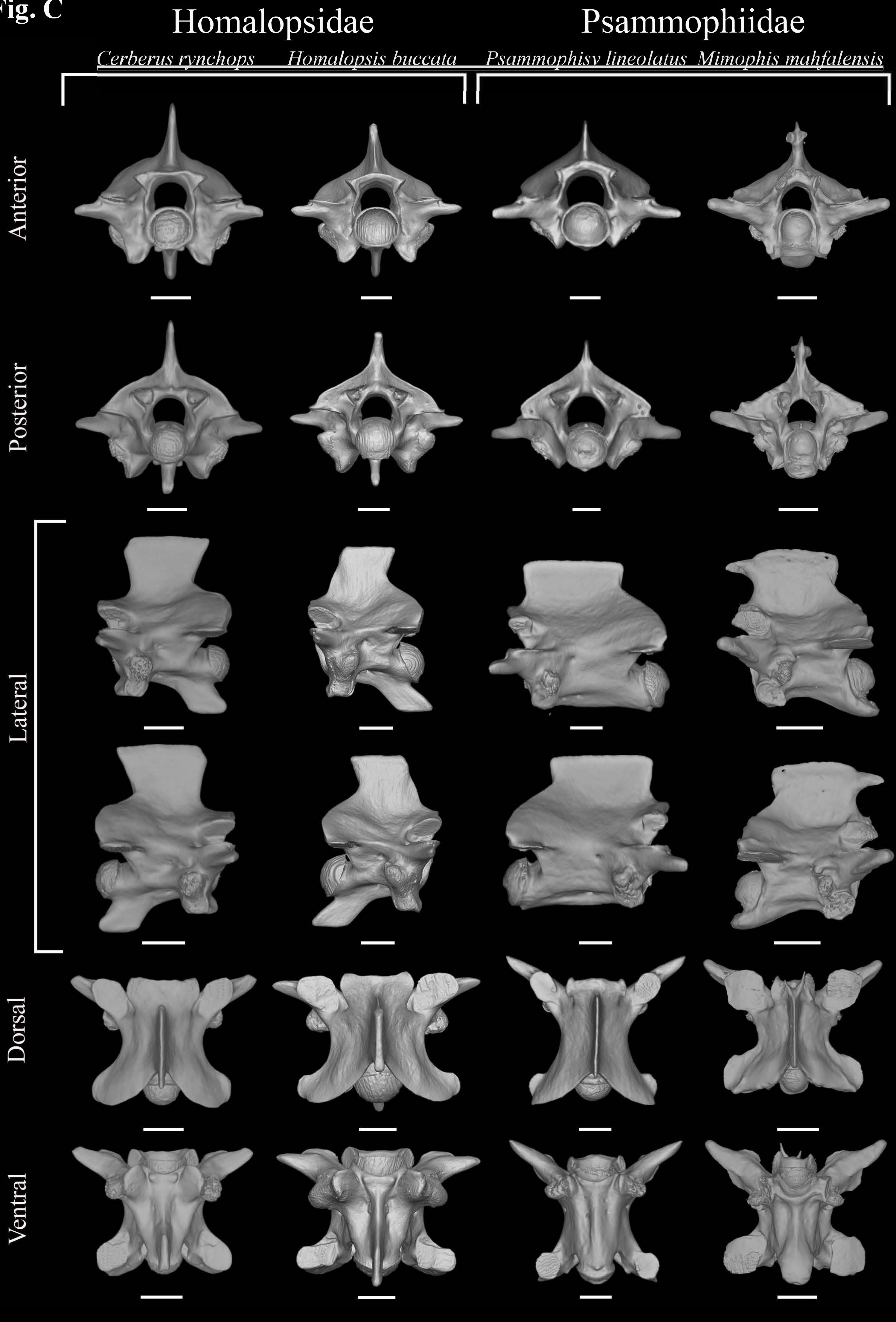
Homalopsidae
Psammophiidae
Cerberus rynchops
Homalopsis buccata
Psammophisv lineolatus
Mimophis mahfalensis
Anterior
Posterior
Lateral
Dorsal
Ventral

Fig. D

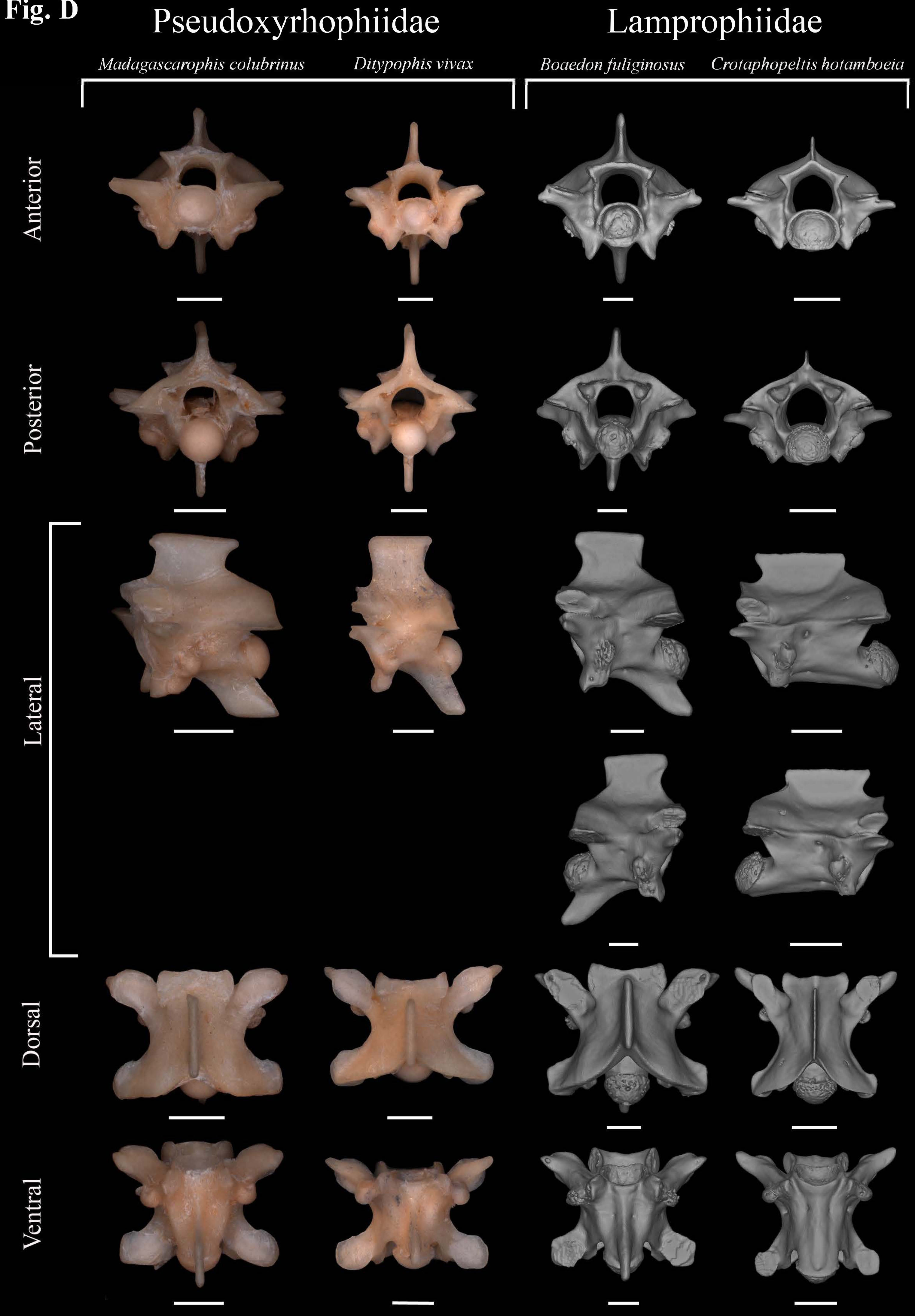

Pseudoxyrhophiidae
Lamprophiidae
Madagascarophis colubrinus
Ditypophis vivax
Boaedon fuliginosus
Crotaphopeltis hotamboeia
Anterior
Posterior
Lateral
Dorsal
Ventral

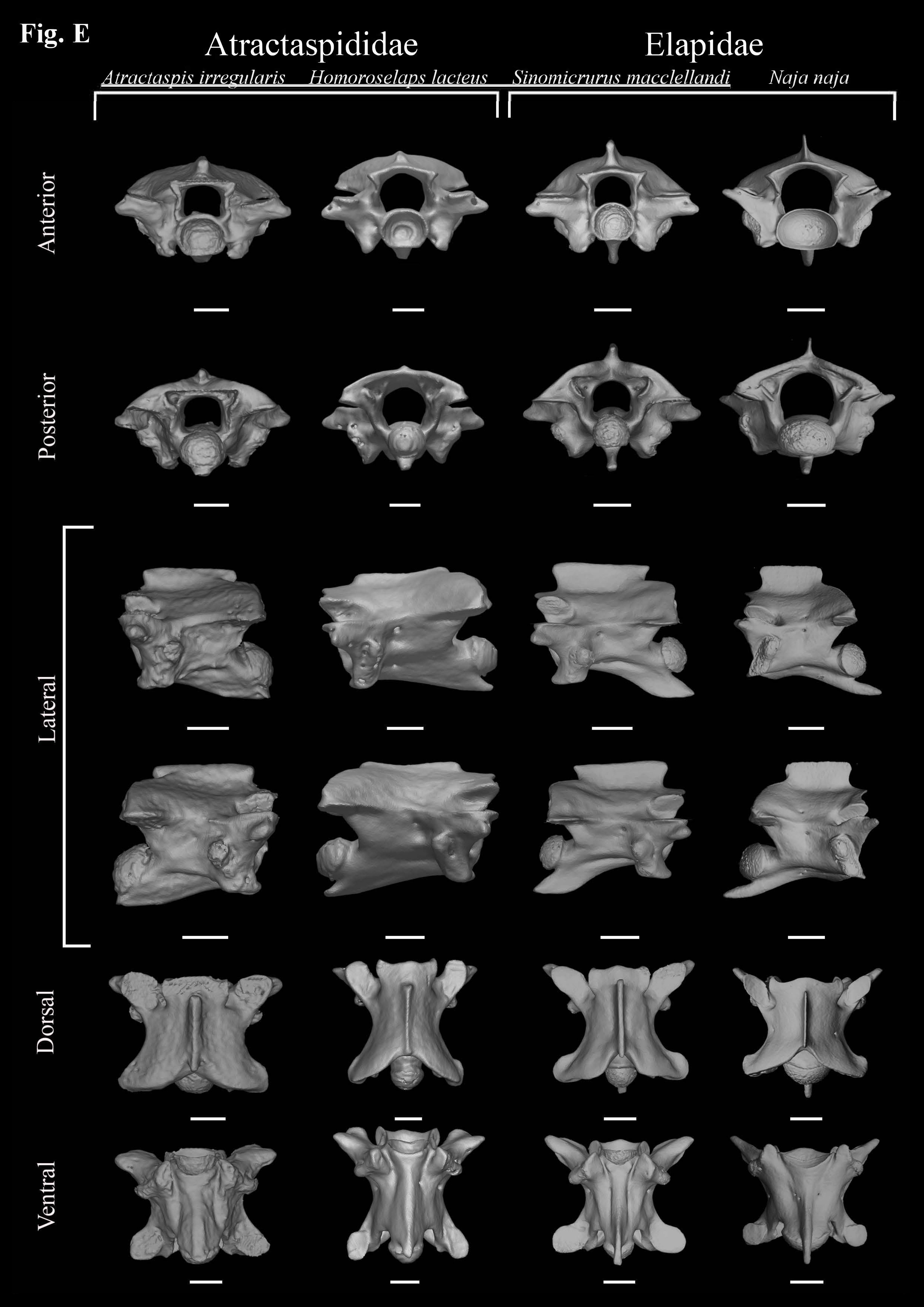
Fig. E
Atractaspididae
Elapidae
Atractaspis irregularis
Homoroselaps lacteus
Sinomicrurus macclellandi
Naja naja
Anterior
Posterior
Lateral
Dorsal
Ventral

Fig. F

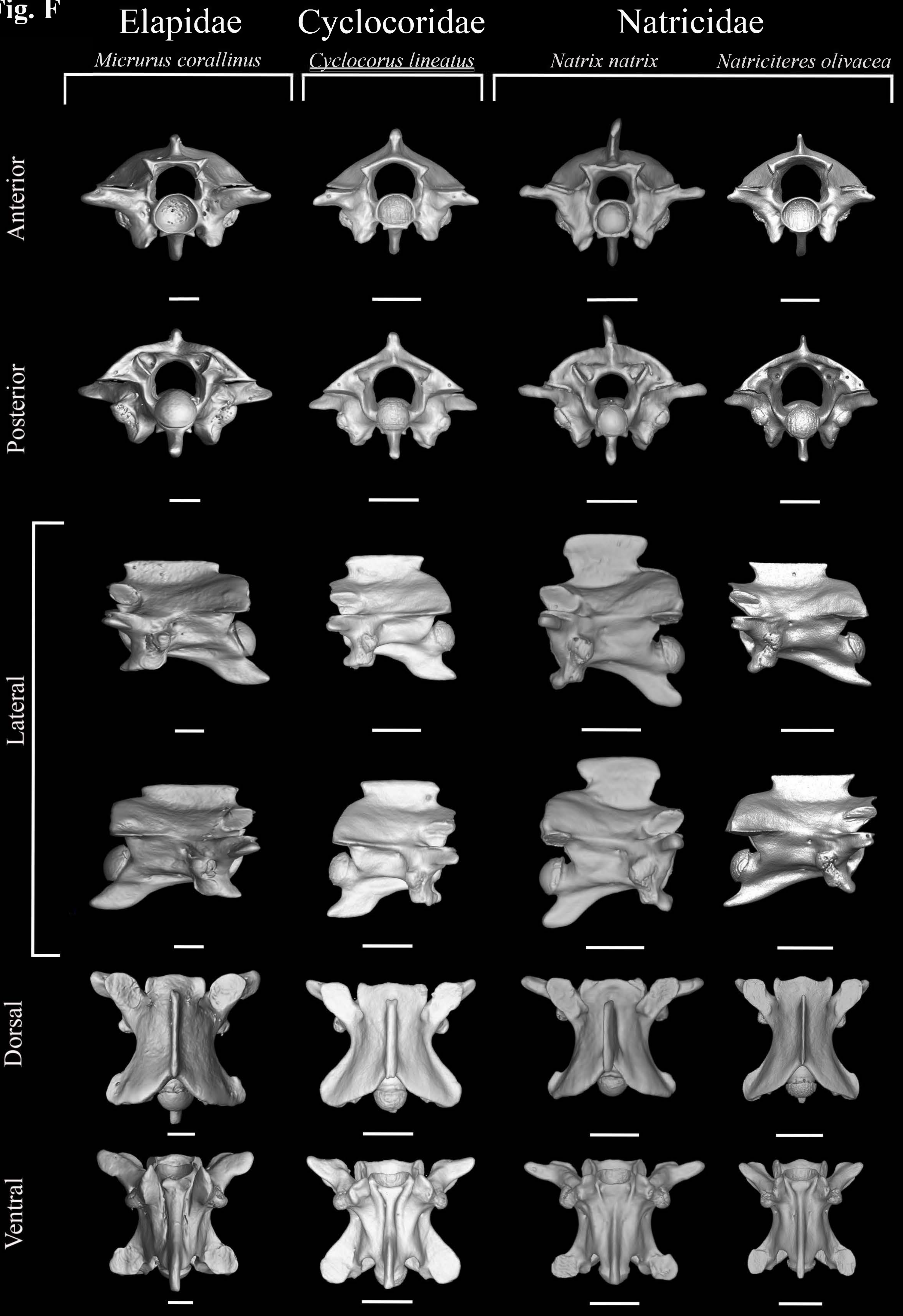
Elapidae
Cyclocoridae
Natricidae
Micrurus corallinus
Cyclocorus lineatus
Natrix natrix
Natriciteres olivacea
Anterior
Posterior
Lateral
Dorsal
Ventral

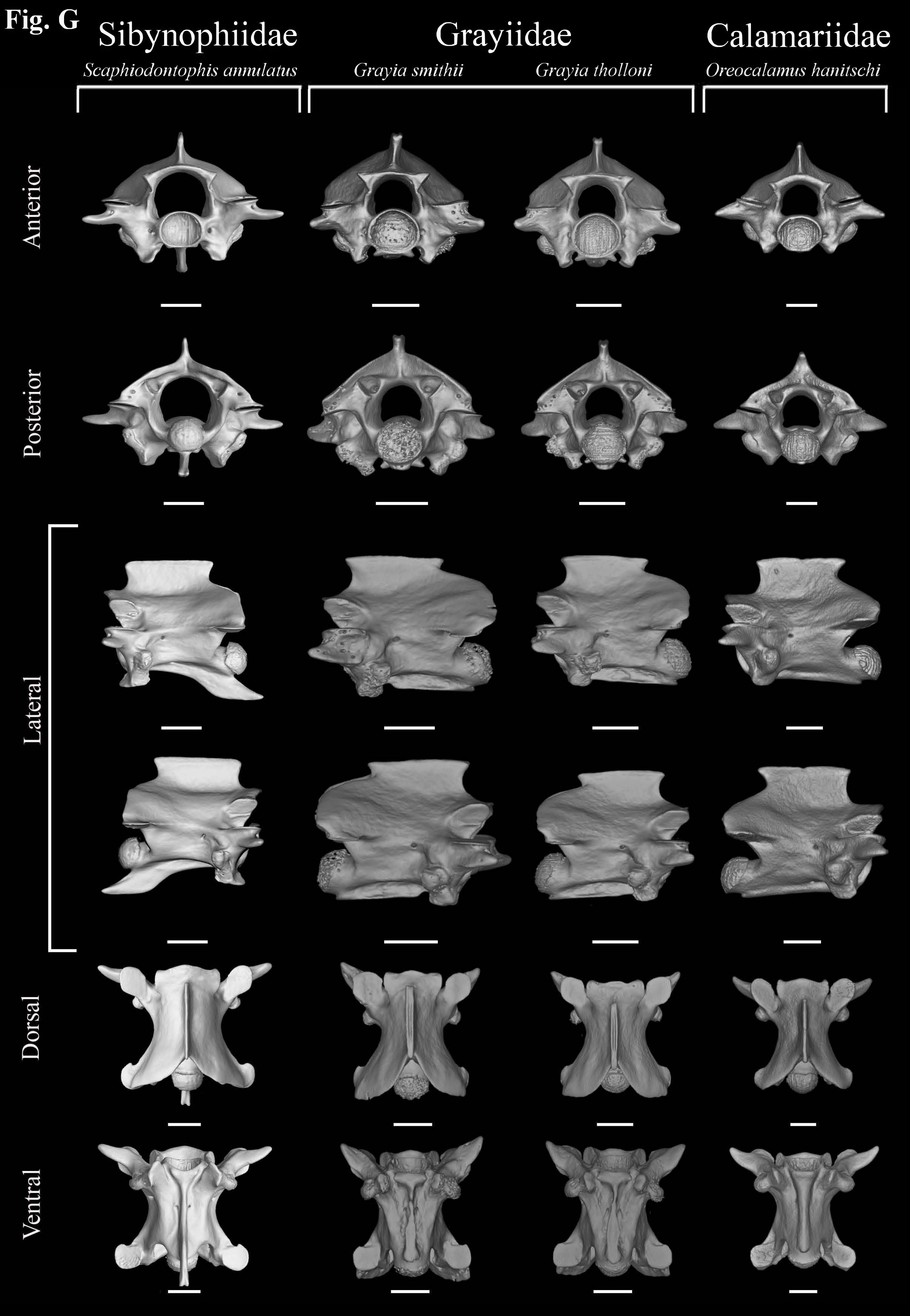
Fig. G
Sibynophiidae
Grayiidae
Calamariidae
Scaphiodontophis annulatus
Grayia smithii
Grayia tholloni
Oreocalamus hanitschi
Anterior
Posterior
Lateral
Dorsal
Ventral

Fig. H

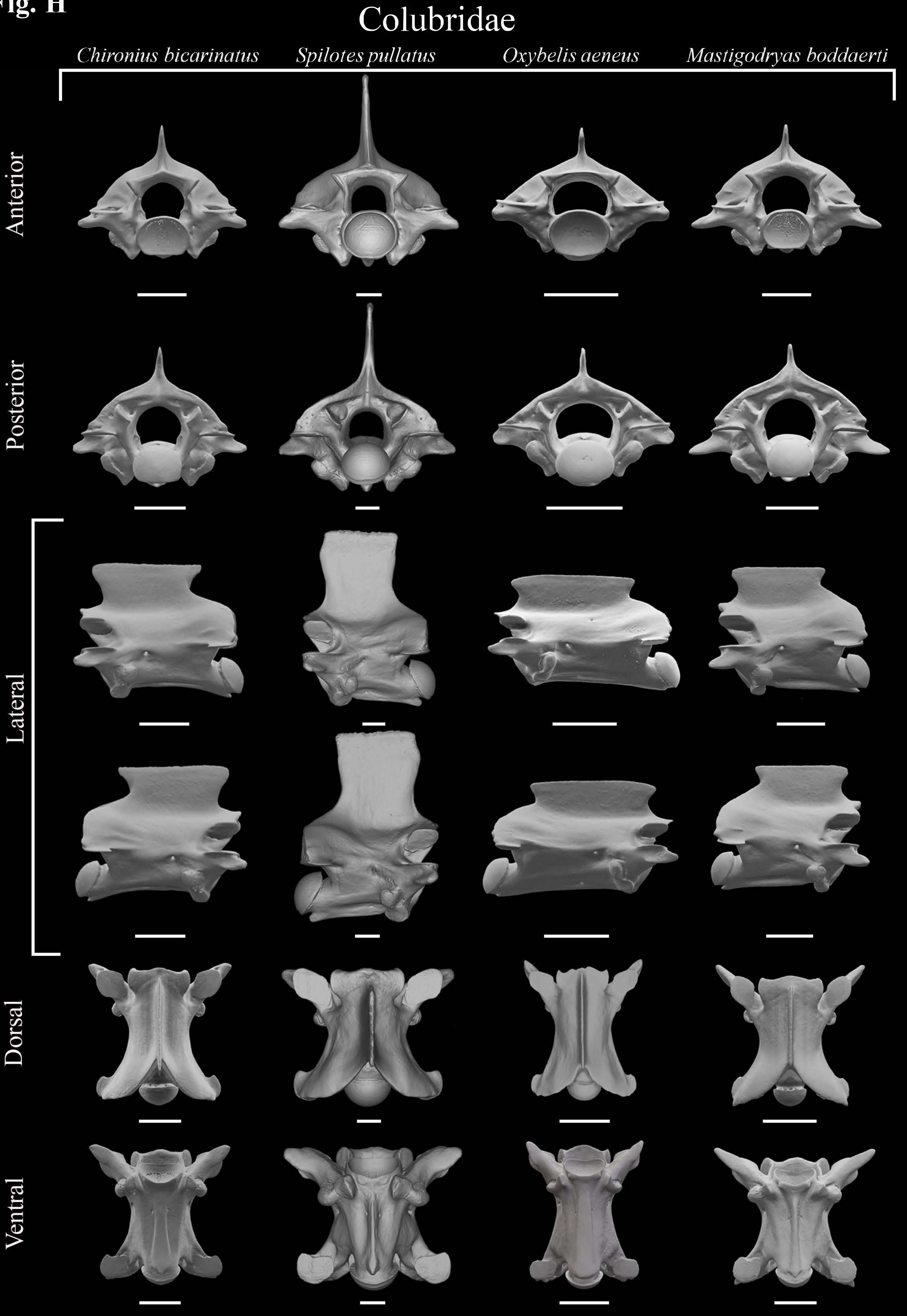
Colubridae
Chironius bicarinatus
Spilotes pullatus
Oxybelis aeneus
Mastigodryas boddaerti
Anterior
Posterior
Lateral
Dorsal
Ventral

Fig. I

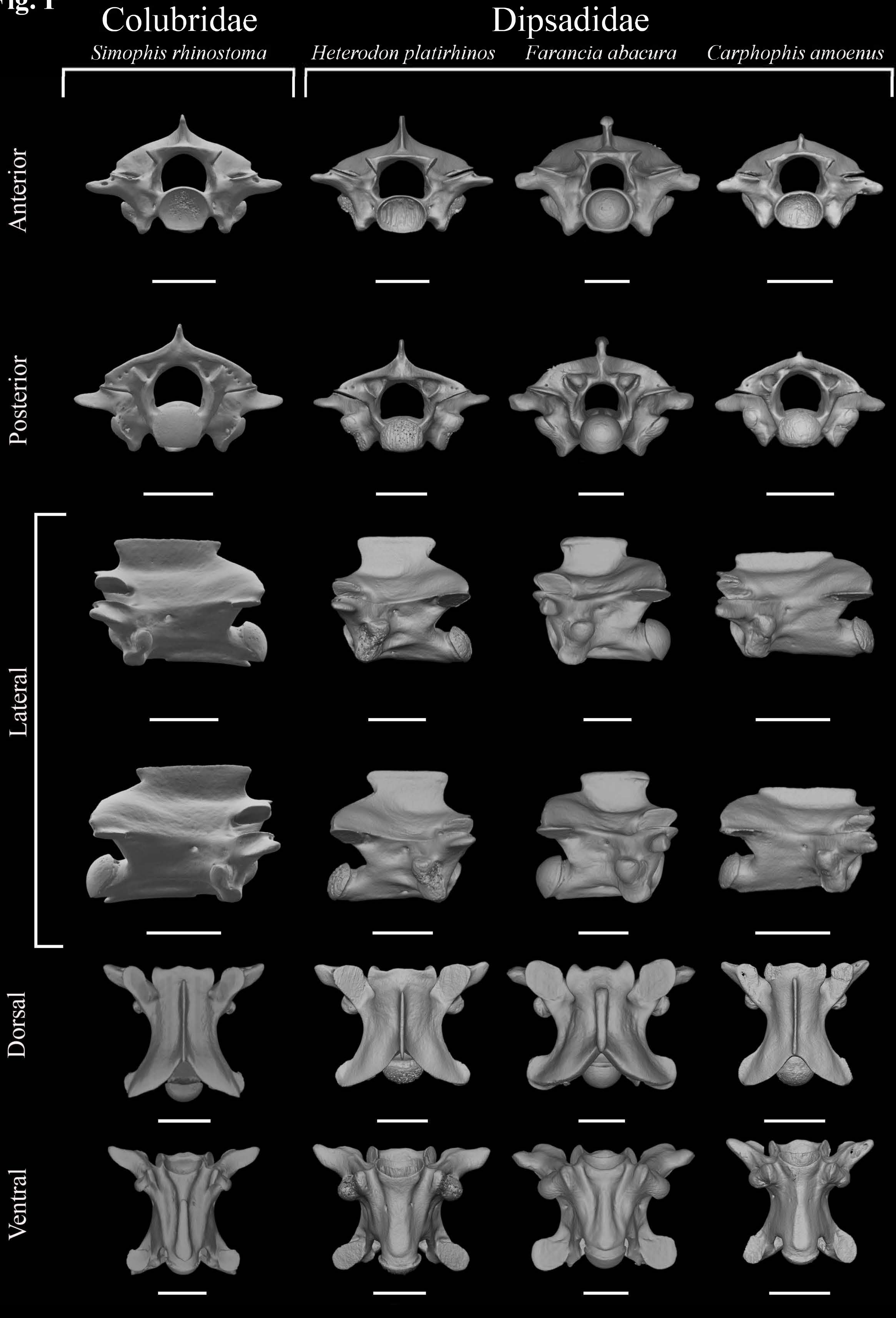

Colubridae
Dipsadidae
Simophis rhinostoma
Heterodon platirhinos
Farancia abacura
Carphophis amoenus
Anterior
Posterior
Lateral
Dorsal
Ventral

Fig. J

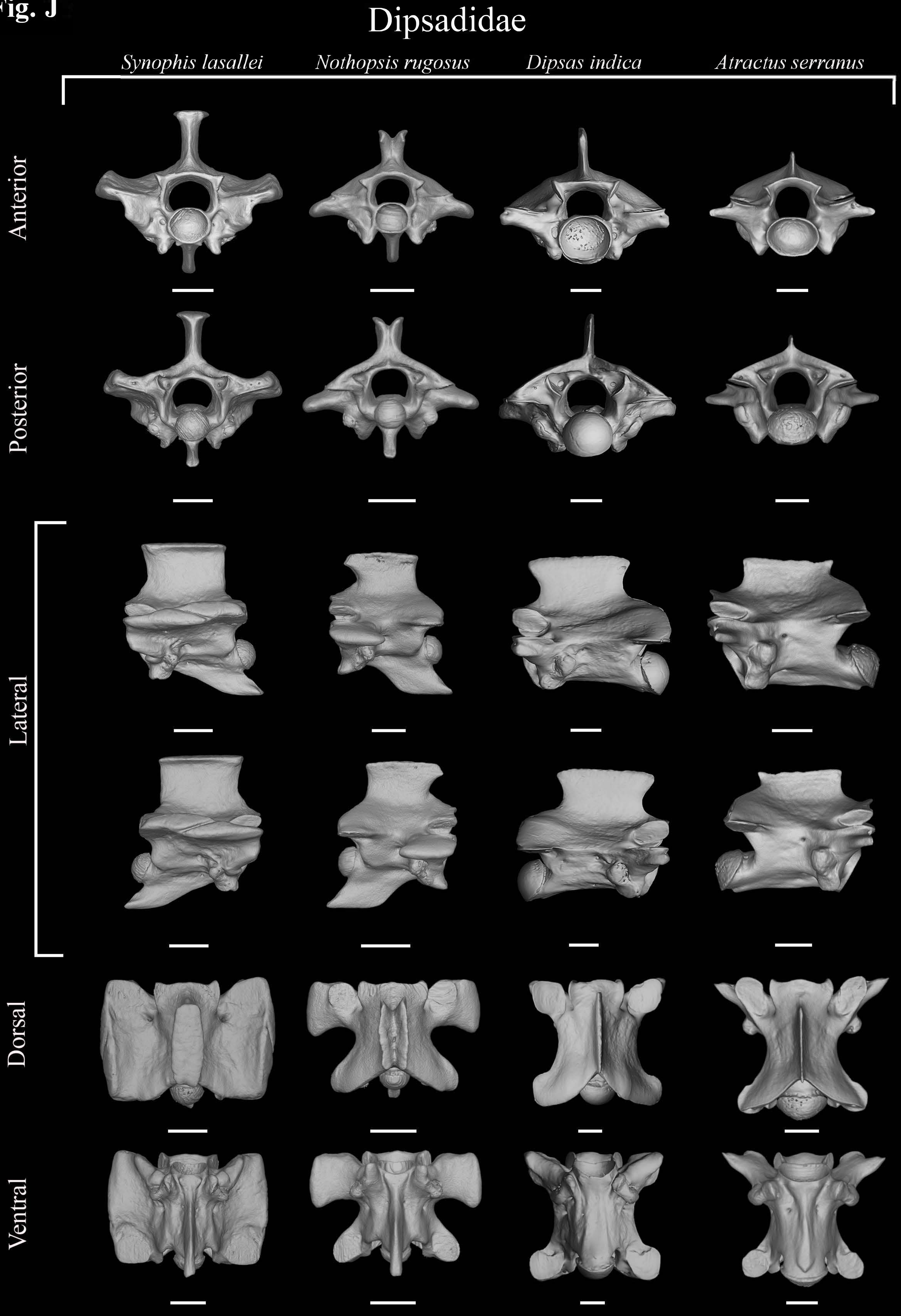

Dipsadidae
Synophis lasallei
Nothopsis rugosus
Dipsas indica
Atractus serranus
Anterior
Posterior
Lateral
Dorsal
Ventral

Fig. K

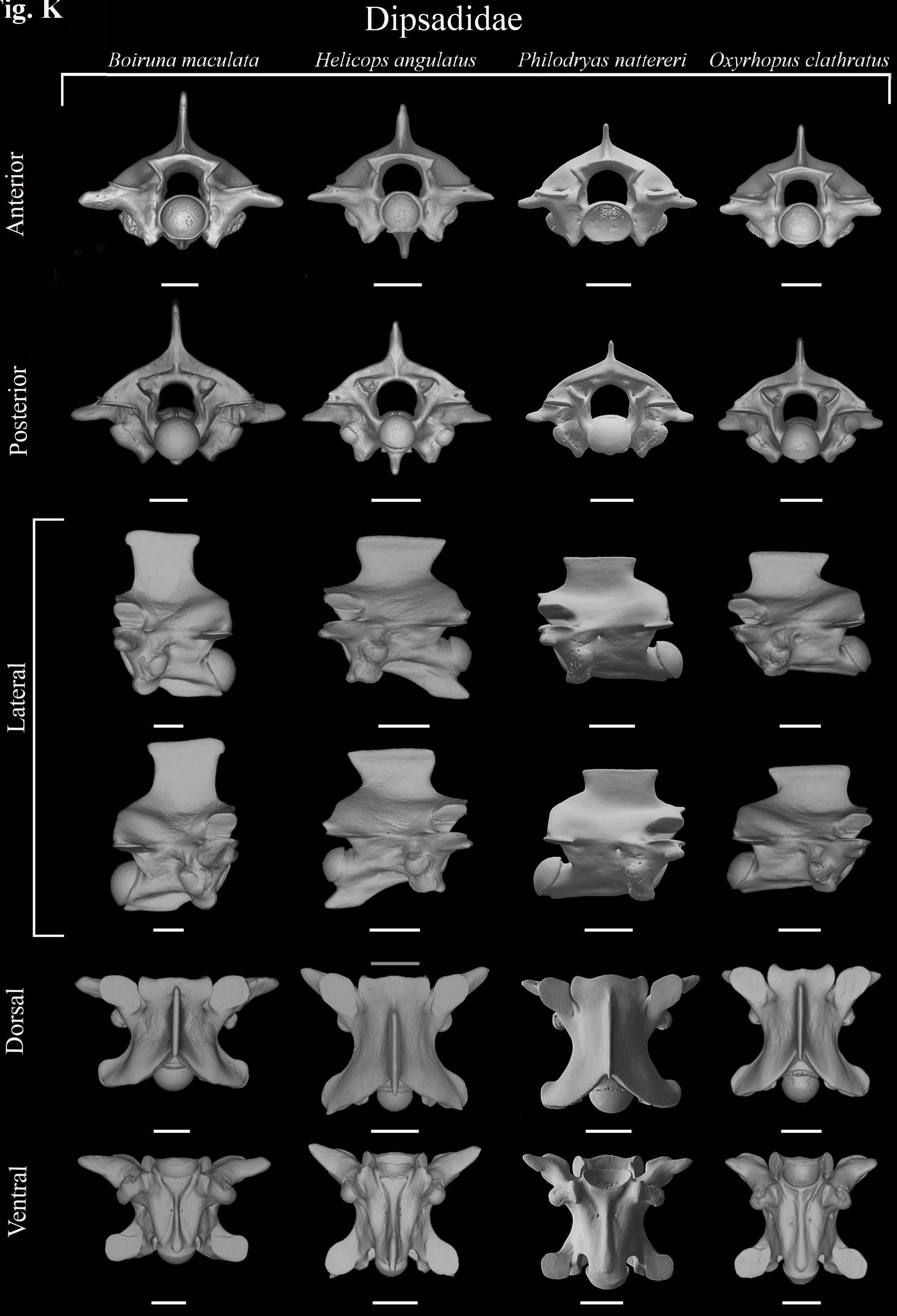
Dipsadidae
Boiruna maculata
Helicops angulatus
Philodryas nattereri
Oxyrhopus clathratus
Anterior
Posterior
Lateral
Dorsal
Ventral
