# Supplementary material for: Large-scale molecular phylogeny, morphology, divergence-time estimation, and the fossil record of advanced caenophidian snakes (Squamata: Serpentes)
Source: PLoS One. 2019 May 10;14(5):e0216148. doi: 10.1371/journal.pone.0216148 (PMC6512042; doi:10.1371/journal.pone.0216148)

S3 Appendix

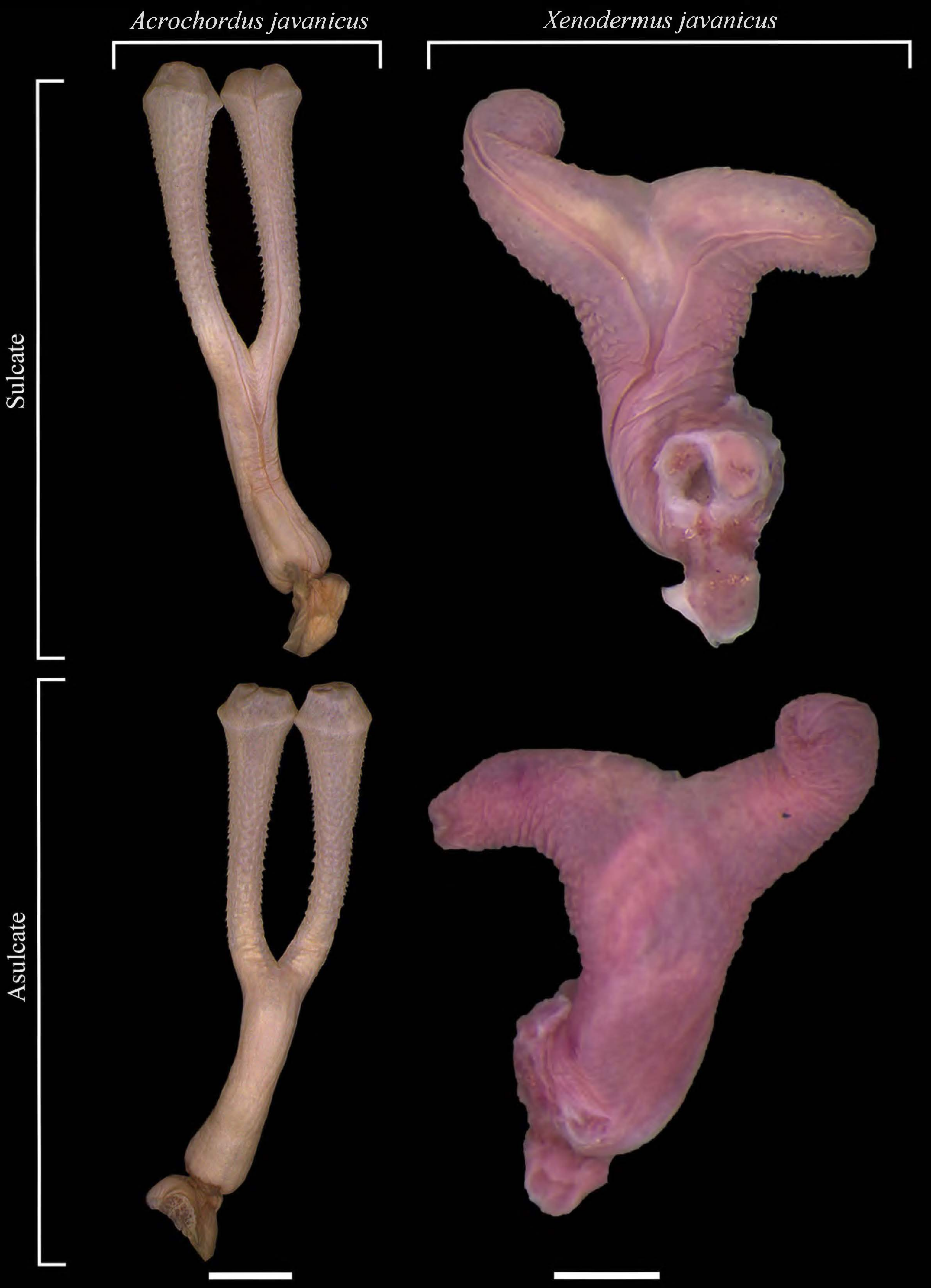
Acrochordidae
Acrochordus javanicus
Xenodermidae
Xenodermus javanicus
Sulcate
Asulcate

Fig. A

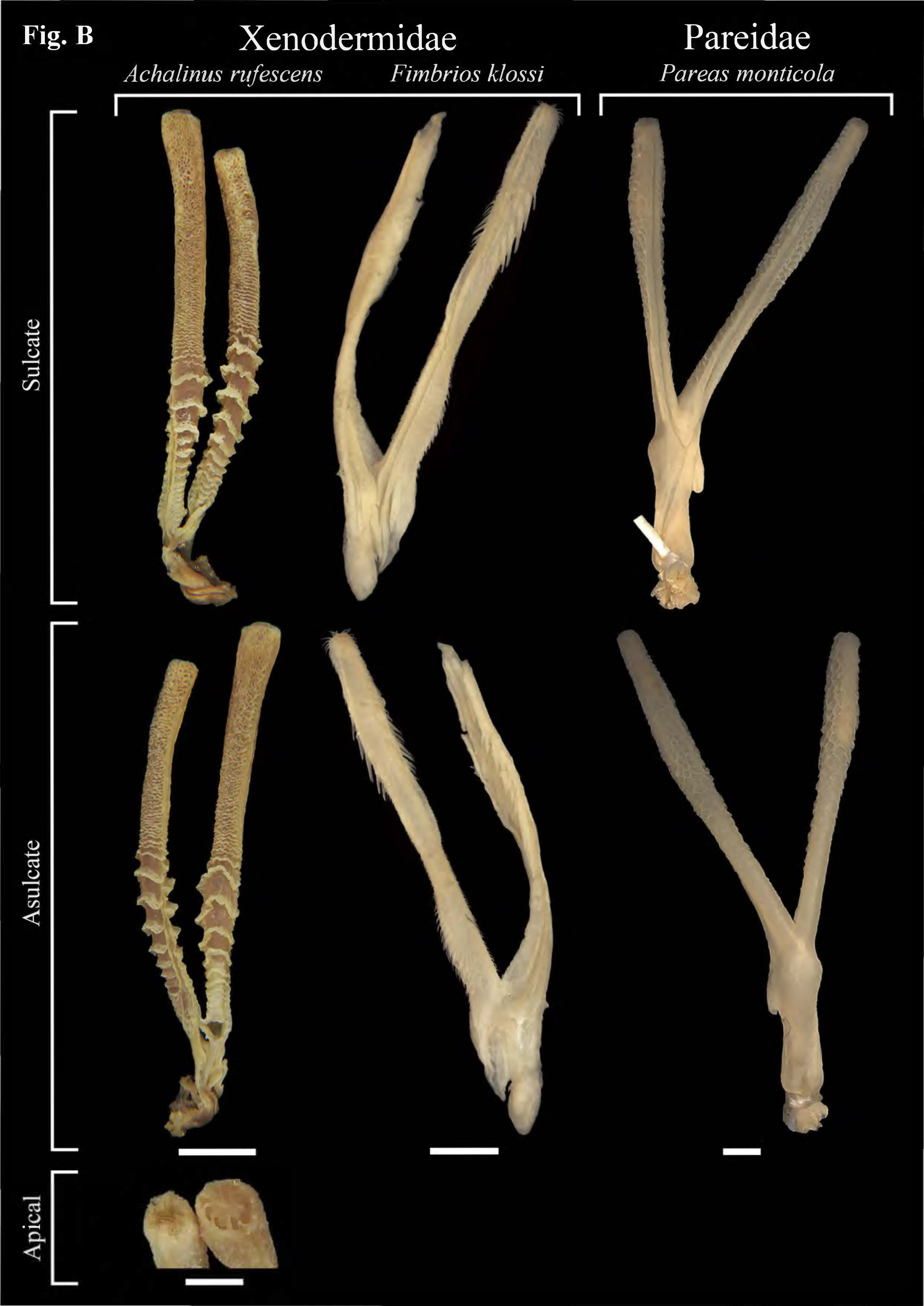
Fig. B
Xenodermidae
Pareidae
Achalinus rufescens
Fimbrios klossi
Pareas monticola
Sulcate
Asulcate
Apical

Fig. C

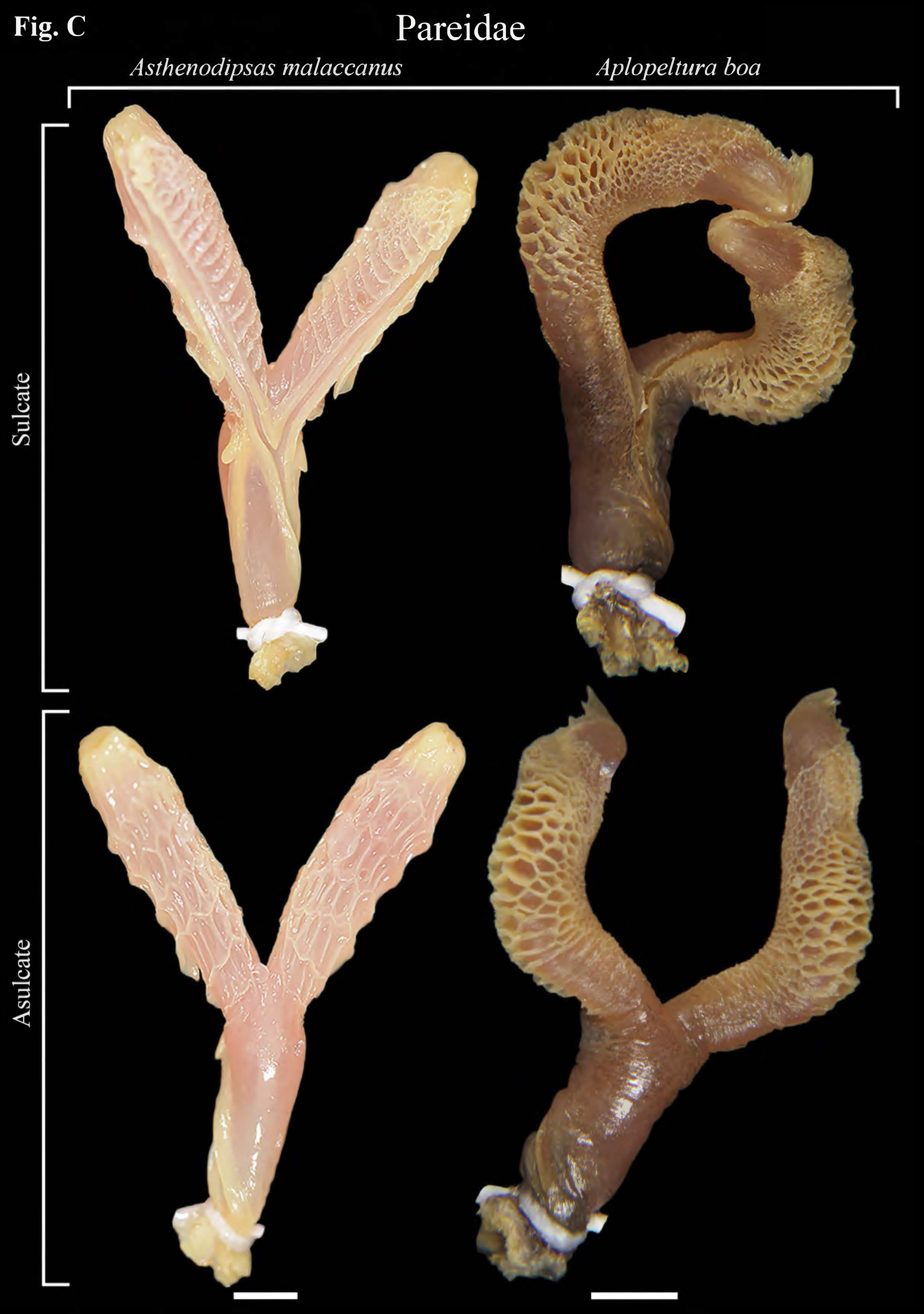
Pareidae
Asthenodipsas malaccanus
Aplopeltura boa
Sulcate
Asulcate

Fig. D

Xylophiidae

Xylophis perroteti

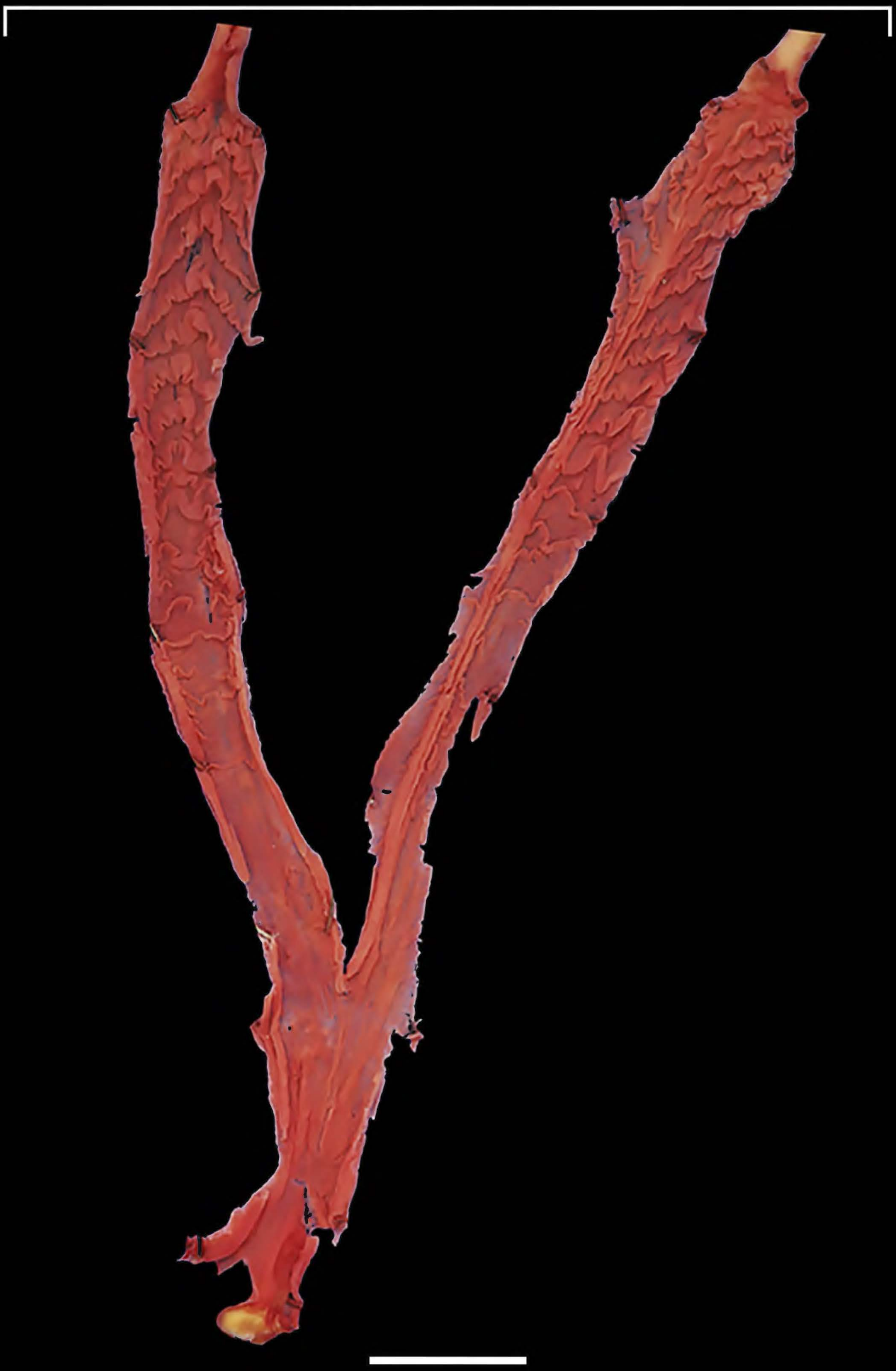

Fig. E

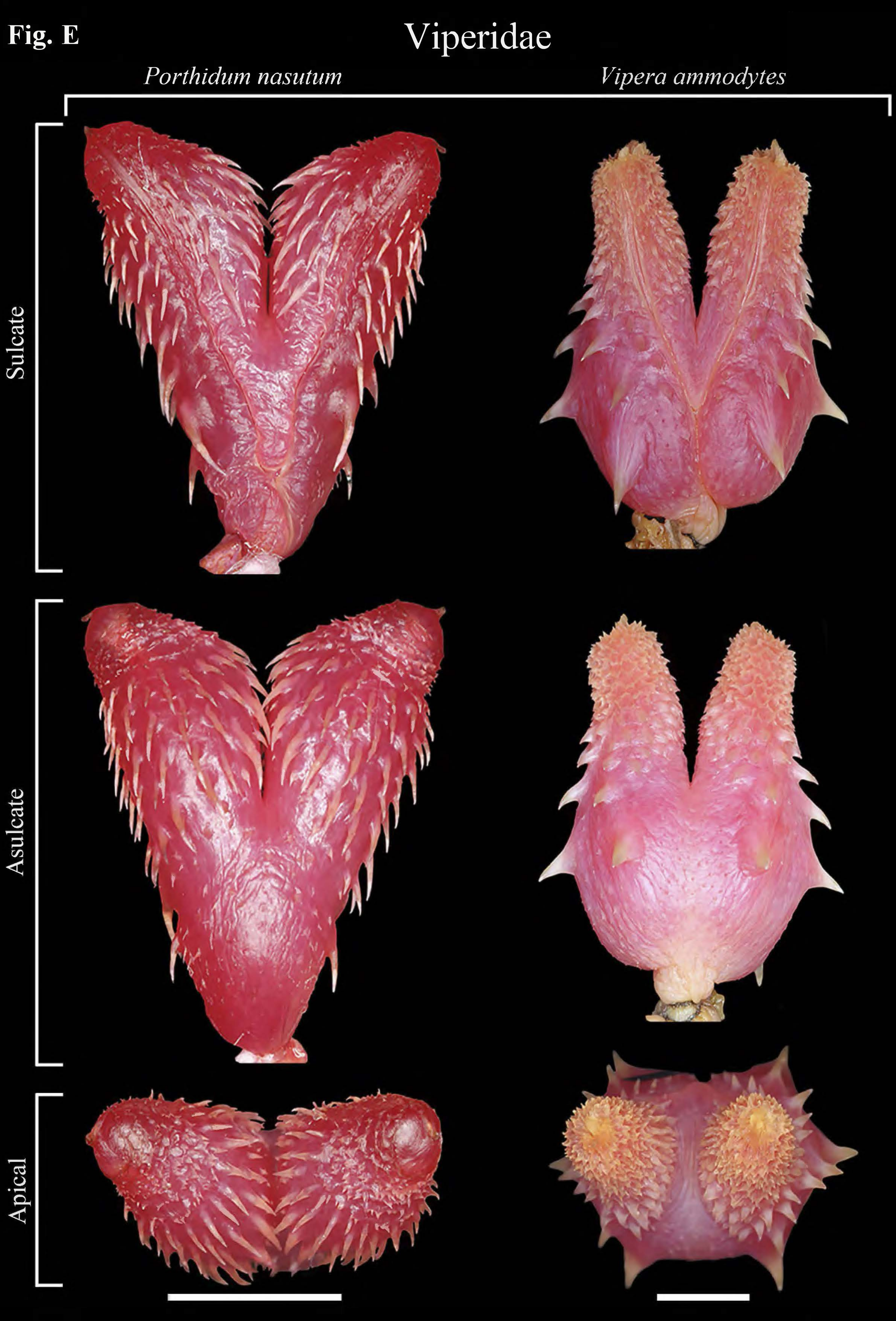

Viperidae
Porthidum nasutum
Vipera ammodytes
Sulcate
Asulcate
Apical

Fig. F

Viperidae

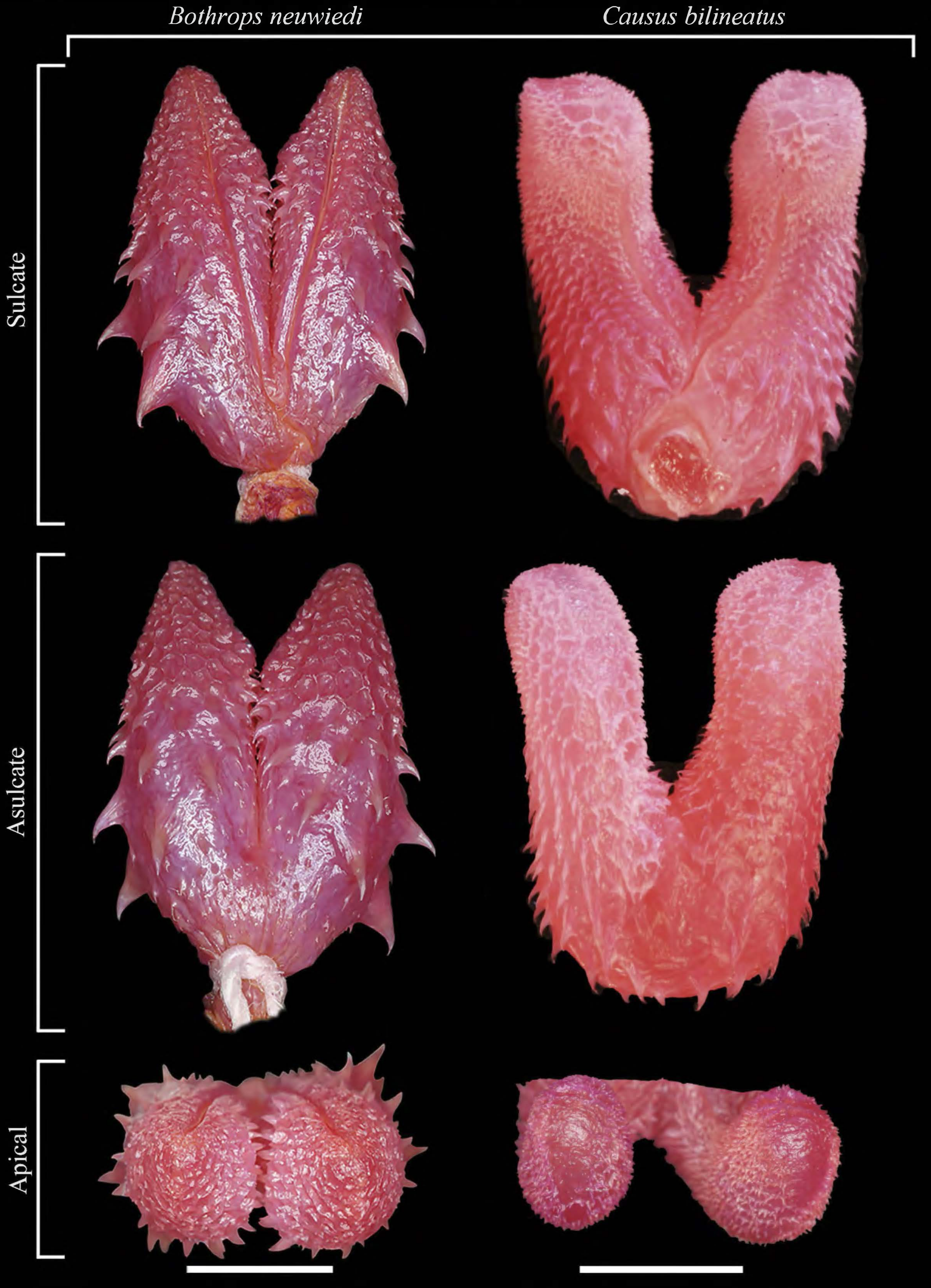

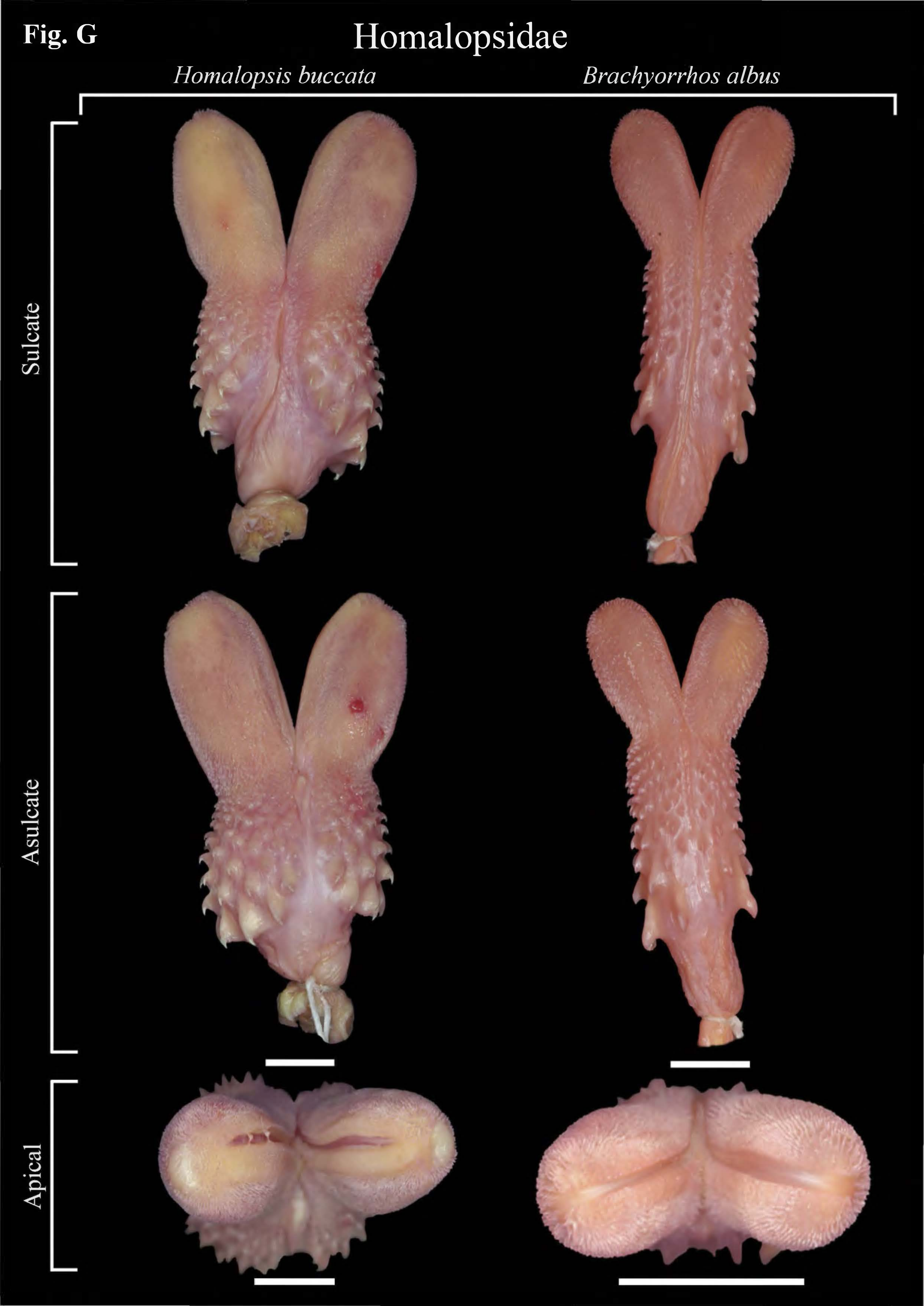
Fig. G
Homalopsidae
Homalopsis buccata
Brachyorrhos albus
Sulcate
Asulcate
Apical

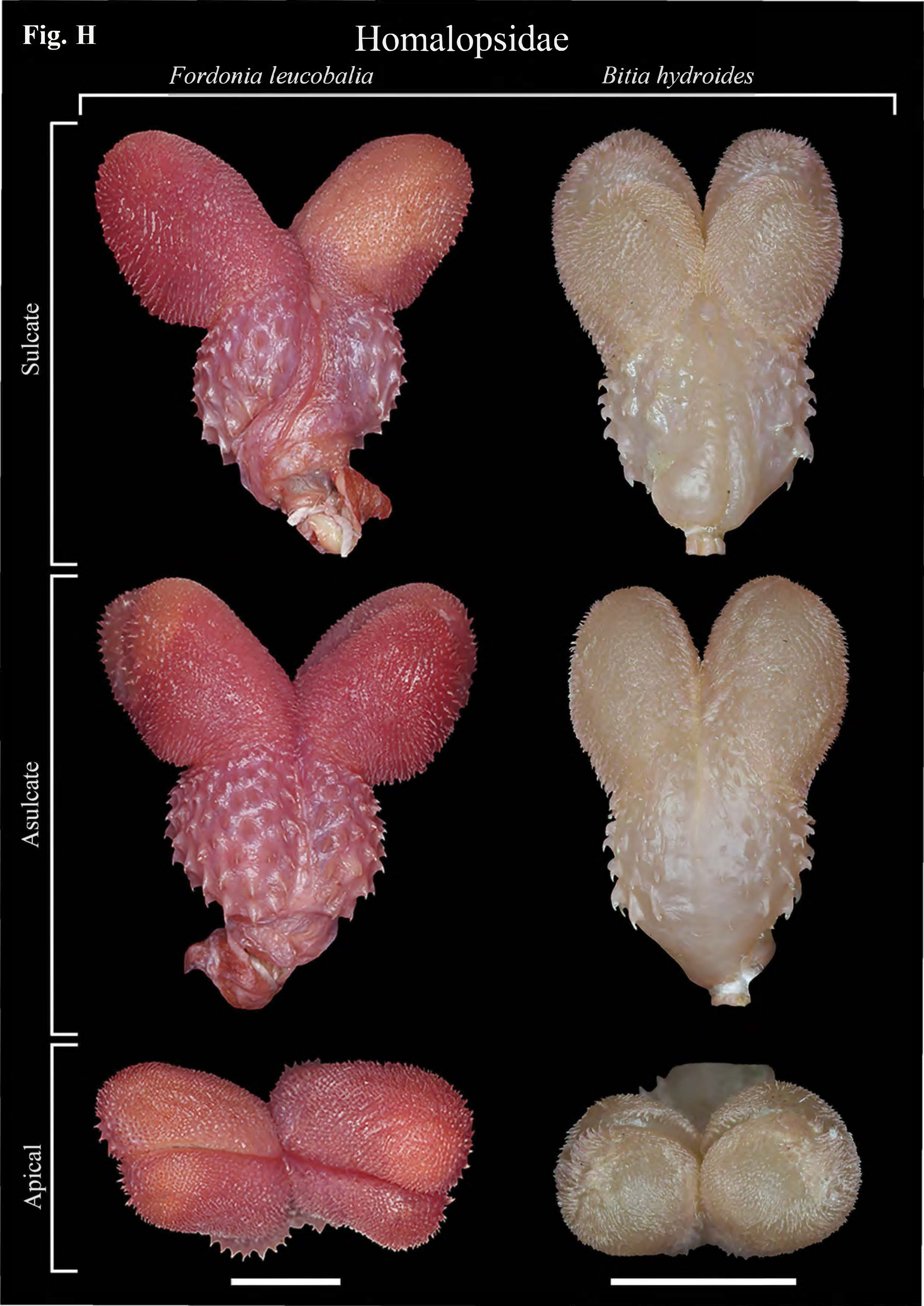
Fig. H
Homalopsidae
Fordonia leucobalia
Bitia hydroides
Sulcate
Asulcate
Apical

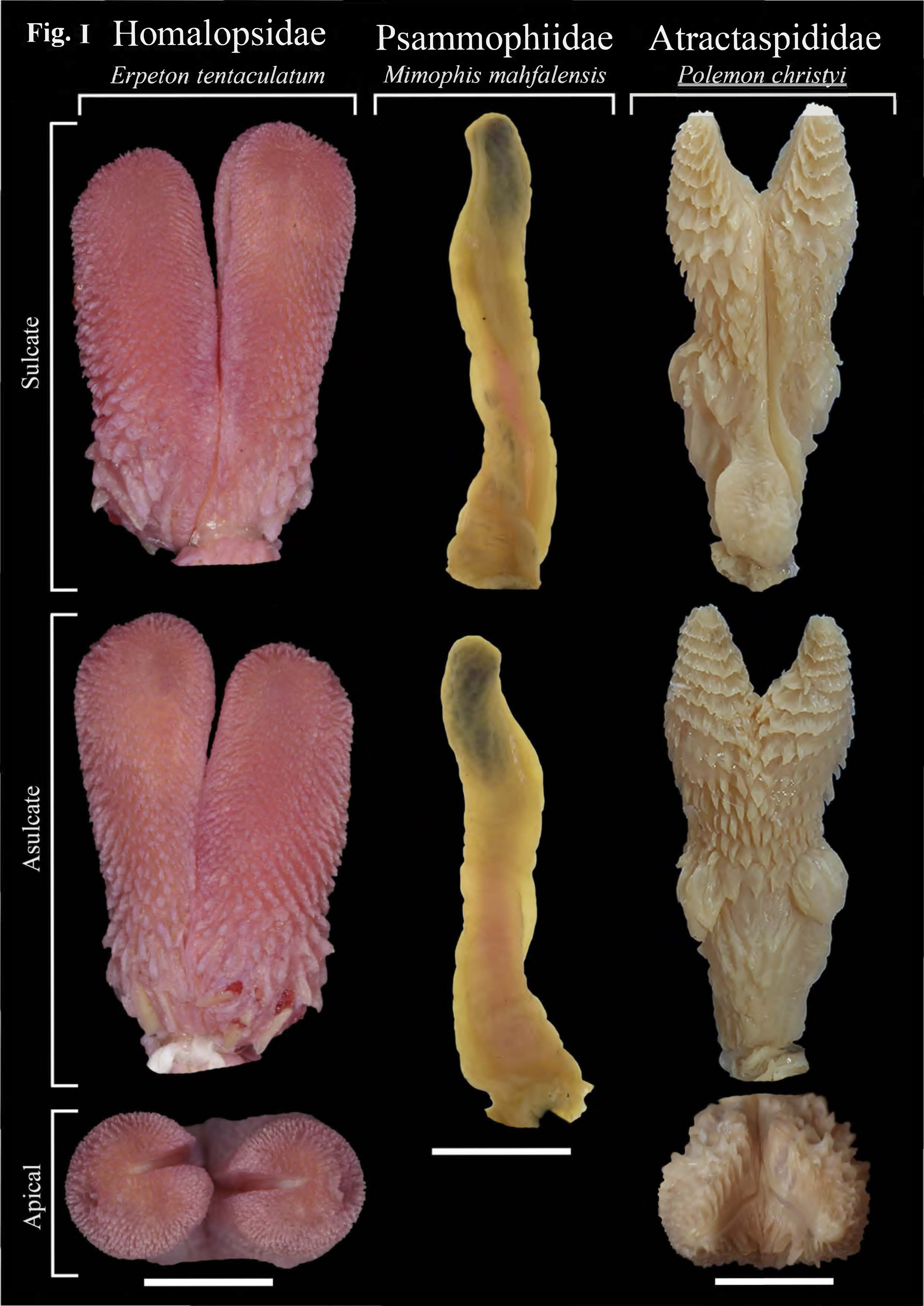
Fig. 1
Homalopsidae
Erpeton tentaculatum
Psammophiidae
Mimophis mahfalensis
Atractaspididae
Polemon christyi
Sulcate
Asulcate
Apical

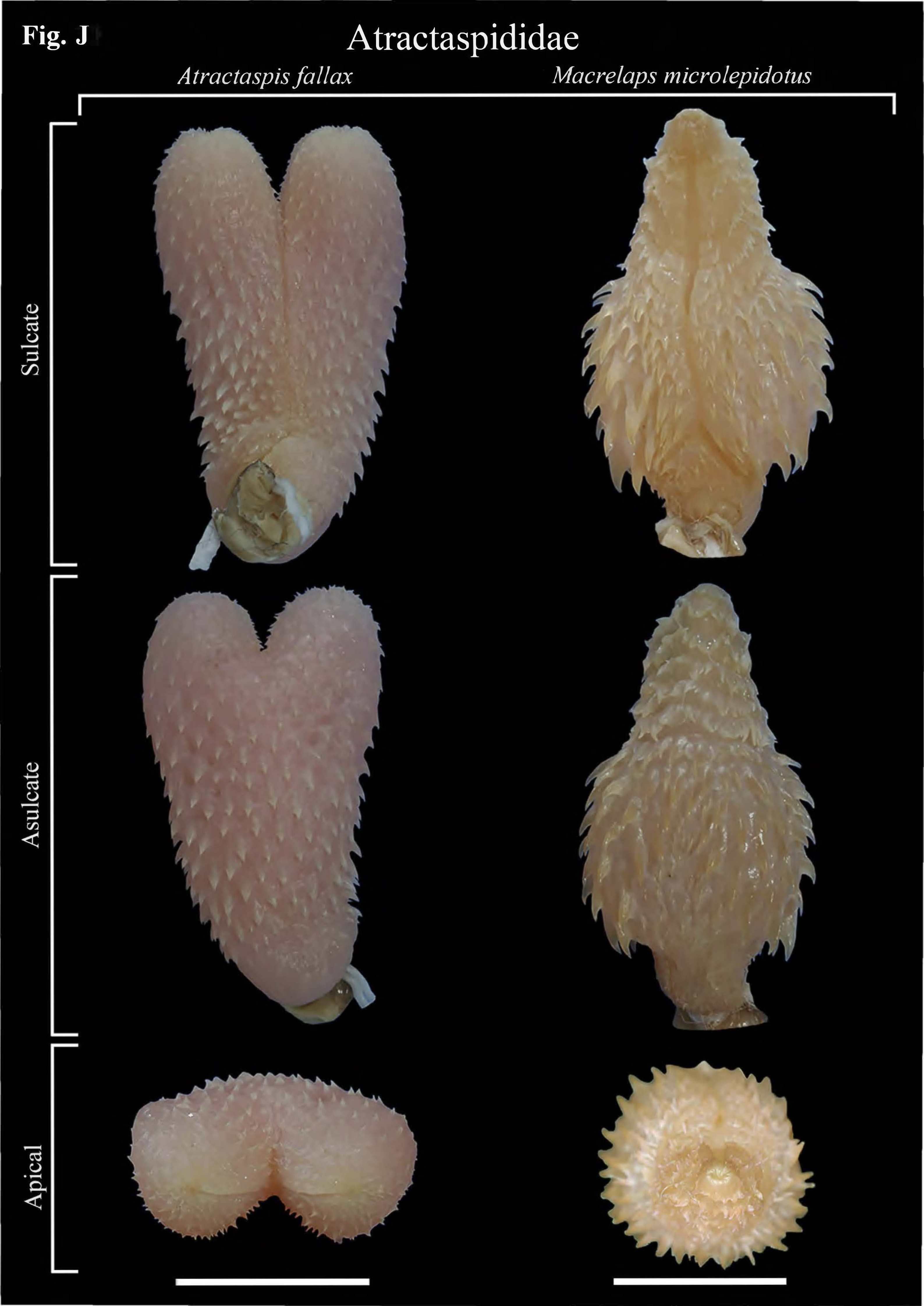
Fig. J
Atractaspididae
Atractaspis fallax
Macrelaps microlepidotus
Sulcate
Asulcate
Apical

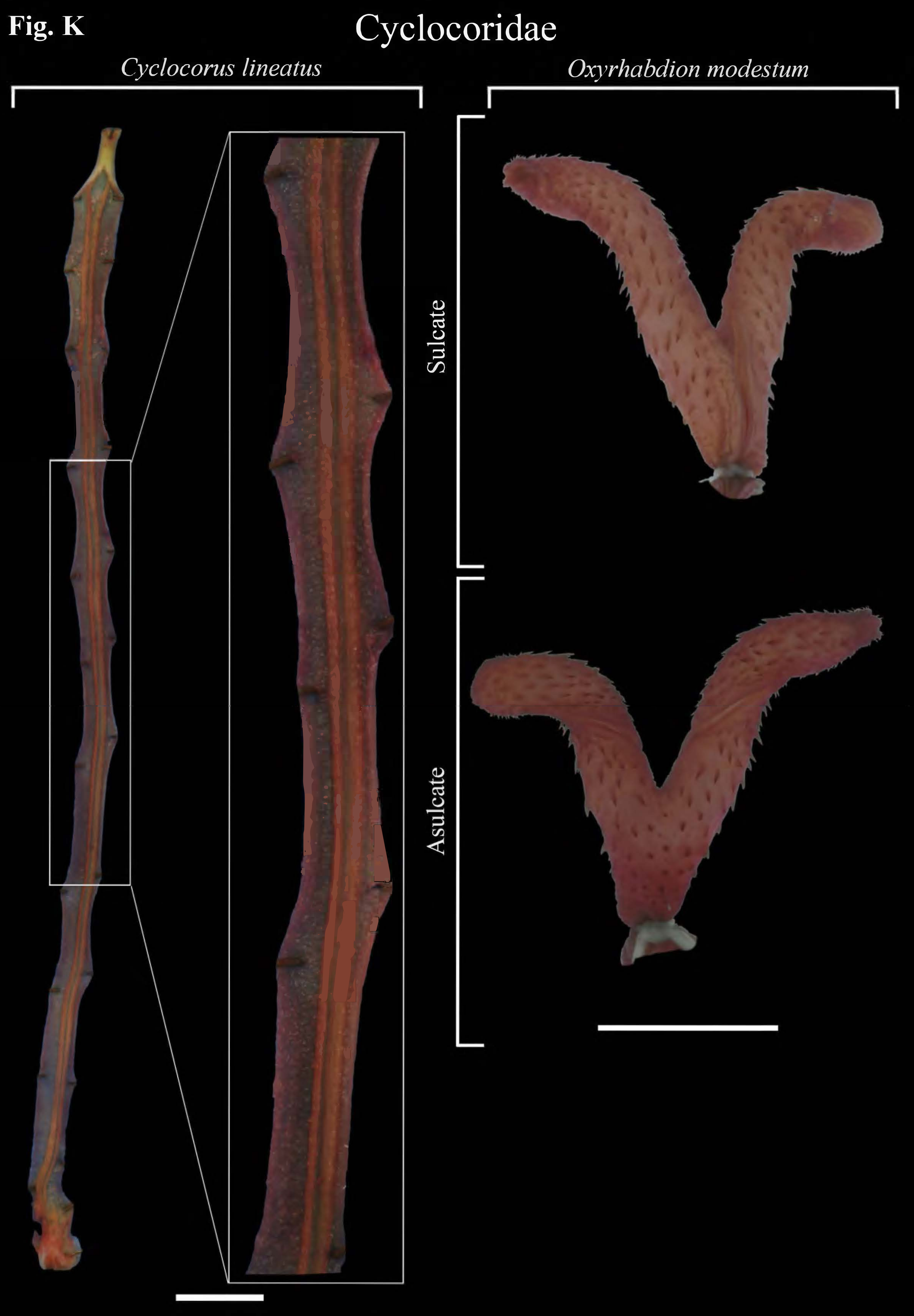
Fig. K
Cyclocoridae
Cyclocorus lineatus
Oxyrhabdion modestum
Sulcate
Asulcate

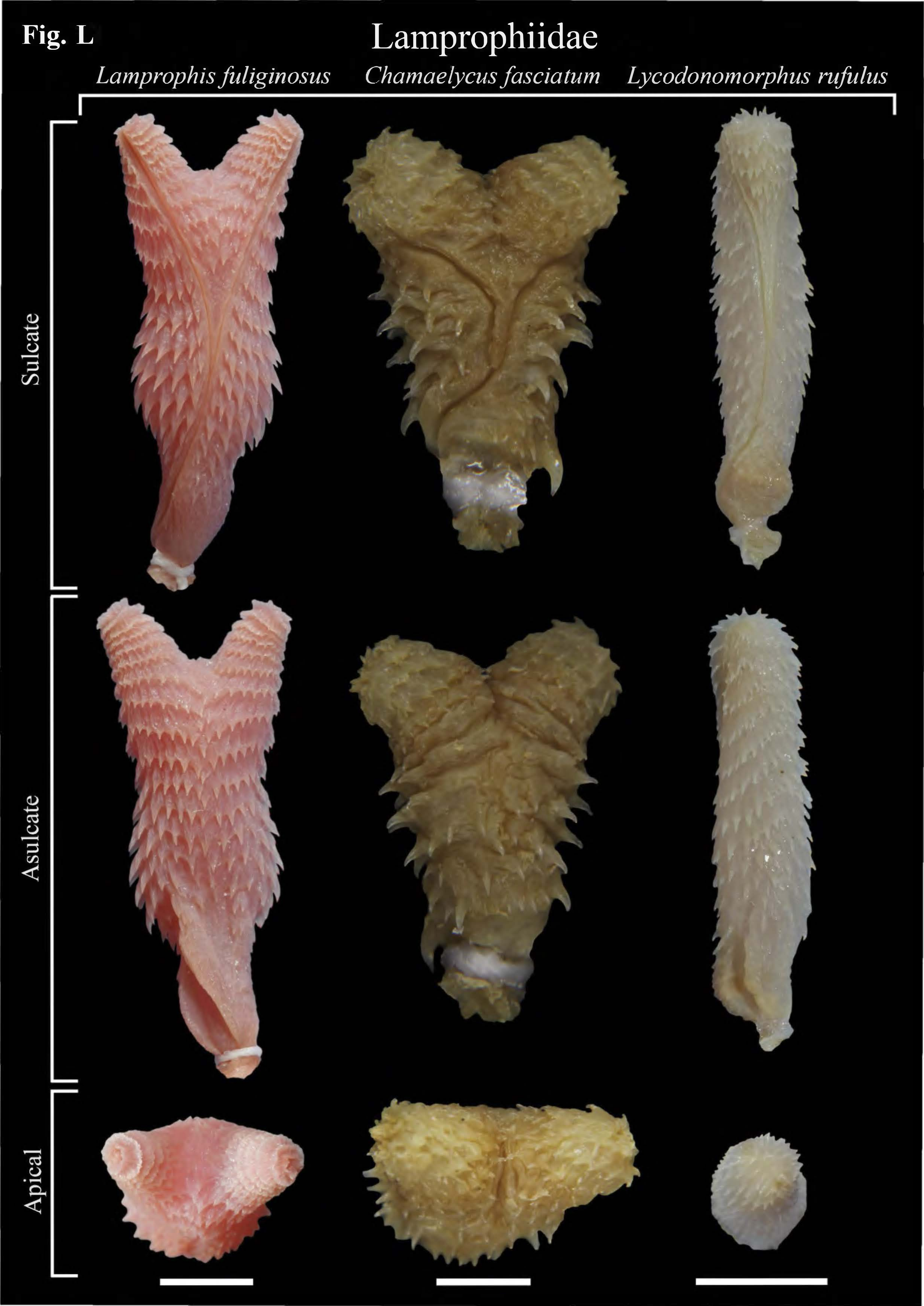
Fig. L
Lamprophiidae
Lamprophis fuliginosus
Chamaelycus fasciatum
Lycodonomorphus rufulus
Sulcate
Asulcate
Apical

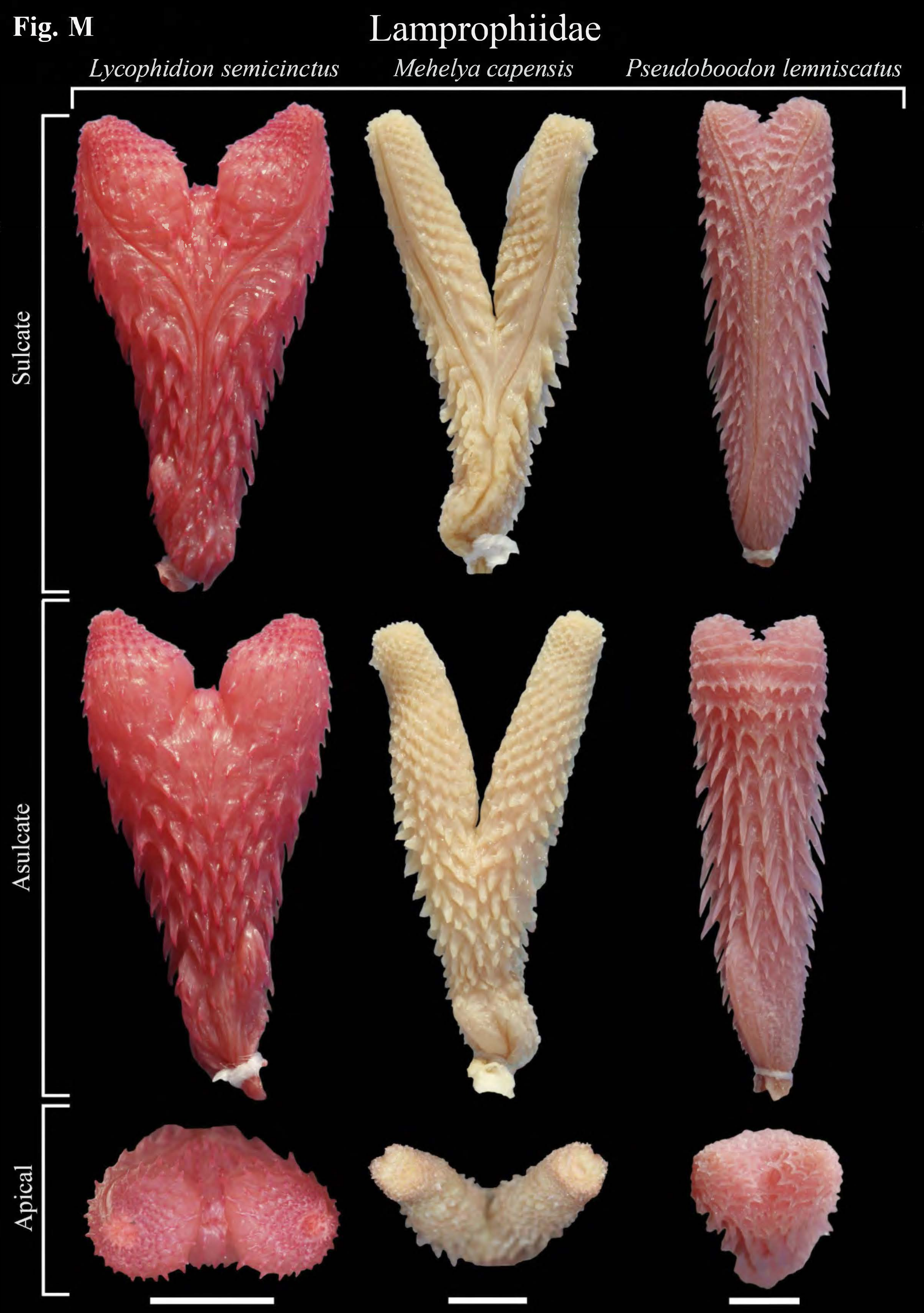
Fig. M
Lamprophiidae
Lycophidion semicinctus
Mehelya capensis
Pseudoboodon lemniscatus
Sulcate
Asulcate
Apical

Fig. N

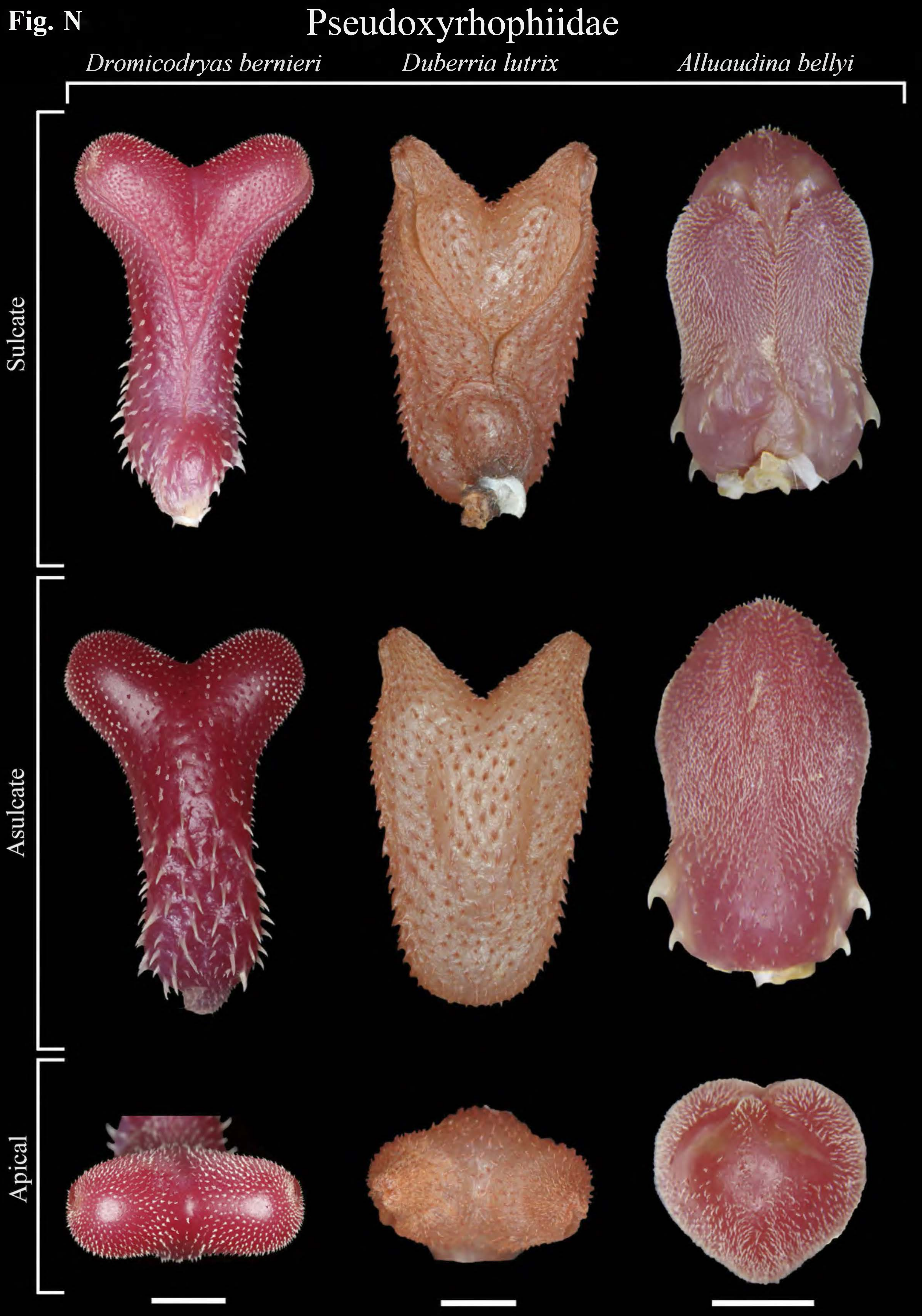
Pseudoxyrhophiidae
Dromicodryas bernieri
Duberria lutrix
Alluaudina bellyi
Sulcate
Asulcate
Apical

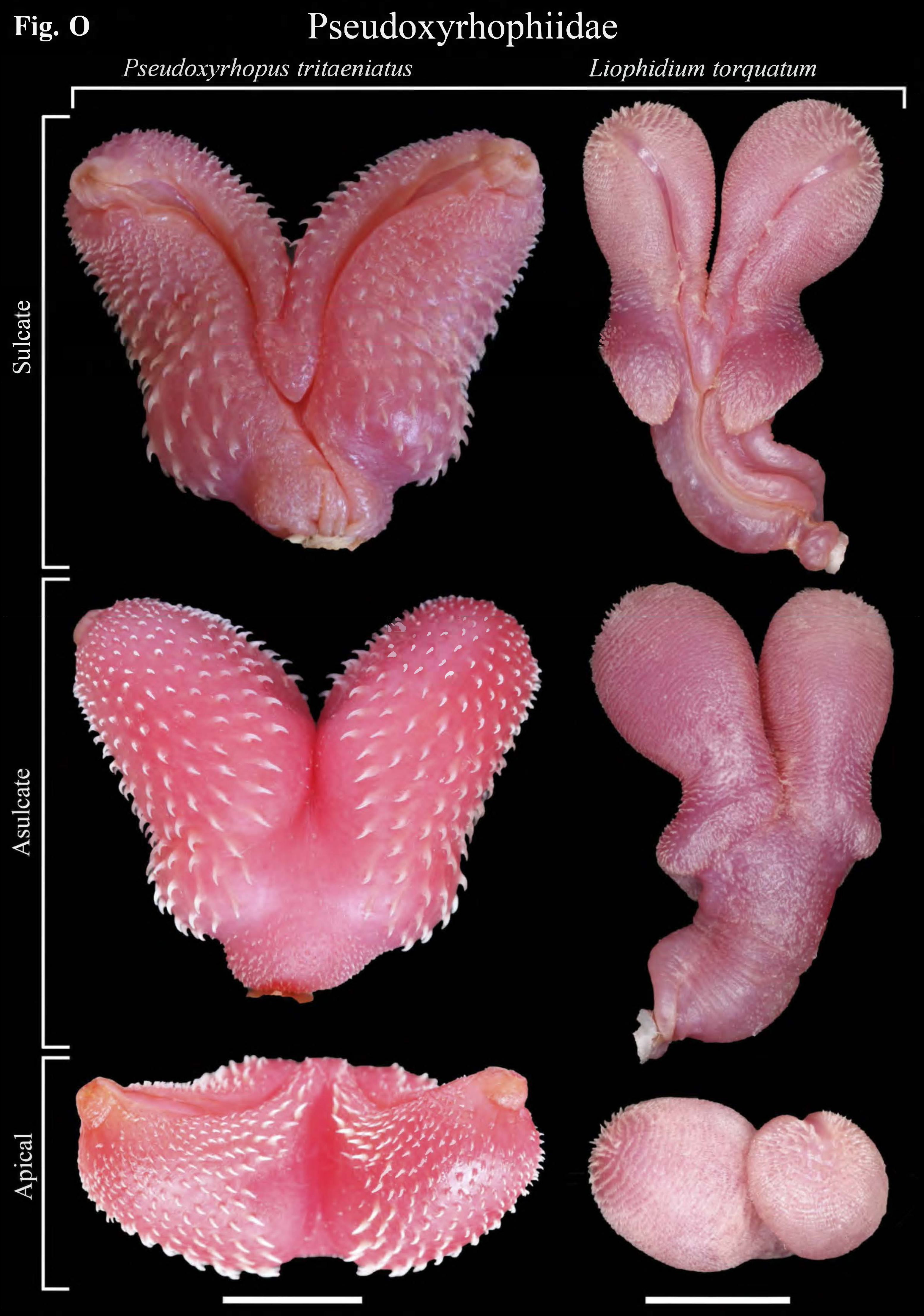
Fig. O
Pseudoxyrhophiidae
Pseudoxyrhopus tritaeniatus
Liophidium torquatum
Sulcate
Asulcate
Apical

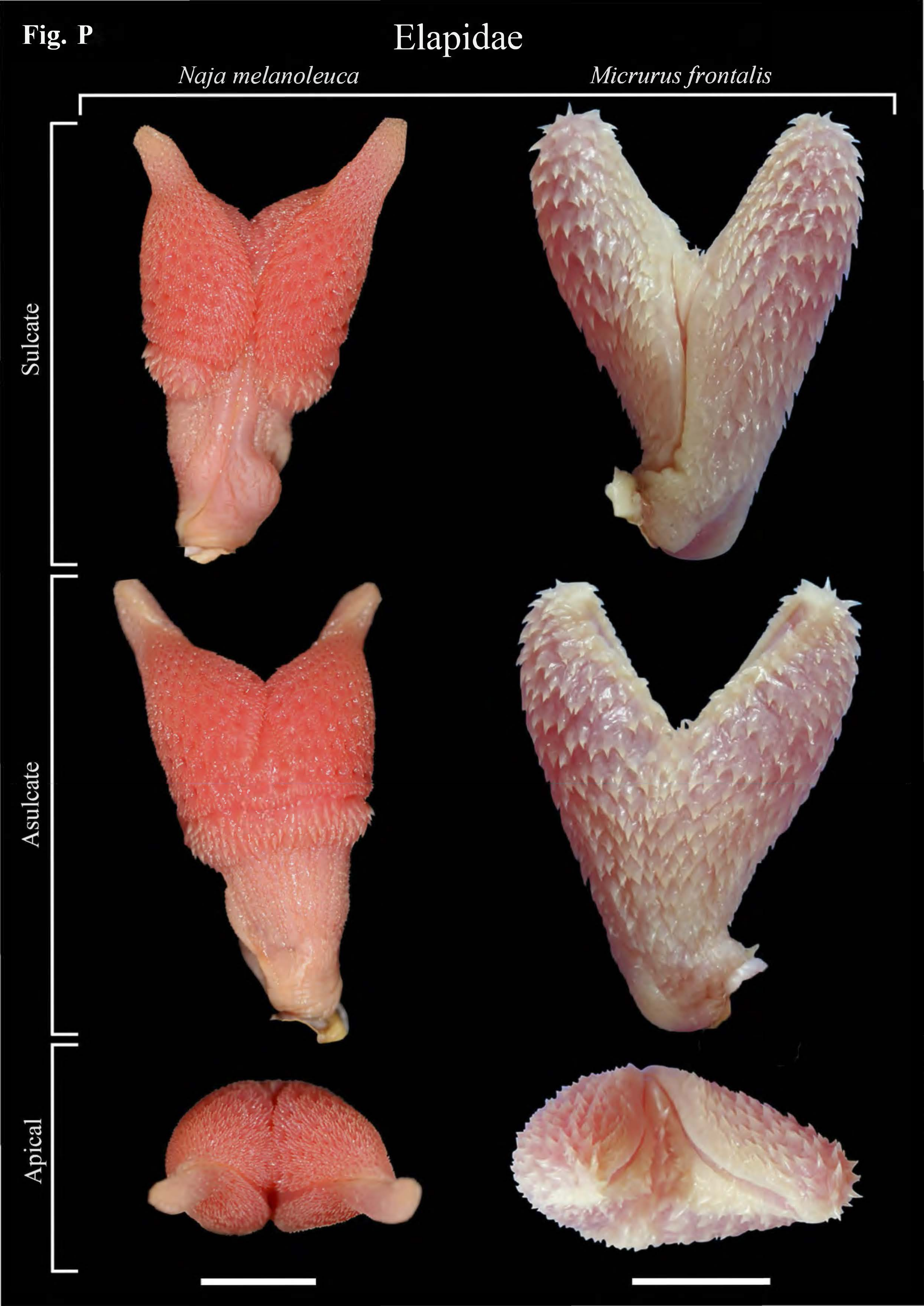

Fig. P
Elapidae
Naja melanoleuca
Micrurus frontalis
Sulcate
Asulcate
Apical

Fig. Q

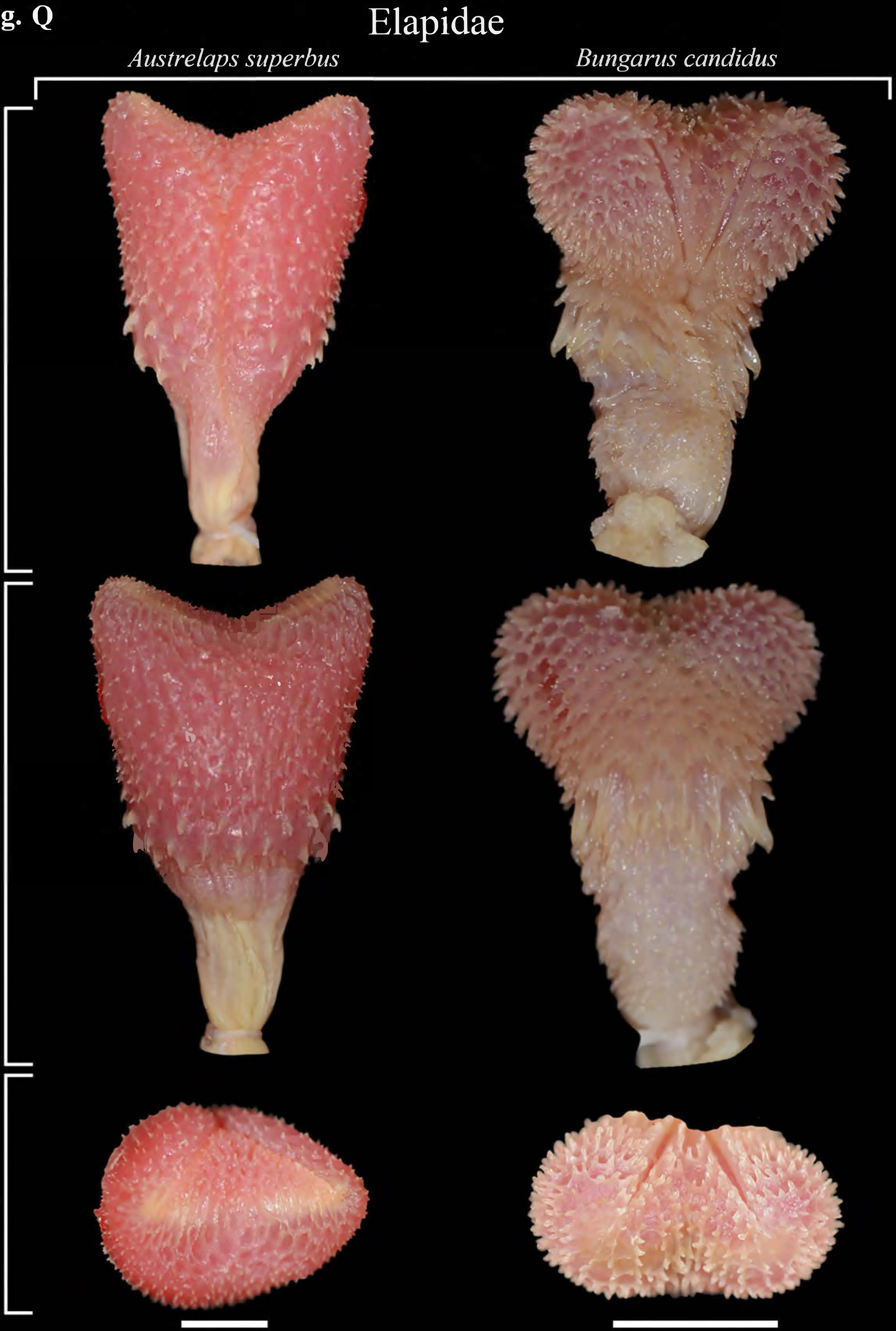
Elapidae
Austrelaps superbus
Bungarus candidus
Sulcate
Asulcate
Apical

Fig. R

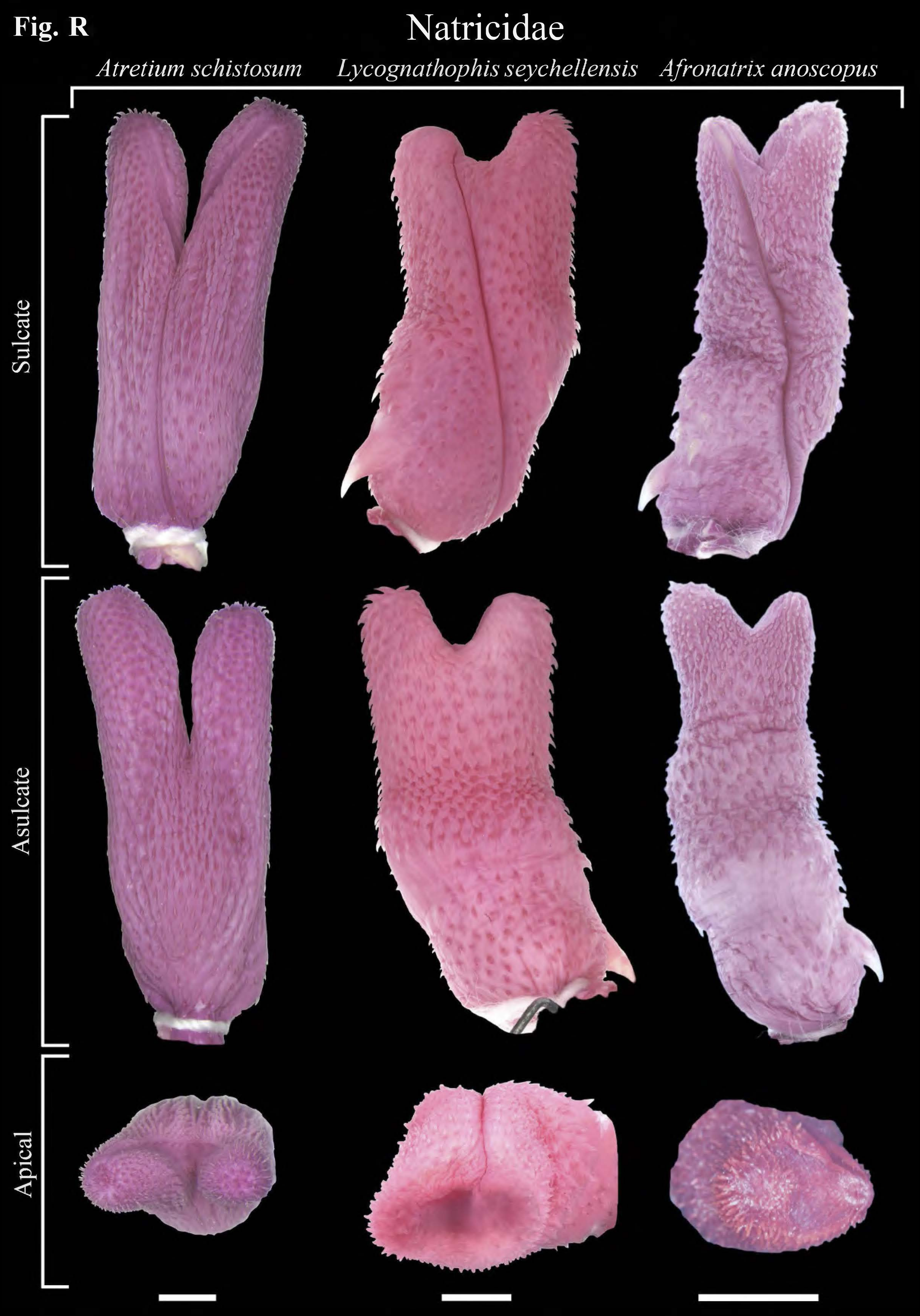
Natricidae
Atretium schistosum
Lycognathophis seychellensis
Afronatrix anoscopus
Sulcate
Asulcate
Apical

Fig. S

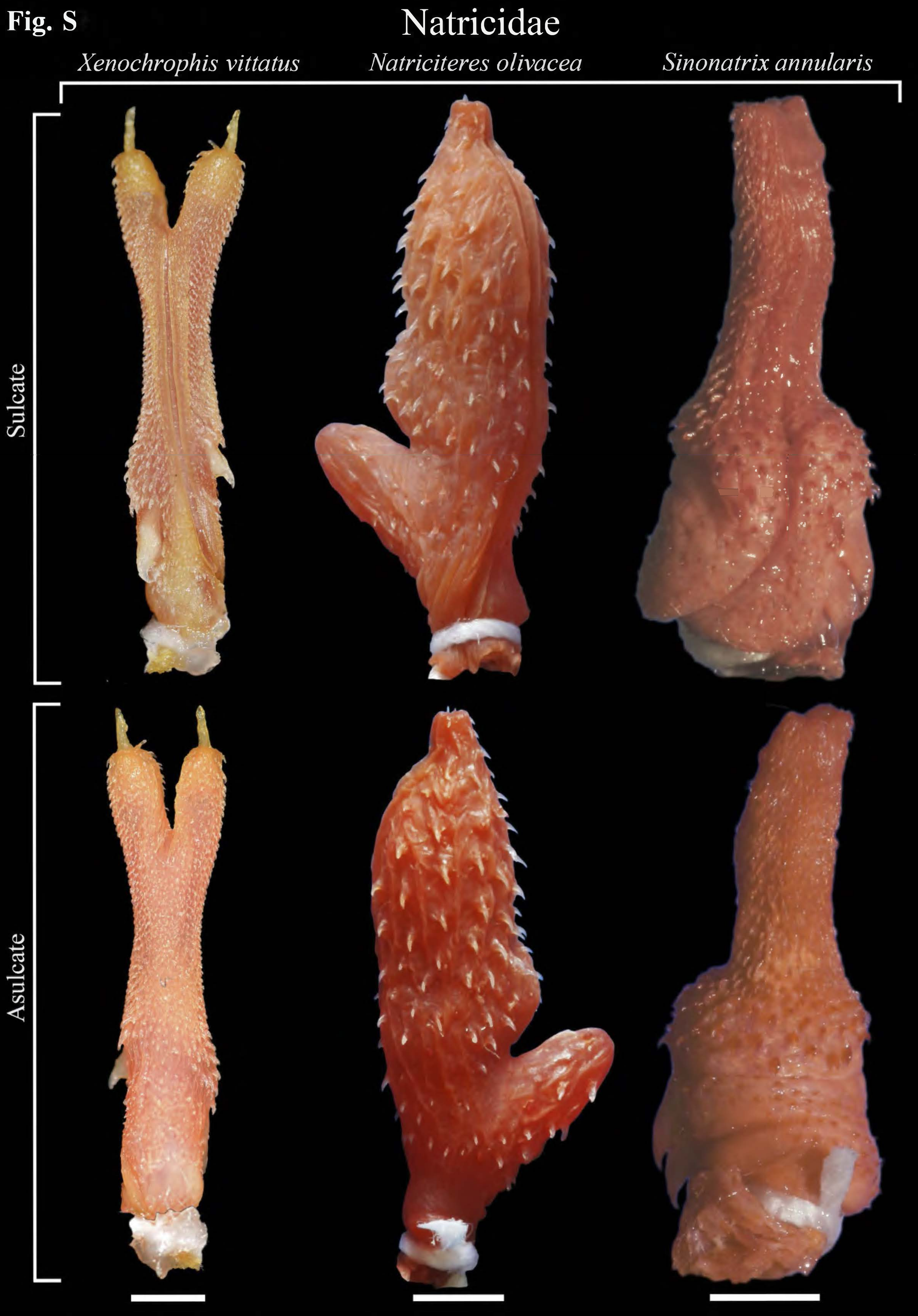

Natricidae
Xenochrophis vittatus
Natriciteres olivacea
Sinonatrix annularis
Sulcate
Asulcate

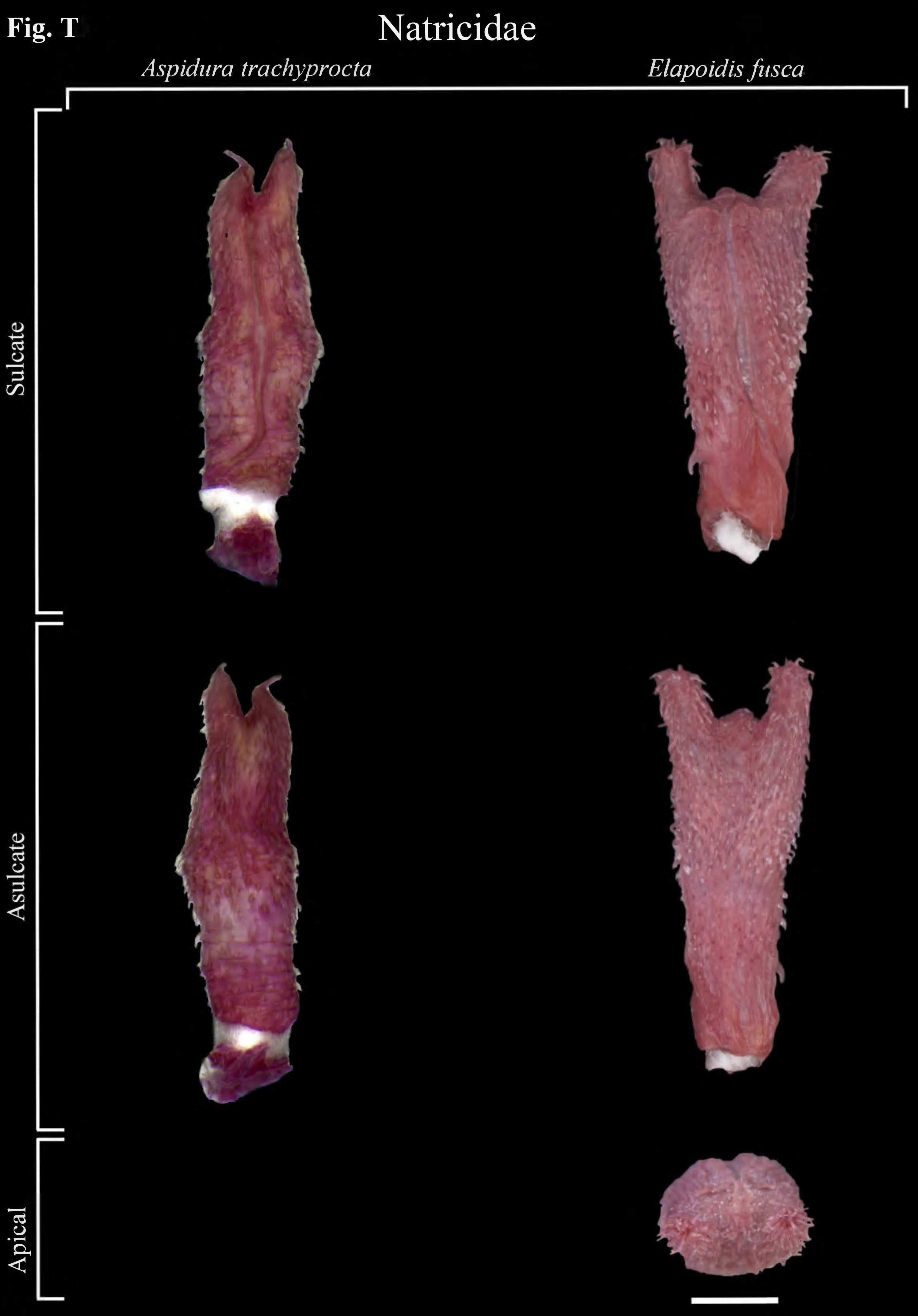
Fig. T
Natricidae
Aspidura trachyprocta
Elapoidis fusca
Sulcate
Asulcate
Apical

Fig. U

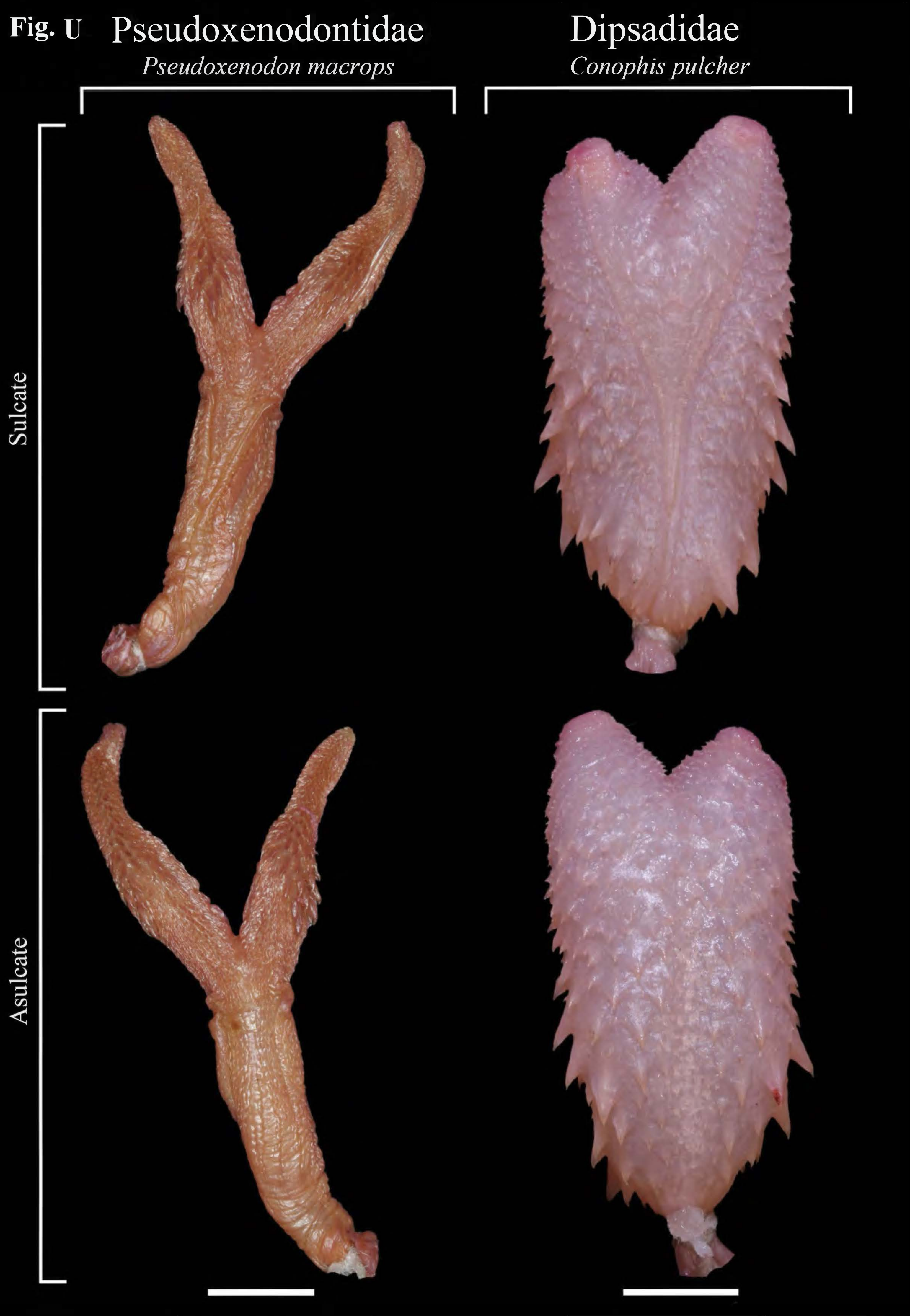
Pseudoxenodontidae
Pseudoxenodon macrops
Dipsadidae
Conophis pulcher
Sulcate
Asulcate

Fig. V

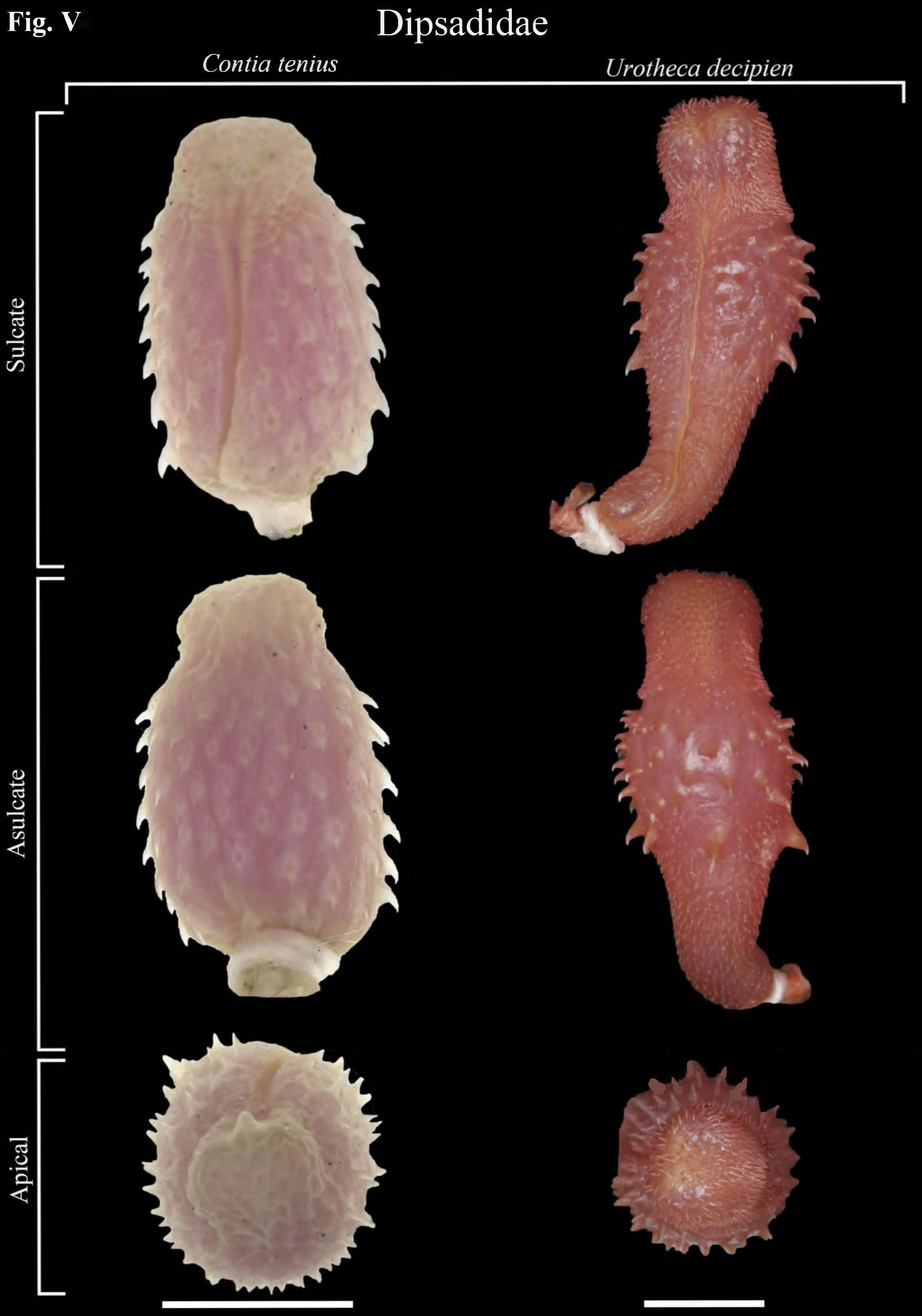
Dipsadidae
Contia tenius
Urotheca decipien
Sulcate
Asulcate
Apical

Fig. W Dipsadidae

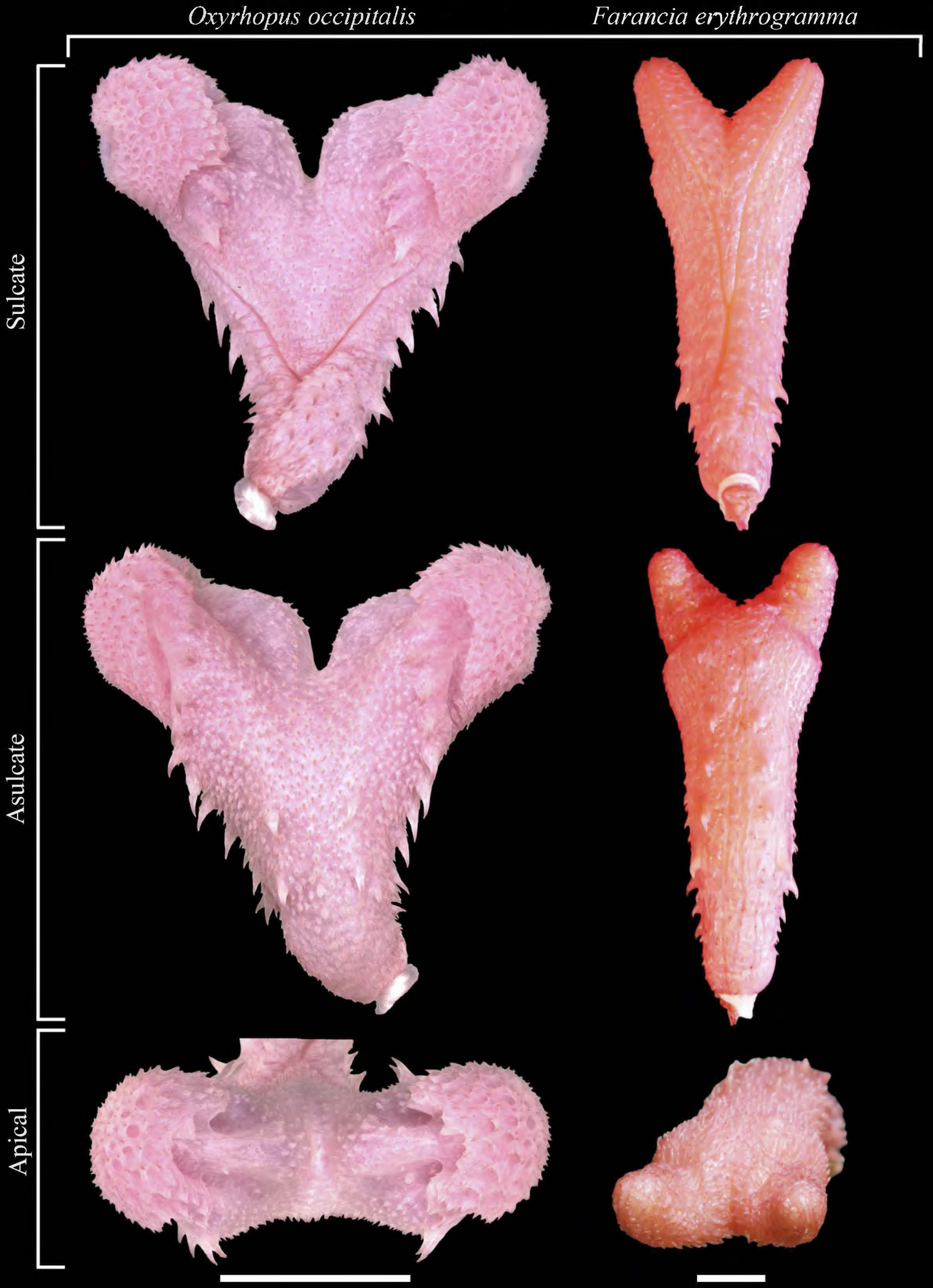

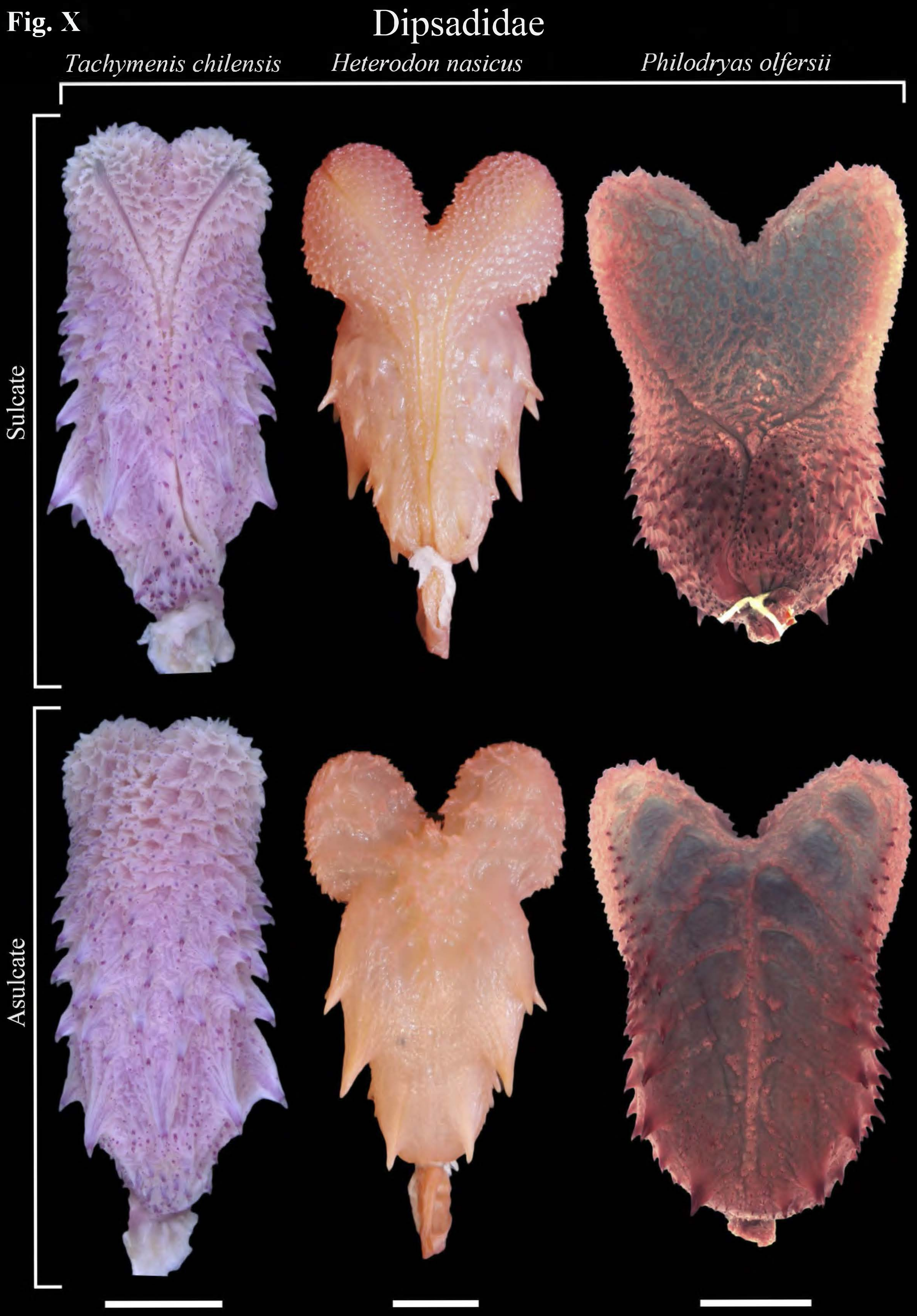
Fig. X
Dipsadidae
Tachymenis chilensis
Heterodon nasicus
Philodryas olfersii
Sulcate
Asulcate

Fig. Y

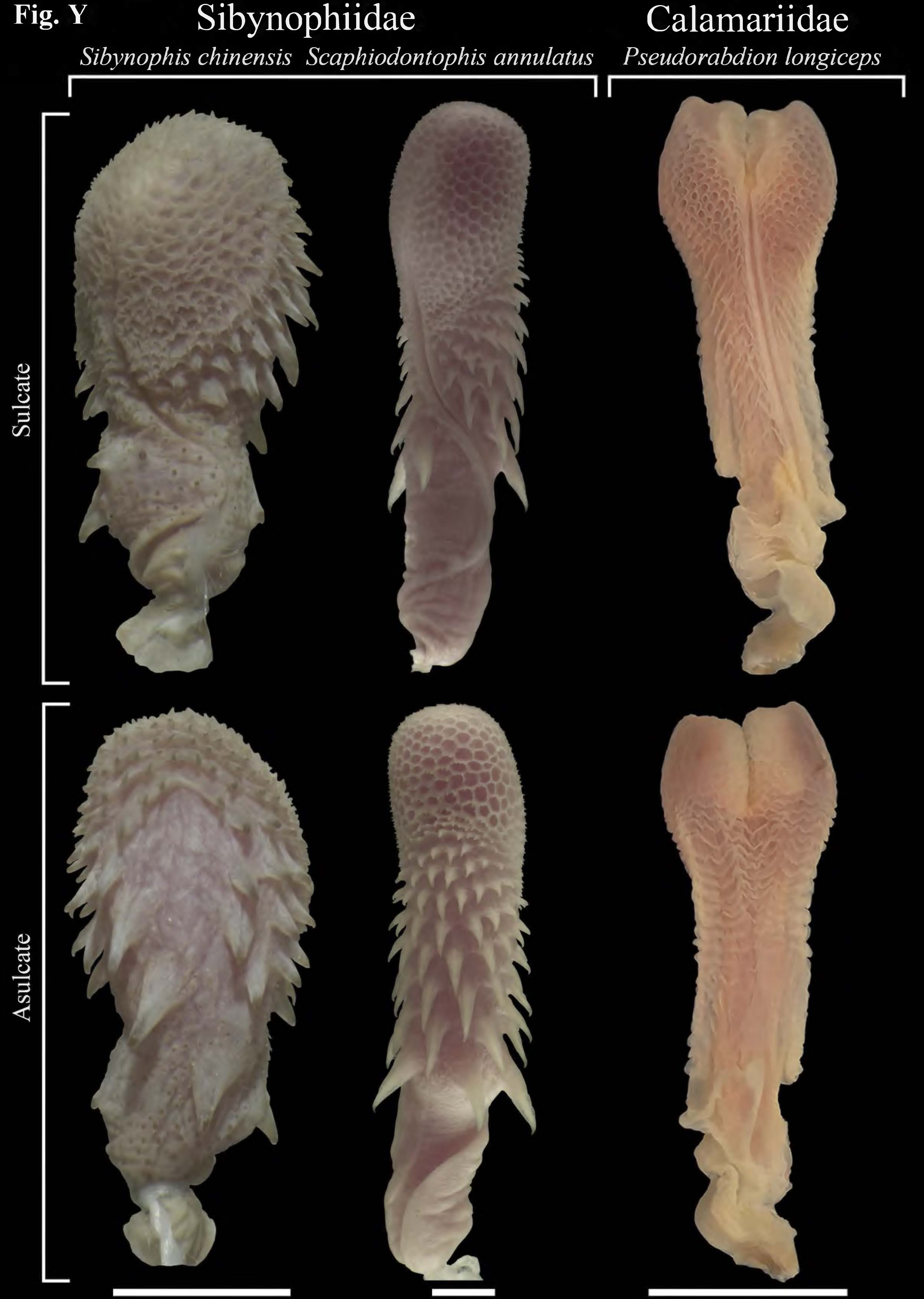
Sibynophiidae
Calamariidae
Sibynophis chinensis
Scaphiodontophis annulatus
Pseudorabdion longiceps
Sulcate
Asulcate

Fig. Z

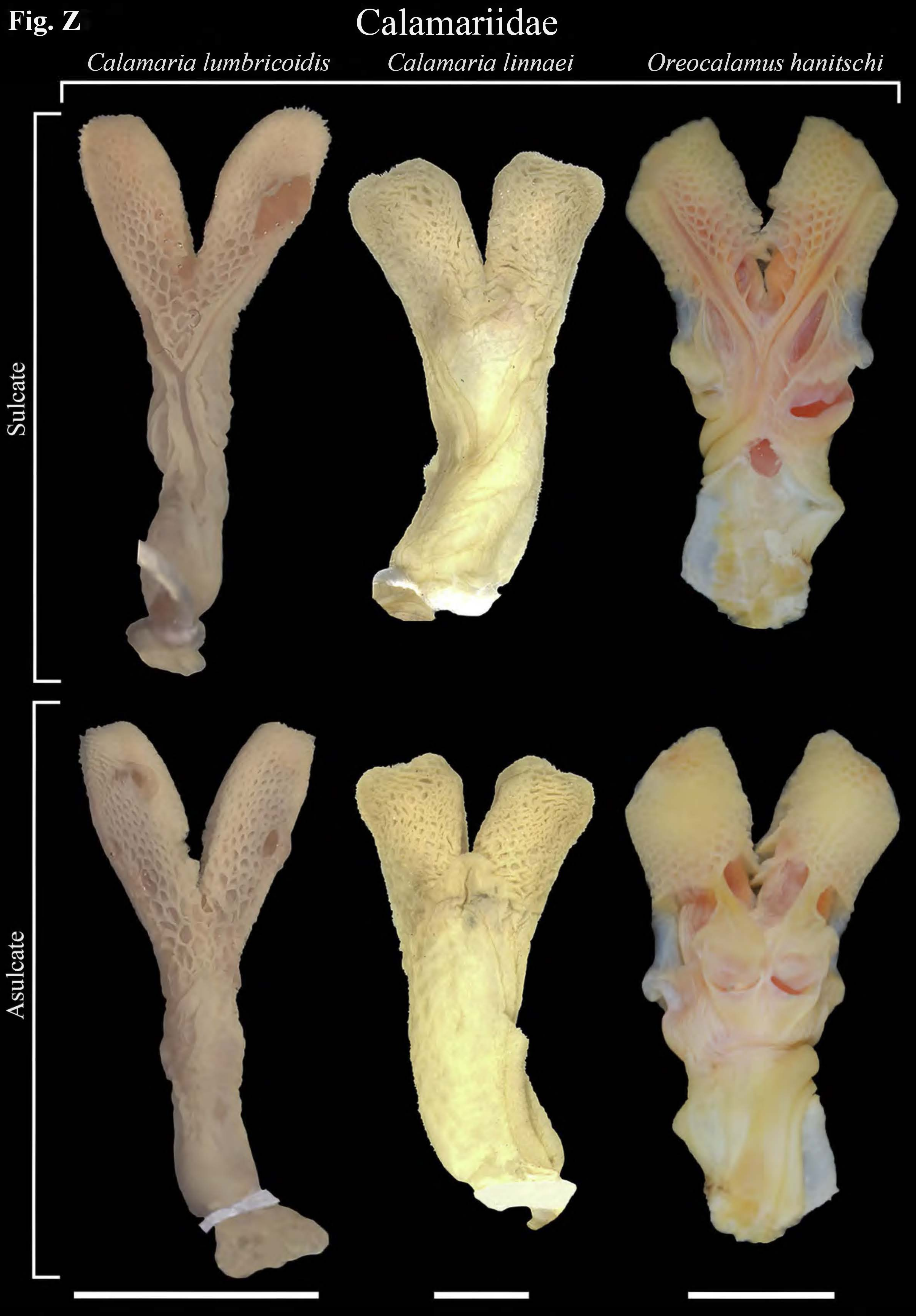
Calamariidae
Calamaria lumbricoidis
Calamaria linnaei
Oreocalamus hanitschi
Sulcate
Asulcate

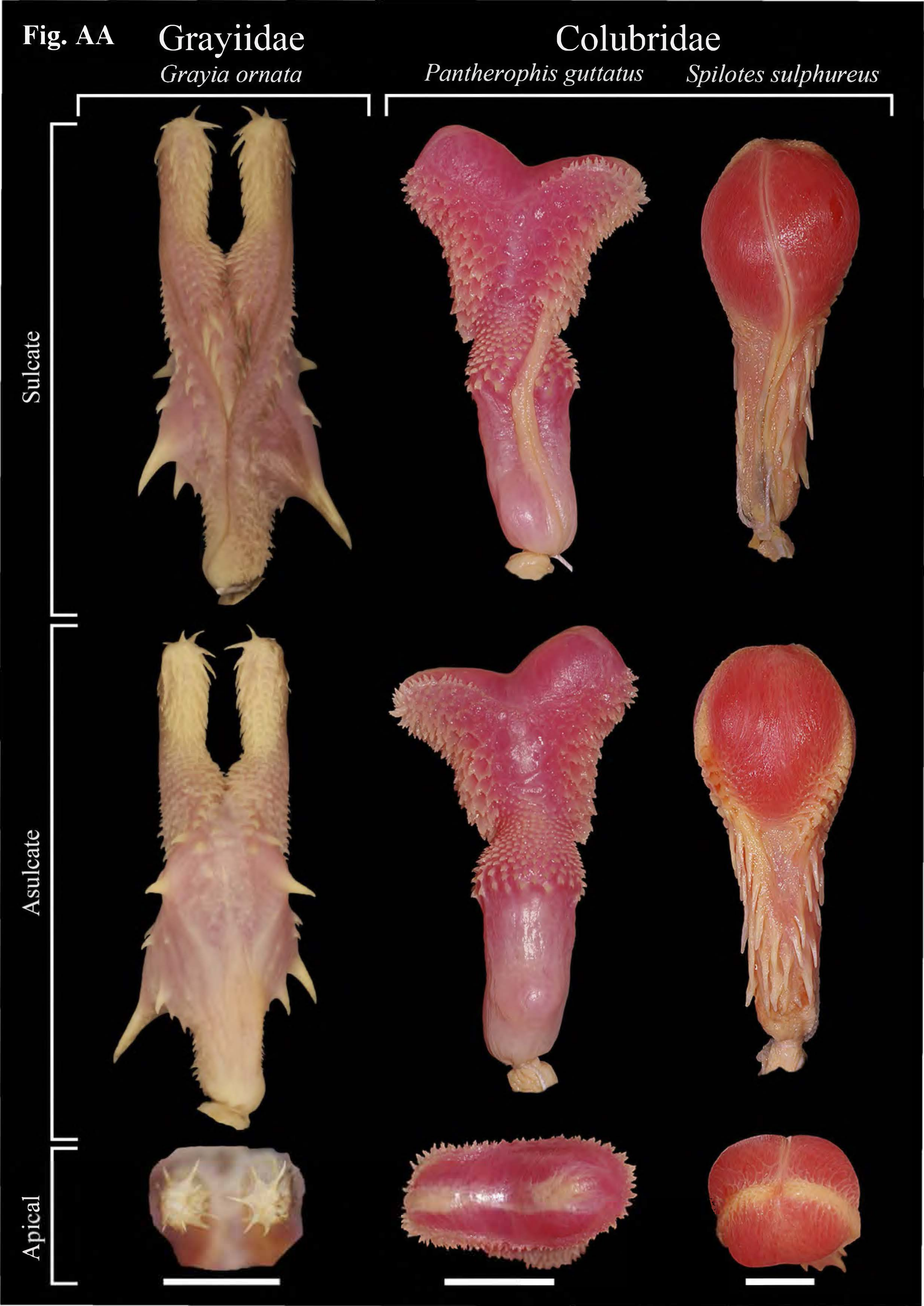
Fig. AA
Grayiidae
Grayia ornata
Colubridae
Pantherophis guttatus
Spilotes sulphureus
Sulcate
Asulcate
Apical

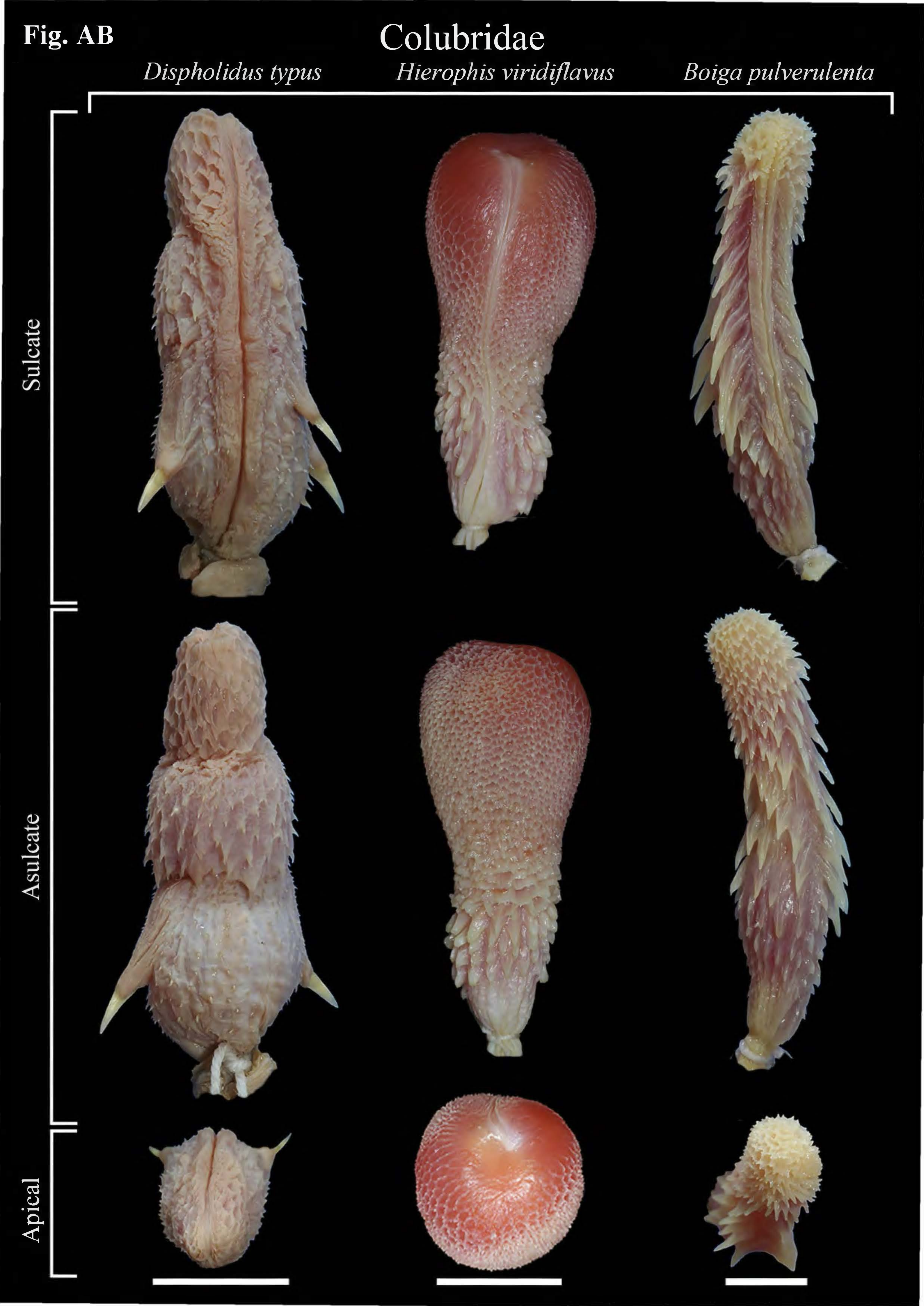
Fig. AB
Colubridae
Dispholidus typus
Hierophis viridiflavus
Boiga pulverulenta
Sulcate
Asulcate
Apical

Fig. AC

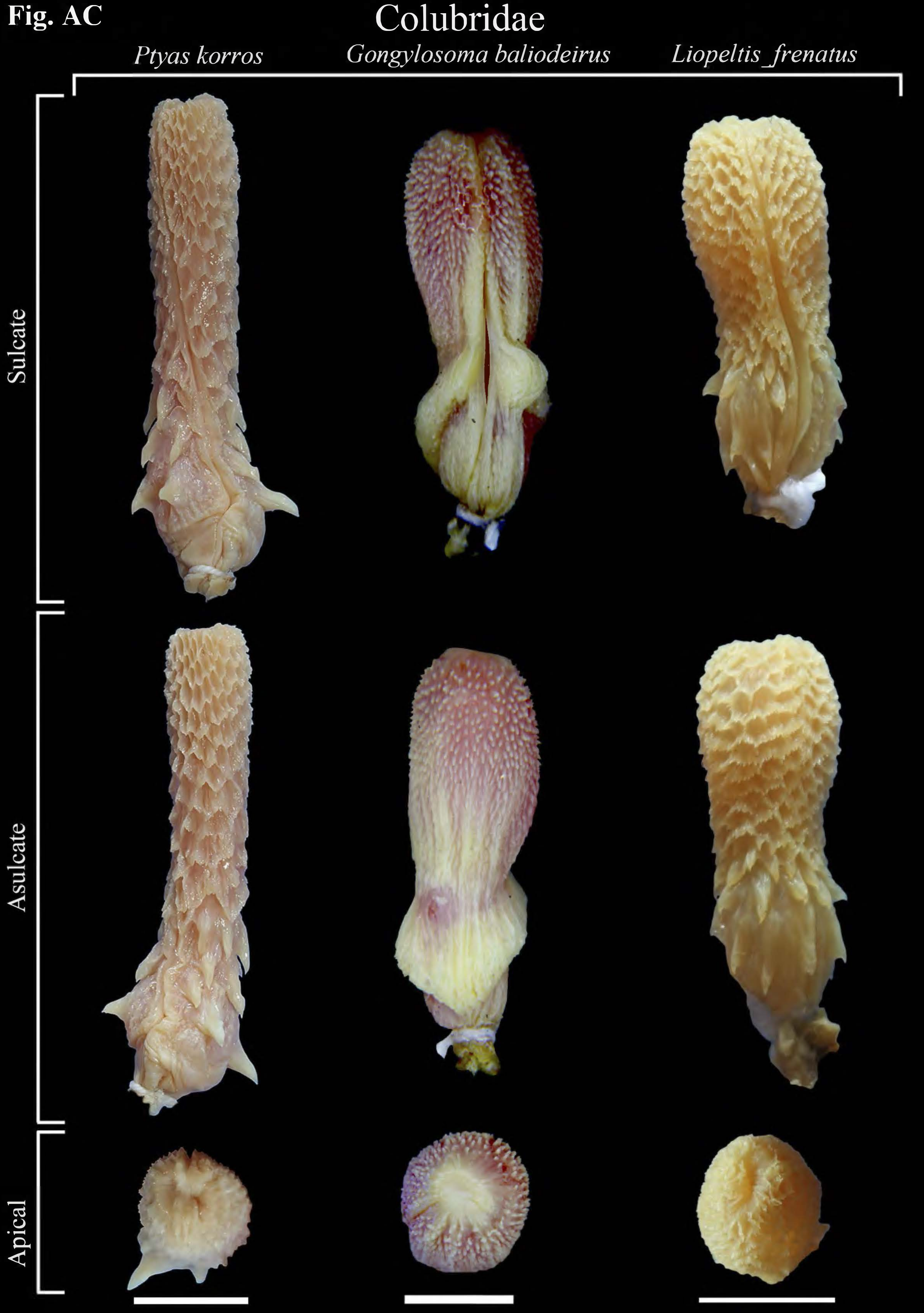

Colubridae
Ptyas korros
Gongylosoma baliodeirus
Liopeltis_frenatus
Sulcate
Asulcate
Apical

Fig. AD Elapoidea *Incertae sedis*

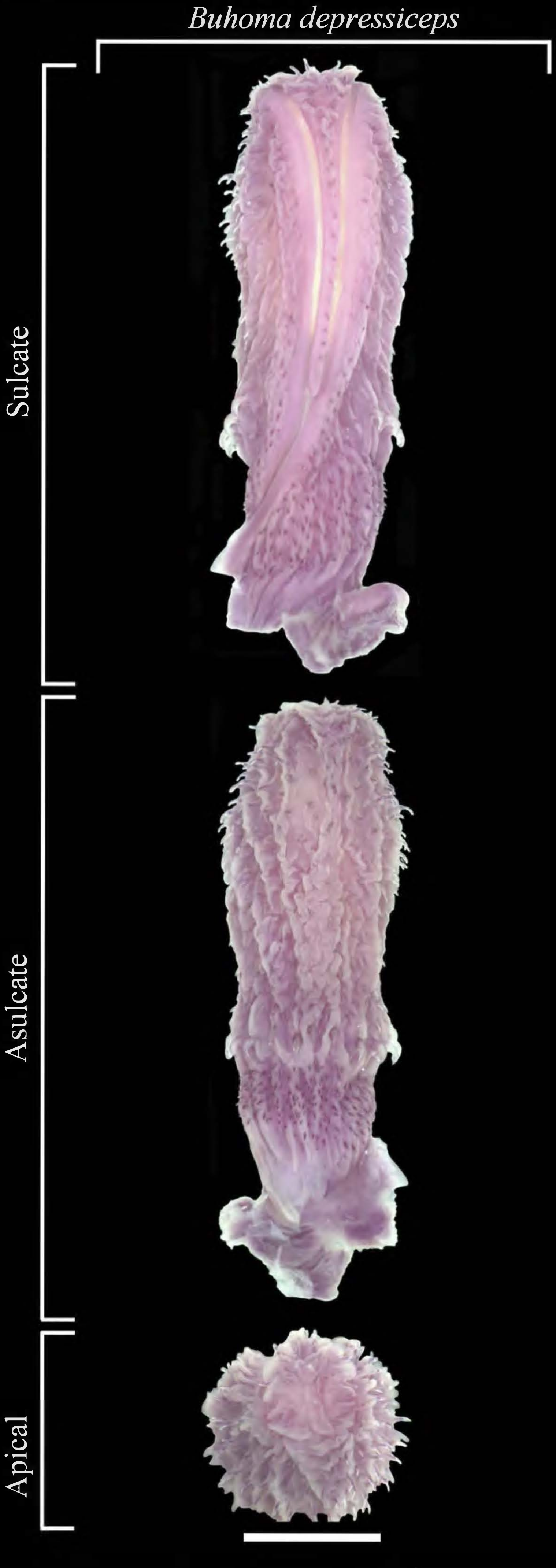

Supplement: S3 Appendix — Hemipenial morphology of representatives of colubroidean families. Figure A, Acrochordidae: Acrochordus javanicus (LSUMZ 34406) completely everted and filled, scale bar = 5 mm; Xenodermidae: Xenodermus javanicus (FMNH 138678) partially everted, partially filled, and dyed with alizarin red, scale bar = 1 mm. Figure B, Xenodermidae: Achalinus rufescens (BMNH 1983.193) completely everted and partially filled, scale bar = 2 mm; Fimbrios klossi (BMNH 1965.2.639) opened through a longitudinal slit, one lobe partially filled, scale bar = 1 mm; Pareidae: Pareas monticola (BMNH 1909.3.9.19) completely everted and filled, scale bar = 1 mm. Figure C, Pareidae: Asthenodipsas malaccanus (BMNH 1924.10.23.7) completely everted and filled; Aplopeltura boa (BMNH 94.6.30.63) completely everted and filled; scale bars = 2 mm. Figure D, Xylophiidae: Xylophis perroteti (BMNH 1955.1.3.10) opened through a longitudinal slit, spread flat, and dyed with alizarin red, scale bar = 5 mm. Figure E, Viperidae: Porthidum nasutum (MZUSP 7480) completely everted and filled; Vipera ammodytes (MZUSP 8223) completely everted and filled; scale bars = 5 mm. Figure F, Viperidae: Bothrops neuwiedi (MZUSP 11851) completely everted and filled; Causus bilineatus (MNHN 1993.5992) completely everted and filled; scale bars = 5 mm. Figure G, Homalopsidae: Homalopsis buccata (MNHN 1963.728) completely everted and filled; Brachyorrhos albus (FMNH 142324) completely everted and filled; scale bars = 5 mm. Figure H, Homalopsidae: Fordonia leucobalia (AMNH 107179) completely everted, filled, and dyed with alizarin red; Bitia hydroides (FMNH 229568) completely everted and filled; scale bars = 5 mm. Figure I, Homalopsidae: Erpeton tentaculatum (AMNH 8850) completely everted and filled, scale bar = 5 mm. Psammophiidae: Mimophis mahfalensis (UMMZ 209646) completely everted and partially filled, scale bar = 2 mm; Atractaspididae: Polemon christyi (FMNH 219912) completely everted and filled, scale bar = 5 mm. Figure J, Atrac [file pone.0216148.s017.pdf]
