# Supplementary material for: Large-scale molecular phylogeny, morphology, divergence-time estimation, and the fossil record of advanced caenophidian snakes (Squamata: Serpentes)
Source: PLoS One. 2019 May 10;14(5):e0216148. doi: 10.1371/journal.pone.0216148 (PMC6512042; doi:10.1371/journal.pone.0216148)

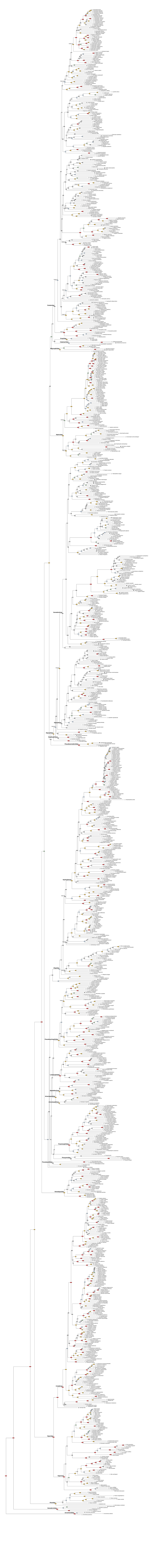

Supplement: S1 Fig — Maximum likelihood tree of Colubroides containing 1263 terminals. Color of the squares follow the categories of combined clade support as described in S2 Table. Numbers inside de squares on the nodes of the full tree represent the bootstrap and SHL values retrieved. Diamonds on each tip represent the percentage of the data for each terminal generated in this study: white, 0%; light gray, between 1% and 50%; dark gray, between 50% and 99%; black, 100%. Terminals in red represent additional samples in relation to Pyron et al. [26]. (PDF) [file pone.0216148.s011.pdf]
